# GC-MS Guided Phytochemical Fingerprinting and Multi-Target Therapeutic Evaluation of *Ixora chinensis* Lam. Leaves: Insights into Its Hypoglycemic and Analgesic Activities

**DOI:** 10.3390/biology15080592

**Published:** 2026-04-08

**Authors:** Joy Baisnab, Md. Saiful Islam, Md Reduanul Haque Kavey, S. M. Yasin Shourav, Md. Riaz Hosen, Md. Faysal Abid, Shaikh Shahinur Rahman, Anuwatchakij Klamrak, Arunrat Chaveerach, Sakda Daduang, Md. Rasul Karim

**Affiliations:** 1 Department of Pharmacy, Islamic University, Kushtia 7003, Bangladesh; joybaisnab.pharm.iu@gmail.com (J.B.); reduanul146@gmail.com (M.R.H.K.); riazhasan9146@gmail.com (M.R.H.); faysalabid5231@gmail.com (M.F.A.); 2 Pharmaceutical Sciences Research Division, BCSIR Dhaka Laboratories, Bangladesh Council of Scientific and Industrial Research (BCSIR), Dhaka 1205, Bangladesh; saifulislam@bcsir.gov.bd; 3 Department of Pharmaceutical Technology, Faculty of Pharmacy, University of Dhaka, Dhaka 1000, Bangladesh; shouravyasin27@gmail.com; 4 Department of Applied Nutrition and Food Technology, Islamic University, Kushtia 7003, Bangladesh; shahin@anft.iu.ac.bd; 5 Division of Pharmacognosy and Toxicology, Faculty of Pharmaceutical Sciences, Khon Kaen University, Khon Kaen 40002, Thailand; anuwat_kla@yahoo.com; 6 Department of Biology, Faculty of Science, Khon Kaen University, Khon Kaen 40002, Thailand; raccha@kku.ac.th

**Keywords:** analgesic activity, GC-MS, hypoglycemic, *Ixora chinensis*, molecular docking

## Abstract

*Ixora chinensis* Lam. has traditionally been used to treat conditions such as acne, high blood pressure, bleeding, tuberculosis, and rheumatism. This study aimed to investigate the methanolic extract of *I. chinensis* leaves to determine their bioactive compounds and evaluate their effects on both central and peripheral pain using in vivo and in silico approaches. The GC-MS analysis revealed 41 phytochemicals, including 14 phenolics, 4 esters, 12 terpenoids, 8 alkaloids, and 3 sulfur-containing compounds. In the glucose tolerance test, both the chloroform-soluble fraction (CF) and n-hexane fraction (NHF) exhibited *p* < 0.05 reductions in blood glucose levels at a dosage of 400 mg/kg with decreases of 51.94% and 46.63%, respectively, compared to the positive control (64.02%). The central analgesic evaluation showed significant (*p* < 0.001) enhancements in tail-flick latency for the fraction (184.94%) and CF (170.51%) following 90 min. In the pain relief assay, NHF showed inhibition (64.33%, *p* < 0.001) followed by an aqueous fraction (57.35%). These pharmacological findings were supported by in silico analysis. Concerning activity, 5-(dimethylamino)-1- acid phenyl ester (−8.9 kcal/mol) and 9,9-dimethyl-9H-fluoren-3-ol (−8.4 kcal/mol) displayed the strongest binding affinity to AMPK. Additionally, 2,3-diphenyl-2-cyclopropen-1-one exhibited favorable interactions with α-amylase (−8.0 kcal/mol) and α-glucosidase (−8.3 kcal/mol). Similarly, the central analgesic effect correlated with the strong μ-opioid receptor affinity of s-Triazine, 2-amino-4-(piperidinomethyl)-4-piperidino (−8.8 kcal/mol). N-Methyl-N-(4-toluenesulfonyl)-benzamide (−8.6 kcal/mol) and s-Triazine derivative (−8.9 kcal/mol) demonstrated notable COX-1 and COX-2 inhibition potential. Overall, the findings indicate *I. chinensis* leaves as a promising source of bioactive compounds with significant antihyperglycemic and analgesic properties.

## Simple Summary

In regions where modern drugs are inaccessible, diabetes and chronic pain illnesses continue to be treated with natural medicines. For many centuries, the flowering plant *Ixora chinensis* Lam. has been used to treat hypertension, abscesses, wounds, rheumatism, and bruises, making it a significant natural remedy. Our study sought to assess the leaves of *I. chinensis* for identifying potential phytochemicals by GC-MS that may reduce pain and/or blood sugar levels. In vivo study results showed that the *I. chinensis* leaves used as test substances exhibited hypoglycemic and analgesic activity, comparable to some standard drugs, in both central and peripheral pain models. In silico studies showed that the pain and glucose-modulating targets, i.e., proteins present in the body, were well caged by the medicinal plant compounds. Therefore, it was concluded that the leaves of *I. chinensis* are of great importance, and the potential for pain amelioration and blood glucose level reduction is substantial. The biological activity of the plant is used to evidence the traditional knowledge, and the herbal medicine *I. chinensis* is naturally and therapeutically valuable, which can help healthcare systems by assisting in the construction of more herbal medicines.

## 1. Introduction

Medicinal and herbal plants are used across various scientific and industrial sectors, playing an important role in food production, food technology, cosmetics, and healthcare [1]. Worldwide fascination with medicine is increasing due to its availability, accessibility, and perceived safety [2]. Furthermore, synthetic tropical therapies are generally more expensive and have more evidence of side effects than natural products [3]. Again, synthetic therapies need a high-definition healthcare infrastructure and distribution channel, which are not easily manageable for the people of low-income countries. Such factors encourage individuals to prefer natural remedies to synthetic drugs.

Diabetes mellitus is a progressive disorder characterized by persistently high blood glucose concentrations. Although antihyperglycemic drugs are accessible, many are limited because of side effects and prolonged toxicity [4]. Consequently, various research efforts employing strategies that focus on natural sources such as plants, foods, and microorganisms aim to identify safer, more effective therapies for managing diabetes [5,6].

Pain is an experience shared by humans and animals. It arises in certain conditions and can evolve into a persistent, disabling condition that adversely affects quality of life [7]. Because pain can result from tissue damage or disruptions in the nervous system, effective pain control remains a major challenge for global public health [8]. Synthetic analgesic medications cause negative impacts on kidney, liver, and heart functions [9]; hence, current studies have focused on sources to reduce the side effects associated with synthetic analgesic drugs.

*Ixora chinensis* Lam. (Rubiaceae) is native to China and Malaysia and is found in southeastern China [10]. It is widely cultivated worldwide for its numerous vibrant flowers and dense, multi-branched evergreen habit [11]. Recent studies on extracts derived from the leaves and flowers of *I. chinensis* have reported mild to moderate antimicrobial activity against several bacterial and fungal strains [12]. The plant leaf extract demonstrated notable in vitro anticancer activity against breast (MCF-7) and colon (CACO-2) cancer cell lines [13]. Again, in vitro antidiabetic properties support their traditional use in the management of diabetes [12]. Alcohol-based extracts from *I. chinensis* leaves exhibited liver-related effects in animal research and in vitro antioxidant activity [14]. The previously reported preliminary phytochemical examination suggested that *I. chinensis* is a promising source of steroids, triterpenoids, flavonoids, and alkaloids. Chemical analysis revealed the existence of rubiothiagepine, ixorene, oleanolic acid, quercetin 3-o-rhamnoside, catechin, and kaempferitrin from various solvent extracts of the species [15]. The different parts of the plant also have evidence of the presence of β-sitosterol, D-mannitol, (10E)-9-oxo-octadec10-en-12-ynoic acid, stearic acid, azelaic acid, dihydro masticadienolic acid, 1, 5- cyclooctadiene and liphatic acid [16]. Again, the presence of glycosides in the plant species made it a prominent species among the Rubiaceae family [17].

This study aims to analyze the phytochemical constituents of the methanolic crude extract of *I. chinensis* leaves via GC-MS analysis and to investigate the hypoglycemic, central analgesic, and peripheral analgesic properties of the different solvent fractions of the methanolic extract using in vivo methods. This study also aims to perform multi-target molecular docking to track the mechanistic pathways of the bioactive phytochemicals, probably responsible for the hypoglycemic and analgesic effects.

## 2. Materials and Methods

### 2.1. Plant Sample Collection and Authentication

Fresh leaves of *I. chinensis* were gathered from Chittagong, Bangladesh. Identification was verified by a botanist at the Institutional Herbarium (Accession No. JUH-10271). The leaves were air-dried at room temperature for several days, then warmed to 40 °C. The dried leaves were then crushed into a powder. The powder was stored in airtight containers until extraction.

### 2.2. Extraction and Fractionation

A 500 g powder sample was macerated in 2.5 L of methanol at room temperature for 14 days with occasional agitation. The mixture was filtered through a sterile cotton mesh and a Whatman No. 1 filter paper (Whatman International Ltd., Maidstone, UK). The filtrates were concentrated to a semisolid crude extract using a rotary evaporator at temperatures below 50 °C, and no additional processing steps, such as lyophilization, were performed. Subsequently, 5 g of the methanolic extract was fractionated using n-hexane, chloroform, ethyl acetate, and water, following a modified Kupchan partitioning technique [18]. The fractionation was performed according to the increasing order of the solvent polarity. A total of 300 mL of solvent, repeated three times (3 × 100 mL), was used for each fractionation step. The yields of the respective fractions were 32.45%, 16.77%, 24.07%, and 26.71%. The resulting crude fractions were designated as the n-hexane fraction (NHF), the chloroform-soluble fraction (CF), the ethyl acetate-soluble fraction (EAF), and the aqueous-soluble fraction (AQF).

### 2.3. Drugs and Chemicals

All reagents used in the whole study were of analytical grade. Methanol, n-hexane, chloroform, ethyl acetate, Tween-80, and glucose were sourced from BDH chemicals Ltd (UK). The saline solution was acquired from Beximco Pharmaceuticals Ltd. (Dhaka, Bangladesh). Additionally, morphine (Gonoshastho Pharmaceuticals Ltd., Dhaka, Bangladesh), diclofenac sodium, and metformin hydrochloride (Square Pharmaceuticals Ltd., Pabna, Bangladesh) served as reference medications.

### 2.4. Phytochemical Analysis

- Gas chromatography-mass spectrometry (GC-MS) analysis

The GC-MS investigation was performed utilizing an 8890 Gas Chromatography System (Agilent 19091S-433UI:2489267H USA, Agilent Technologies, Santa Clara, CA, USA) linked with an Agilent 7010B GC/TQ MS instrument. Using an HP-5MS UI fused silica column (5% phenyl methyl siloxane 30 m × 250 μm × 0.25 μm), helium was the carrier gas at a flow rate of 1.72 mL/min. The oven temperature was maintained at 120 °C for 2 min, then increased to 320 °C at a rate of 10 °C/min, where it was kept for 5 min. The ion source temperature was established at 280 °C. The injection volume was 1.0 μL (with a hold time of 5.00 min), the injector temperature was 250 °C, and the split ratio was 20:1. Then, an electron ionization was performed at 70 eV, and mass spectra were gathered from 30 *m*/*z* to 550 *m*/*z* during 24 min. The overall duration was 27.0 min, including a solvent cut period of 3.0 min. The phytochemicals in the samples were recognized by matching their retention times and mass spectra with the 2020 National Institute of Standards and Technology (NIST) database. In GC-MS analysis, the compound identification based on library spectral matching is exploratory and hypothetical.

### 2.5. Experimental Animals

Swiss Albino mice of either sex (weighing between 32 and 38 g and aged 4–5 weeks) were utilized for analgesic evaluations. The mice were maintained under the protocols (24.0 ± 1 °C, 55–65% relative humidity, and a 12 h light/dark cycle) established by the Federation of European Laboratory Animal Science Associations (FELASA).

- Ethical approval and method of sacrifice

All experimental procedures involving animals were authorized by the Biosafety, Biosecurity and Ethical Committee of the University of Dhaka (Ref No: 270/Biol.Scs./Sep-2024). Following the completion of the experiment, mice were euthanized by administering a dose of ketamine (100 mg/kg) and xylazine (7.5 mg/kg) as per Zimmermann’s protocol [19].

- Acute toxicity test

The acute toxicity assessment of *I. chinensis* was conducted in accordance with the OECD (Organization for Economic Co-operation and Development) Test No. 423: Acute Oral Toxicity Acute Toxic Class Method (2002), with slight adjustments [20]. The extract was administered orally at concentrations of 200, 400, 800, 1600, and 3200 mg/kg [21]. Before the administration of the extract, all mice fasted for 16 h. Following treatment, the mice were monitored continuously for 1 h, then at intervals for the next 4 h, and finally observed for 24 h to identify any behavioral changes, signs of toxicity, or mortality. The animals were additionally monitored for the time to death over 14 days [22]. The LD_50_ values of the fractions were calculated.

### 2.6. Preparation of Oral Doses

All experimental crude fraction doses (200 mg/kg and 400 mg/kg) and standard drugs, viz., metformin hydrochloride, diclofenac sodium, and morphine, were prepared as oral suspensions with normal saline (0.9% NaCl), and 1% Tween-80 was used as a suspending agent. The formulated oral experimental crude doses were expressed as NHF-200 and NHF-400 for the n-hexane-soluble fraction, CF-200 and CF-400 for the chloroform-soluble fraction, EAF-200 and EAF-400 for the ethyl acetate-soluble fraction, and AQF-200 and AQF-400 for the aqueous-soluble fraction. Metformin hydrochloride (20 mg/kg), diclofenac sodium (10 mg/kg), and morphine (5 mg/kg) were used as positive standards [23].

### 2.7. Hypoglycemic Activity Test

- Oral glucose tolerance test (OGTT)

The hypoglycemic activity of *I. chinensis* leaves extract was evaluated by oral glucose tolerance test [24]. Blood glucose levels of mice (*n* = 6) in each category (control, positive control, and test groups) were initially checked with a glucometer by drawing blood from the tail vein. Subsequently, all groups received a 10% glucose solution (2 g/kg) orally to trigger hyperglycemia, and blood glucose was measured 30 min afterward. The negative control group was orally administered a 1% Tween-80 solution mixed with saline, whereas positive controls were given metformin hydrochloride (20 mg/kg), and the test groups were treated with crude fractions at doses of 200 mg/kg and 400 mg/kg orally. Blood glucose levels, for each group, were measured at 60, 120, and 180 min to calculate the percentage of glucose reduction and evaluate the effect.

$$\%\;glucose\;reduction=\frac{BGL30-BGLtime}{BGL30}\times 100$$

Here, BGL30 is the average blood glucose level for any group at 30 min, and BGLtime is the average blood glucose level for that group at other time points.

### 2.8. Analgesic Activity Test

- Tail-flicking test

The central pain-relieving action of *I. chinensis* leaves was assessed using the tail immersion method [25]. The negative control group of mice received a blank oral suspension of 1% Tween-80 in normal saline at 10 mL/kg body weight, and the positive control group received morphine subcutaneously at 2 mg/kg body weight. The test group mice received two doses of each crude fraction of the leaves (200 and 400 mg/kg of body weight). After 30 min of administration of the design doses, the tip of the tail (5 cm) from each group was submerged in warm water of 50 °C ± 0.5 °C to elicit pain. The tail immersion times, the amount of time it took for each mouse to move their tail after receiving the extract or drug, were measured at 30, 60, and 90 min. The percent time elongations for each group were measured at 30, 60, and 90 min by the following equation.

$$\%\;time\;elongation=\frac{Tt-Tc}{Tc}\times 100$$

Here, Tt is the average tail immersion time in the positive control and test groups, and Tc is the average tail immersion time in the control group.

- Acetic acid-induced writhing test

The acetic acid-induced writhing method was applied to explore the peripheral analgesic properties of *I. chinensis* leaves extract [26]. At first, the positive control group was administered diclofenac sodium orally at a dosage of 10 mg/kg, and the test groups received oral crude fractions at doses of 200 and 400 mg/kg body weight. A blank oral suspension of 1% Tween-80 in normal saline at a dose of 10 mL/kg of body weight was given to the negative control group. After 30 min, a 0.7% acetic acid solution at a dose of 0.2 mL was given intraperitoneally to induce peripheral pain [27]. Then, writhing, evidence of peripheral pain, was monitored and documented for five minutes following the five minutes of acetic acid injection [28]. The formula for determining the percentage of inhibition of writhing was as follows:

$$\%\;of\;inhibition=\frac{Writhing\;response\;of\;control\;group-Writhing\;response\;of\;test\;group}{Writhing\;response\;of\;control\;group}\times 100$$

### 2.9. In Silico Study

- Molecular docking

The crystal structures of cyclooxygenase-1 (PDB ID: 3N8Y) and cyclooxygenase-2 (PDB ID: 5F19) were selected as peripheral analgesic targets, whereas the μ-opioid receptor (PDB ID: 5C1M) was used as the central analgesic target. For evaluating hypoglycemic potential, the AMPK complex structure (PDB ID: 4RER), α-amylase (PDB ID: 1HNY), and α-glucosidase (PDB ID: 3TOP) were included. All protein structures were retrieved from the RCSB Protein Data Bank (https://www.rcsb.org/) and prepared by removing crystallographic water molecules and non-essential heteroatoms using PyMol [29]. The phytoconstituents identified from the GC-MS analysis were downloaded from the PubChem database and subjected to energy minimization using the MMFF94 force field in PyRx v0.8 [30,31]. The minimized ligands were converted to PDBQT format and used for blind docking with AutoDock Vina v1.1.2 integrated in the PyRx v0.8 software [32]. Binding affinities (kcal mol^−1^) were recorded for each ligand–protein complex, and the best binding poses were analyzed using Discovery Studio Visualizer 2020 20.1.0 and UCSF Chimera to identify key interacting residues.

- Pharmacokinetic and toxicity evaluation

The pharmacokinetic behavior, drug-likeness, and toxicity profiles of the selected ligands were assessed using online prediction tools. ADME parameters such as molecular weight, hydrogen-bond donors and acceptors, TPSA, lipophilicity (LogP), gastrointestinal absorption, blood–brain barrier permeability, and compliance with Lipinski’s rules were predicted using the SwissADME server (http://www.swissadme.ch) [33,34]. Toxicity endpoints, including hepatotoxicity, mutagenicity, cytotoxicity, immunotoxicity, and overall toxicity class, were evaluated using the ProTox-3.0 platform (https://tox.charite.de/protox3/), accessed on 9 November 2025 [35].

### 2.10. Statistical Methods

The observational data, derived from in vivo studies, were displayed as mean values accompanied by their corresponding standard error. To ascertain the presence of statistically significant differences between test samples and negative control groups, the *p*-value for each tested group was computed by a one-way ANOVA test. As the analysis focused on the overall group effect, no post hoc test was performed. If the *p*-value is less than 0.05, the differences between the control and test groups are thought to be statistically significant.

## 3. Results

### 3.1. Phytochemicals from GC-MS Analysis

**Figure 1:**
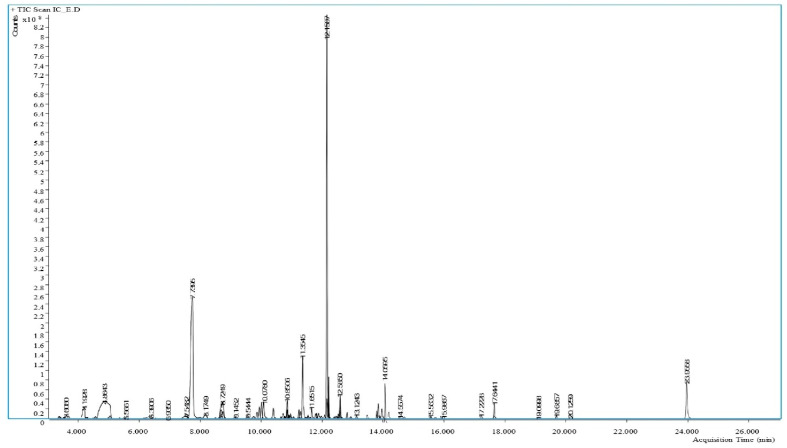
GC-MS chromatogram of the methanolic extract of *I. chinensis* leaves.

A wide range of phytochemicals was tentatively identified based on the library matching through the GC-MS analysis of the methanolic extract of *I. chinensis* leaves. A total of 41 phytochemicals were identified, and their structures were confirmed by comparing the specific parameters, such as retention times, fragmentation patterns, molecular weights, and match factors of the compounds with the NIST library database (Supplementary Materials, File S1). Figure 1 shows the GC-MS chromatogram along with the retention times. Among all the compounds we detected, 8 belong to alkaloids (Table 1), 14 are categorized as phenolics (Table 2), 4 are esters (Table 3), 12 are classified as terpenoids (Table 4), and 3 contain sulfur (Table 5). The alkaloids include subclasses such as pyrazole, aromatic amine, sulfonamide, triazole, furan-triazole, piperidone, and isoquinoline. The majority of substances are part of the phenolic acid category, and we also identified a combination of esters and terpenoids. This varied assortment of phytochemicals emphasizes the metabolic abundance in *I. chinensis* leaves.

### 3.2. Acute Toxicity Test

Oral administration of crude methanol extract at concentrations of 200, 400, 800, 1600, and 3200 mg/kg did not result in any observed signs of toxicity or deaths within the first 48 h, indicating that the oral LD_50_ exceeds these doses. Physical and behavioral examinations of the experimental mice revealed no signs of acute poisoning, including vomiting, diarrhea, ataxia, piloerection, and loss of consciousness.

### 3.3. Hypoglycemic Activity

**Figure 2:**
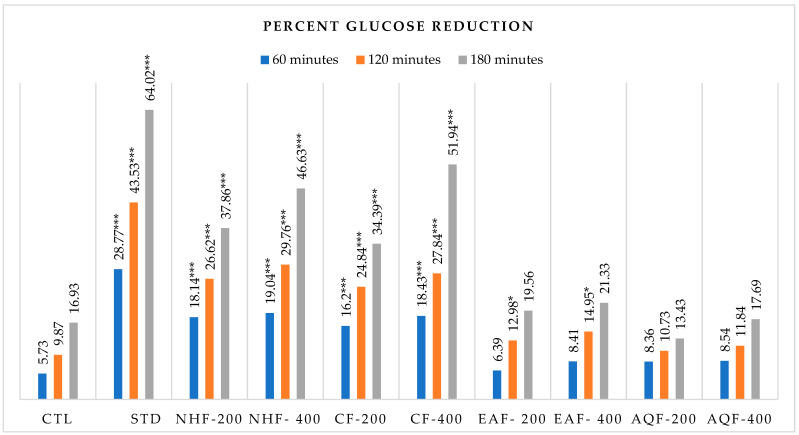
Percent reduction in blood glucose level with time. * *p* < 0.05; *** *p* < 0.001.

The oral glucose tolerance test (OGTT) was performed to evaluate the plant extract’s ability to lower blood glucose levels at various dosages, using metformin hydrochloride as the standard, and plasma glucose levels were measured at 60, 120, and 180 min. This study found that both 200 mg/kg and 400 mg/kg body weight dosages of CF and NHF exhibited significant (*p* < 0.05) outcomes (Table 6). After 180 min, blood glucose levels were reduced by 51.94% and 46.63%, respectively, for the 400 mg/kg body weight dosages of CF and NHF, which were comparable to the 64.02% decrease in blood glucose level measured with the positive control medication metformin hydrochloride shown in Figure 2. At the same time, reductions of 34.39% and 37.86% were noted for the 200 mg/kg doses of CF and NHF (Figure 2).

### 3.4. Analgesic Activity

#### 3.4.1. Tail-Flicking Test in Mice

The various fractions of the methanolic extract of *I. chinensis* exhibited significant central analgesic effects by prolonging the tail deflection time in Swiss albino mice (Table S8). The percentage elongation times were observed at 30-, 60-, and 90-min post-administration intervals in Swiss-Albino mice, as indicated in Table 7. In our studies, mice administered morphine exhibited increases in response time, reaching 171.47%, 274.23%, and 312.84% after 30, 60, and 90 min, respectively, all showing strong statistical significance (*p* < 0.001). Among the tested fractions, the aqueous (AQF) and chloroform (CF) fractions at a dose of 400 mg/kg produced pronounced effects. At the 90 min mark, AQF demonstrated a 184.94% increase while CF showed a 170.51% increase, both significant in duration. At doses of 200 mg/kg, both AQF and CF fractions produce moderate analgesic activity with 117.767% (*p* < 0.001) and 114.70% (*p* < 0.001) after 90 min. Except for the ethyl acetate fraction (EAF) and n-hexane (NHF) fraction at 200 mg/kg and 400 mg/kg of body weight dose, all other fractions revealed significant (*p* < 0.001) central analgesic activity after 90 min, compared to the control group.

#### 3.4.2. Acetic Acid-Induced Writhing Test in Mice

In the peripheral analgesic activity assay, diclofenac sodium was selected as a positive control. Compared to the control group, the standard drug inhibited 69.77% (*p* < 0.001) of writhing (Table 8, Table S9). The n-hexane fraction (400 mg/kg) showed the strongest analgesic activity (64.33% inhibition), followed by aqueous activity (57.35%). At 200 mg/kg, moderate effects were observed with NHF (27.11%) and AQF (19.40%) (Table 8). In contrast, chloroform and ethyl acetate at both doses do not produce any peripheral analgesic activity, as indicated in Table S9 (Supplementary Materials, File S2).

### 3.5. Molecular Docking

**Figure 3:**
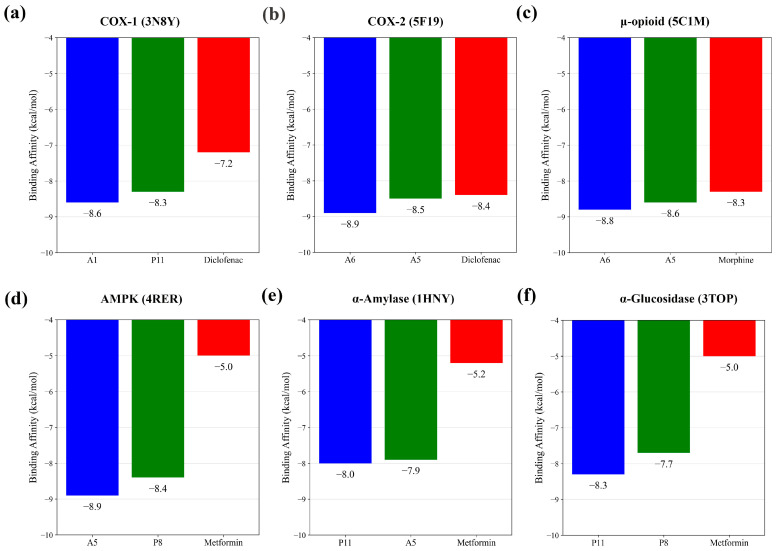
Comparative binding affinities of selected phytoconstituents of *I. chinensis* against analgesic and hypoglycemic targets.

Molecular docking was performed for all GC-MS-identified phytoconstituents of *I. chinensis* (Table 1, Table 2, Table 3, Table 4 and Table 5) to evaluate their predicted binding potential toward peripheral analgesic, central analgesic, and hypoglycemic targets. For the peripheral analgesic targets, compounds A1 and P11 showed predicted binding affinities of −8.6 and −8.3 kcal/mol, respectively, toward COX-1, compared with −7.2 kcal/mol for diclofenac. At the COX-2 target, compound A6 exhibited a docking score of −8.9 kcal/mol, while P11 showed −8.5 kcal/mol, both of which were comparable to the binding affinity observed for the reference drug diclofenac sodium (−8.4 kcal/mol) under the applied docking condition. For the μ-opioid receptor, compounds A6 (−8.8 kcal/mol) and A5 (−8.6 kcal/mol) showed predicted binding affinities comparable to morphine (−8.3 kcal/mol). Upon examining the hypoglycemic targets, A5 stood out with a binding affinity of −8.9 kcal/mol against AMPK, followed by P8 at −8.4 kcal/mol, while the reference drug metformin displayed a lower docking score (−5.0 kcal/mol). Comparable trends were observed for α-amylase and α-glucosidase, where P11 and A5 showed predicted binding scores of −8.0 and −7.9 kcal/mol, respectively, against α-amylase, compared to metformin’s −5.2 kcal/mol. Similarly, P11 and compound P8 exhibited predicted docking scores of −8.3 and −7.7 kcal/mol, respectively, with α-glucosidase, while metformin again showed a lower binding affinity (−5.0 kcal/mol). The complete docking scores of all screened phytocompounds against each target protein are provided in Table S1 (Supplementary Materials, File S2). Figure 3 summarizes the binding affinities of top-performing phytocompounds across the six molecular targets.

**Figure 4:**
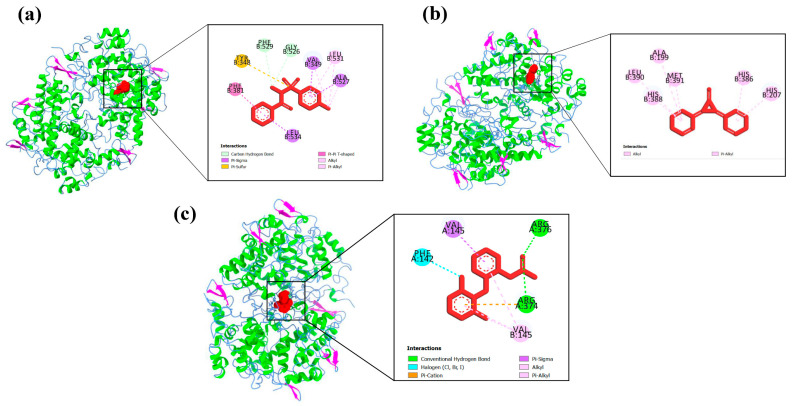
Binding interactions of (**a**) A1 (N-Methyl-N-(4-toluensulfonyl)-benzamide) with GLY526, PHE529, VAL349, TYR348, PHE381, ALA527, LEU531, and LEU534 amino acid residues, (**b**) P11 (2,3-diphenyl-2-Cyclopropen-1-one) with ALA199, LEU390, MET391, HIS207, HIS386, and HIS388 amino acid residues, and (**c**) diclofenac sodium (Standard drug) with PHE142, VAL145, ARG374 and ARG376 amino acid residues of the active site of COX-1 (3N8Y).

**Figure 5:**
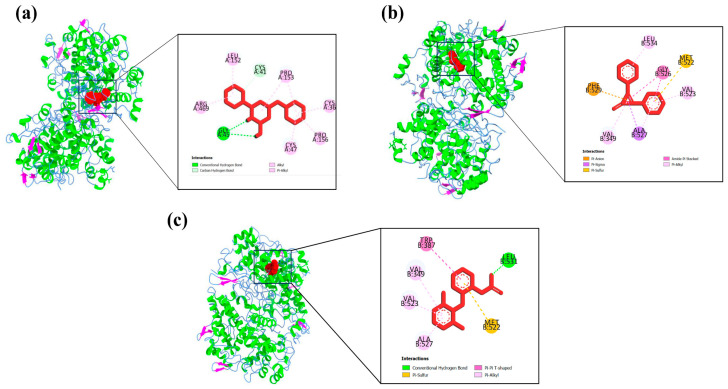
Binding interactions of (**a**) A6 (s-Triazine, 2-amino-4-(piperidinomethyl)-4-piperidino-) with GLY45, CYS41, CYS36, CYS47, LEU152, PRO153, PRO156, ARG469 amino acid residues, (**b**) P11 (2,3-diphenyl-2-Cyclopropen-1-one) with PHE529, ALA527, VAL349, VAL523, LEU534, GLY52, and MET522 amino acid residues, and (**c**) diclofenac sodium (Standard drug) with LEU531, TRP387, VAL349, VAL523, ALA527, and MET522 amino acid residues of the active site of COX-2 (5F19).

**Figure 6:**
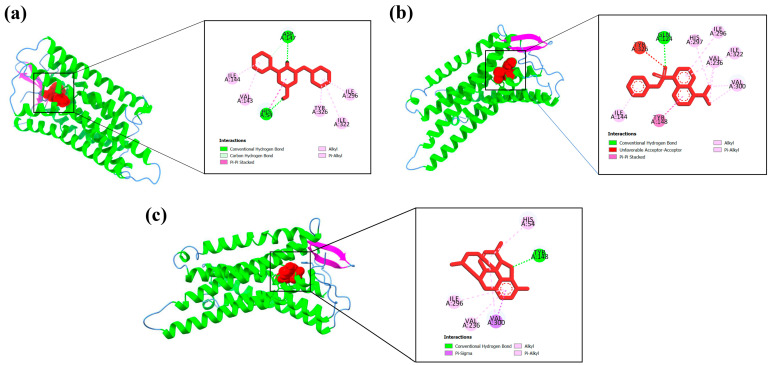
Binding interactions of (**a**) A6 (s-Triazine, 2-amino-4-(piperidinomethyl)-4-piperidino-) with HIS54, ASP147, VAL143, ILE144, ILE296, ILE322, TYR326 amino acid residues, (**b**) A5 (5-(dimethylamino)-1-Naphthalenesulfonic acid phenyl ester) with GLN124, VAL236, ILE296, ILE322, ILE144, and TYR148 amino acid residues, and (**c**) morphine (standard drug) with TYR148, VAL300, VAL236, ILE296, and HIS54 amino acid residues of the active site of μ-opioid (5C1M).

**Figure 7:**
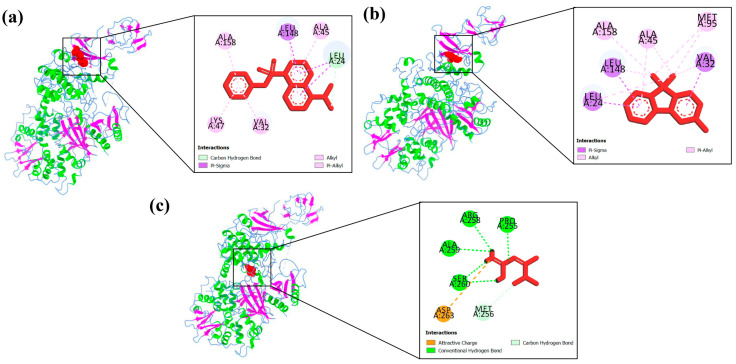
Binding interactions of (**a**) A5 (5-(dimethylamino)-1-Naphthalenesulfonic acid phenyl ester) with LEU24, LEU148, ALA45, VAL32, LYS47, and ALA158 amino acid residues, (**b**) P8 (9,9-dimethyl-9H-Fluoren-3-ol) with LEU24, VAL32, LEU148, MET95, ALA158, and ALA45 amino acid residues, and (**c**) metformin hydrochloride (Standard drug) with SER260, ARG258, ALA259, PRO255, MET256, and ASP263 amino acid residues of the active site of AMPK (4RER).

**Figure 8:**
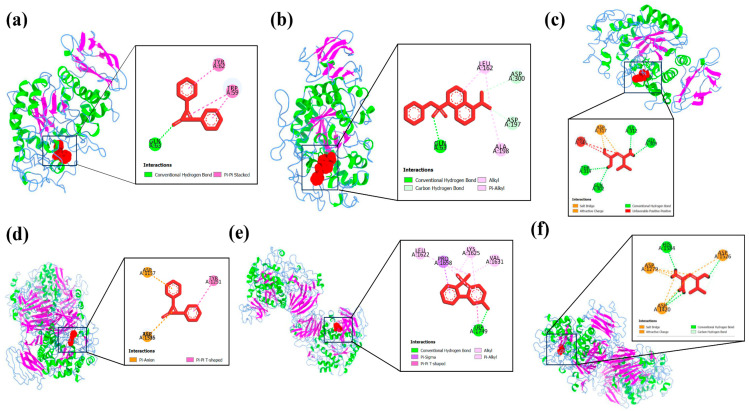
Binding interactions of (**a**) P11 (2,3-diphenyl-2-Cyclopropen-1-one) with GLN63, TRP59, and TRP62 amino acid residues, (**b**) A5 (5-(dimethylamino)-1-Naphthalenesulfonic acid phenyl ester) with GLN63, ASP300, ASP197, LEU162, and ALA198 amino acid residues, (**c**) metformin hydrochloride (standard drug) with GLY309, GLN302, THR314, ILE312, and ASP317 amino acid residues of the active site of α-amylase (PDB ID: 1HNY), (**d**) P11 (2,3-diphenyl-2-Cyclopropen-1-one) with ASP1157, ASP1526, and TYR1251 amino acid residues, (**e**) P8 (9,9-dimethyl-9H-Fluoren-3-ol) with TRP1749, LYS1625, LEU1622, VAL1631, and PRO1658 amino acid residues, and (**f**) metformin hydrochloride (standard drug) with ASP1526, ASP1584, ASP1420, and ASP1279 amino acid residues of the active site of α-glucosidase (PDB ID: 3TOP).

Protein–ligand interaction profiles of the highest-scoring ligand for each target protein are presented in Figure 4, Figure 5, Figure 6, Figure 7 and Figure 8, while the detailed molecular interaction data for all complexes are provided in Tables S2–S7 (Supplementary Materials, File S2).

### 3.6. Drug Likeness and Pharmacokinetic Parameters Analysis

The top five docked phytoconstituents, A1, A5, A6, P8, and P11, were subjected to pharmacokinetic and toxicity analyses alongside the standard drugs diclofenac sodium, morphine, and metformin hydrochloride. The detailed results are summarized in Table 9. The phytoconstituents had molecular weights ranging from 206.24 to 327.4 g/mol, LogP values between 2.32 and 2.98, and TPSA values of 17.07–71.17 Å^2^. In comparison, the reference drugs had molecular weights of 296.15 g/mol (diclofenac sodium), 285.34 g/mol (morphine), and 129.16 g/mol (metformin hydrochloride); LogP values of 1.98, 2.55, and 0.34; and TPSA values of 49.33, 52.93, and 91.18 Å^2^, respectively. SwissADME predictions suggested high gastrointestinal absorption for all compounds, with blood–brain barrier permeability predicted for all except A6 and metformin hydrochloride. However, compounds A1, A5, P8, and P11 were predicted to be BBB-permeable based on computational models; this is interpreted as a possibility rather than secure evidence of brain access. None of the tested compounds, including the reference drugs, violated Lipinski’s rules. According to ProTox-III, A1 was classified as Toxicity Class 6, A5, P11, and P8 as Class 4, and A6 as Class 5. Diclofenac sodium was classified as Class 3, and both morphine and metformin hydrochloride were classified as Class 4. Hepatotoxicity was predicted only for diclofenac sodium, and all other compounds were inactive for hepatotoxicity, mutagenicity, and cytotoxicity. An immunotoxicity alert was observed only for A5.

## 4. Discussion

Secondary metabolites derived from plants play an essential role in supplying pharmacologically active substances that can be utilized to develop new drug candidates [36]. These plant-produced secondary metabolites can be classified into various chemical groups, including alkaloids, saponins, glycosides, terpenoids, and phenolics [37].

This study confirms that *I. chinensis* leaves contain several alkaloids belonging to the triazole, pyrazole, isoquinoline, piperidine, and aromatic amine subclasses. The presence of different alkaloids directly bridges the present study with the previous reported alkaloid confirmation of the plant [15]. Among these alkaloids, etidocaine is an amide-type local anesthetic and also acts as a voltage-gated sodium channel blocker [38]. Though other alkaloids lack established pharmacological properties, the furan-triazole carboxamide and the piperidino-s-triazine derivative may exhibit fungicidal and antiviral activity owing to their chemical structures [39,40]. The present study also confirmed that the *I. chinensis* leaf extract contains numerous phenolics and terpenoids, which are primarily responsible for reducing free radicals generated by oxidative stress [41]. The presence of phenolics and terpenoids in the plant was supported by previous reports [15]. But the direct identification of the phenolics without derivatization in the GC-MS analysis is questionable due to their non-volatile nature. The phenolics identified in the study may be relatively volatile, low-molecular-weight, or thermally stable derivatives that can be identified under the applied GC-MS conditions. Among these phenolics and terpenoids, Loliolide [42]; phytol [43]; dl-Menthol; 2-Methyl-3-(3-methyl-but-2-enyl)-2-(4-methyl-pent-3-enyl)-oxetane [44]; 1-(2-Hydroxyphenyl)-1-butanone [45]; 2,4-Di-tert-butylphenol (DTBP) [46]; and Benzenepropanoic acid, 3,5-bis(1,1-dimethylethyl)-4-hydroxy-, methyl ester [47] have demonstrated antioxidant properties. This connectivity supports the previous report on the antioxidant activity of *I. chinensis* [48]. Additionally, 1-(2-Hydroxyphenyl)-1-butanone; N-(dimethylcarbamoylmethyl)-o-Acetophenetidide; 2,4-Di-tert-butylphenol; p-Octylacetophenone; and 1,4-Benzenediol, 2-methyl-, 4-acetate exhibit an anti-inflammatory effect as established by earlier studies [49,50].

Sulfur-containing phytochemicals are an important indicator that *I. chinensis* leaves have additional pharmacologically active components. Analgesic, antipyretic, cytotoxic, hepatoprotective, contraceptive, antidiabetic, and antimicrobial effects can be produced by sulfur-containing phytochemicals [51]. For example, sulfurous acid ester derivatives found in the plant under study have been shown to have hepatoprotective and analgesic properties [52].

Plant-derived secondary metabolites are a vital source of drug candidates to manage type 2 diabetes [53]. In the study, the OGTT method was applied to assess the blood glucose-lowering capability of the solvent fractions of the plant. The findings indicate that the comparatively non-polar fractions of the plant extract exerted more potent antihyperglycemic activity at the end of the study period. The past evidence of in vitro antidiabetic activity of the *I. chinensis* strengthens the outcomes of the present in vivo and in silico study on antidiabetic properties [12]. The n-hexane and chloroform soluble fractions exhibited statistically significant outcomes at both higher and lower doses. The polar fractions, such as ethyl acetate and aqueous soluble fractions, showed very little response, which was not statistically significant compared to the control group.

The central analgesic activity may be obtained when any chemical substance can bind with the Mu-opioid receptor of the central nervous system [54]. For these, the compound must have the ability to cross the blood–brain barrier, and the compounds are mainly lipophilic non-polar compounds. In the present study, non-polar chloroform-soluble fractions of the mother extract showed greater response than other fractions at both 200 and 400 mg/kg body weight doses. Although no previous reports of analgesic activity were available for the plant species, other species of the ixora genus have ethnopharmacological uses in pain management [14].

In the acetic acid-induced writhing test, the experimental mice exhibit writhing as a symptom of peripheral pain. The two cyclooxygenase (COX) enzymes, COX-1 and COX-2, produce prostaglandins, which are mainly responsible for inducing pain [55]. In this study, the peripheral analgesic effect of *I. chinensis* leaves was observed for both polar (AQF) and non-polar (NHF) fractions at both doses, with a response that reached statistical significance (*p* < 0.05). Thus, the plant extract may contain bioactive phytochemicals that may inhibit COX-1 and COX-2 like NSAIDs.

The findings of the in vivo studies are aligned with ethnobotanical uses and reported pharmacological activities of the different species of the Rubiaceae family. This family contains alkaloids, flavonoids, glycosides, terpenoids, volatile oils, and phenols [56]. The present chemical study is fully aligned with the reported studies in terms of broad chemical classes. Crude extracts and compounds derived from the Rubiaceae family have demonstrated a wide range of pharmacological effects, including antibacterial, antihypertensive, antidiabetic, antioxidant, and anti-inflammatory properties, as observed in biological screenings based on leads from traditional healers [56].

Backing up these results, the in silico molecular docking study offered additional proof pinpointing the particular phytoconstituents probably accountable for the noted antihyperglycemic and analgesic effects [57]. Regarding impact, 5-(dimethylamino)-1-Naphthalenesulfonic acid phenyl ester (A5) and 9,9-dimethyl-9H-fluoren-3-ol (P8) exhibited the highest binding affinity to AMPK, and these interactions might be functionally significant considering AMPK’s key role in glucose metabolism [58]. Additionally, 2,3-diphenyl-2-cyclopropen-1-one (P11) and 5-(dimethylamino)- phenyl ester (A6) showed favorable interactions with α-amylase, while 2,3-diphenyl-2-cyclopropen-1-one (P11) and compound 9,9-dimethyl-9H-Fluoren-3-ol (P8) demonstrated high binding affinity to α-glucosidase; both enzymes are capable of breaking down dietary carbohydrates. So, these phytoconstituents may be potential contributors to exert hypoglycemic effects through multi-target inhibition [59]. Similarly, the central pain-relieving effect seen in the tail-flick test may be corroborated by the μ-opioid receptor binding of s-Triazine 2-amino-4-(piperidinomethyl)-4-piperidino (A6) and 1-Naphthalenesulfonic acid, 5-(dimethylamino)- phenyl ester (A5); both compounds are also forecasted to penetrate the blood–brain barrier. Likewise, N-Methyl-N-(4-toluenesulfonyl)-benzamide (A1), s-Triazine, 2-amino-4-(piperidinomethyl)-4-piperidino (A6), and 2,3-diphenyl-2-cyclopropen-1-one (P11) demonstrated strong COX-1 and COX-2 binding. These may be promising candidates to support the peripheral analgesic effects observed in the writhing assay [60].

Assessing drug likeness, pharmacokinetics, and toxicity is crucial to determine if the identified compounds possess characteristics that warrant advancement as therapeutic agents [61]. In this study, 1-Naphthalenesulfonic acid, 5-(dimethylamino)- phenyl ester (A5) exerted immunotoxicity, which can be a serious consideration in drug development with the compound. But overall results showed acceptable pharmacokinetic profiles without significant toxicity issues, although these predictions should be interpreted with caution. For instance, blood–brain barrier permeability was predicted for several compounds; computational predictions do not necessarily confirm effective CNS bioavailability in vivo. Furthermore, compounds classified under Toxicity Class 4 may still require careful toxicological evaluation. Therefore, the ADMET and toxicity predictions presented in this study should be considered as preliminary computational insights that require further experimental validation.

Overall, the fraction-specific biological activities enable the probable attribution of pharmacological effects to chemically distinct fractions, and the docking, pharmacokinetic, and toxicity results support the fact that multiple phytoconstituents of *I. chinensis* are potential contributors to antihyperglycemic and pain-relieving effects. But the mechanistic pathways of the therapeutic effects were fully predicted by multi-target molecular docking strategies that evaluate interactions across pain-, inflammation-, and diabetes-related targets.

While this study yields unique and valuable scientific findings, it does have certain limitations. The identification of phytochemicals from the plant was conducted solely through GC-MS analysis. This method is limited to volatile compounds and omits other techniques, such as HPLC/MS, LC-MS, and NMR. On the other hand, the match factors of the identified compounds in GC-MS analysis ranged from 65 to 75, which indicate relatively low confidence in compound identification. At the same time, phenolics are identified directly by GC-MS analysis without derivatization, which does not align with the non-volatile nature of the high-molecular-weight and thermally unstable phenolics. Again, some compounds like etidocaine, sulfonamide, and triazine derivatives are not commonly reported as natural plant metabolites. This may also occur from the limitations of spectral library matching. The positive results of the in vivo analgesic activity assay were corroborated by molecular docking of the identified phytochemicals targeting a limited set of receptors, including the mu-opioid receptor (G protein-coupled receptor) and COX-1 and COX-2 (enzymes). But no Transient Receptor Potential (TRP)and Acid-Sensing Ion Channels (ASIC) receptors were targeted to validate the analgesic effect. Additionally, no molecular dynamics simulations were carried out for the compounds demonstrating the highest binding affinity in the in-silico study. It should be noted that molecular docking provides predictive insights into ligand–protein interactions and does not fully account for the dynamic stability of ligand–protein complexes in a biological environment. Therefore, further studies involving molecular dynamics simulations and experimental enzyme inhibition assays will be required to validate the stability and biological relevance of the identified ligand–protein interactions.

To entirely map the therapeutic potential of *I. chinensis*, future research should target the metabolite profiles of stems, roots, or flowers of the plant and assess other pharmacological activities.

## 5. Conclusions

The current study reveals that the methanolic crude extract of *I. chinensis* leaves contains many phytochemicals with chemical and pharmacological value. The fractions of the crude extract exerted hypoglycemic, central, and peripheral analgesic activity in an in vivo experiment. The in silico molecular docking analysis provided further support and identified a few phytochemicals likely responsible for these biological findings based on their binding affinity. For hypoglycemic activity, the n-hexane-soluble and chloroform-soluble fractions showed significant effects. In addition, 5-(dimethylamino)-1-Naphthalenesulfonic acid phenyl ester (A5), 2,3-diphenyl-2-cyclopropen-1-one (P11) demonstrated high binding affinity to AMPK and α-amylase, respectively. Again, 2,3-diphenyl-2-cyclopropen-1-one (P11) and compound 9,9-dimethyl-9H-Fluoren-3-ol (P8) showed a favorable interaction with α-glucosidase. The chloroform soluble fraction and the aqueous fraction exerted a significant central analgesic effect, which may be supported by the highest μ-opioid receptor affinity of s-Triazine, 2-amino-4-(piperidinomethyl)-4-piperidino (A6). Again, a strong binding tendency toward COX-1 and COX-2 suggests the probable peripheral analgesic activity of N-Methyl-N-(4-toluenesulfonyl)-benzamide (A1), s-Triazine, 2-amino-4-(piperidinomethyl)-4-piperidino (A6), and 2,3-diphenyl-2-cyclopropen-1-one (P11) in the computational study, which may support the significant outcome of the n-hexane and aqueous fractions in the writhing test. So, isolation and characterization of these bioactive compounds from the plant and mechanistic exploration of their hypoglycemic and analgesic activities will be the future objective.

## Figures and Tables

**Table 1:** GC-MS guided alkaloids obtained from the methanolic extract of *I. chinensis* leaves.

SL No.	Compound Name	Retention Time	Match Factor	Fragment Ion *m*/*z*	Formula	Molecular Weight	% Peak Area	Sub-Class
A1	N-Methyl-N-(4-toluensulfonyl)-benzamide	3.3612	65.39	118.0, 105.0, 77.0, 51.0	C_15_H_15_NO_3_S	289.077	0.17	Sulfonamide
A2	Etidocaine	4.1928	60.31	128.0, 105.0, 75.0	C_17_H_28_N_2_O	276.22	15.55	Isoquinoline alkaloid
A3	N,N,3-trimethylbenzenamine	5.3521	66.39	84.0	C_9_H_13_N	135.105	0.13	Aromatic amine
A4	5-Methyl-furan-2-carboxylic acid (1H-[1,2,4]triazol-3-yl)-amide	6.5377	68.05	110.1, 68.1, 42.4	C_8_H_8_N_4_O_2_	192.065	0.03	Furan-triazole hybrid
A5	5-(dimethylamino)-1-Naphthalenesulfonic acid phenyl ester	8.5007	80.08	170.2, 107.1	C_18_H_17_NO_3_S	327.093	0.11	Sulfonic ester
A6	s-Triazine, 2-amino-4-(piperidinomethyl)-4-piperidino-	11.2335	64.40	193.1, 179.1, 86.1	C_14_H_24_N_6_	276.206	1.89	Piperidine alkaloid
A7	4-methyl-N-(4-methylphenyl)-N-[4-[2-(4-methylphenyl)ethenyl]phenyl]-benzenamine	17.2228	65.62	307.0, 179.0	C_29_H_27_N	389.214	0.60	Aromatic amine
A8	1H-Pyrazole, 4,5-dihydro-3,4,5-trimethyl-	20.1259	62.81	97.0	C_6_H_12_N_2_	112.1	0.20	Pyrazole alkaloid

**Table 2:** GC-MS guided phenolics obtained from the methanolic extract of *I. chinensis* leaves.

SL No.	Compound Name	Retention Time	Match Factor	Fragment Ion *m*/*z*	Formula	Molecular Weight	% Peak Area	Sub-Class
P1	1-(2-hydroxyphenyl)-1-Butanone	3.5240	62.64	132.0, 121.0, 88.0	C_10_H_12_O_2_	164.084	0.13	Phenolic acid derivative
P2	Benzeneacetic acid, alpha-methoxy-, methyl ester	3.6061	67.44	121.1, 77.1	C_10_H_12_O_3_	180.079	2.03	Phenolic acid ester
P3	o-Acetophenetidide, N-(dimethylcarbamoylmethyl)-	6.9350	63.90	222.2, 202.2, 192.1, 149.9	C_14_H_20_N_2_O_3_	264.147	0.02	Phenolic amide
P4	2,4-Di-tert-butylphenol	7.7395	93.02	191.0, 163.0, 91.0	C_14_H_22_O	206.167	58.15	Simple Phenol (Antioxidant)
P5	3,5-Dihydroxybenzhydrazide	7.9937	63.57	137.0, 125.0, 81.0, 69.0	C_7_H_8_N_2_O_3_	168.053	0.43	Phenolic acid
P6	2(4H)-Benzofuranone, 5,6,7,7a-tetrahydro-4,4,7a-trimethyl-, I-	8.1749	60.22	137.1, 124.1, 111.1, 81.1, 67.1	C_11_H_16_O_2_	180.115	1.72	Lignan-like benzofuran
P7	p-Octylacetophenone	9.1452	64.81	217.2, 189.1, 133.1, 91.1	C_16_H_24_O	232.183	0.39	Phenolic ketone
P8	9,9-dimethyl-9H-Fluoren-3-ol	9.7511	60.56	195.0, 180.0, 165.0	C_15_H_14_O	210.104	0.32	Polycyclic aromatic alcohol
P9	Benzoic acid, 2-ethylhexyl ester	10.0084	87.45	105.0, 77.0, 55.0	C_15_H_22_O_2_	234.162	2.36	Phenolic acid ester
P10	1,4-benzenediol, 2-methyl-, 4-acetate	10.8903	66.21	124.0, 95.0, 79.0, 67.0	C_9_H_10_O_3_	166.063	0.61	Phenolic acid ester
P11	2,3-diphenyl-2-Cyclopropen-1-one	10.9368	70.21	178.0, 152.0	C_15_H_10_O	206.073	0.68	Stilbene-like aromatic ketone
P12	4-Methoxyphenylaldehyde trimethylene acetal	11.2309	60.72	193.0, 179.0	C_11_H_14_O_3_	194.094	1.89	Stilbene-like acetal
P13	m-Toluic acid, 2-ethylhexyl ester	11.2905	70.90	119.0, 91.0, 83.0, 70.0	C_16_H_24_O_2_	248.178	0.92	Aromatic ester
P14	Benzenepropanoic acid, 3,5-bis(1,1-dimethylethyl)-4-hydroxy-, methyl ester	12.3993	65.37	277.0, 219.0, 161.0, 147.0, 134.0	C_18_H_28_O_3_	292.204	0.01	Phenolic acid ester

**Table 3:** GC-MS guided esters obtained from the methanolic extract of *I. chinensis* leaves.

SL No.	Compound Name	Retention Time	Match Factor	Fragment Ion *m*/*z*	Formula	Molecular Weight	% Peak Area	Sub-Class
E1	d-Proline, N-ethoxycarbonyl-, isohexyl ester	5.4841	75.73	142.0, 85.0, 70.0, 41.0	C_14_H_25_NO_4_	271.178	0.21	Amino acid ester
E2	2-Ethylbutyric acid, tetrahydrofurfuryl ester	7.5483	68.81	84.0, 71.0, 55.0	C_11_H_20_O_3_	200.141	1.05	Fatty acid ester
E3	Succinic acid, tridec-2-yn-1-yl tetrahydrofurfuryl ester	11.5293	67.97	85.0, 71.0, 43.0	C_22_H_36_O_5_	380.256	0.18	Dicarboxylic ester
E4	Pentanedioic acid, 2-oxo-, dimethyl ester	12.5257	63.37	115.0, 87.0, 55.0	C_7_H_10_O_5_	174.053	0.16	Dicarboxylic ester

**Table 4:** GC-MS guided terpenoids obtained from the methanolic extract of *I. chinensis* leaves.

SL No.	Compound Name	Retention Time	Match Factor	Fragment Ion *m*/*z*	Formula	Molecular Weight	% Peak Area	Sub-Class
T1	Loliolide	10.7158	73.34	111.0, 95.0, 81.0	C_11_H_16_O_3_	196.11	0.22	Monoterpene lactone
T2	2-Ethyl-5-propylcyclopentanone	10.7865	60.24	118.0, 97.0, 83.0, 69.0	C_10_H_18_O	154.136	0.07	Cyclopentanone
T3	Phytol	11.3545	72.75	109.0, 95.0, 81.0, 71.0	C_20_H_40_O	296.308	2.96	Diterpene alcohol
T4	7,9-Di-tert-butyl-1-oxaspiro(4,5)deca-6,9-diene-2,8-dione	12.2055	79.97	205.0, 175.0, 91.0	C_17_H_24_O_3_	276.173	1.07	Flavonoid-like oxaspiro compound
T5	(Z,Z)-1,8,11-Heptadecatriene	13.7925	74.18	95.1, 81.1, 67.1	C_17_H_30_	234.235	0.34	Polyunsaturated hydrocarbon
T6	Tricyclo[3.2.1.0(2,4)]octan-8-one, 3,3-dimethyl-, (1.alpha.,2.alpha.,4.alpha.,5.alpha.)	13.8552	63.96	93.0, 79.0, 56.0	C_10_H_14_O	150.104	0.57	Monoterpene ketone
T7	5-Cyclohexyl-1-pentene	13.8895	64.39	95.1, 81.1	C_11_H_20_	152.157	0.38	Cycloalkene
T8	dl-Menthol	13.9583	79.98	95.0, 81.0, 71.0	C_10_H_20_O	156.151	0.13	Monoterpene alcohol
T9	3-Ethyl-3-methylheptane	14.7003	64.27	85.0, 71.0, 57.0, 43.0	C_10_H_22_	142.172	2.31	Alkane
T10	(3-octylundecyl)-Benzene	15.8950	60.25	91.0, 71.0, 43.0	C_25_H_44_	344.344	0.11	Alkylbenzene
T11	2-Methyl-3-(3-methyl-but-2-enyl)-2-(4-methyl-pent-3-enyl)-oxetane	19.6856	64.23	157.0, 81.0, 41.0	C_15_H_26_O	222.198	0.08	Sesquiterpene oxetane
T12	Epilupeol; 20(29)-Lupen-3alpha-ol, acetate (isomer 1)	23.9558	68.36	107.1, 93.1, 81.1	C_32_H_52_O_2_	468.397	0.32	Triterpene alcohol

**Table 5:** GC-MS guided sulfur-containing compounds obtained from the methanolic extract of *I. chinensis* leaves.

SL No.	Compound Name	Retention Time	Match Factor	Fragment Ion *m*/*z*	Formula	Molecular Weight	% Peak Area	Sub-Class
S1	Sulfurous acid, cyclohexylmethyl isohexyl ester	11.7824	73.44	97.0, 85.0, 69.0, 55.0, 43.0	C_13_H_26_O_3_S	262.16	0.33	Sulfur ester
S2	Sulfurous acid, isohexyl pentyl ester	14.5574	61.05	85.0, 71.0, 57.0	C_11_H_24_O_3_S	236.145	1.04	Sulfur ester
S3	Sulfurous acid, nonyl pentyl ester	15.5630	70.87	97.1, 85.1, 71.1, 57.1, 43.2	C_14_H_30_O_3_S	278.192	0.12	Sulfur ester

**Table 6:** Average glucose level (mmol/L) after loading the glucose sample.

Group	Average Glucose Level (mmol/L) After Loading the Glucose Sample				
	0 min	30 min	60 min	120 min	180 min
CTL	5.68 ± 0.20	10.63 ± 0.69	10.02 ± 0.53	9.58 ± 0.26	8.83 ± 0.37
STD	5.45 ± 0.27	10.98 ± 0.59	7.82 ± 0.32 ***	6.20 ± 0.39 ***	3.95 ± 0.14 ***
NHF-200	5.25 ± 0.11	8.45 ± 0.11	6.92 ± 0.08 ***	6.20 ± 0.09 ***	5.25 ± 0.11 ***
NHF-400	5.20 ± 0.17	8.40 ± 0.26	6.80 ± 0.15 ***	5.90 ± 0.09 ***	4.48 ± 0.17 ***
CF-200	6.25 ± 0.11	11.00 ± 0.41	9.22 ± 0.15 ***	8.27 ± 0.14 ***	7.22 ± 0.12 ***
CF-400	5.40 ± 0.32	9.40 ± 0.26	7.67 ± 0.21 ***	6.78 ± 0.17 ***	4.52 ± 0.08 ***
EAF-200	5.86 ± 0.17	10.63 ± 0.34	9.95 ± 0.24	9.25 ± 0.23 *	8.55 ± 0.11
EAF-400	5.70 ± 0.13	10.70 ± 0.23	9.80 ± 0.13	9.10 ± 0.13 **	8.42 ± 0.38
AQF-200	5.30 ± 0.28	10.55 ± 0.20	9.67 ± 0.10	9.42 ± 0.08	9.13 ± 0.20
AQF-400	6.52 ± 0.08	11.12 ± 0.21	10.17 ± 0.10	9.80 ± 0.46	9.15 ± 0.19

Data are mentioned as mean ± SD, *n* = 6. * *p* < 0.05; ** *p* < 0.01; *** *p* < 0.001 versus negative control.

**Table 7:** Central analgesic activities of *I. chinensis* leaves in mice by the tail immersion method.

Treatment	30 min		60 min		90 min	
	Mean ± SD (seconds)	% Time Elongation	Mean ± SD (seconds)	% Time Elongation	Mean ± SD (seconds)	% Time Elongation
Control	1.61 ± 0.34	-	1.81 ± 0.49	-	1.85 ± 0.22	-
Morphine	4.38 ± 0.05	171.49 ***	6.77 ± 0.08	274.38 ***	7.63 ± 0.05	312.81 ***
NHF-200	1.79 ± 0.05	10.84	2.25 ± 0.03	24.52	1.89 ± 0.04	2.44
NHF-400	1.85 ± 0.55	14.67	2.20 ± 0.11	21.84	1.95 ± 0.03	5.24
CF-200	1.72 ± 0.28	6.51	3.68 ± 0.73	103.32 ***	3.97 ± 0.51	114.70 ***
CF-400	1.95 ± 0.34	20.76	4.57 ± 0.50	152.72 ***	5.00 ± 0.21	170.51 ***
EAF-200	1.68 ± 0.16	3.92	1.89 ± 0.21	4.88	1.98 ± 0.83	7.30
EAF-400	1.72 ± 0.28	6.51	2.10 ± 0.27	16.31	2.03 ± 0.67	9.83
AQF-200	1.70 ± 0.22	5.58	3.30 ± 0.21	82.58 ***	4.03 ± 0.25	117.77 ***
AQF-400	1.85 ± 0.48	14.67	4.32 ± 0.43	139.08 ***	5.27 ± 0.19	184.94 ***

Here, *n* = 6; *** *p* < 0.001 against negative control.

**Table 8:** Peripheral analgesic activities of *I. chnensis* by the acetic acid-induced writhing method.

Groups	Mean Writhing (Mean ± SD)	% Inhibition of Writhing
Control	21.50 ± 3.01	-
Diclofenac sodium	6.50 ± 1.05 ***	69.77
NHF-200	15.67 ± 1.86 **	27.11
NHF-400	7.67 ± 0.81 ***	64.33
CF-200	20.67 ± 0.81	3.86
CF-400	20.67 ± 0.81	6.97
EAF-200	20.83 ± 0.76	3.11
EAF-400	19.67 ± 1.20	8.51
AQF-200	17.33 ± 1.86 *	19.40
AQF-400	9.17 ± 0.98 ***	57.35

Data are mentioned as mean ± SD, *n* = 6. * *p* < 0.05; ** *p* < 0.01; *** *p* < 0.001 versus negative control.

**Table 9:** Pharmacokinetics, drug-likeness and toxicity profile.

Criteria	A1	A5	A6	P8	P11	Diclofenac	Morphine	Metformin
PubChem CID	561987	598567	210894	606915	65057	3033	5288826	4091
MW	289.35	327.4	276.38	210.27	206.24	296.15	285.34	129.16
H-bond donors	0	1	1	1	0	2	2	3
H-bond acceptors	3	3	4	1	1	2	4	2
TPSA	62.83	54.99	71.17	20.23	17.07	49.33	52.93	91.49
LogP	2.66	2.88	2.98	2.32	2.39	1.98	2.55	0.34
BBB permeant	Yes	Yes	No	Yes	Yes	Yes	Yes	No
GI absorption	High	High	High	High	High	High	High	High
Lipinski Violations	0	0	0	0	0	0	0	0
Toxicity Class	6	4	5	4	4	3	4	4
Hepatotoxicity	Inactive	Inactive	Inactive	Inactive	Inactive	Active	Inactive	Inactive
Mutagenicity	Inactive	Inactive	Inactive	Inactive	Inactive	Inactive	Inactive	Inactive
Cytotoxicity	Inactive	Inactive	Inactive	Inactive	Inactive	Inactive	Inactive	Inactive
Immunotoxicity	Inactive	Active	Inactive	Inactive	Inactive	Inactive	Inactive	Inactive

## Data Availability

Data is contained in the article or the Supplementary Materials.

## Supplementary Materials

The following supporting information can be downloaded at: https://www.mdpi.com/article/10.3390/biology15080592/s1, File S1: Unknown Analysis Report—Best Hits. File S2: Table S1: Molecular docking binding affinities (kcal/mol) of all screened phytocompounds identified from Ixora chinensis against peripheral analgesic, central analgesic, and hypoglycemic target proteins. Table S2. Detailed molecular interactions between COX-1 (3N8Y) and the phytoconstituents A1 and P11, showing interacting amino acid residues, interaction types, distances (Å), and interaction categories. Table S3. Detailed molecular interactions between COX-2 (5F19) and the phytoconstituents A6 and P11, showing interacting amino acid residues, interaction types, distances (Å), and interaction categories. Table S4. Detailed molecular interactions between μ-opioid (5C1M) and the phytoconstituents A6 and A5, showing interacting amino acid residues, interaction types, distances (Å), and interaction categories. Table S5. Detailed molecular interactions between AMPK (4RER) and the phytoconstituents A5 and P8, showing interacting amino acid residues, interaction types, distances (Å), and interaction categories. Table S6. Detailed molecular interactions between α-amylase (1HNY) and the phytoconstituents P11 and A5, showing interacting amino acid residues, interaction types, distances (Å), and interaction categories. Table S7. Detailed molecular interactions between α-glucosidase (3TOP) and the phytoconstituents P11 and P8, showing interacting amino acid residues, interaction types, distances (Å), and interaction categories. Table S8. Evaluation of the Central analgesic activity of *Ixora chinensis* by the tail-flicking method. Table S9. Evaluation of Analgesic activity by intraperitoneal administration of 1% Acetic acid.

## References

[1] Al-Wajih A.M.M., El-Shaibany A., Alburyhi M.M., Abdelkhalek A.S., Elaasser M.M., Raslan A.E. (2025). Comparative study of phytochemical composition, oral toxicity, antioxidant, and anticancer activities of both Aloe vera and Aloe vacillans (Asphodelaceae family) flowers extract: In vitro, in vivo, and in silico studies. Trends Phytochem. Res..

[2] Karimi A., Majlesi M., Rafieian-Kopaei M. (2015). Herbal versus synthetic drugs; beliefs and facts. J. Nephropharmacol..

[3] d Sarker S., Nahar L. (2018). Evidence-based phytotherapy: What, why and how?. Trends Phytochem. Res..

[4] Hossain M.A., Pervin R. (2018). Current antidiabetic drugs: Review of their efficacy and safety. Nutr. Ther. Interv. Diabetes Metab. Syndr..

[5] Tran N., Pham B., Le L. (2020). Bioactive compounds in anti-diabetic plants: From herbal medicine to modern drug discovery. Biology.

[6] L Harvey A. (2010). Plant natural products in anti-diabetic drug discovery. Curr. Org. Chem..

[7] Falk S., Dickenson A.H. (2014). Pain and nociception: Mechanisms of cancer-induced bone pain. J. Clin. Oncol..

[8] Patterson D.R., Hoflund H., Espey K., Sharar S. (2004). Nursing Committee of the International Society for Burn Injuries. Pain management. Burns.

[9] Harirforoosh S., Asghar W., Jamali F. (2013). Adverse effects of nonsteroidal antiinflammatory drugs: An update of gastrointestinal, cardiovascular and renal complications. J. Pharm. Pharm. Sci..

[10] Bian A., Lu L. (2021). The complete chloroplast genome of *Ixora chinensis* and phylogenetic relationships. Mitochondrial DNA Part B.

[11] Sraboni N.H., Rafe M.R., Hussain M.M. (2025). Phytochemical Screening and Biological Activity Evaluation of the Methanolic Crude Extract of *Ixora chinensis* (Family: Rubiaceae). Bangladesh Pharm. J..

[12] Bhagyasri Y., Ali P., Raja M., Reddy N., Praveen D., Mounika K., Latha D., Parameshwari N. (2019). Determination of in-vitro anti microbial activity and anti-diabetic activity of *Ixora chinensis*. Am. J. Public Health Res..

[13] Hemalatha K., Sunitha D., Sirajunisa T., Suresh H.M. (2023). Isolation, characterization and evaluation of antioxidant and anticancer activities from isolated components of *Ixora chinensis* Lam. flowers. Ann. Phytomed..

[14] Nadeem R., Imran M., Saeed Z., Pervaiz M., Younas U. (2025). Exploring the therapeutic potential of *Ixora extract*: A comprehensive review of mediated studies. Adv. Tradit. Med..

[15] Sunitha D., Hemalatha K., Manthripragada B.R., Chary N. (2015). Extraction and isolation of active constituents from Ixora chinensis Lam leaves. Der Pharma Chem..

[16] Dontha S., Kamurthy H., Mantripragada B. (2015). Phytochemical and pharmacological profile of Ixora: A review. Int. J. Pharm. Sci. Res..

[17] Takeda Y., Nishimura H., Inouye H. (1975). Two new iridoid glucosides from Ixora chinensis. Phytochemistry.

[18] Aktar F., Kuddus M.R., Kabir S., Rashid M.A., Chakma K. (2013). Membrane stabilizing and cytotoxic activities of different Kupchan partitionates of *Oroxylum indicum* (L.) Vent. leaf and bark extracts. Dhaka Univ. J. Pharm. Sci..

[19] Zimmermann M. (1983). Ethical guidelines for investigations of experimental pain in conscious animals. Pain.

[20] Bedi O., Krishan P. (2020). Investigations on acute oral toxicity studies of purpurin by application of OECD guideline 423 in rodents. Naunyn-Schmiedeberg’s Arch. Pharmacol..

[21] El Hilaly J., Israili Z.H., Lyoussi B. (2004). Acute and chronic toxicological studies of Ajuga iva in experimental animals. J. Ethnopharmacol..

[22] Jegnie M., Abula T., Woldekidan S., Chalchisa D., Asmare Z., Afework M. (2023). Acute and sub-acute toxicity evaluation of the crude methanolic extract of Justicia schimperiana leaf in Wistar Albino Rats. J. Exp. Pharmacol..

[23] Rahman M.M., Soma M.A., Sultana N., Hossain M.J., Sufian M.A., Rahman M.O., Rashid M.A. (2023). Exploring therapeutic potential of *Woodfordia fruticosa* (L.) Kurz leaf and bark focusing on antioxidant, antithrombotic, antimicrobial, anti-inflammatory, analgesic, and antidiarrheal properties. Health Sci. Rep..

[24] Karim M.R., Hossain M.S., Islam M.S., Hossen M.R., Ali M.S. (2025). Exploration of Hypoglycemic, Analgesic, Antidiarrheal, and Antioxidant Properties of the Methanolic Extract of the Stems of *Gymnema inodorum* (Lour.) Decne. Dhaka Univ. J. Pharm. Sci..

[25] Alam S., Emon N.U., Rashid M.A., Arman M., Haque M.R. (2020). Investigation of biological activities of *Colocasia gigantea* Hook. f. leaves and PASS prediction, in silico molecular docking with ADME/T analysis of its isolated bioactive compounds. bioRxiv.

[26] Azad S.A.K., Sayeed M.A., Meah M.S., Mawa S.J., Alam S., Hasan M.N., Hanif M.A., Khan R., Arman M., Kim M.G. (2024). Unveiling the therapeutic potentialities and chemical characterization of methanolic *Merremia vitifolia* (Burm. f) Hallier f. stem extract: A Multi-faceted investigation via in vitro, in vivo, and in silico approaches. Heliyon.

[27] Labu Z.K., Karim S., Rahman M.T., Hossain M.I., Arifuzzaman S., Shakil M. (2025). Assessment of phytochemical screening, antibacterial, analgesic, and antipyretic potentials of *Litsea glutinosa* (L.) leaves extracts in a mice model. PLoS ONE.

[28] Shahriar S., Shermin S.A., Hasnat H., Hossain F., Han A., Geng P., Alam S., Mamun A.A. (2024). Chemico-pharmacological evaluation of the methanolic leaf extract of Catharanthus ovalis: GC–MS/MS, in vivo, in vitro, and in silico approaches. Front. Pharmacol..

[29] Yuan S., Chan H.C.S., Hu Z. (2017). Using PyMOL as a platform for computational drug design. WIREs Comput. Mol. Sci..

[30] Kim S., Chen J., Cheng T., Gindulyte A., He J., He S., Li Q., Shoemaker B.A., Thiessen P.A., Yu B. (2023). PubChem 2023 update. Nucleic Acids Res..

[31] Dallakyan S., Olson A.J. (2015). Small-Molecule Library Screening by Docking with PyRx. Chemical Biology: Methods and Protocols.

[32] Trott O., Olson A.J. (2010). AutoDock Vina: Improving the speed and accuracy of docking with a new scoring function, efficient optimization, and multithreading. J. Comput. Chem..

[33] Daina A., Michielin O., Zoete V. (2017). SwissADME: A free web tool to evaluate pharmacokinetics, drug-likeness and medicinal chemistry friendliness of small molecules. Sci. Rep..

[34] Lipinski C.A., Lombardo F., Dominy B.W., Feeney P.J. (2012). Experimental and computational approaches to estimate solubility and permeability in drug discovery and development settings. Adv. Drug Deliv. Rev..

[35] Banerjee P., Kemmler E., Dunkel M., Preissner R. (2024). ProTox 3.0: A webserver for the prediction of toxicity of chemicals. Nucleic Acids Res..

[36] Hussein R.A., El-Anssary A.A. (2019). Plants secondary metabolites: The key drivers of the pharmacological actions of medicinal plants. Herbal Med..

[37] Crozier A., Clifford M.N., Ashihara H. (2006). Plant secondary metabolites. Occurrence, Structure and Role in the Human Diet.

[38] Lund P.C., Cwik J.C., Pagdanganan R.T. (1973). Etidocaine—A new long-acting local anesthetic agent: A clinical evaluation. Anesth. Analg..

[39] Peyton L.R., Gallagher S., Hashemzadeh M. (2015). Triazole antifungals: A review. Drugs Today.

[40] Maliszewski D., Drozdowska D. (2022). Recent advances in the biological activity of s-triazine core compounds. Pharmaceuticals.

[41] Gutiérrez-del-Río I., López-Ibáñez S., Magadán-Corpas P., Fernández-Calleja L., Pérez-Valero Á., Tuñón-Granda M., Miguélez E.M., Villar C.J., Lombó F. (2021). Terpenoids and polyphenols as natural antioxidant agents in food preservation. Antioxidants.

[42] Chen L.J., Zhang Y., Chen Y.G. (2016). Chemical constituents of plants from the Genus Ixora. Chem. Biodivers..

[43] Yang X., Kang M.C., Lee K.W., Kang S.M., Lee W.W., Jeon Y.J. (2011). Antioxidant activity and cell protective effect of loliolide isolated from Sargassum ringgoldianum subsp. coreanum. Algae.

[44] Santos C.C.D.M.P., Salvadori M.S., Mota V.G., Costa L.M., de Almeida A.A.C., de Oliveira G.A.L., Costa J.P., de Sousa D.P., de Freitas R.M., de Almeida R.N. (2013). Antinociceptive and antioxidant activities of phytol in vivo and in vitro models. Neurosci. J..

[45] Ihegboro G.O., Ononamadu C.J., Owolarafe T.A., Onifade O., Udeh J.J., Saliu A.O., Abolaji D.D., Ibrahim Y.M. (2024). In vitro Investigation and GC-MS Analysis of the Chemical Constituents in the Fraction of Hexane Leaf Extract of Tapinanthus bangwensis (Engl. and K. Krause). Trop. J. Phytochem. Pharm. Sci..

[46] Xin F., Du C., Lan G., Wu Z. (2013). Synthesis, Characterization, and Agricultural Biological Activities of 5-Fluoro-2-hydroxy Butyrophenone. J. Chem..

[47] Zhao F., Wang P., Lucardi R.D., Su Z., Li S. (2020). Natural sources and bioactivities of 2, 4-di-tert-butylphenol and its analogs. Toxins.

[48] Shelke D.B., Tayade S., Gawande P., Sonawane H.B. (2022). GC-MS analysis and antioxidant potential of wild underutilized medicinally important legume, velvet bean (*Mucuna pruriens* L. DC.). Not. Sci. Biol..

[49] Mozibullah M., Ahammad H., Tarin T., Islam M.J., Khatun M., Islam M.J., Sikder M.A. (2023). Evaluation of anti-oxidant and antibacterial activities of *Ixora chinensis* and *Cascabela thevetia* leaf extracts: An in vitro study. J. Pharmacogn. Phytochem..

[50] Aquino R., De Feo V., De Simone F., Pizza C., Cirino G. (1991). Plant metabolites. New compounds and anti-inflammatory activity of Uncaria tomentosa. J. Nat. Prod..

[51] Walag A.M.P., Ahmed O., Jeevanandam J., Akram M., Ephraim-Emmanuel B.C., Egbuna C., Semwal P., Iqbal M., Hassan S., Uba J.O. (2020). Health benefits of organosulfur compounds. Functional Foods and Nutraceuticals: Bioactive Components, Formulations and Innovations.

[52] Kharasch N., Potempa S.J., Wehrmeister H.L. (1946). The sulfenic acids and their derivatives. Chem. Rev..

[53] Rahman S.S., Klamrak A., Nopkuesuk N., Nabnueangsap J., Janpan P., Choowongkomon K., Daduang J., Daduang S. (2024). Impacts of Plu kaow (*Houttuynia cordata* Thunb.) ethanolic extract on diabetes and dyslipidemia in STZ induced diabetic rats: Phytochemical profiling, cheminformatics analyses, and molecular docking studies. Antioxidants.

[54] Henriksen G., Willoch F. (2008). Imaging of opioid receptors in the central nervous system. Brain.

[55] Simon L.S. (1999). Role and regulation of cyclooxygenase-2 during inflammation. Am. J. Med..

[56] Roy D., Brar S., Bhatia R., Rangra N.K. (2024). An insight into the ethnopharmacological importance of Indian subcontinent medicinal plant species of Rubiaceae family. Adv. Tradit. Med..

[57] Chaudhary M., Tyagi K. (2024). A review on molecular docking and it’s application. Int. J. Adv. Res..

[58] Zhang B.B., Zhou G., Li C. (2009). AMPK: An emerging drug target for diabetes and the metabolic syndrome. Cell Metab..

[59] Wang X., Li J., Shang J., Bai J., Wu K., Liu J., Yang Z., Ou H., Shao L. (2022). Metabolites extracted from microorganisms as potential inhibitors of glycosidases (α-glucosidase and α-amylase): A review. Front. Microbiol..

[60] Martinez R.V., Reval M.I., Campos M.D., Terrón J.A., Ramírez A.M., López-Muñoz F.J. (2002). Involvement of peripheral cyclooxygenase-1 and cyclooxygenase-2 in inflammatory pain. J. Pharm. Pharmacol..

[61] Honorio K.M., Moda T.L., Andricopulo A.D. (2013). Pharmacokinetic Properties and In Silico ADME Modeling in Drug Discovery. Med. Chem..

