# Supplementary material for: GC-MS Guided Phytochemical Fingerprinting and Multi-Target Therapeutic Evaluation of Ixora chinensis Lam. Leaves: Insights into Its Hypoglycemic and Analgesic Activities
Source: Biology (Basel). 2026 Apr 8;15(8):592. doi: 10.3390/biology15080592 (PMC13114147; doi:10.3390/biology15080592)
Supplement: Supplementary file 1 [file biology-15-00592-s001.zip › biology-4239433-supplementary/biology-4239433-Supplementary file 1.pdf]

# Unknown Analysis Report - Best Hits

| | | | |
|---|---|---|---|
| **Batch Path** | D:\MassHunter\GCMS\1\data\Adhoc_21092025 | | |
| **Analysis File Name** | BBBB.uaf | | |
| **Analyst Name** | Admin | | |
| **Analysis Time** | 9/22/2025 5:01:51 PM | | |
| **Data File Name** | IC_E.D | **Data Path Name** | D:\MassHunter\GCMS\1\data\Adhoc_21092025 |
| **Sample Name** | IC_E | **Sample Type** | Sample |
| **Acq Method File** | Adhoc_method_27072025 | **Acq Method Path** | D:\MassHunter\GCMS\1\methods\ |
| **Acq Time** | 9/9/2025 3:08:07 PM | **Operator** | |
| **Instrument Name** | GCMSMS-01 | **Dilution** | 1 |

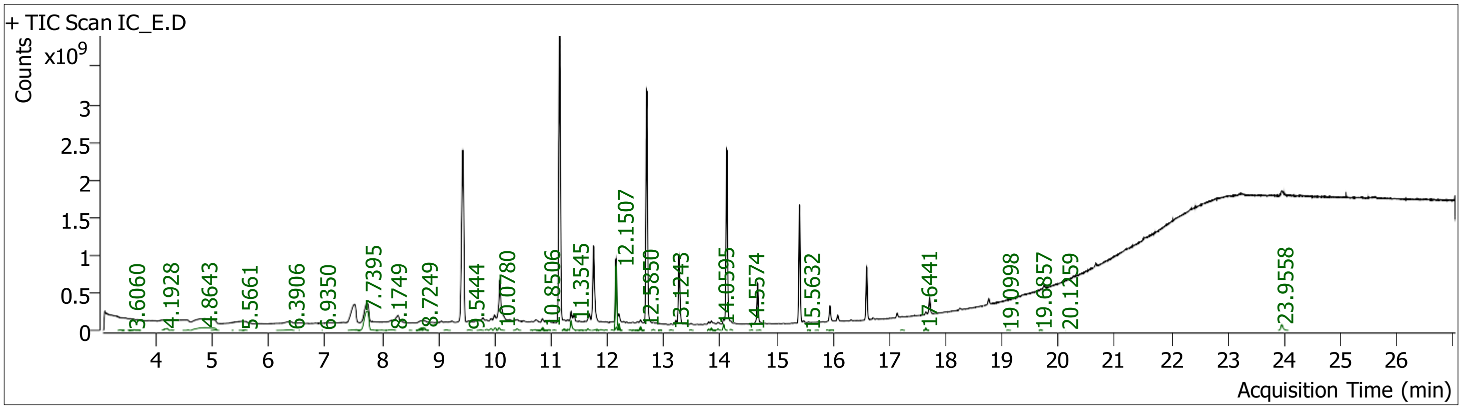


| Code | RT | Compound Name | CAS# | Forula | Area | % peak area | Match Score | Sample | Sample |
|---|---|---|---|---|---|---|---|---|---|
| A1 | 3.3612 | N-Methyl-N-(4-toluensulfonyl)-benzamide | 10533-83-2 | C15H15NO3S | 9707903 | 0.17% | 65.4 | 0.14 | 0.63 |
| P1 | 3.5240 | 1-Butanone, 1-(2-hydroxyphenyl)- | 2887-61-8 | C10H12O2 | 2887307 | 0.13% | 62.6 | 0.04 | 0.19 |
| P2 | 3.6061 | Benzeneacetic acid, .alpha.-methoxy-, methyl ester, (.+/-.)- | 56143-21-6 | C10H12O3 | 31936234 | 2.03% | 67.4 | 0.46 | 2.09 |
| A2 | 4.1928 | Etidocaine | 36637-18-0 | C17H28N2O | 110090288 | 15.55% | 60.3 | 1.59 | 7.20 |
| A3 | 5.3521 | Benzenamine, N,N,3-trimethyl- | 121-72-2 | C9H13N | 842752 | 0.13% | 66.4 | 0.01 | 0.06 |
| E1 | 5.4841 | d-Proline, N-ethoxycarbonyl-, isohexyl ester | 1000320-83-6 | C14H25NO4 | 1127826 | 0.21% | 75.7 | 0.02 | 0.07 |
| A4 | 6.5377 | 5-Methyl-furan-2-carboxylic acid (1H-[1,2,4]triazol-3- yl)-amide | 1010275-85-3 | C8H8N4O2 | 459131 | 0.03% | 68.0 | 0.01 | 0.03 |
| P3 | 6.9350 | o-Acetophenetidide, N-(dimethylcarbamoylmethyl)- | 97214-80-7 | C14H20N2O3 | 429897 | 0.02% | 63.9 | 0.01 | 0.03 |
| E2 | 7.5483 | 2-Ethylbutyric acid, tetrahydrofurfuryl ester | 1000370-65-1 | C11H20O3 | 14419760 | 1.05% | 68.8 | 0.21 | 0.94 |
| P4 | 7.7395 | 2,4-Di-tert-butylphenol | 96-76-4 | C14H22O | 1421709555 | 58.15% | 93.0 | 20.58 | 92.93 |
| P5 | 7.9937 | 3,5-Dihydroxybenzhydrazide | 7732-32-3 | C7H8N2O3 | 8723291 | 0.43% | 63.6 | 0.13 | 0.57 |
| P6 | 8.1749 | 2(4H)-Benzofuranone, 5,6,7,7a-tetrahydro-4,4,7a- trimethyl-, (R)- | 17092-92-1 | C11H16O2 | 44248161 | 1.72% | 60.2 | 0.64 | 2.89 |
| A5 | 8.5007 | 1-Naphthalenesulfonic acid, 5-(dimethylamino)-, phenyl ester | 55837-12-2 | C18H17NO3S | 1340026 | 0.11% | 80.1 | 0.02 | 0.09 |
| P7 | 9.1452 | p-Octylacetophenone | 10541-56-7 | C16H24O | 14607422 | 0.39% | 64.8 | 0.21 | 0.95 |
| P8 | 9.7511 | 9H-Fluoren-3-ol, 9,9-dimethyl- | 1000141-55-1 | C15H14O | 10495502 | 0.32% | 60.6 | 0.15 | 0.69 |
| P9 | 10.0084 | Benzoic acid, 2-ethylhexyl ester | 5444-75-7 | C15H22O2 | 84319768 | 2.36% | 87.5 | 1.22 | 5.51 |
| T1 | 10.7158 | Loliolide | 5989-02-6 | C11H16O3 | 28394896 | 0.22% | 73.3 | 0.41 | 1.86 |
| T2 | 10.7865 | 2-Ethyl-5-propylcyclopentanone | 38468-47-2 | C10H18O | 3770899 | 0.07% | 60.2 | 0.05 | 0.25 |
| P10 | 10.8903 | 1,4-benzenediol, 2-methyl-, 4-acetate | 1000404-50-8 | C9H10O3 | 14845863 | 0.61% | 66.2 | 0.21 | 0.97 |
| P11 | 10.9367 | 2-Cyclopropen-1-one, 2,3-diphenyl- | 886-38-4 | C15H10O | 14370581 | 0.68% | 70.2 | 0.21 | 0.94 |
| P12 | 11.2309 | 4-Methoxyphenylaldehyde trimethylene acetal | 1010429-63-9 | C11H14O3 | 28359542 | 1.89% | 60.7 | 0.41 | 1.85 |
| A6 | 11.2335 | s-Triazine, 2-amino-4-(piperidinomethyl)-4-piperidino- | 21868-43-9 | C14H24N6 | 36602731 | 1.89% | 64.4 | 0.53 | 2.39 |
| P13 | 11.2905 | m-Toluic acid, 2-ethylhexyl ester | 16397-66-3 | C16H24O2 | 35764182 | 0.92% | 70.9 | 0.52 | 2.34 |
| T3 | 11.3545 | Phytol | 150-86-7 | C20H40O | 278860886 | 2.96% | 82.7 | 4.04 | 18.23 |
| E3 | 11.5293 | Succinic acid, tridec-2-yn-1-yl tetrahydrofurfuryl ester | 1000390-72-9 | C22H36O5 | 13688696 | 0.18% | 68.0 | 0.20 | 0.89 |
| S1 | 11.7824 | Sulfurous acid, cyclohexylmethyl isohexyl ester | 1000309-21-5 | C13H26O3S | 20190385 | 0.33% | 73.4 | 0.29 | 1.32 |

| | | | | | | | | | |
|---|---|---|---|---|---|---|---|---|---|
| T4 | 12.2051 | 7,9-Di-tert-butyl-1-oxaspiro(4,5)deca-6,9-diene-2,8- dione | 82304-66-3 | C17H24O3 | 140017492 | 1.07% | 79.7 | 2.03 | 9.15 |
| P14 | 12.3993 | Benzenepropanoic acid, 3,5-bis(1,1-dimethylethyl)-4- hydroxy-, methyl ester | 6386-38-5 | C18H28O3 | 5597808 | 0.01% | 65.4 | 0.08 | 0.37 |
| E4 | 12.5257 | Pentanedioic acid, 2-oxo-, dimethyl ester | 13192-04-6 | C7H10O5 | 15447030 | 0.16% | 63.4 | 0.22 | 1.01 |
| T5 | 13.7925 | 1,8,11-Heptadecatriene, (Z,Z)- | 56134-03-3 | C17H30 | 23339813 | 0.34% | 74.2 | 0.34 | 1.53 |
| T6 | 13.8552 | Tricyclo[3.2.1.0(2,4)]octan-8-one, 3,3-dimethyl-, (1.alpha.,2.alpha.,4.alpha.,5.alpha.)- | 66930-01-6 | C10H14O | 13491723 | 0.57% | 64.0 | 0.20 | 0.88 |
| T7 | 13.8895 | 5-Cyclohexyl-1-pentene | 5729-54-4 | C11H20 | 7466289 | 0.38% | 64.4 | 0.11 | 0.49 |
| T8 | 13.9583 | dl-Menthol | 89-78-1 | C10H20O | 44635658 | 0.13% | 80.0 | 0.65 | 2.92 |
| T9 | 14.1866 | 3-Ethyl-3-methylheptane | 17302-01-1 | C10H22 | 29404028 | 2.31% | 79.9 | 0.43 | 1.92 |
| S2 | 14.5574 | Sulfurous acid, isohexyl pentyl ester | 1000309-14-0 | C11H24O3S | 8084505 | 1.04% | 61.0 | 0.12 | 0.53 |
| S3 | 15.5630 | Sulfurous acid, nonyl pentyl ester | 1000309-14-2 | C14H30O3S | 7762090 | 0.12% | 70.9 | 0.11 | 0.51 |
| T10 | 15.8950 | Benzene, (3-octylundecyl)- | 5637-96-7 | C25H44 | 7628143 | 0.11% | 60.2 | 0.11 | 0.50 |
| A7 | 17.2228 | benzenamine, 4-methyl-N-(4-methylphenyl)-N-[4-[2- (4-methylphenyl)ethenyl]phenyl]- | 1000402-70-2 | C29H27N | 13203886 | 0.60% | 65.6 | 0.19 | 0.86 |
| T11 | 19.6856 | 2-Methyl-3-(3-methyl-but-2-enyl)-2-(4-methyl-pent-3- enyl)-oxetane | 1000144-10-2 | C15H26O | 11278308 | 0.08% | 64.2 | 0.16 | 0.74 |
| A8 | 20.1259 | 1H-Pyrazole, 4,5-dihydro-3,4,5-trimethyl- | 22591-95-3 | C6H12N2 | 647995 | 0.20% | 62.8 | 0.01 | 0.04 |
| T12 | 23.9558 | Epilupeol; 20(29)-Lupen-3alpha-ol, acetate (isomer 1) | 1000513-01-7 | C32H52O2 | 220811549 | 0.32% | 68.4 | 3.20 | 14.43 |

| RT | Compound Name | CAS# | Formula | Area | MI | Match Score | Sample | Sample |
|---|---|---|---|---|---|---|---|---|
| 3.3612 | N-Methyl-N-(4-toluensulfonyl)-benzamide | 10533-83-2 | C15H15NO3S | 9707903 | | 65.4 | 0.14 | 0.63 |

Component RT: 3.3612

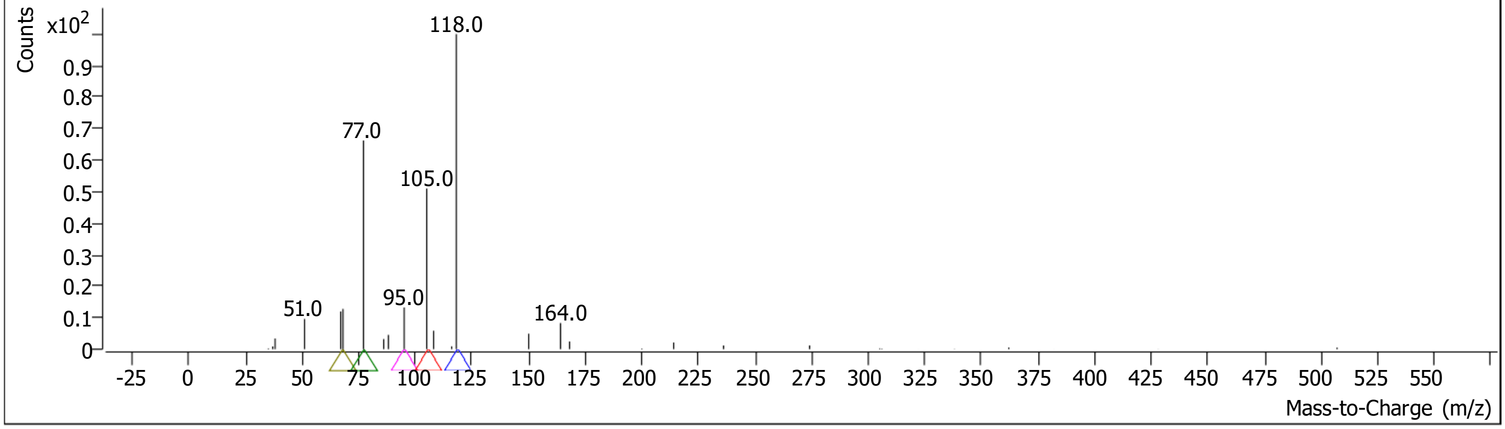


N-Methyl-N-(4-toluensulfonyl)-benzamide (NIST20.L)

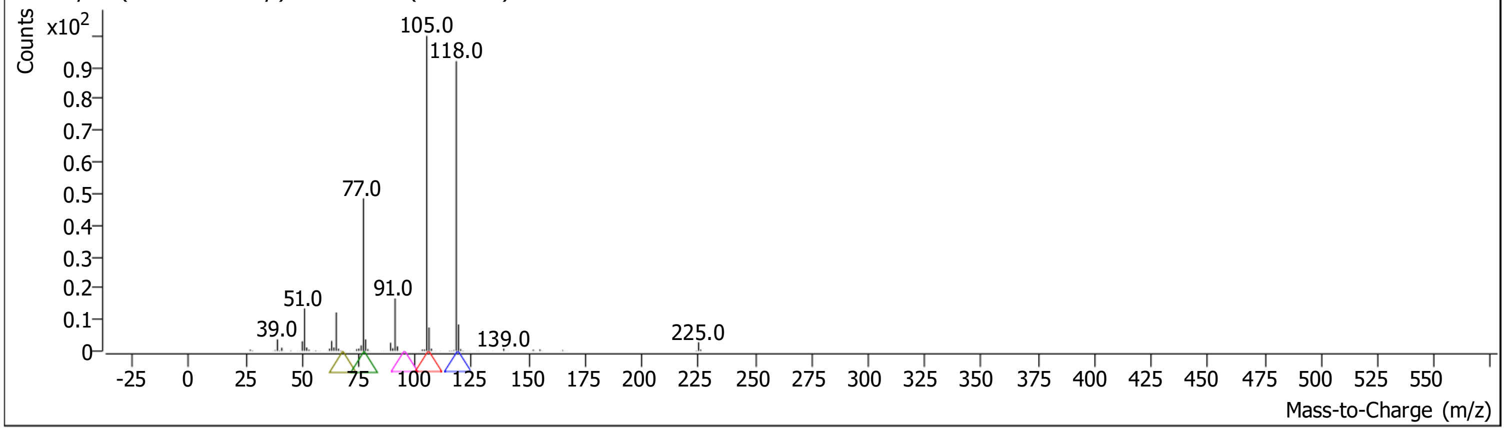


+ Scan (3.3333-3.4380 min, 55 scans) IC_E.D

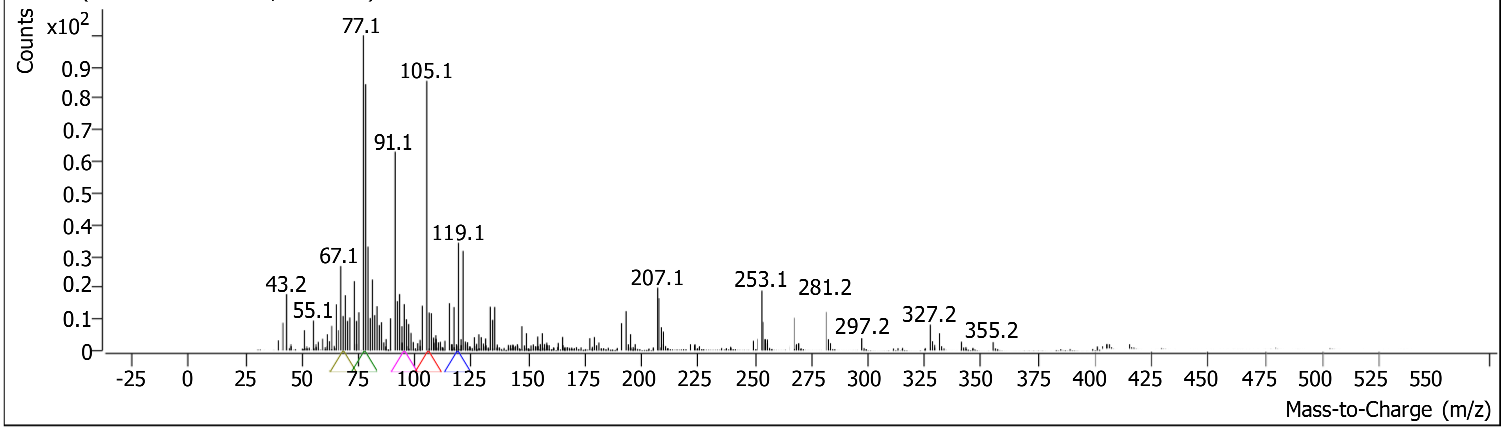


Component RT: 3.3612

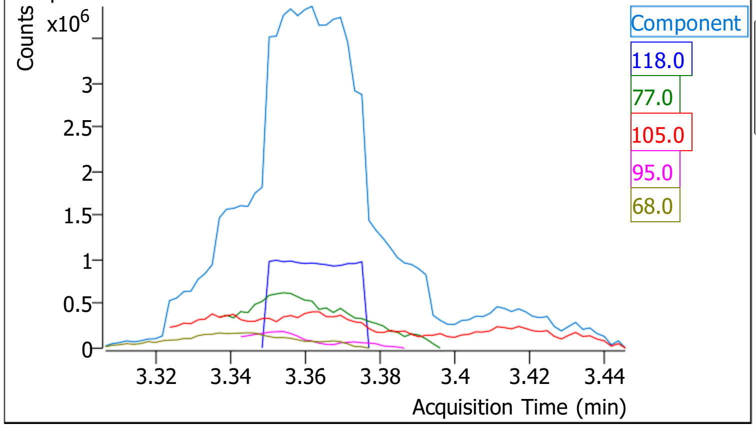


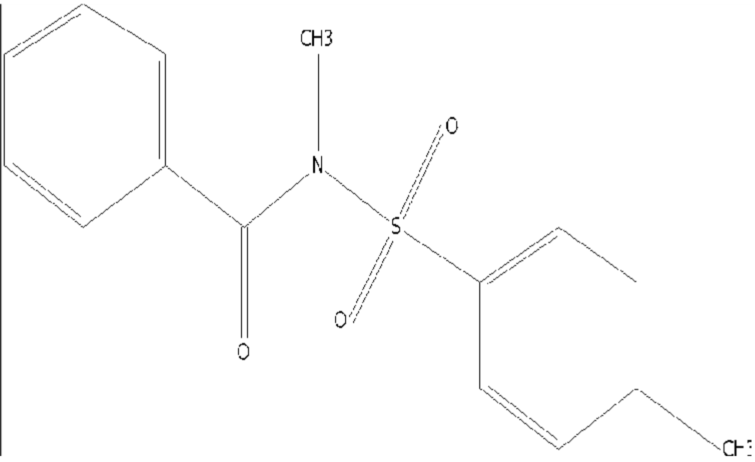


 Generated at 5:24 PM on 9/22/2025

# Unknown Analysis Report - Best Hits

| RT | Compound Name | CAS# | Formula | Area | MI | Match Score | Sample | Sample |
|---|---|---|---|---|---|---|---|---|
| 3.5240 | 1-Butanone, 1-(2-hydroxyphenyl)- | 2887-61-8 | C10H12O2 | 2887307 | | 62.6 | 0.04 | 0.19 |

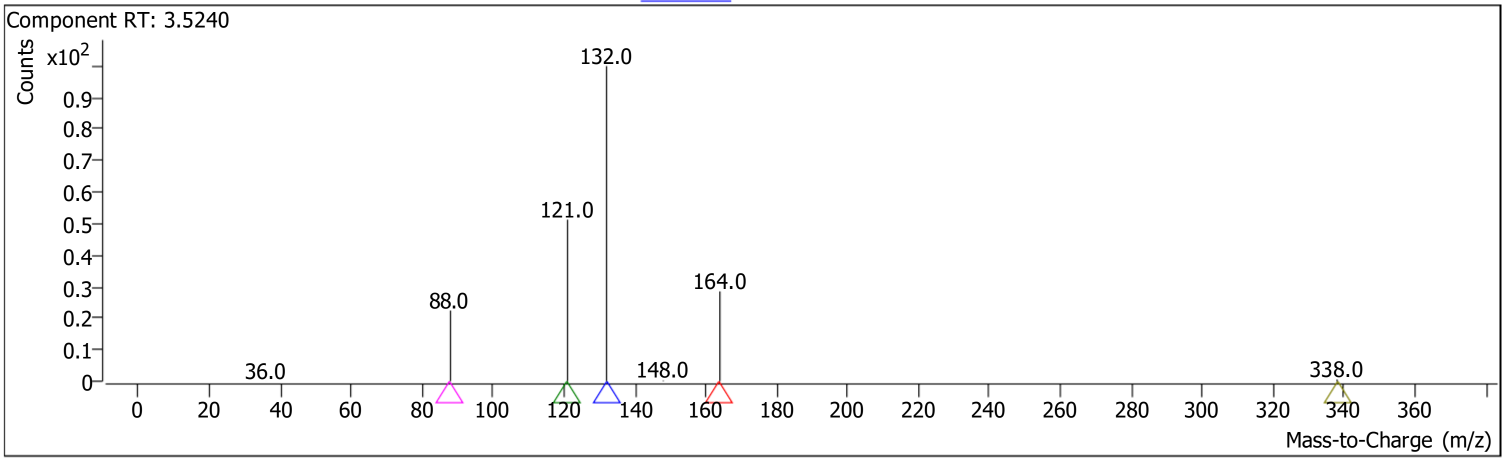


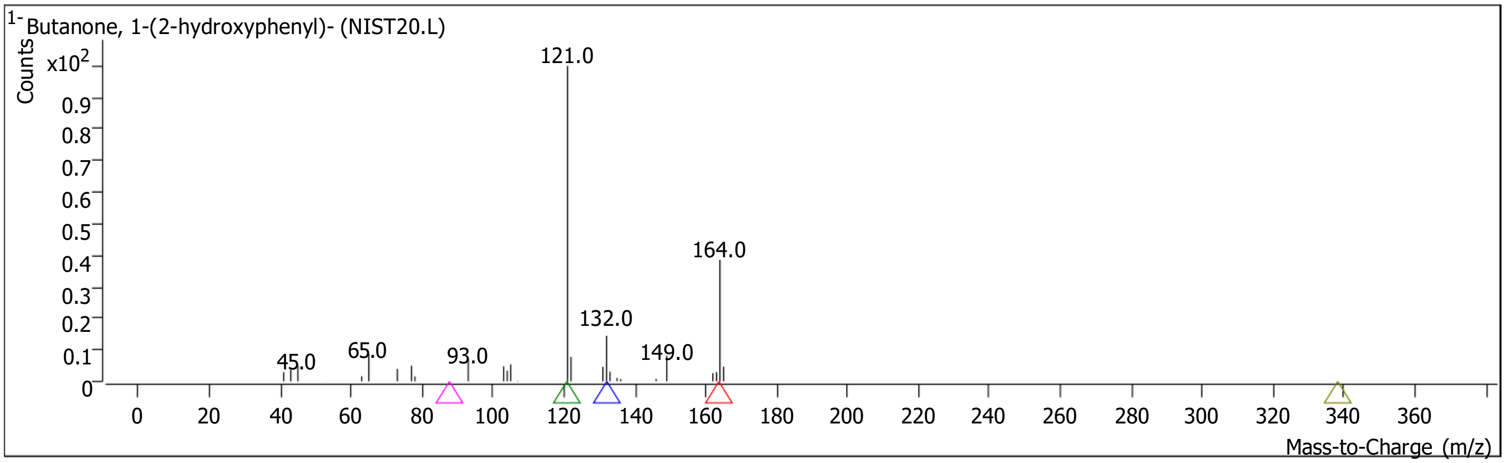


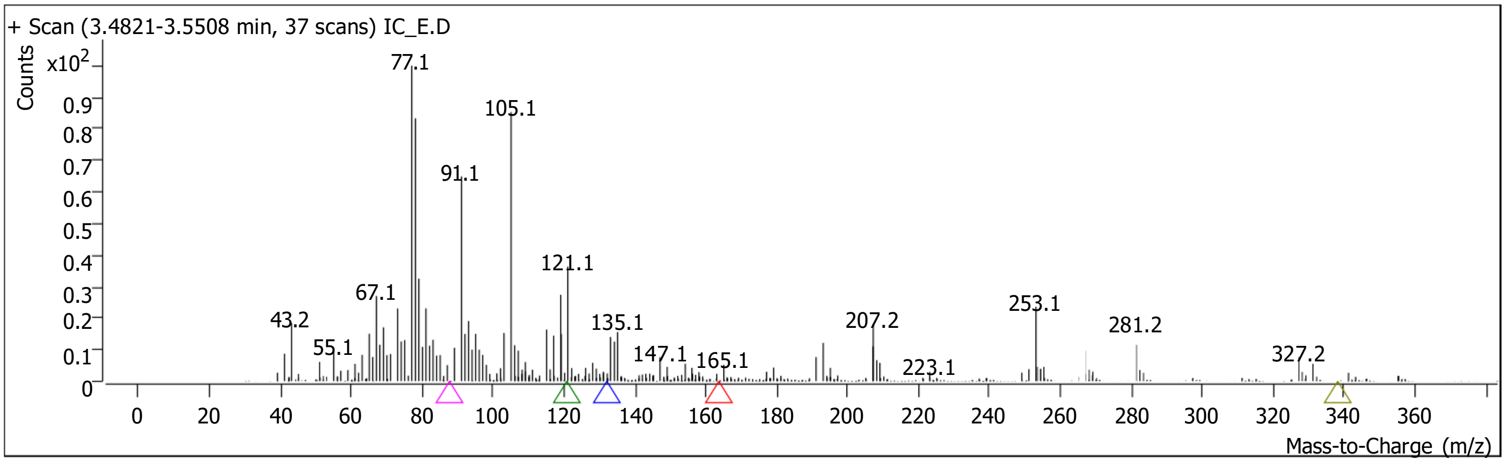


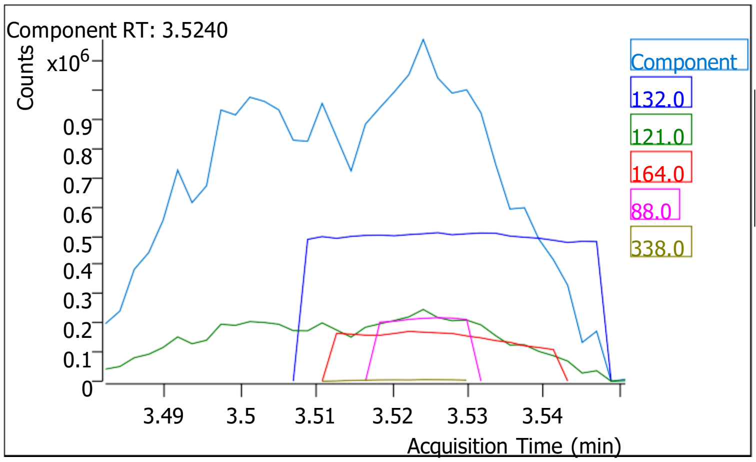


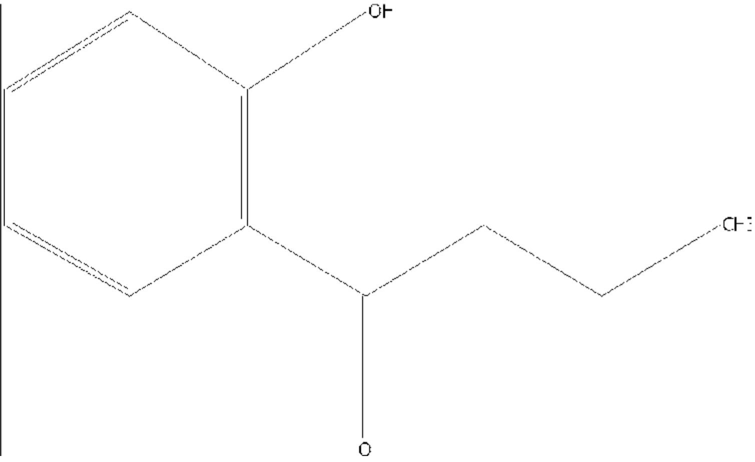

# Unknown Analysis Report - Best Hits

| RT | Compound Name | CAS# | Formula | Area | MI | Match Score | Sample | Sample |
|---|---|---|---|---|---|---|---|---|
| 3.6061 | Benzeneacetic acid, .alpha.-methoxy-, methyl ester, (.+/-.)- | 56143-21-6 | C10H12O3 | 31936234 | | 67.4 | 0.46 | 2.09 |

Component RT: 3.6061

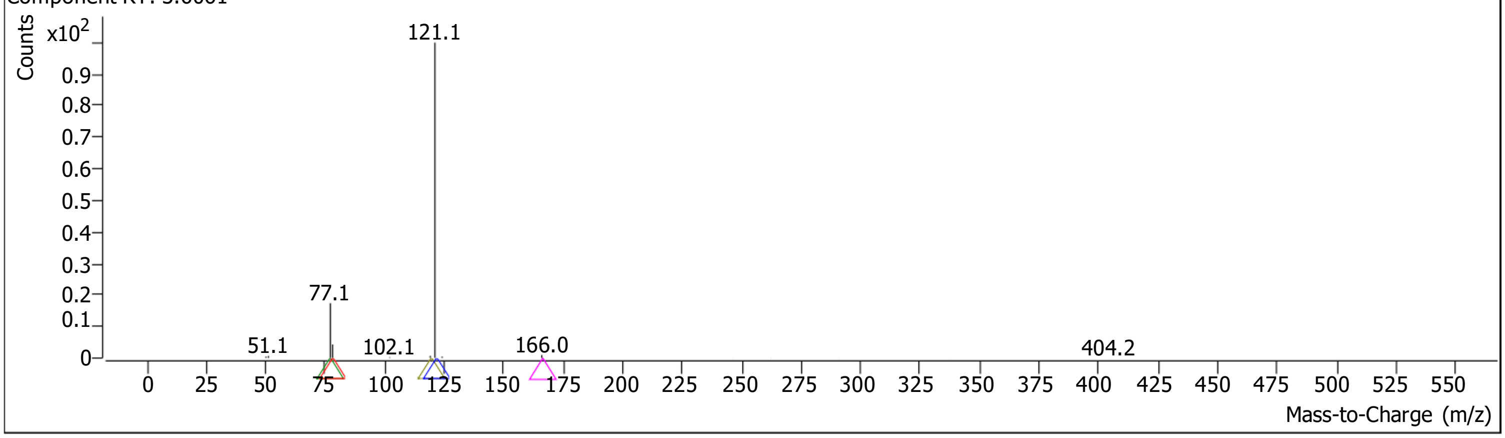


Benzeneacetic acid, .alpha.-methoxy-, methyl ester, (.+/-.)- (NIST20.L)

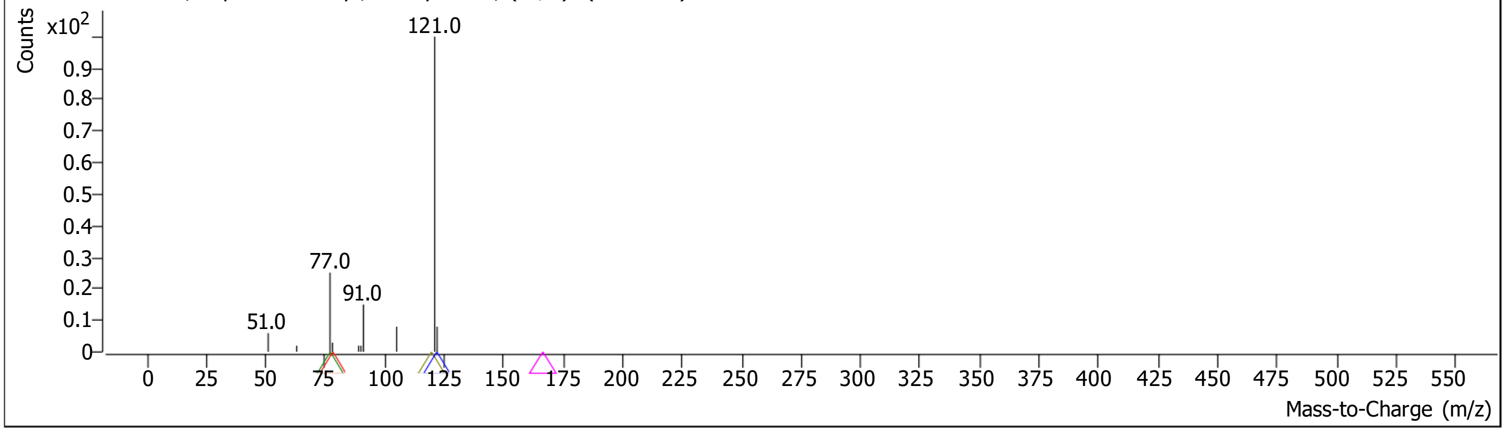


+ Scan (3.5965-3.6303 min, 18 scans) IC_E.D

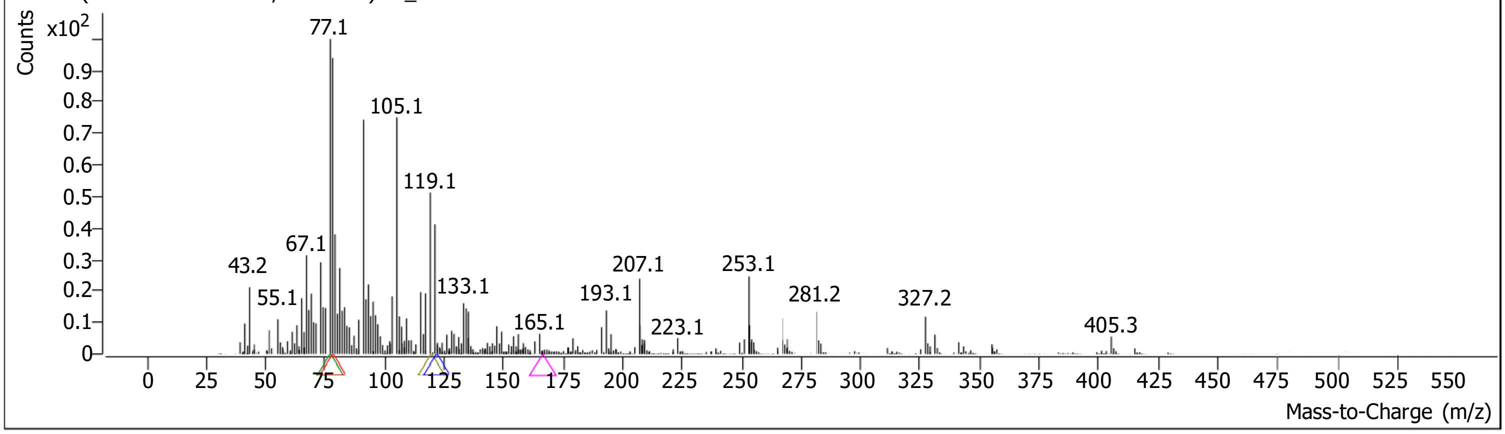


Component RT: 3.6061

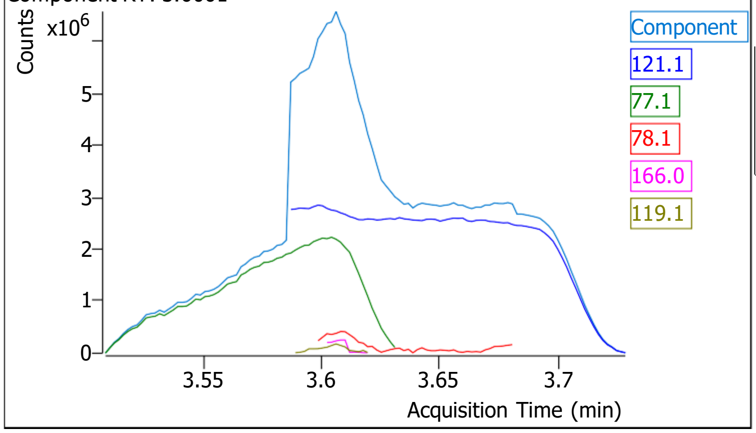


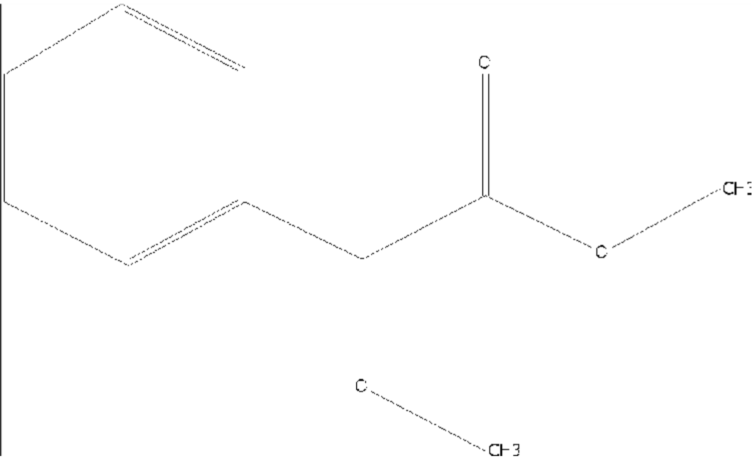


 Generated at 5:24 PM on 9/22/2025

| RT | Compound Name | CAS# | Formula | Area | MI | Match Score | Sample | Sample |
|---|---|---|---|---|---|---|---|---|
| 4.1928 | Etidocaine | 36637-18-0 | C17H28N2O | 110090288 | | 60.3 | 1.59 | 7.20 |

Component RT: 4.1928

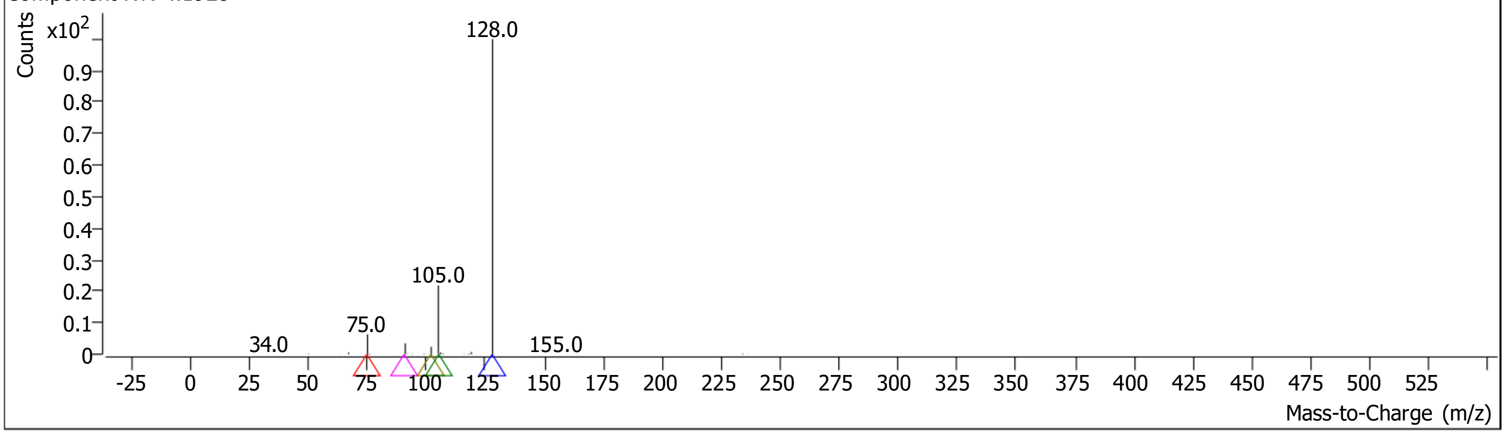

Etidocaine (NIST20.L)

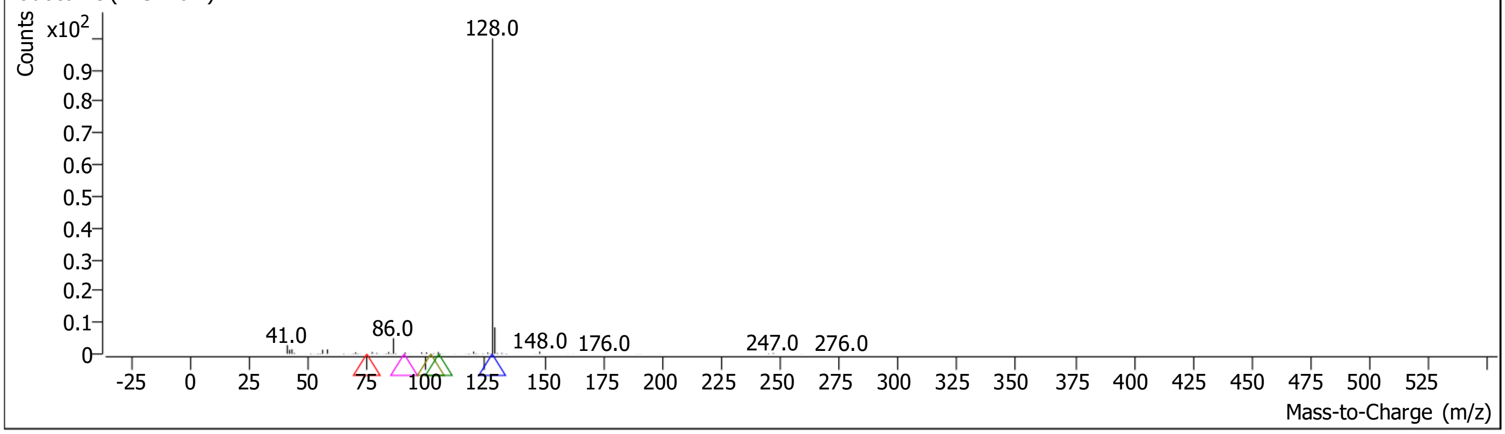

+ Scan (4.2648-4.3172 min, 27 scans) IC_E.D

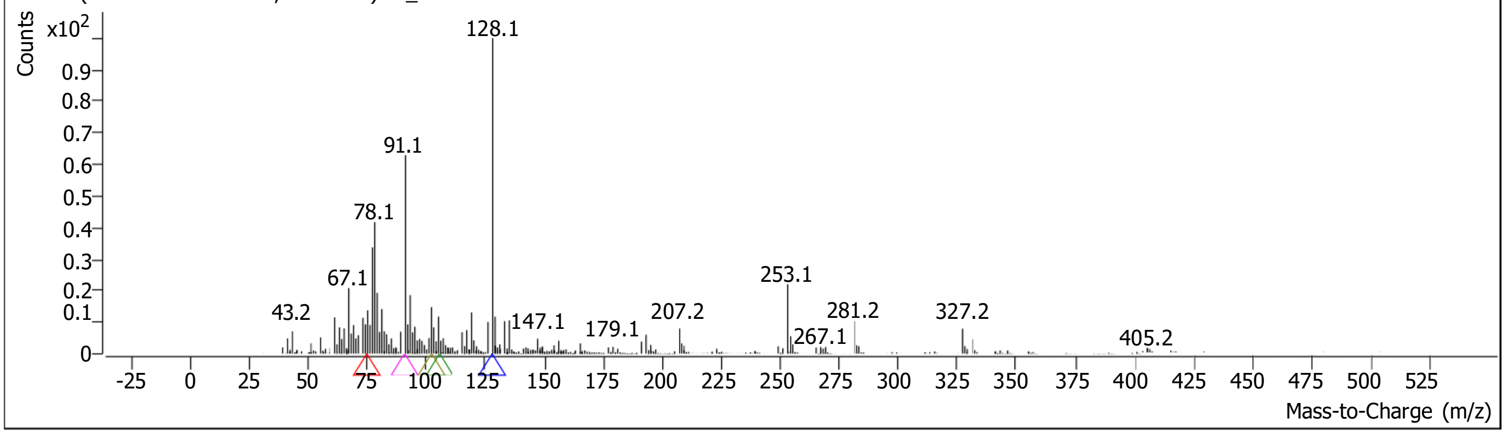

Component RT: 4.1928

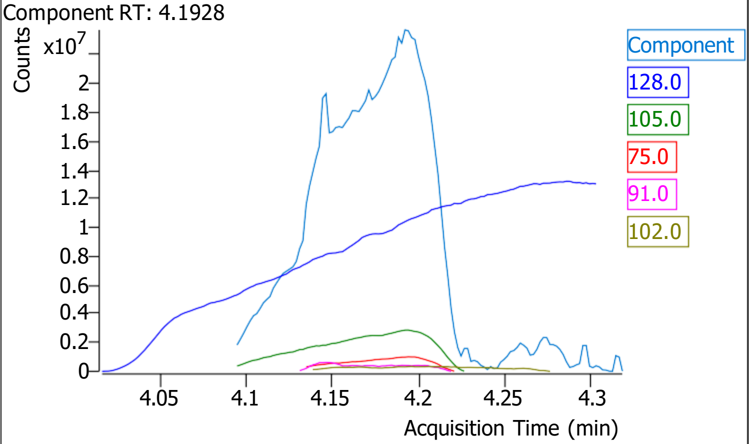

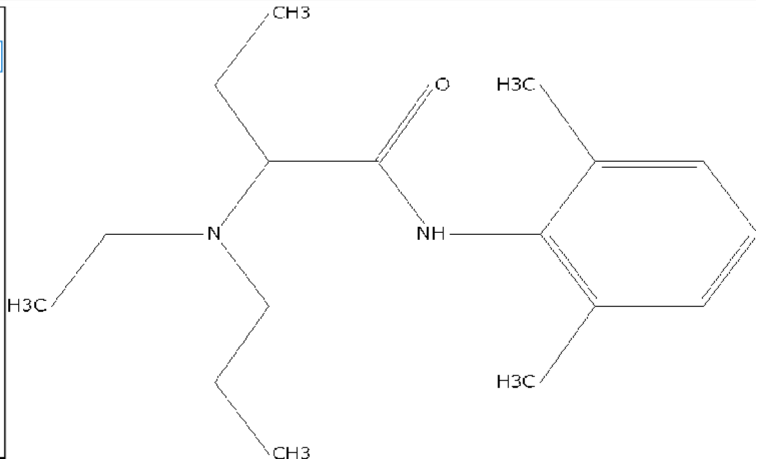

# Unknown Analysis Report - Best Hits

| RT | Compound Name | CAS# | Formula | Area | MI | Match Score | Sample | Sample |
|---|---|---|---|---|---|---|---|---|
| 5.3521 | Benzenamine, N,N,3-trimethyl- | 121-72-2 | C9H13N | 842752 | | 66.4 | 0.01 | 0.06 |

Component RT: 5.3521

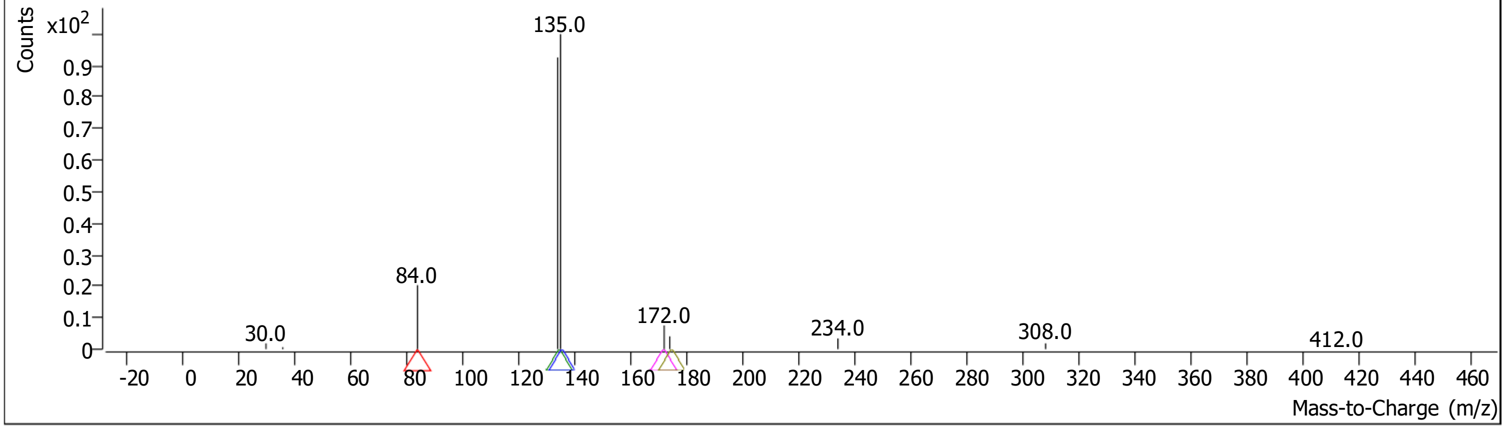


Benzenamine, N,N,3-trimethyl- (NIST20.L)

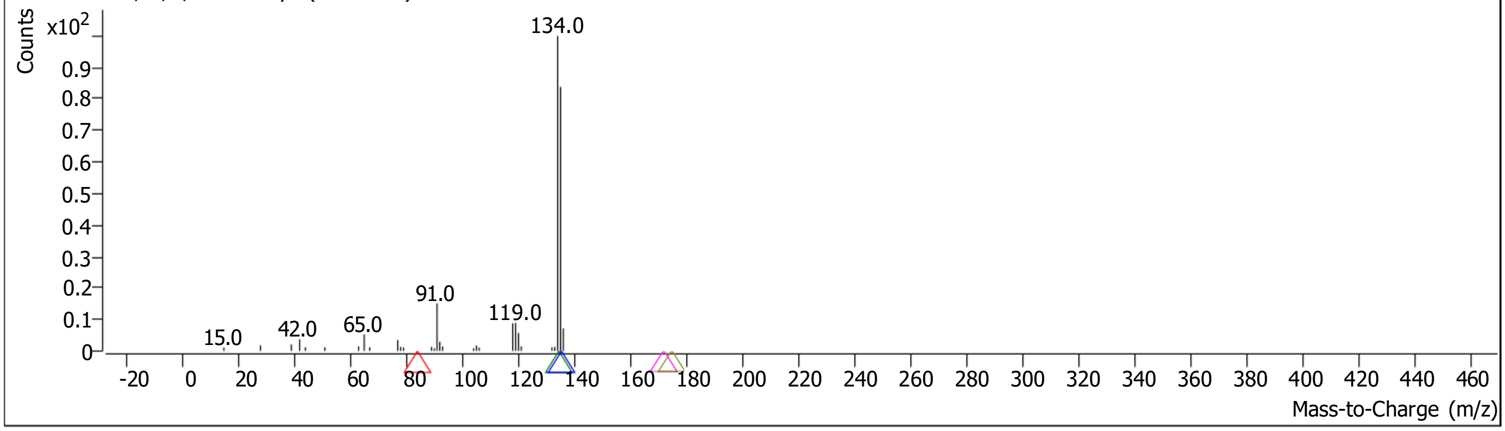


+ Scan (5.3293-5.3560 min, 15 scans) IC_E.D

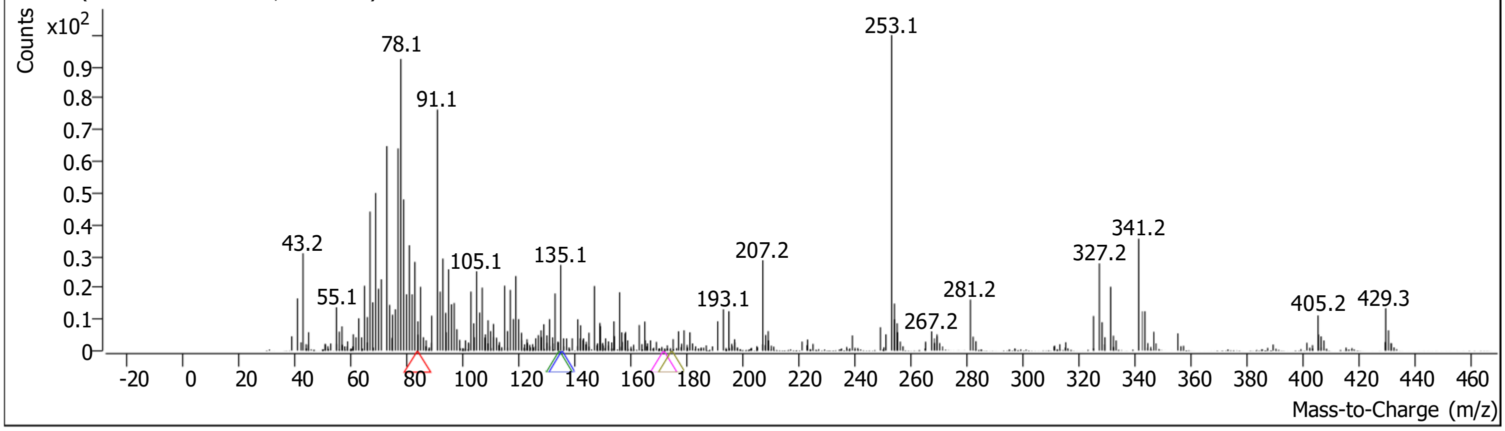


Component RT: 5.3521

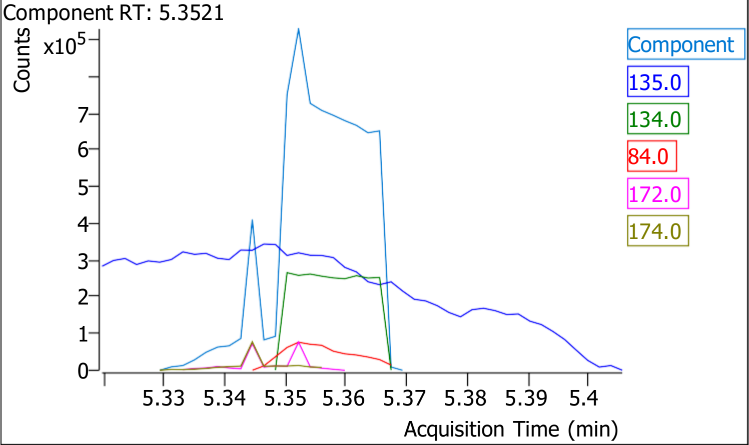


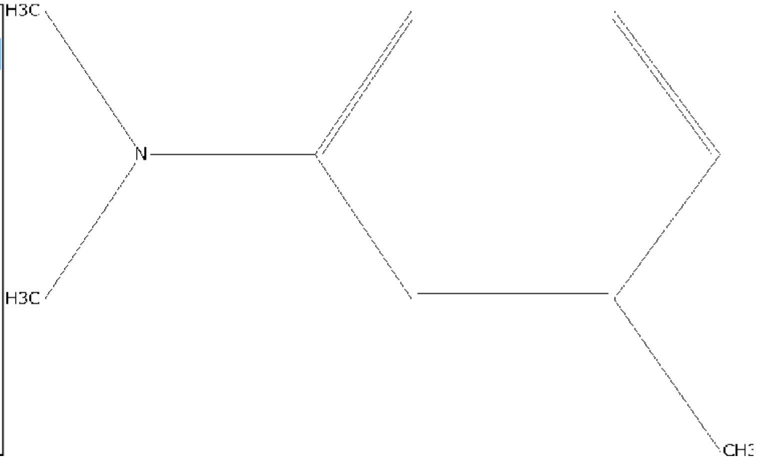

# Unknown Analysis Report - Best Hits

| RT | Compound Name | CAS# | Formula | Area | MI | Match Score | Sample | Sample |
|---|---|---|---|---|---|---|---|---|
| 5.4841 | d-Proline, N-ethoxycarbonyl-, isohexyl ester | 1000320-83-6 | C14H25NO4 | 1127826 | | 75.7 | 0.02 | 0.07 |

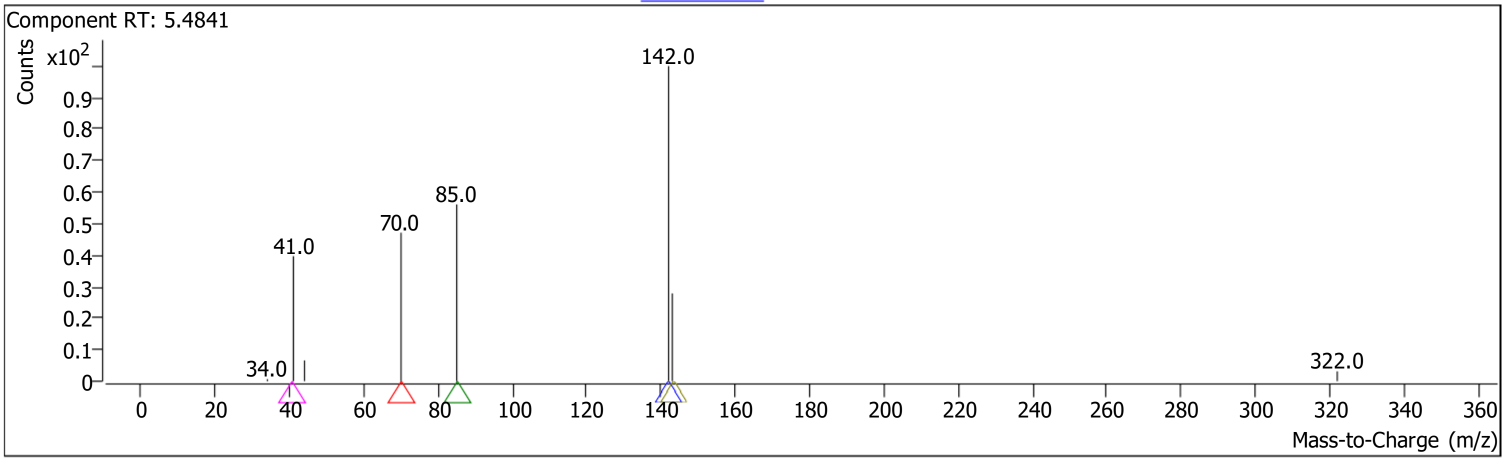


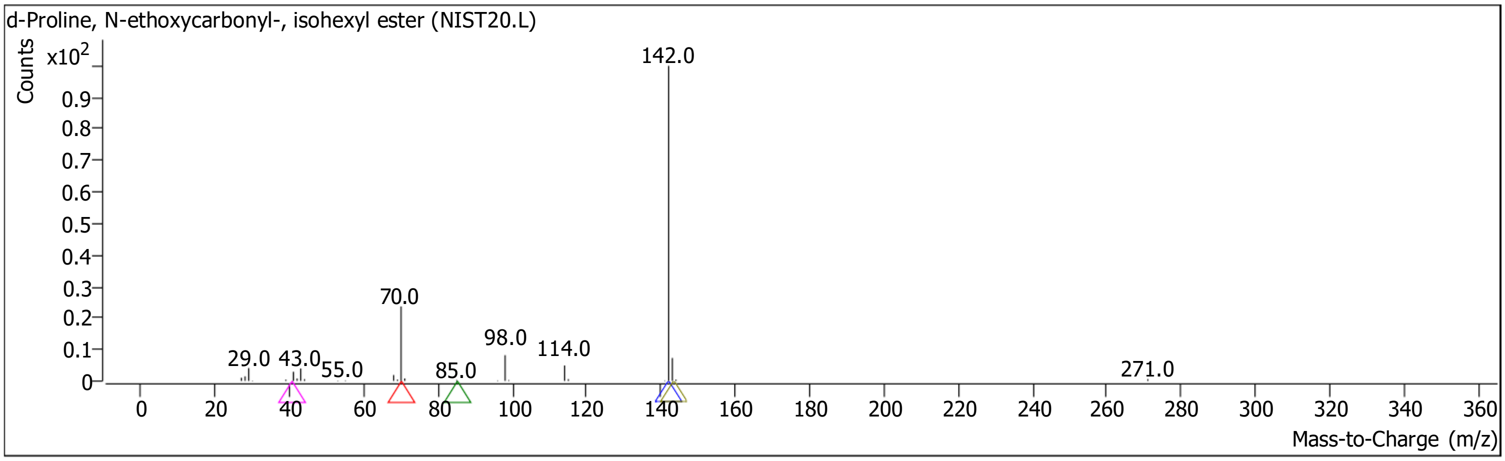


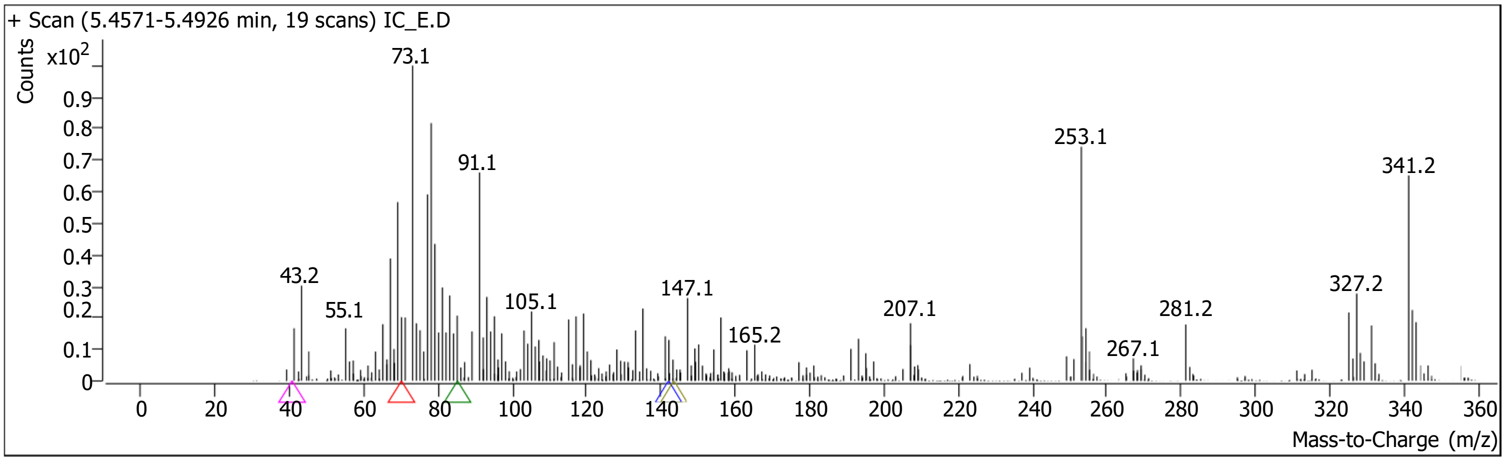


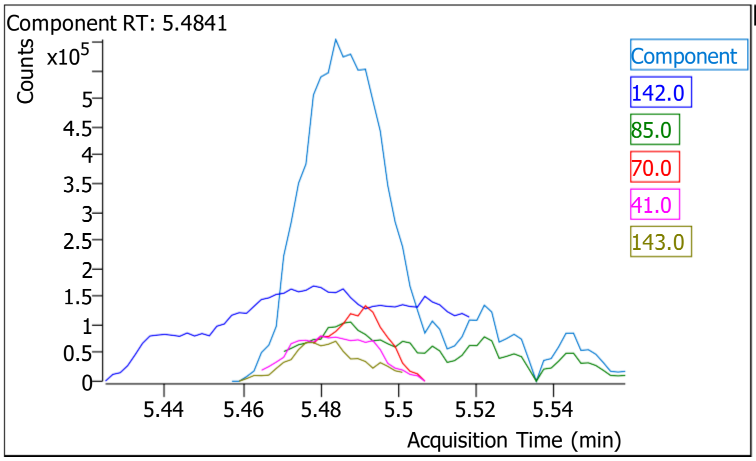


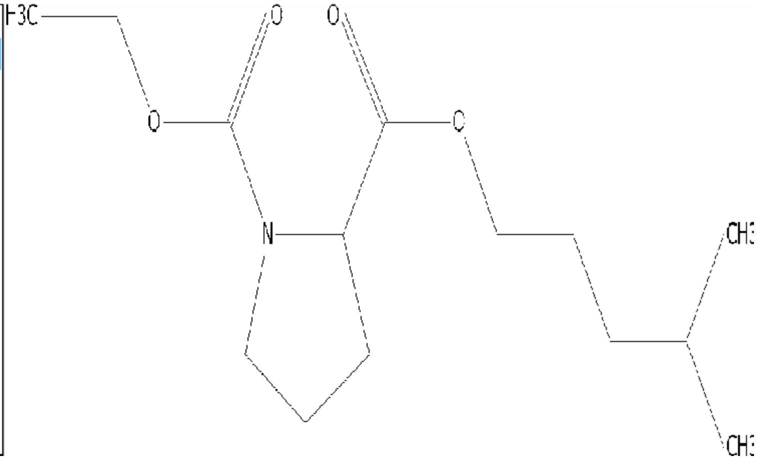


 Generated at 5:24 PM on 9/22/2025

# Unknown Analysis Report - Best Hits

| RT | Compound Name | CAS# | Formula | Area MI | Match Score | Sample | Sample |
|---|---|---|---|---|---|---|---|
| 6.5377 | 5-Methyl-furan-2-carboxylic acid (1H-[1,2,4]triazol-3-yl)-amide | 1010275-85-3 | C8H8N4O2 | 459131 | 68.0 | 0.01 | 0.03 |

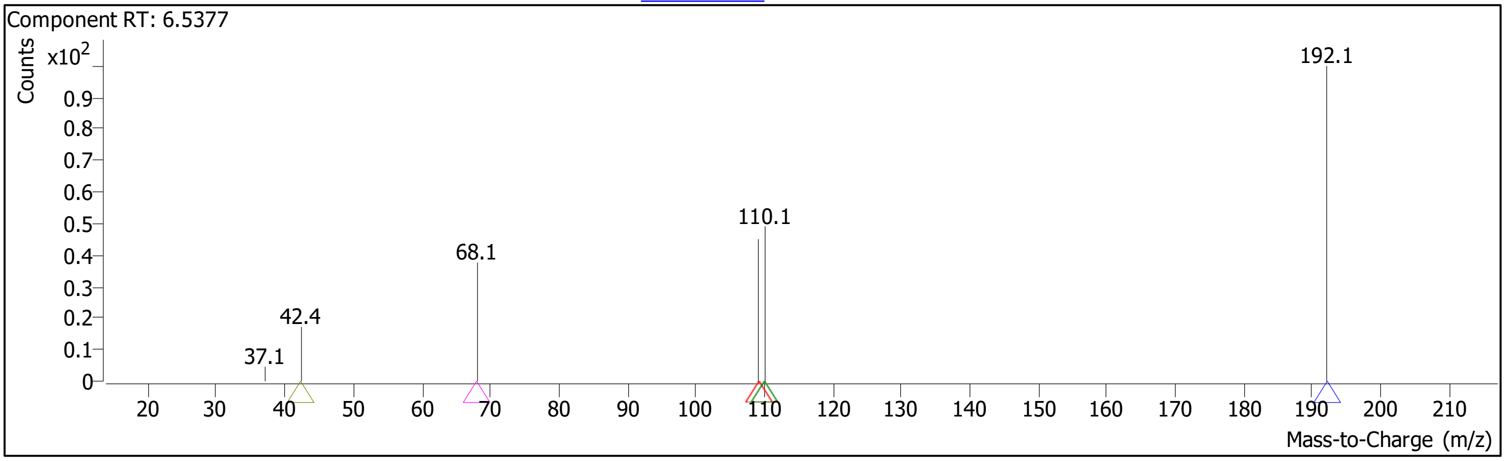


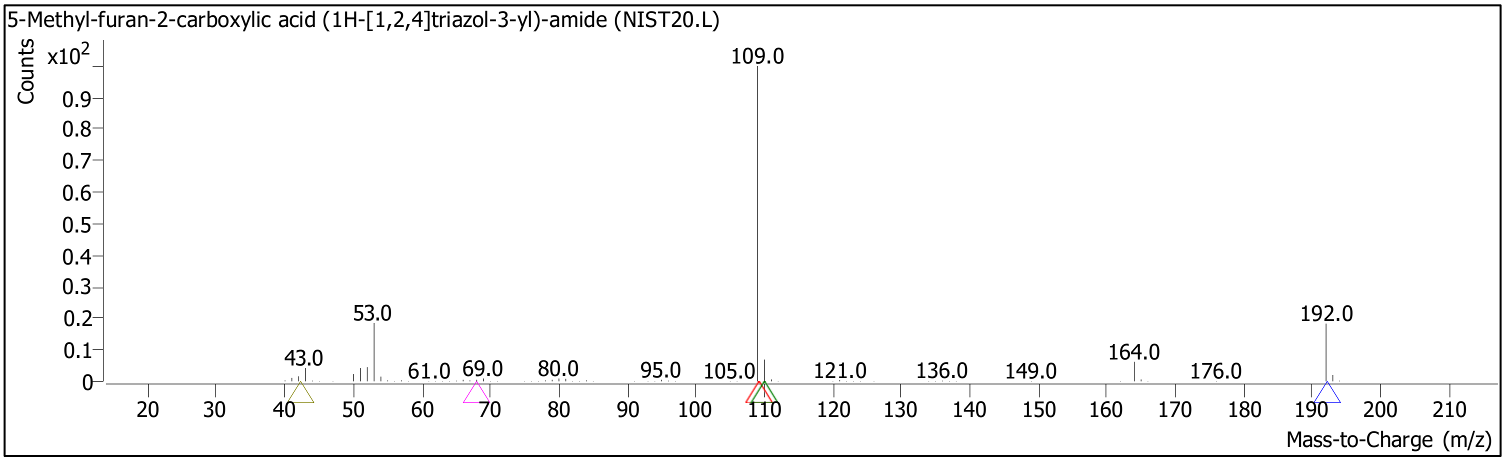


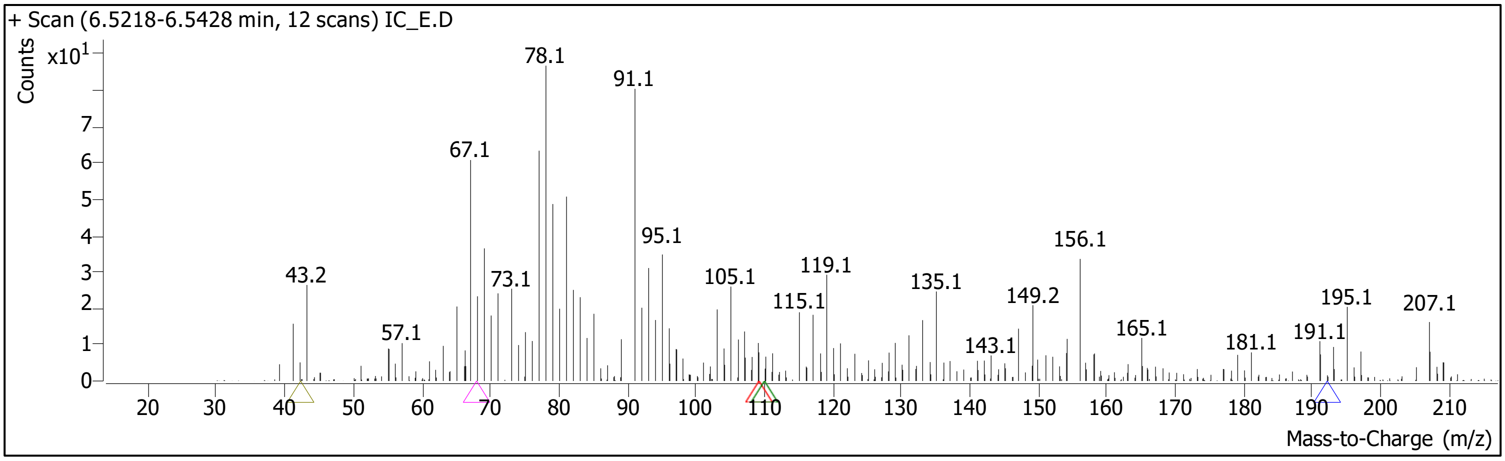


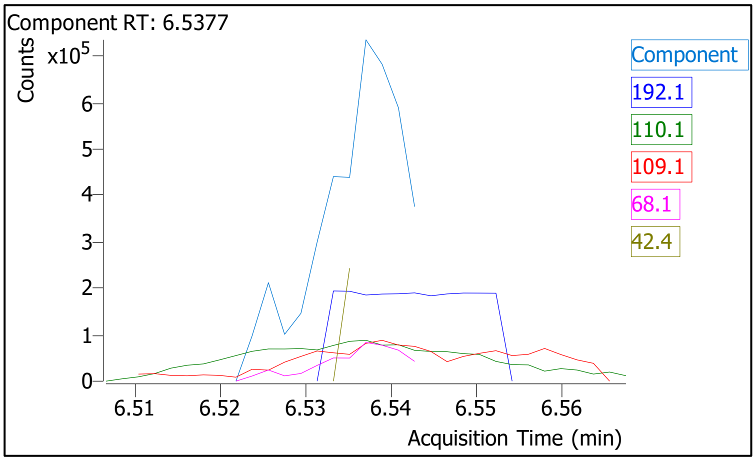


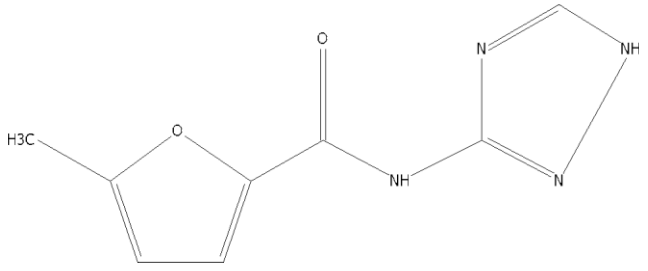

| RT | Compound Name | CAS# | Formula | Area | MI | Match Score | Sample | Sample |
|---|---|---|---|---|---|---|---|---|
| 6.9350 | o-Acetophenetidide, N-(dimethylcarbamoylmethyl)- | 97214-80-7 | C14H20N2O3 | 429897 | | 63.9 | 0.01 | 0.03 |

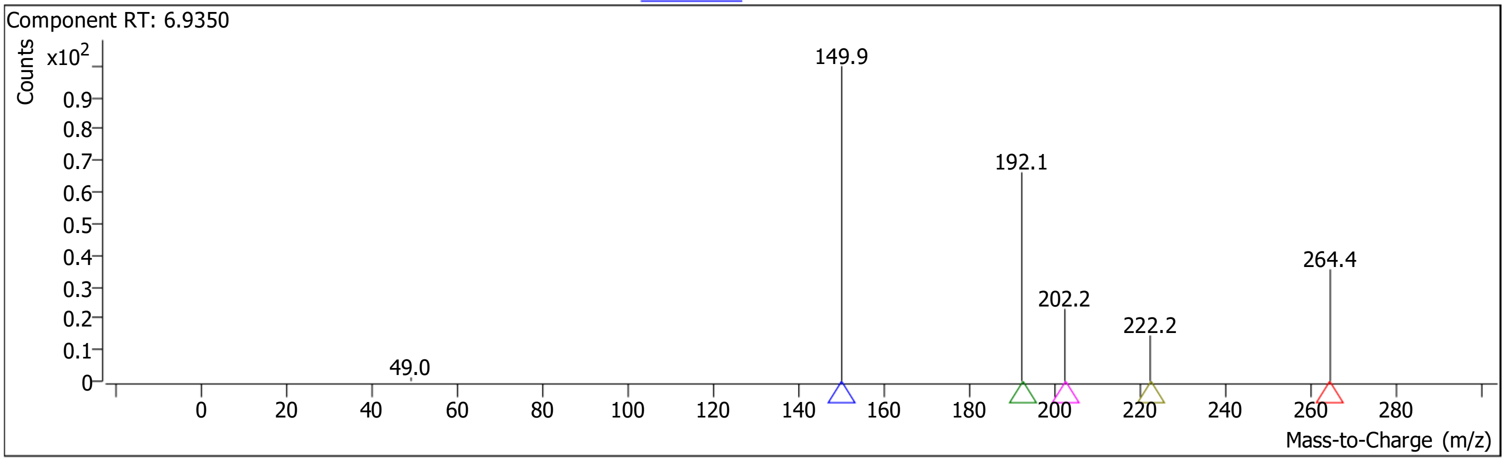


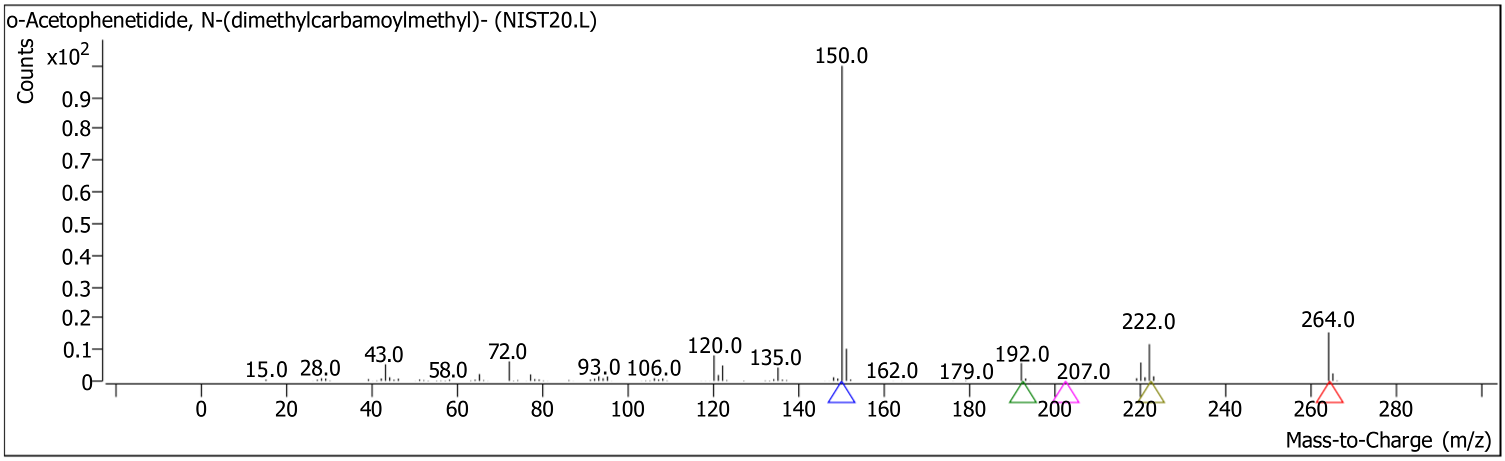


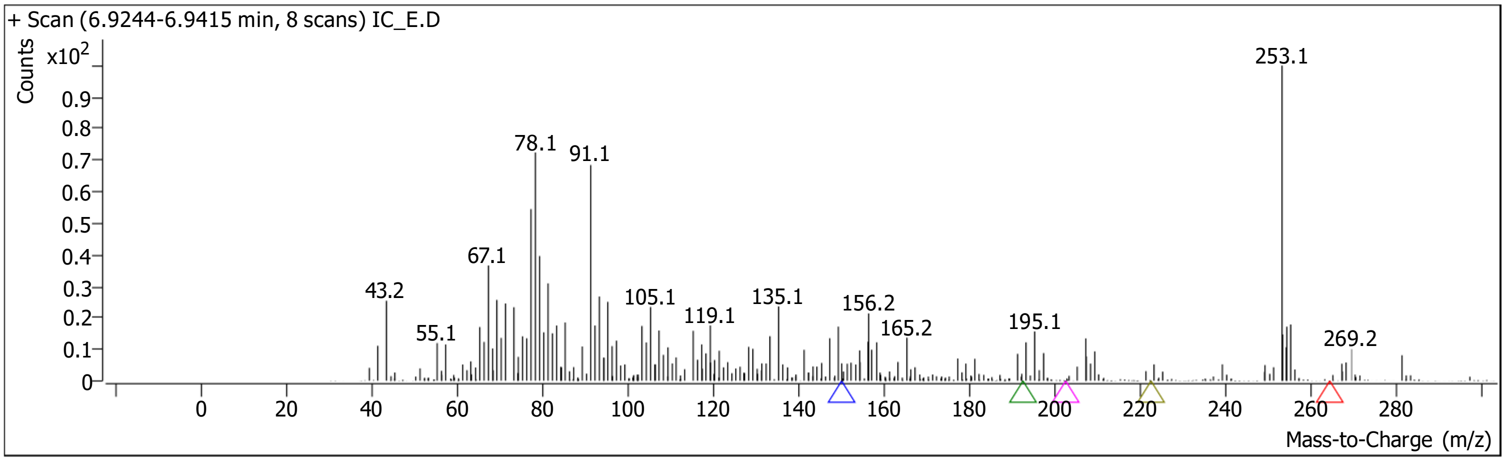


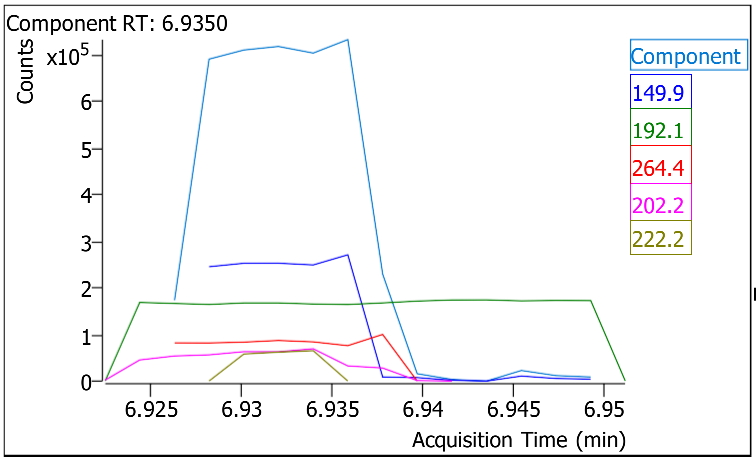


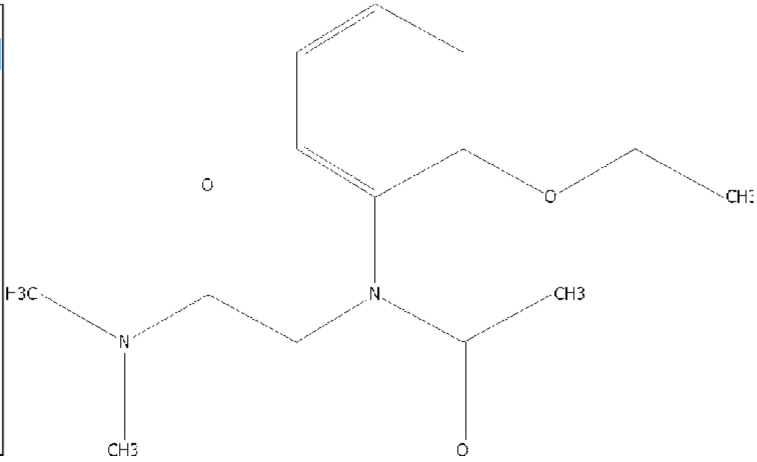

# Unknown Analysis Report - Best Hits

| RT | Compound Name | CAS# | Formula | Area | MI | Match Score | Sample | Sample |
|---|---|---|---|---|---|---|---|---|
| 7.5483 | 2-Ethylbutyric acid, tetrahydrofurfuryl ester | 1000370-65-1 | C11H20O3 | 14419760 | | 68.8 | 0.21 | 0.94 |

Component RT: 7.5483

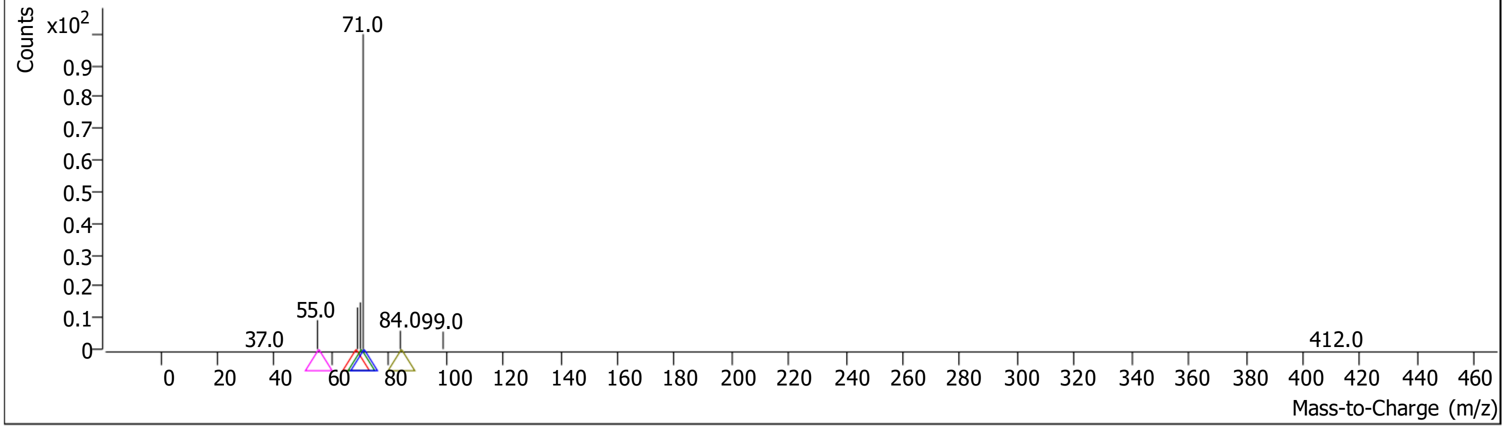


2-Ethylbutyric acid, tetrahydrofurfuryl ester (NIST20.L)

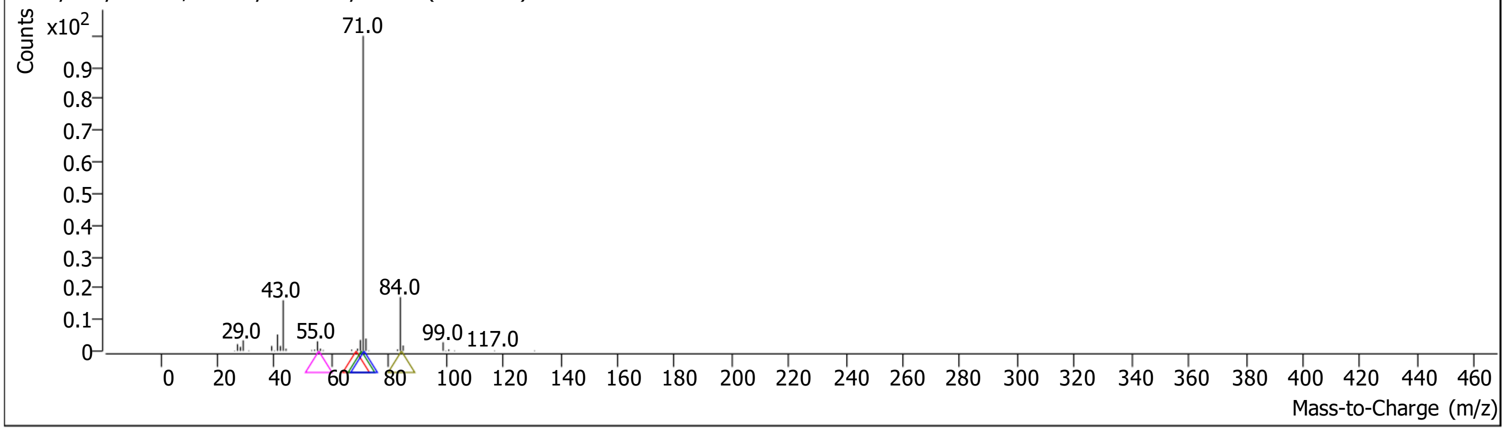


+ Scan (7.5140-7.6029 min, 47 scans) IC_E.D

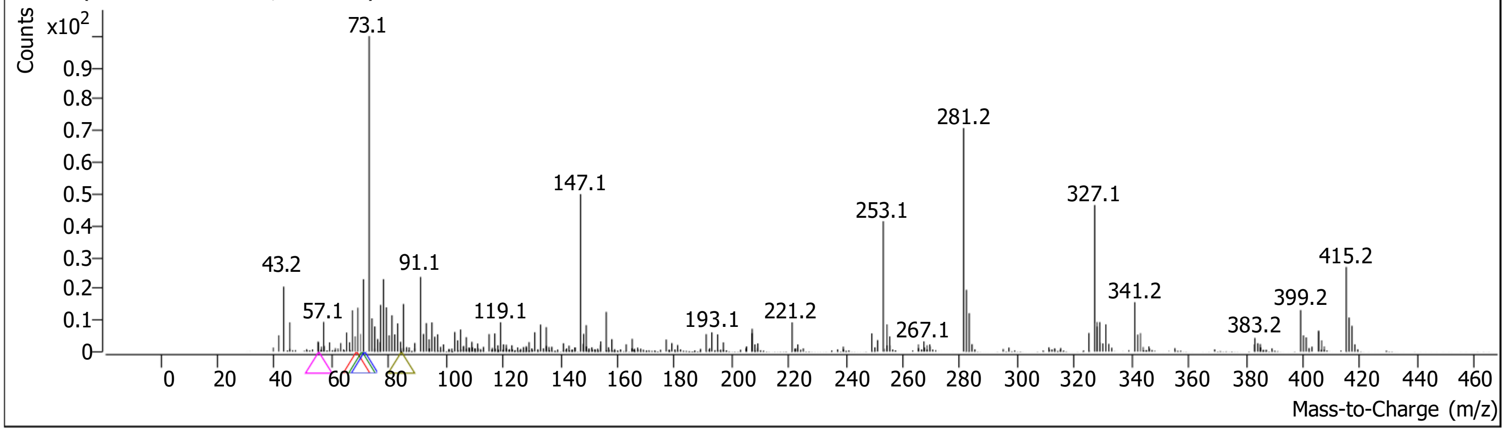


Component RT: 7.5483

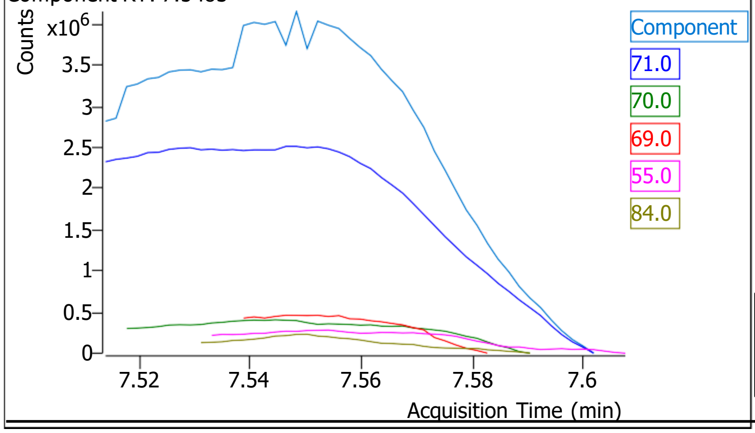


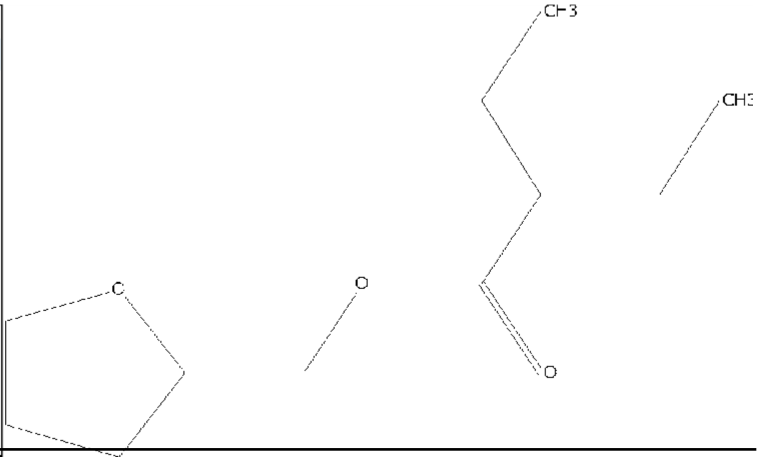


Acquisition Time (min)

| RT | Compound Name | CAS# | Formula | Area | MI | Match Score | Sample | Sample |
|---|---|---|---|---|---|---|---|---|
| 7.7395 | 2,4-Di-tert-butylphenol | 96-76-4 | C14H22O | 1421709555 | | 93.0 | 20.58 | 92.93 |

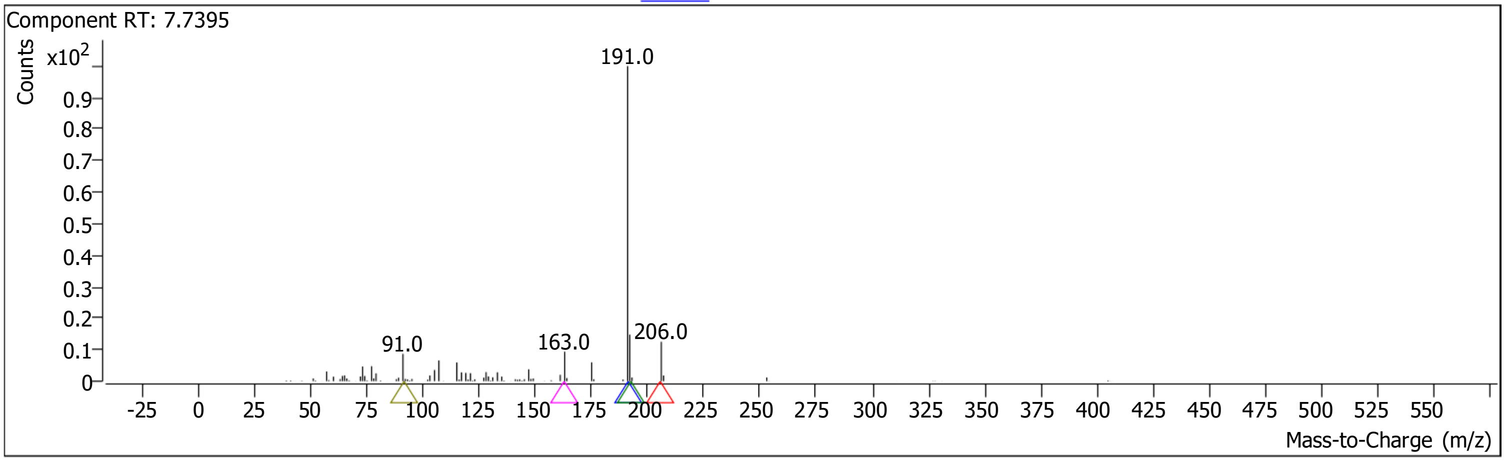


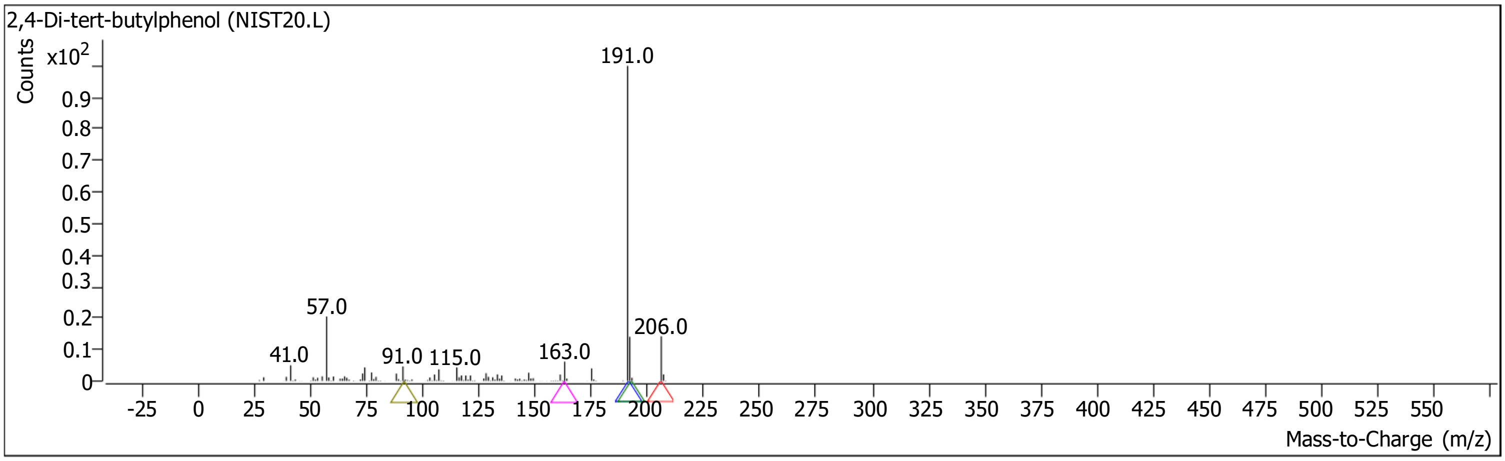


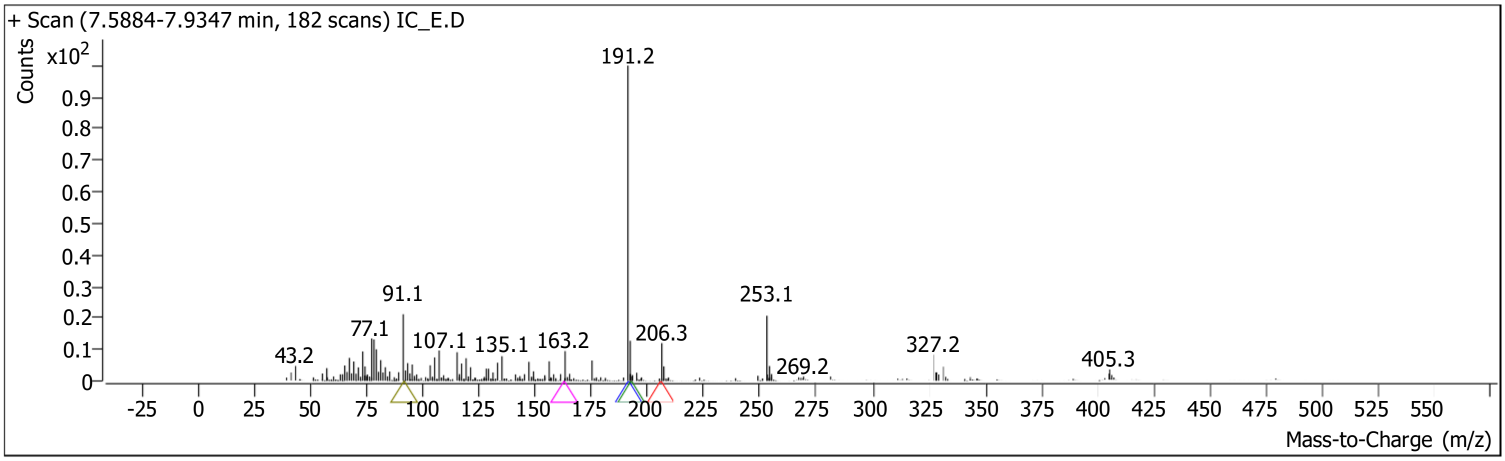


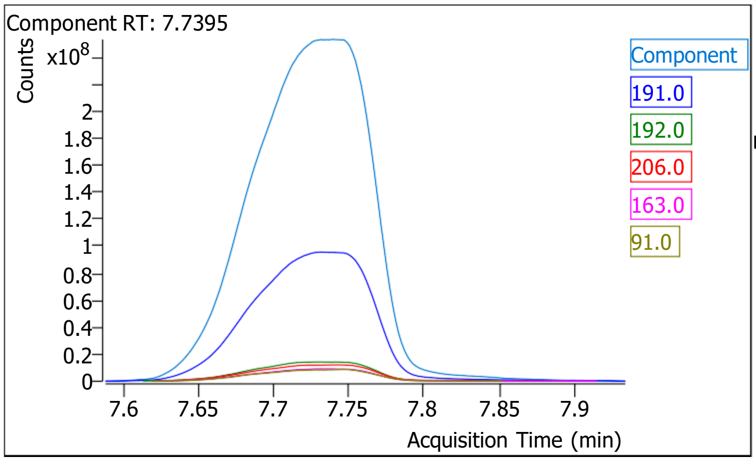


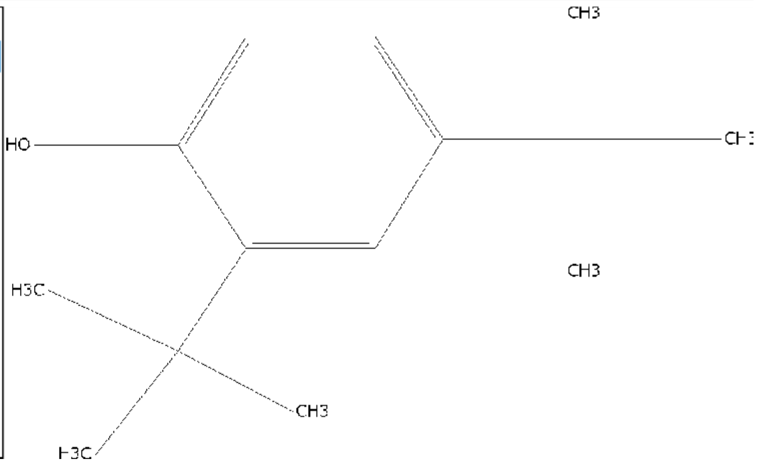


 Generated at 5:24 PM on 9/22/2025

| RT | Compound Name | CAS# | Formula | Area MI | Match Score | Sample | Sample |
|---|---|---|---|---|---|---|---|
| 7.9937 | 3,5-Dihydroxybenzhydrazide | 7732-32-3 | C7H8N2O3 | 8723291 | 63.6 | 0.13 | 0.57 |

Component RT: 7.9937

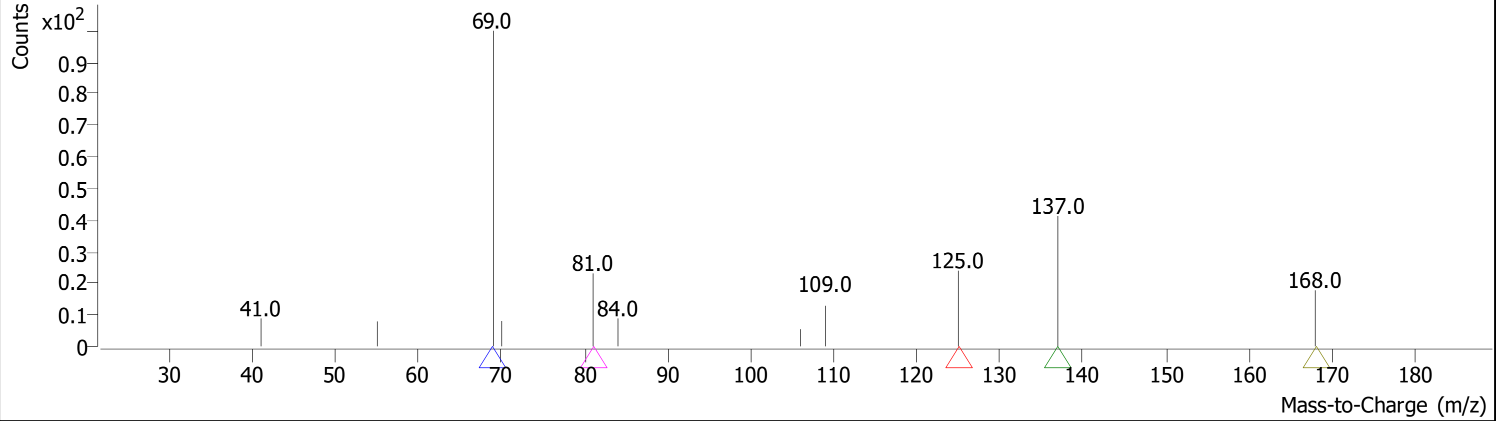


3,5-Dihydroxybenzhydrazide (NIST20.L)

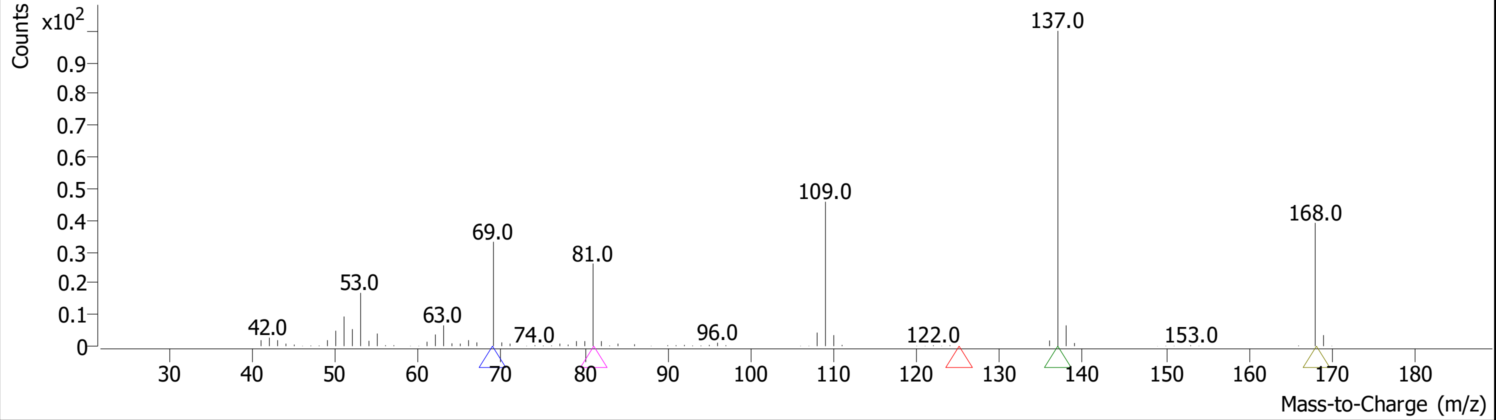


+ Scan (7.9813-8.0044 min, 13 scans) IC_E.D

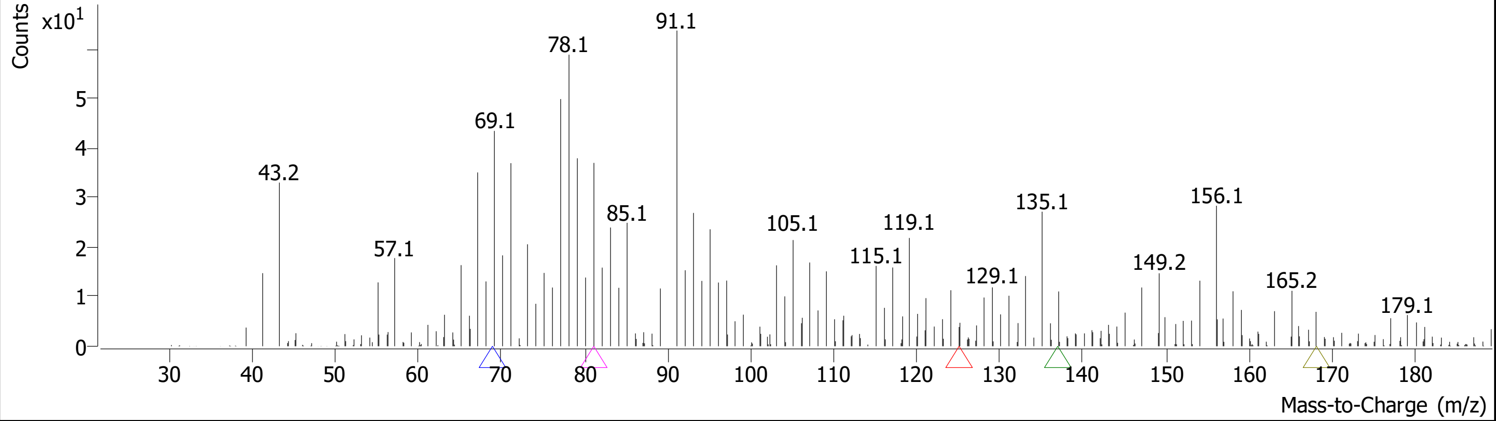


Component RT: 7.9937

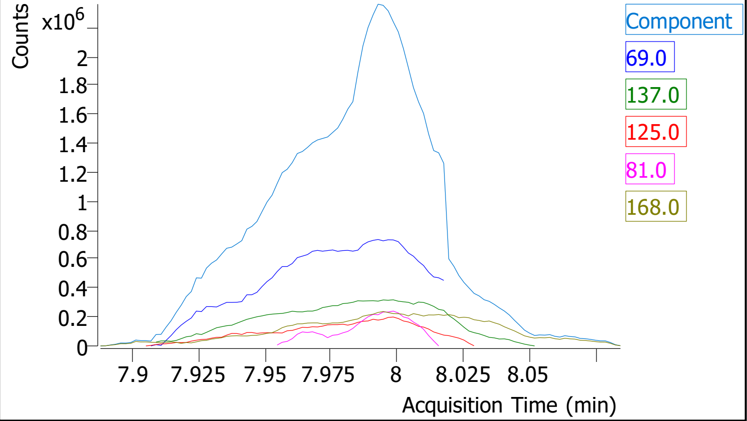


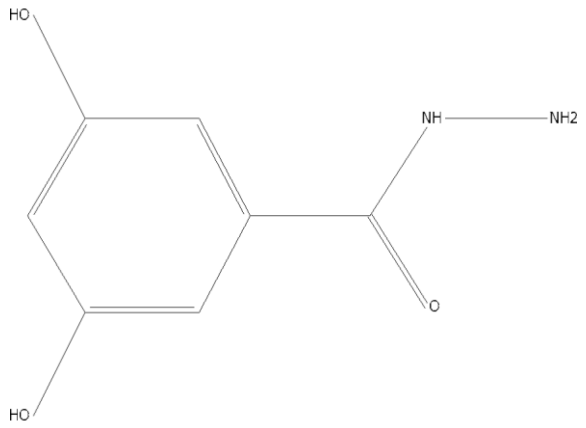


 

# Unknown Analysis Report - Best Hits

| RT | Compound Name | CAS# | Formula | Area | MI | Match Score | Sample | Sample |
|---|---|---|---|---|---|---|---|---|
| 8.1749 | 2(4H)-Benzofuranone, 5,6,7,7a-tetrahydro-4,4,7a-trimethyl-, (R)- | 17092-92-1 | C11H16O2 | 44248161 | | 60.2 | 0.64 | 2.89 |

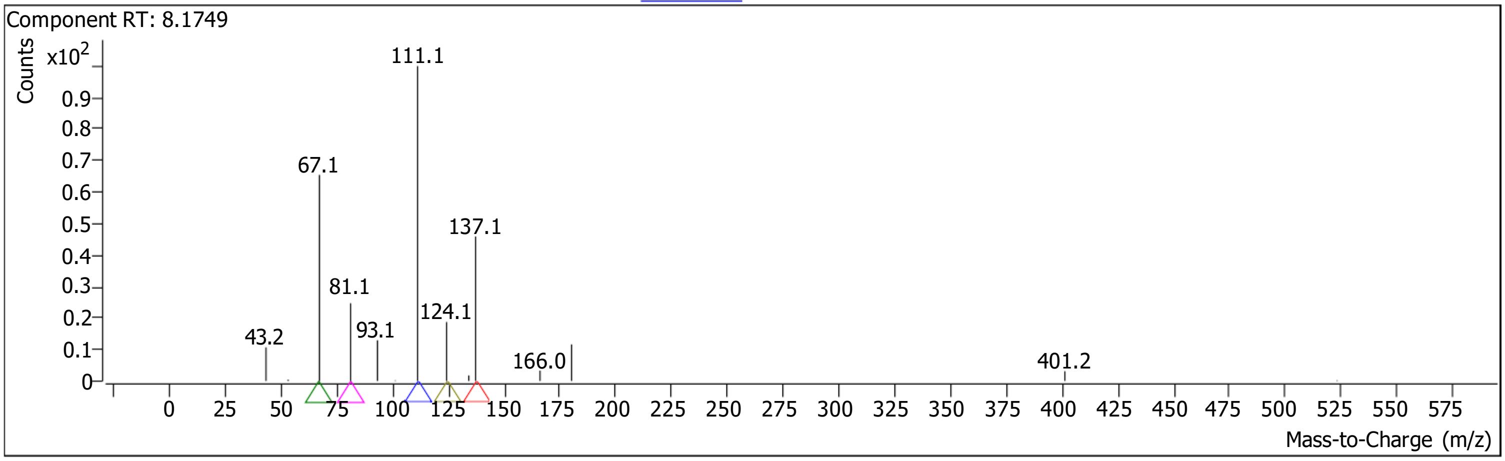


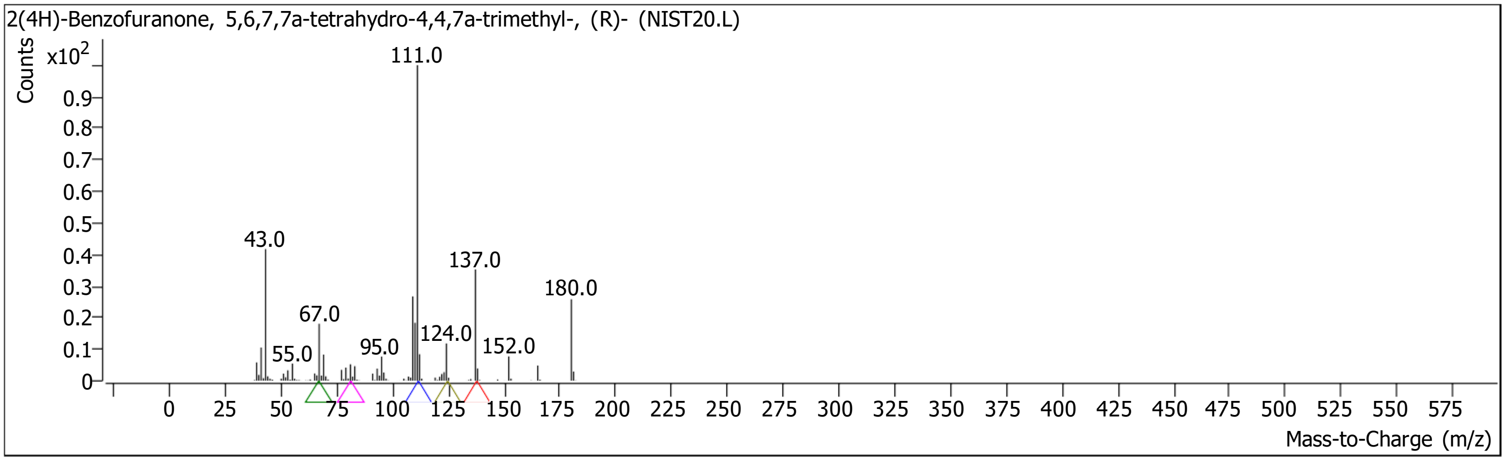


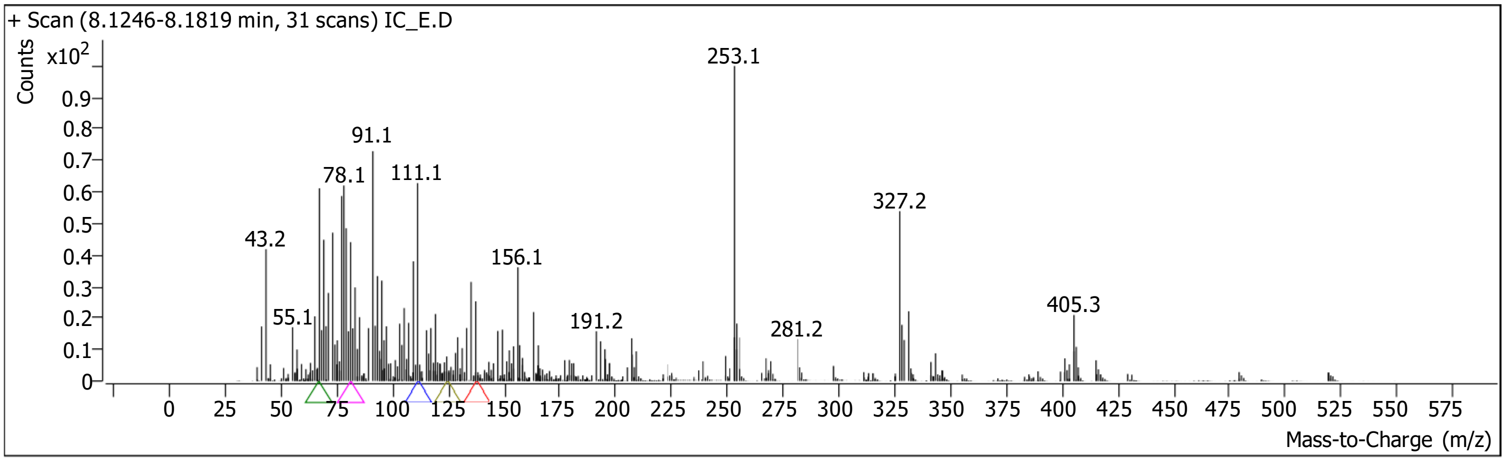


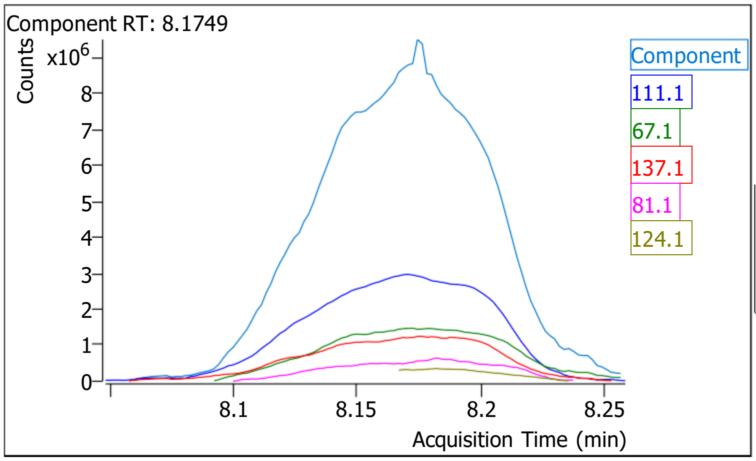


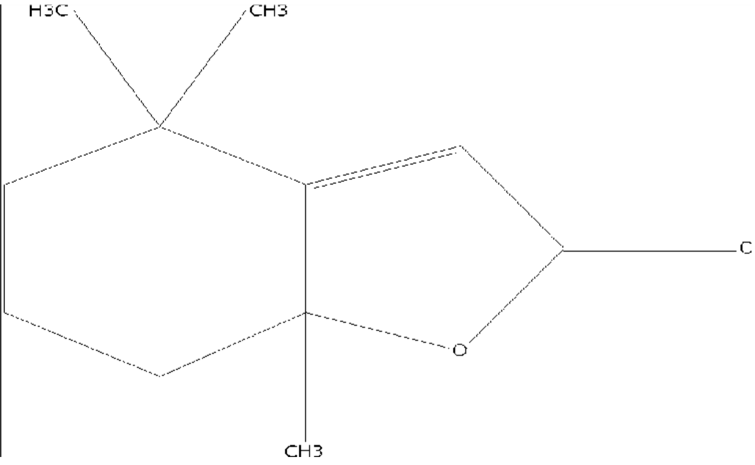


 Generated at 5:24 PM on 9/22/2025

| RT | Compound Name | CAS# | Formula | Area | MI | Match Score | Sample | Sample |
|---|---|---|---|---|---|---|---|---|
| 8.5007 | 1-Naphthalenesulfonic acid, 5-(dimethylamino)-, phenyl ester | 55837-12-2 | C18H17NO3S | 1340026 | | 80.1 | 0.02 | 0.09 |

Component RT: 8.5007

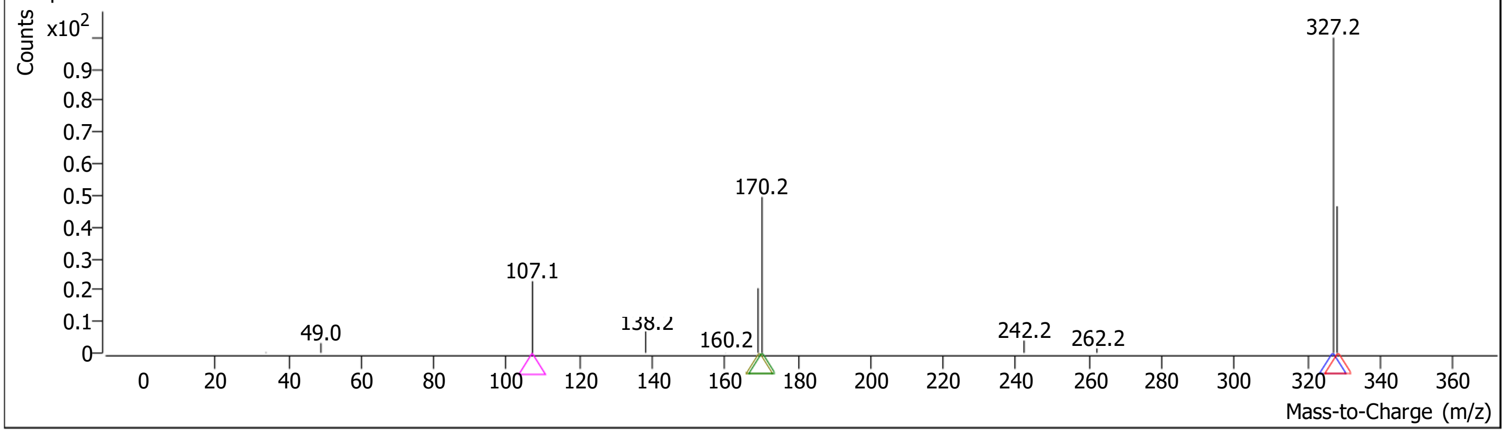


1-Naphthalenesulfonic acid, 5-(dimethylamino)-, phenyl ester (NIST20.L)

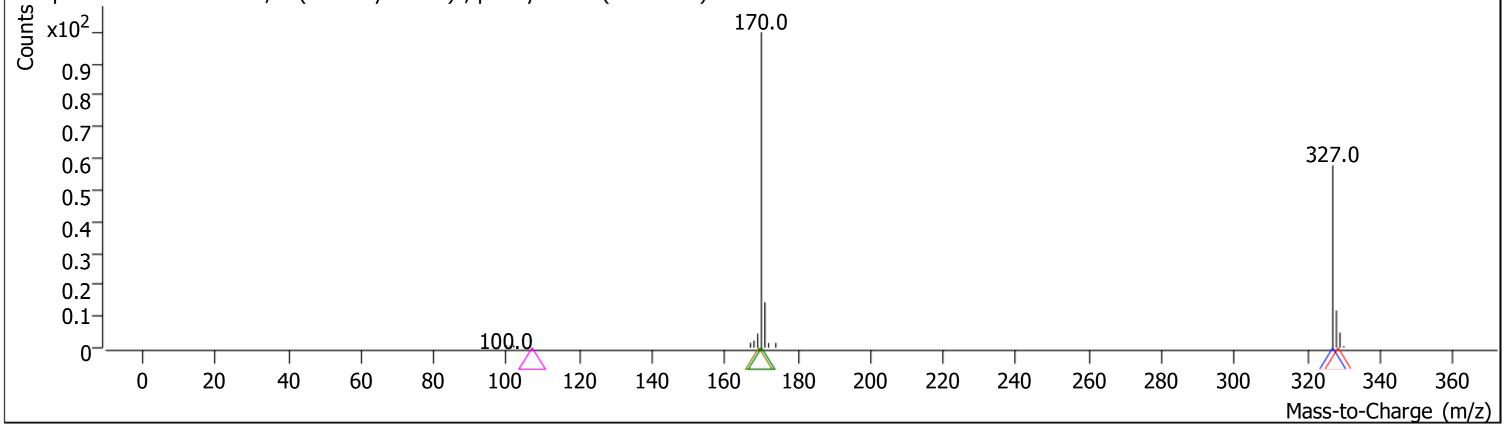


+ Scan (8.4951-8.5137 min, 9 scans) IC_E.D

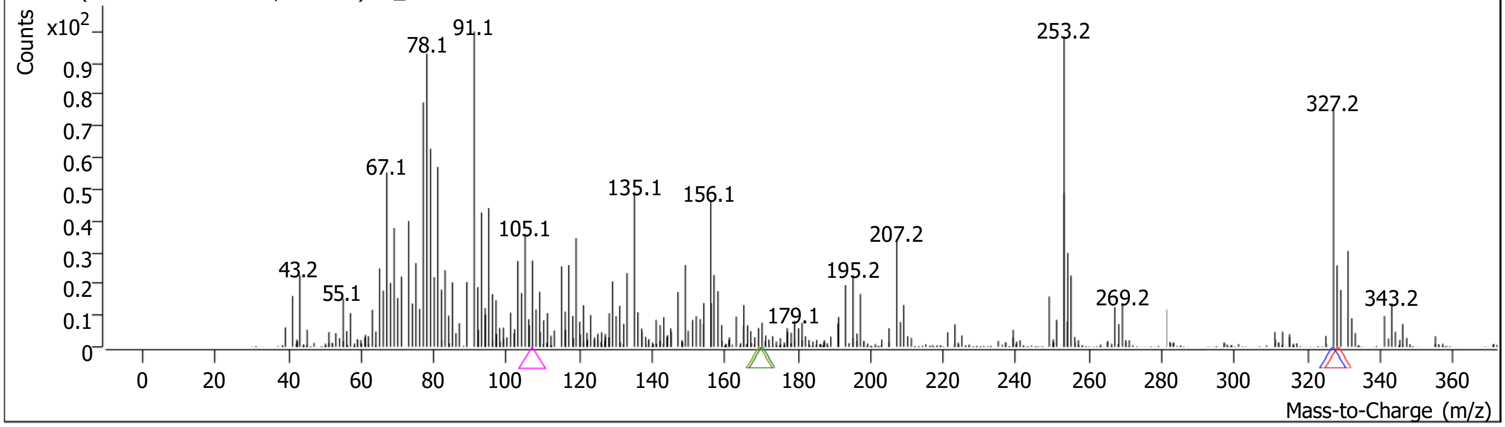


Component RT: 8.5007

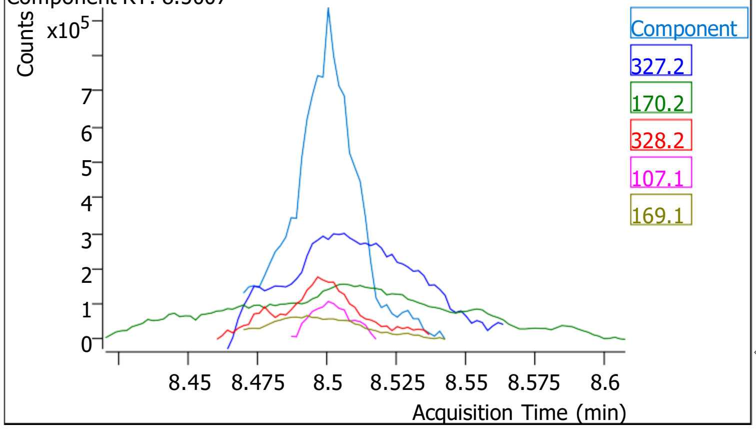


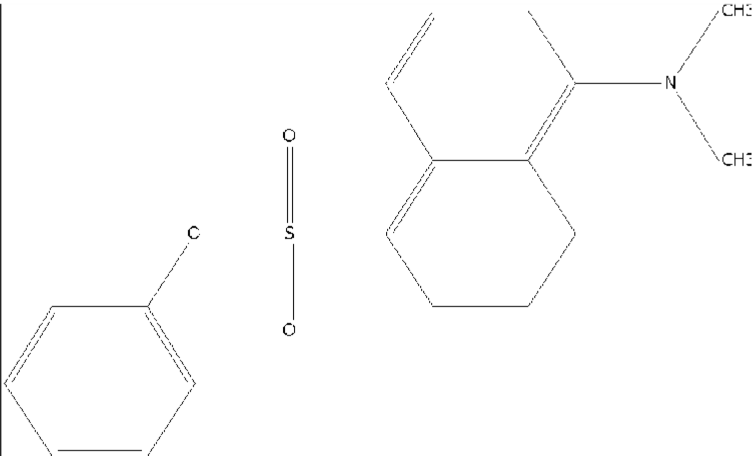

# Unknown Analysis Report - Best Hits

| RT | Compound Name | CAS# | Formula | Area MI | Match Score | Sample | Sample |
|---|---|---|---|---|---|---|---|
| 9.1452 | p-Octylacetophenone | 10541-56-7 | C16H24O | 14607422 | 64.8 | 0.21 | 0.95 |

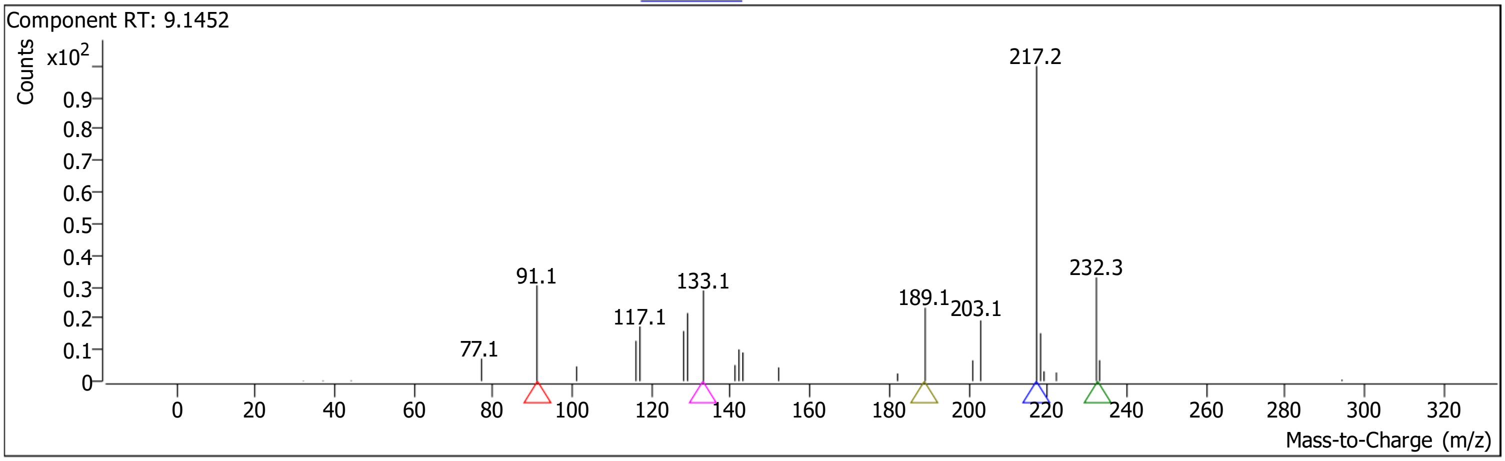


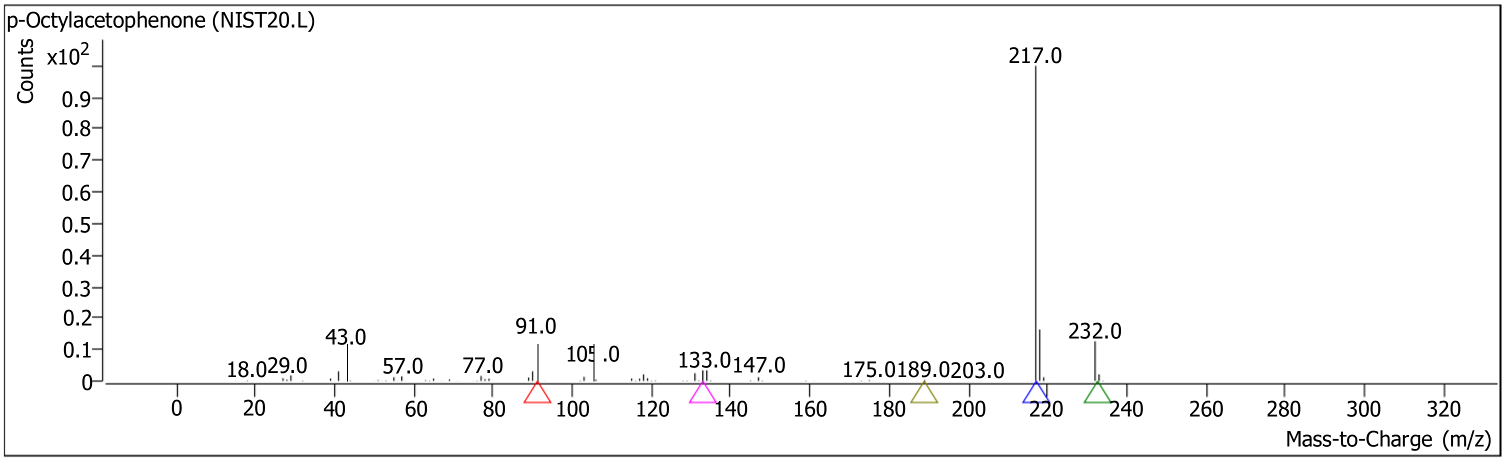


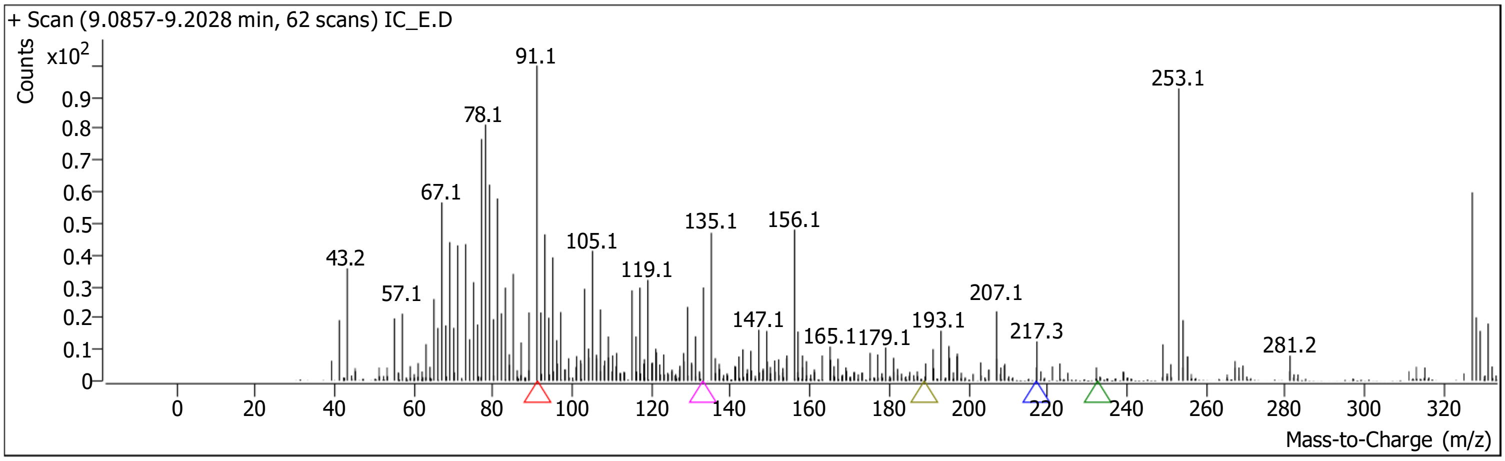


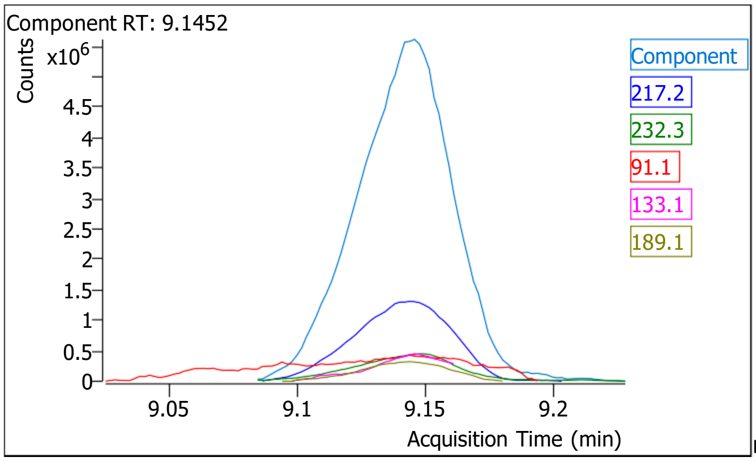


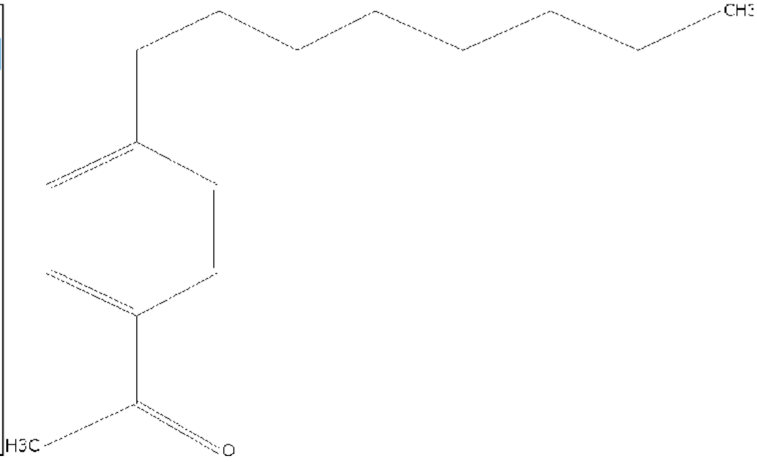

| RT | Compound Name | CAS# | Formula | Area | MI | Match Score | Sample | Sample |
|---|---|---|---|---|---|---|---|---|
| 9.7511 | 9H-Fluoren-3-ol, 9,9-dimethyl- | 1000141-55-1 | C15H14O | 10495502 | | 60.6 | 0.15 | 0.69 |

Component RT: 9.7511

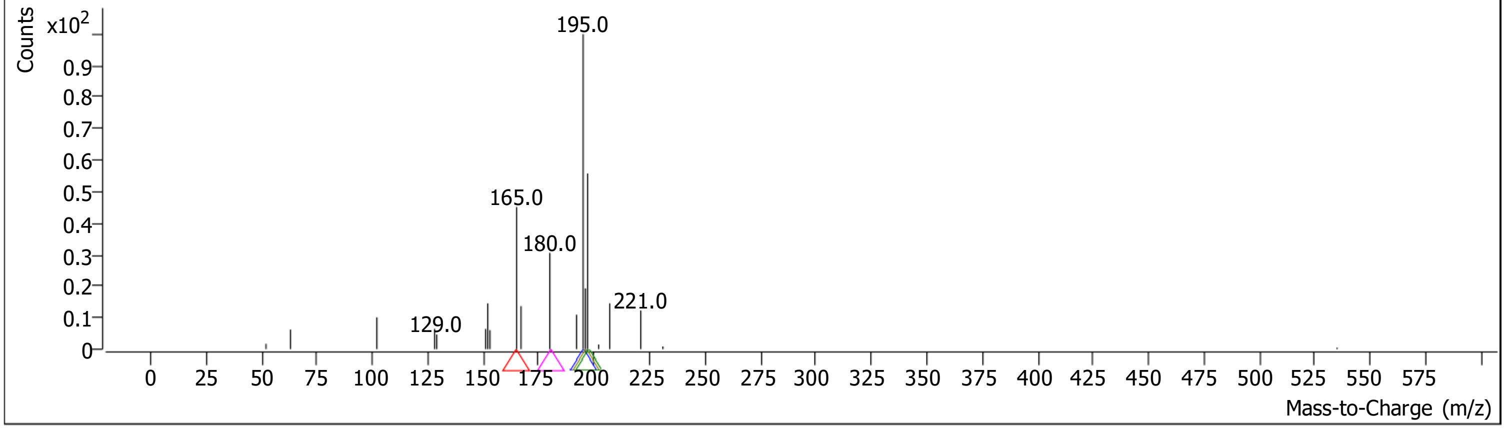


9H-Fluoren-3-ol, 9,9-dimethyl- (NIST20.L)

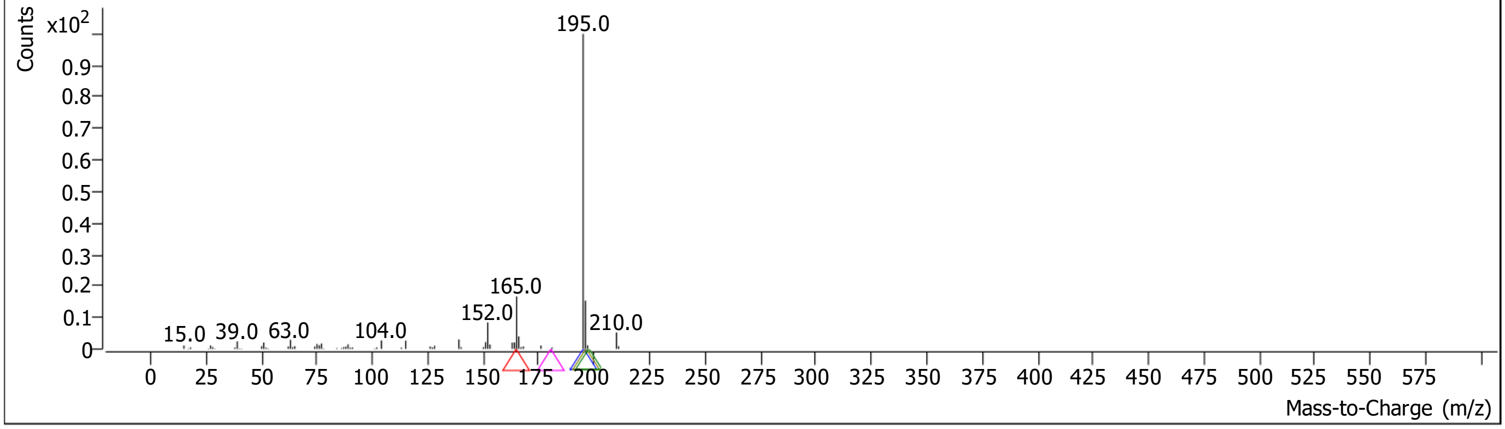


+ Scan (9.7447-9.7562 min, 7 scans) IC_E.D

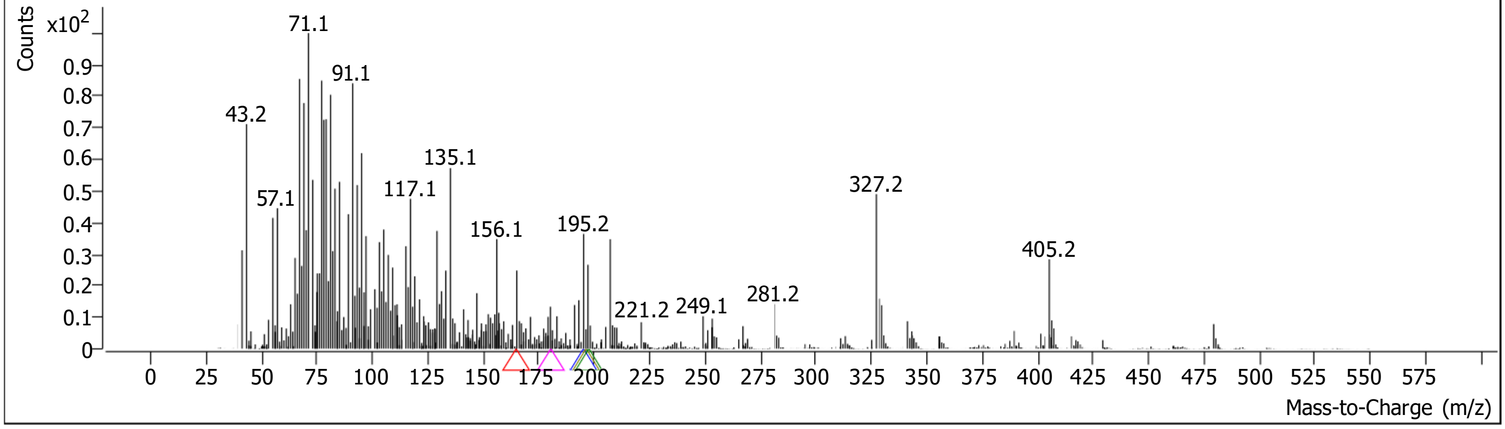


Component RT: 9.7511

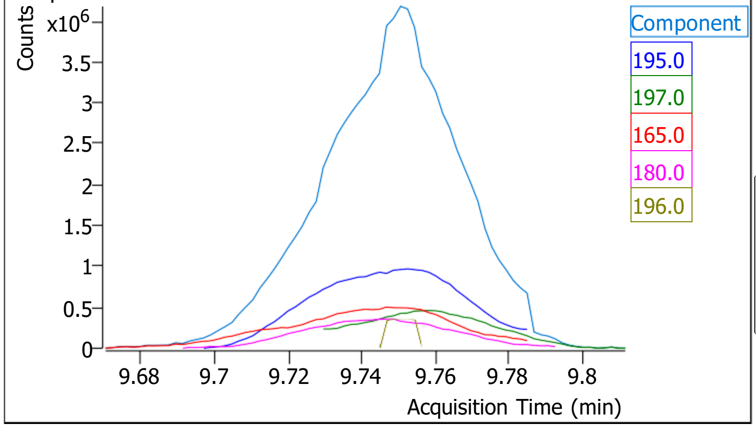


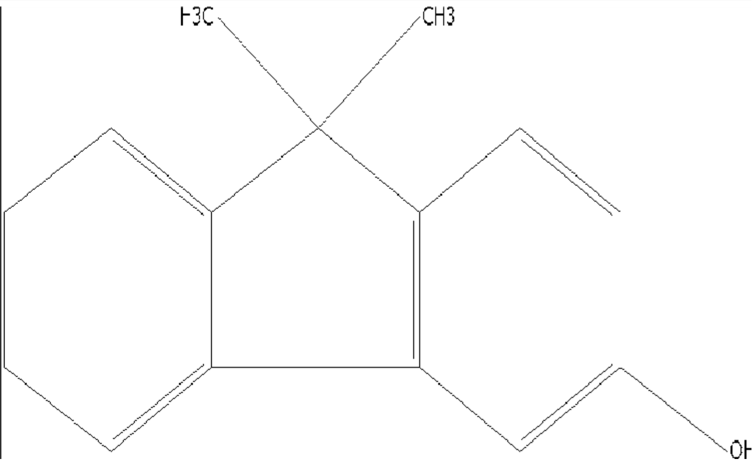

| RT | Compound Name | CAS# | Formula | Area | MI | Match Score | Sample | Sample |
|---|---|---|---|---|---|---|---|---|
| 10.0084 | Benzoic acid, 2-ethylhexyl ester | 5444-75-7 | C15H22O2 | 84319768 | | 87.5 | 1.22 | 5.51 |

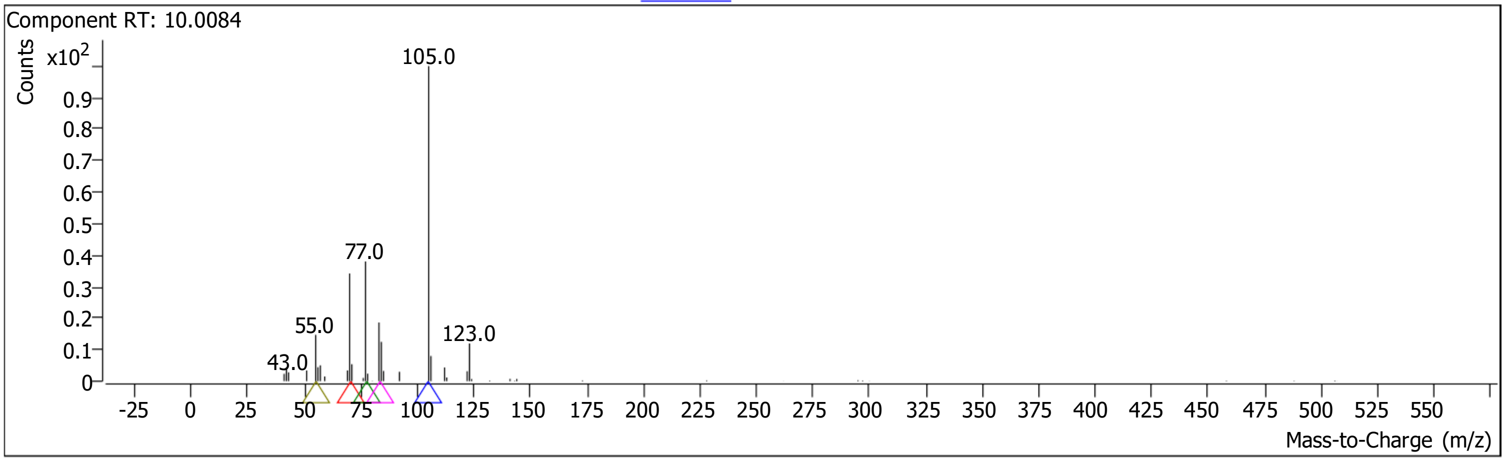


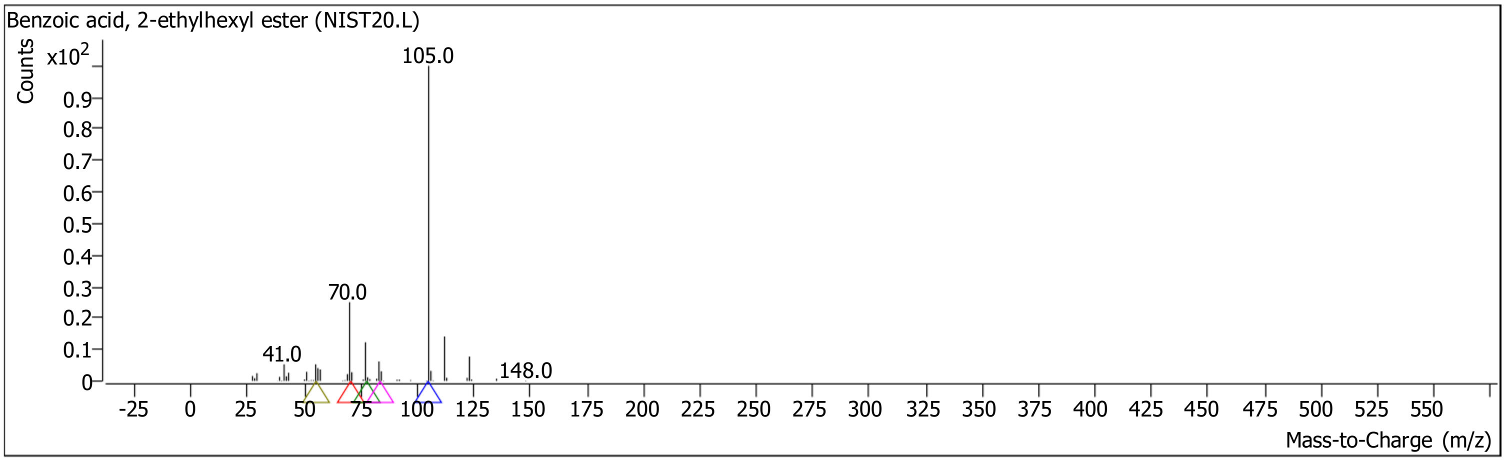


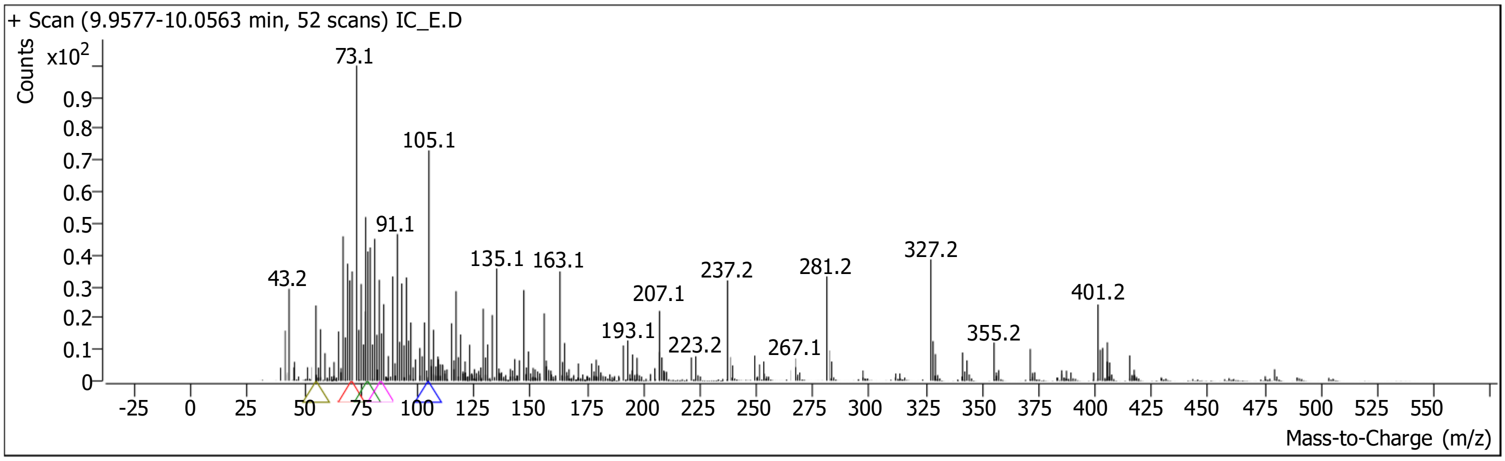


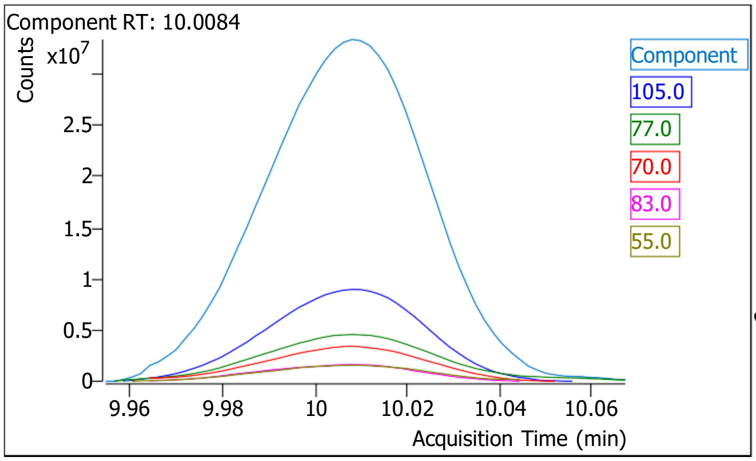


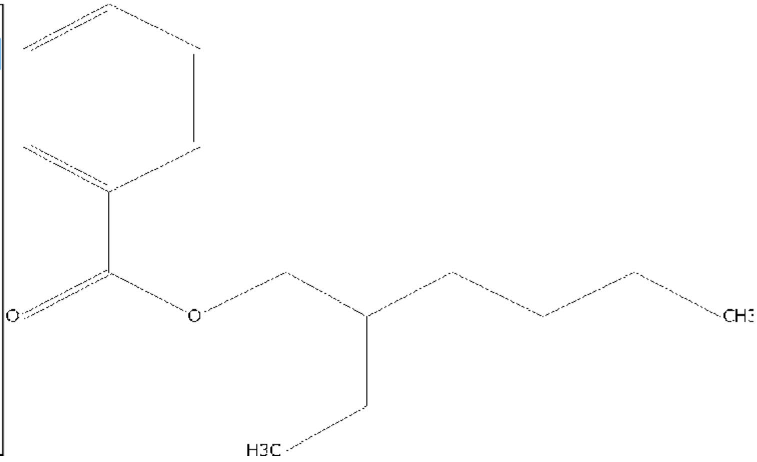

| RT | Compound Name | CAS# | Formula | Area | MI | Match Score | Sample | Sample |
|---|---|---|---|---|---|---|---|---|
| 10.7158 | Loliolide | 5989-02-6 | C11H16O3 | 28394896 | | 73.3 | 0.41 | 1.86 |

Component RT: 10.7158

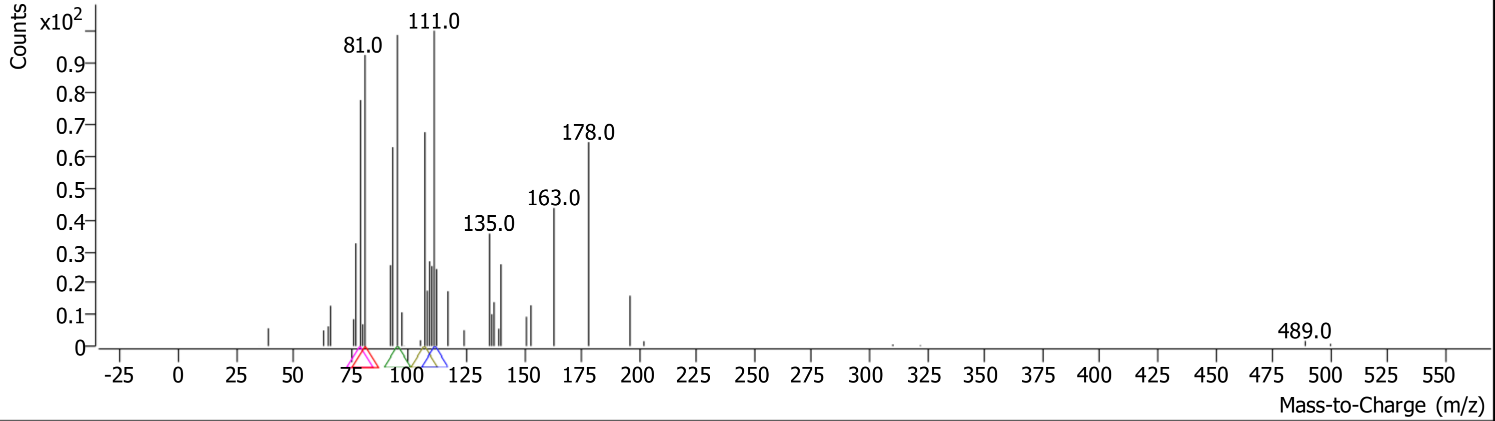


Loliolide (NIST20.L)

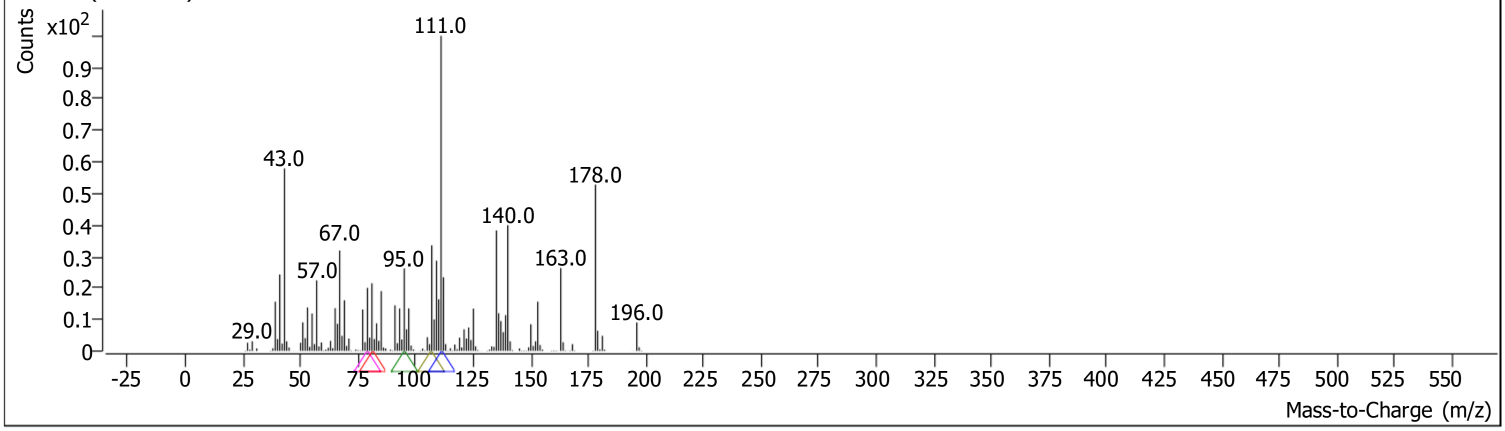


+ Scan (10.6894-10.7161 min, 14 scans) IC_E.D

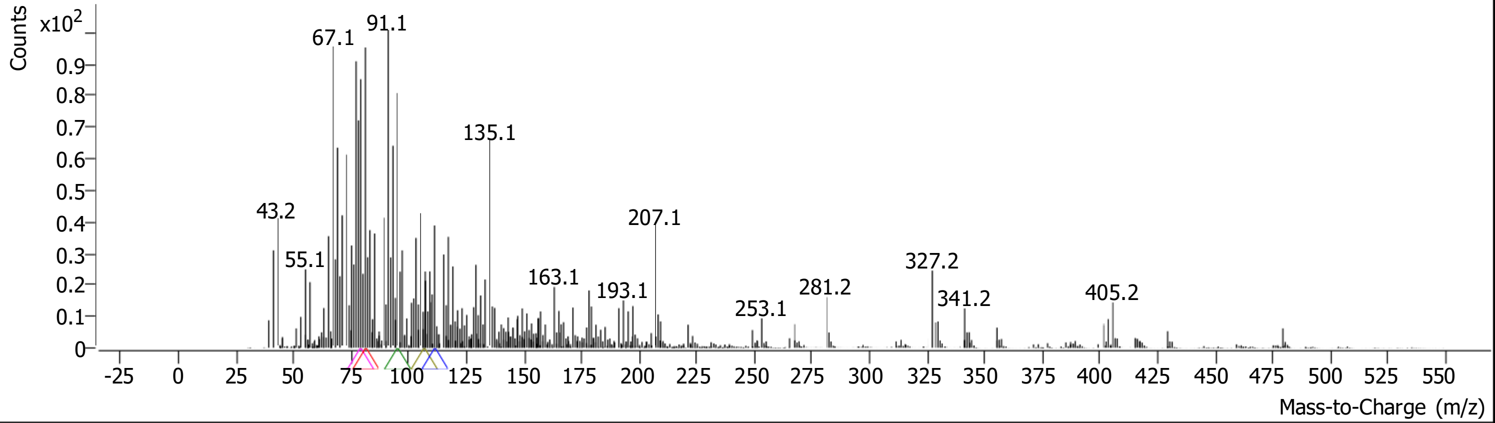


Component RT: 10.7158

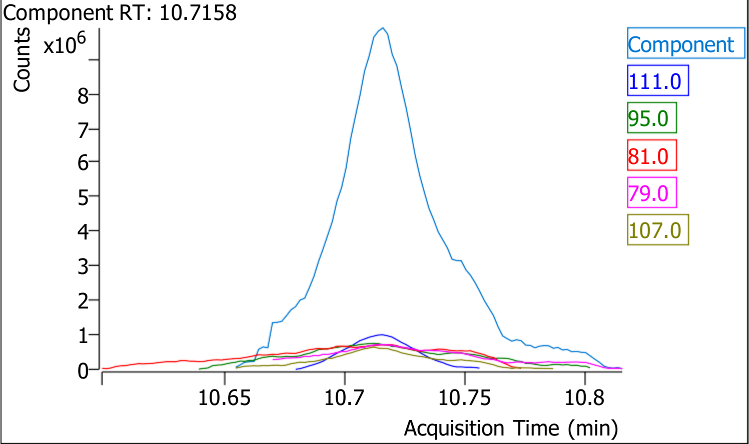


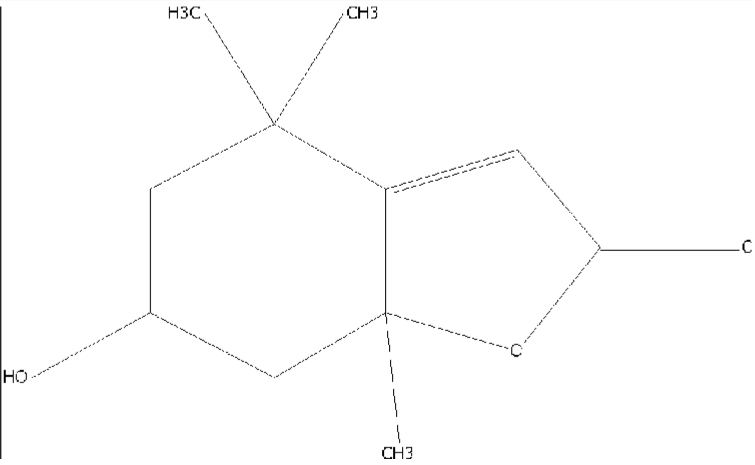

# Unknown Analysis Report - Best Hits

| RT | Compound Name | CAS# | Formula | Area | MI | Match Score | Sample | Sample |
|---|---|---|---|---|---|---|---|---|
| 10.7865 | 2-Ethyl-5-propylcyclopentanone | 38468-47-2 | C10H18O | 3770899 | | 60.2 | 0.05 | 0.25 |

Component RT: 10.7865

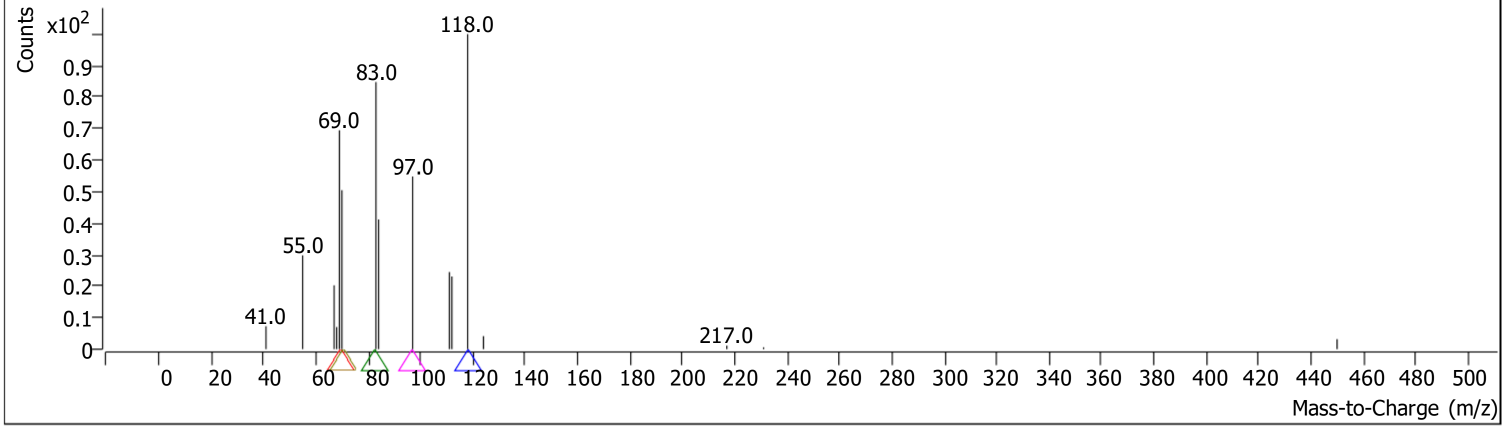


2-Ethyl-5-propylcyclopentanone (NIST20.L)

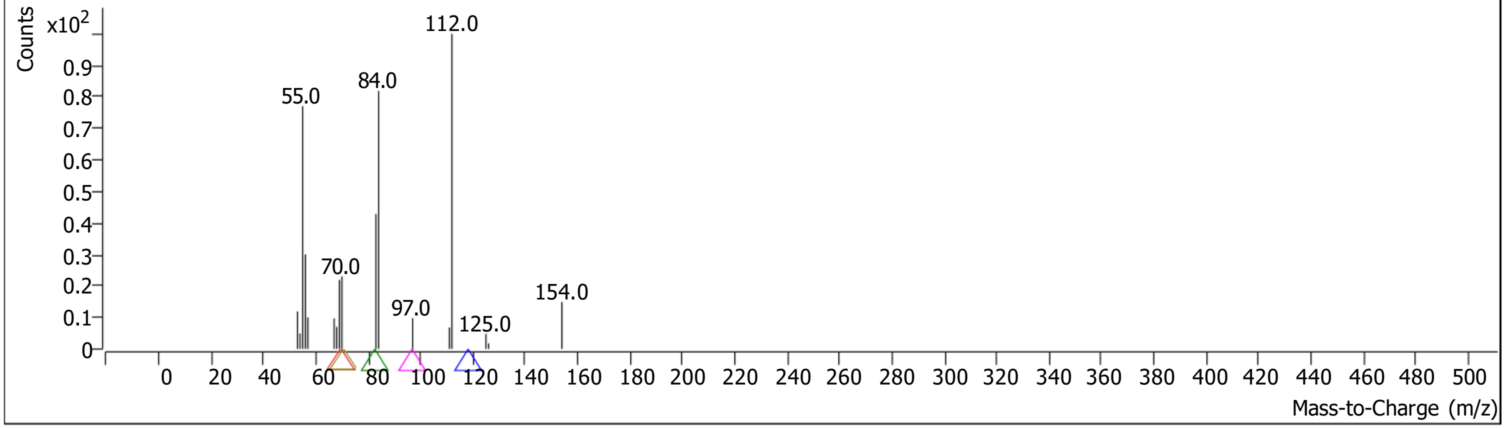


+ Scan (10.7780-10.7981 min, 11 scans) IC_E.D

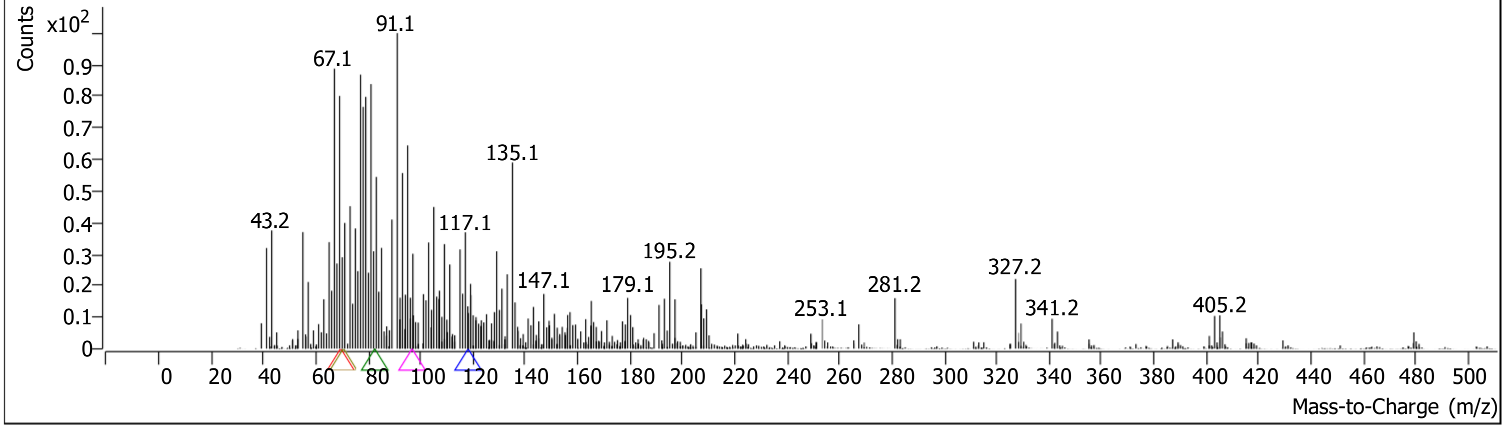


Component RT: 10.7865

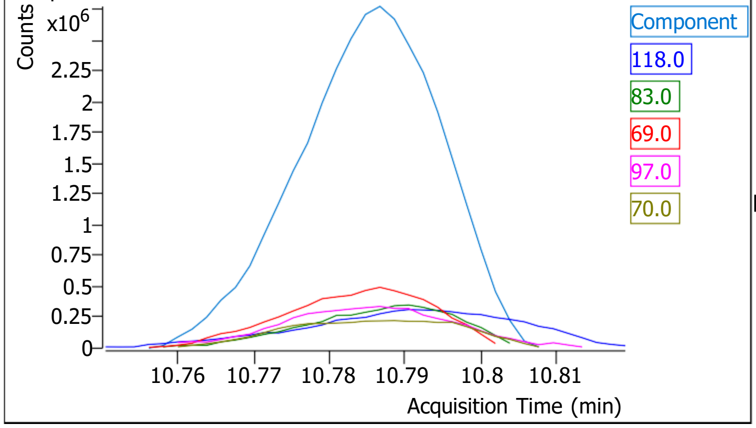


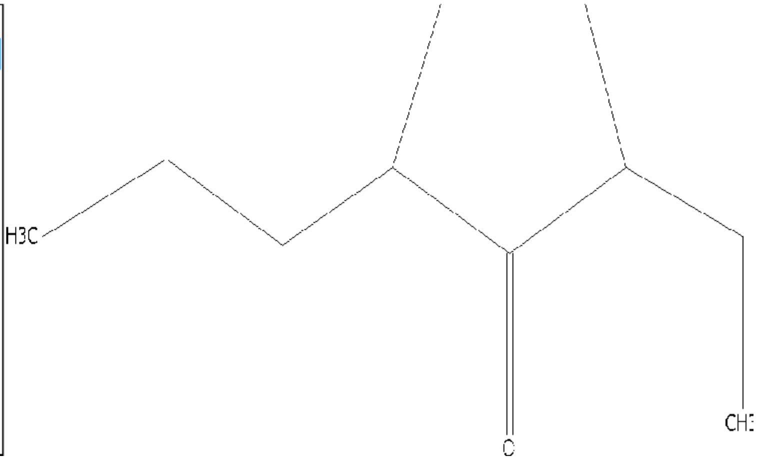

| RT | Compound Name | CAS# | Formula | Area | MI | Match Score | Sample | Sample |
|---|---|---|---|---|---|---|---|---|
| 10.8903 | 1,4-benzenediol, 2-methyl-, 4-acetate | 1000404-50-8 | C9H10O3 | 14845863 | | 66.2 | 0.21 | 0.97 |

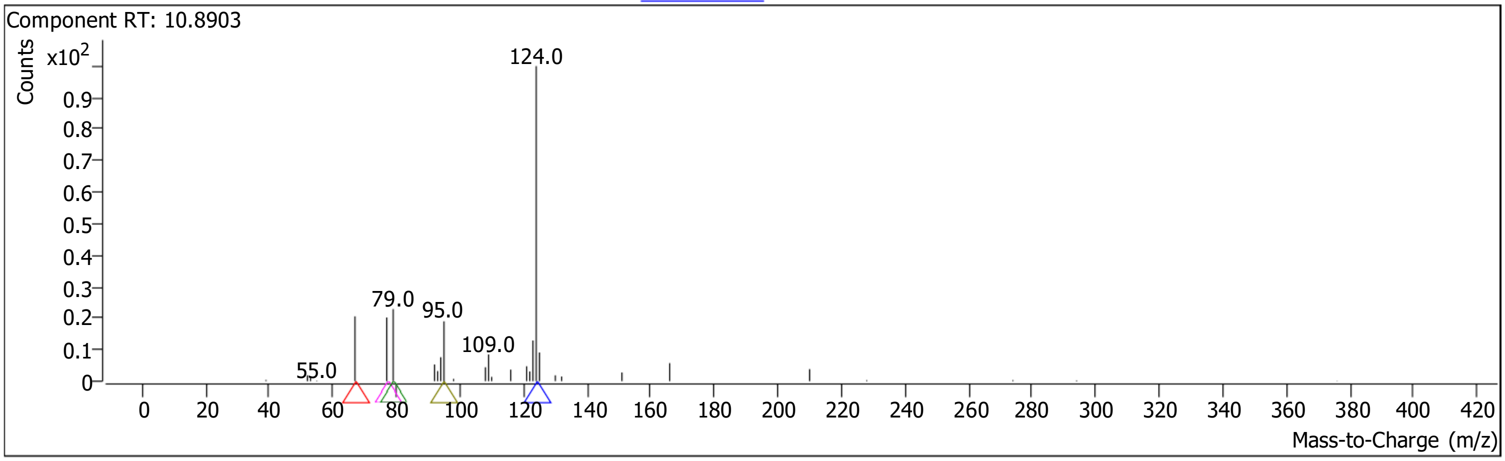


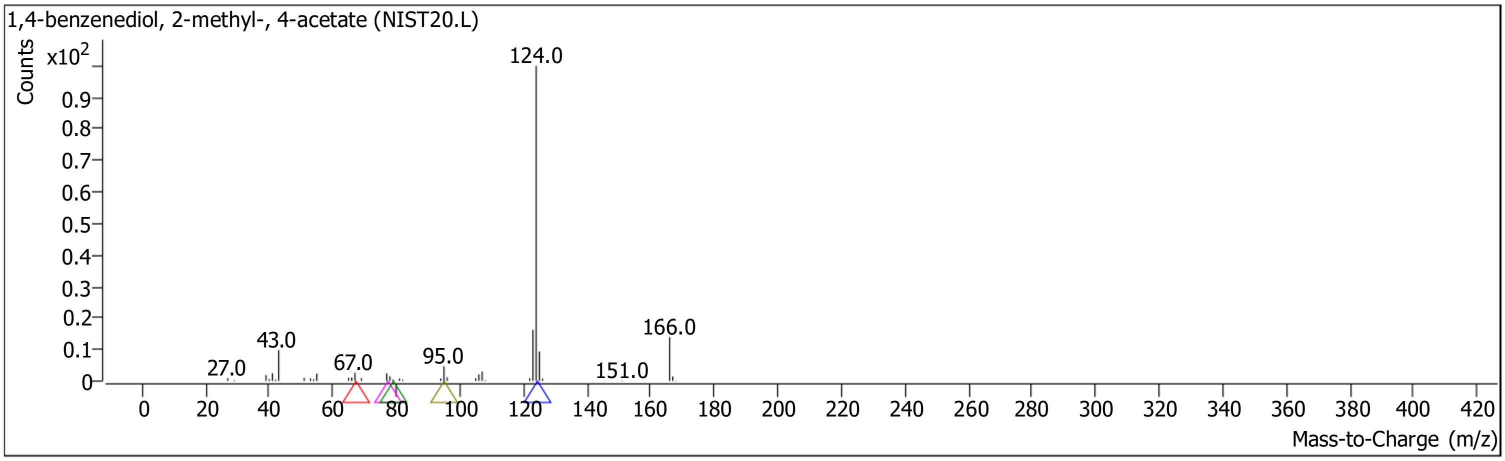


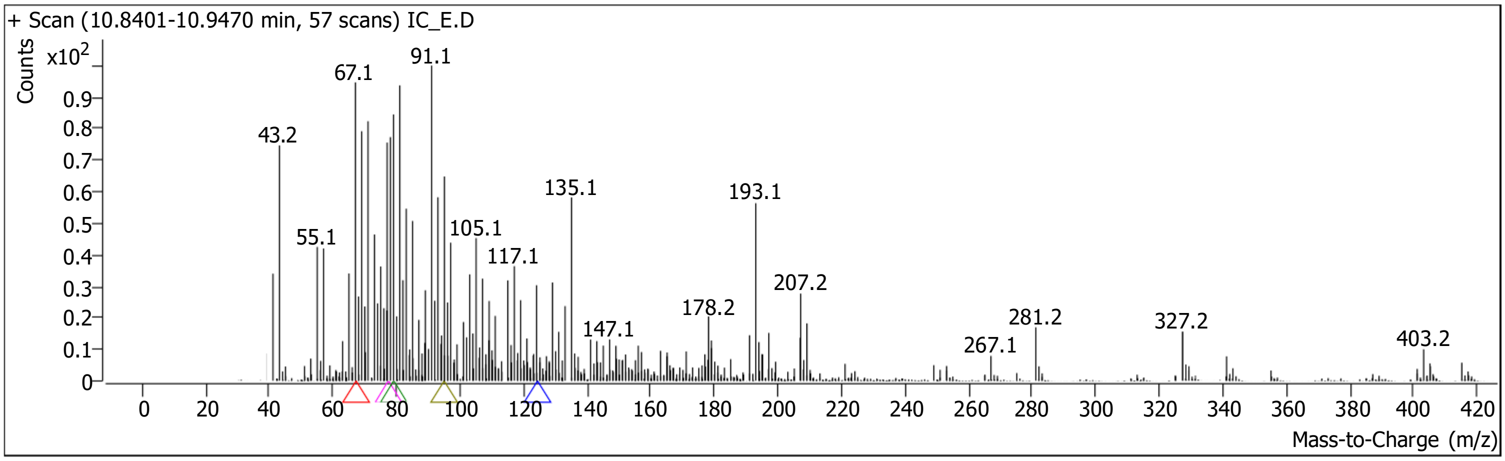


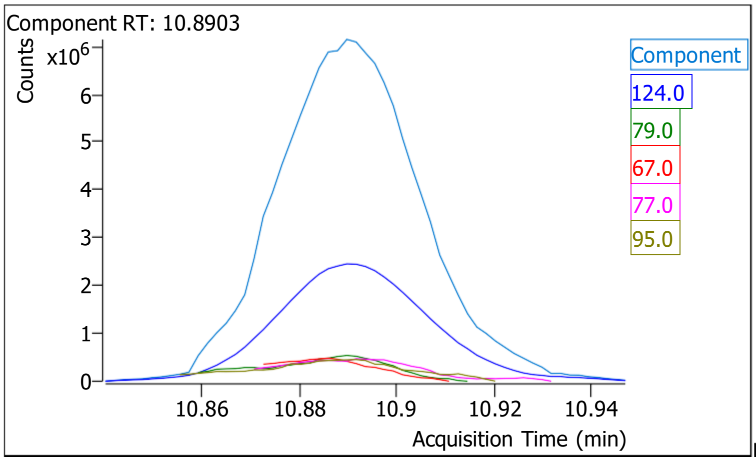


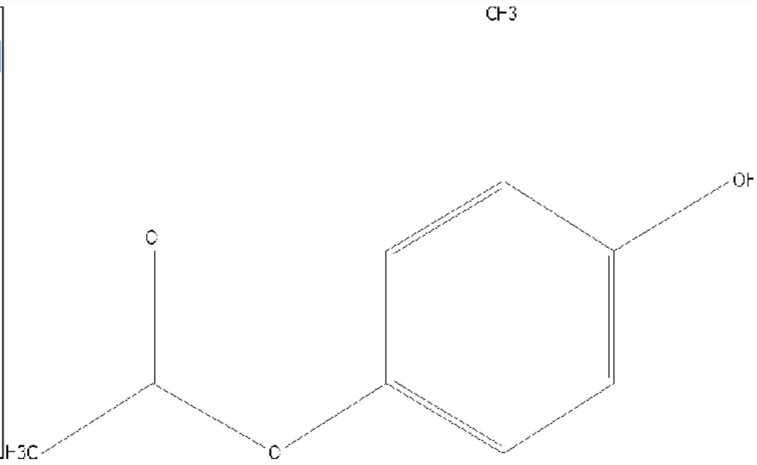

| RT | Compound Name | CAS# | Formula | Area | MI | Match Score | Sample | Sample |
|---|---|---|---|---|---|---|---|---|
| 10.9367 | 2-Cyclopropen-1-one, 2,3-diphenyl- | 886-38-4 | C15H10O | 14370581 | | 70.2 | 0.21 | 0.94 |

Component RT: 10.9367

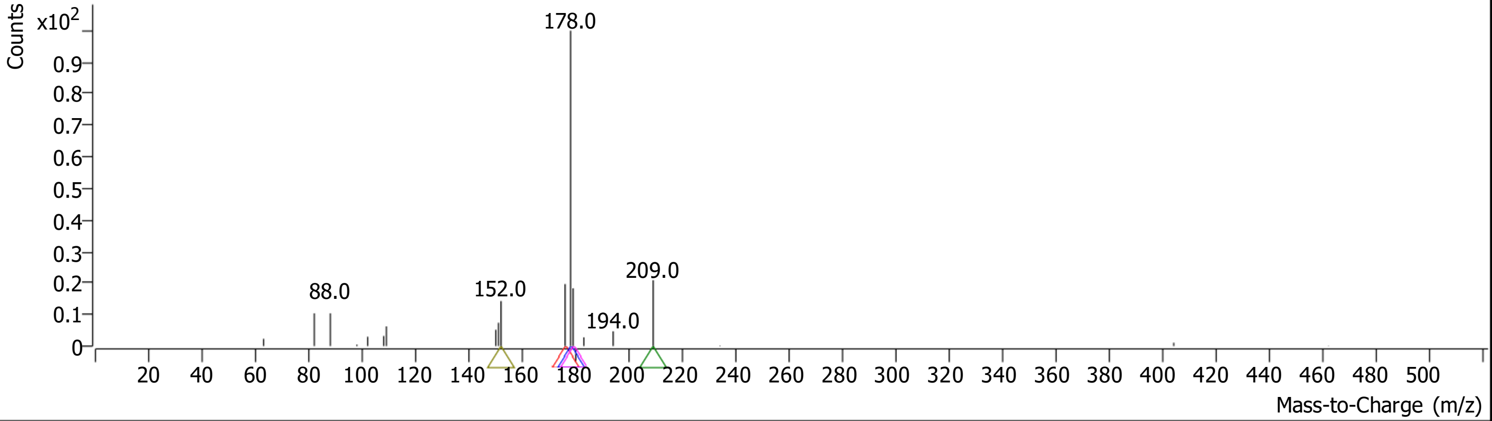


2-Cyclopropen-1-one, 2,3-diphenyl- (NIST20.L)

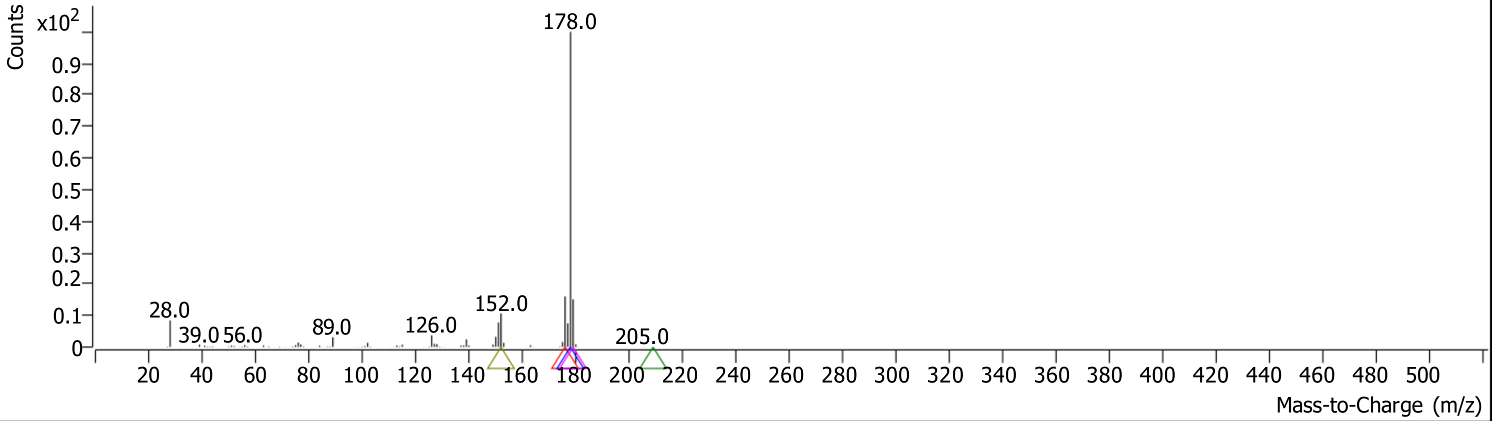


+ Scan (10.9289-10.9326 min, 2 scans) IC_E.D

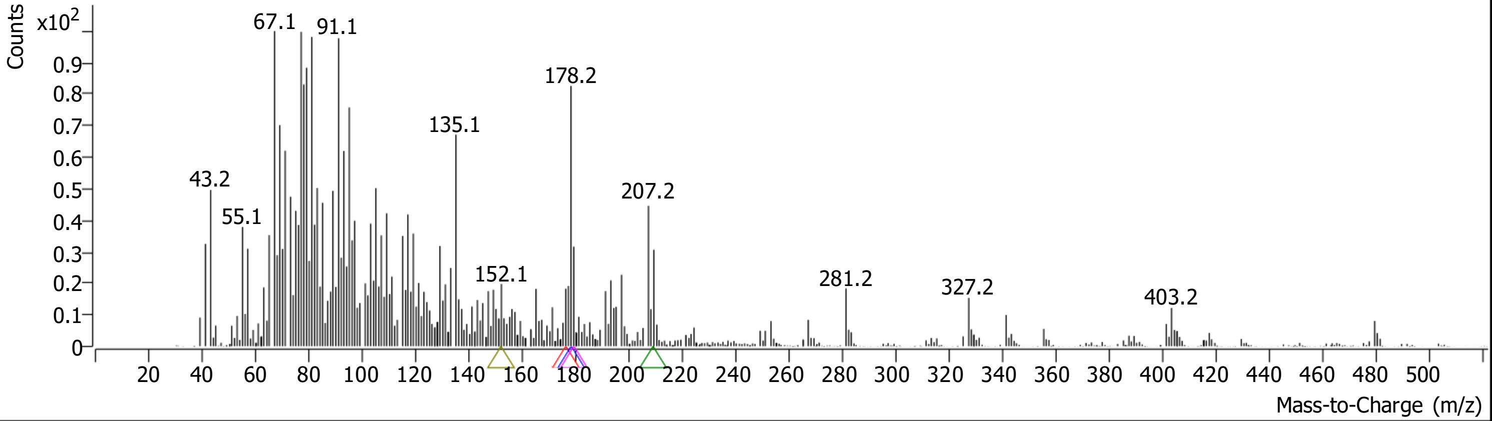


Component RT: 10.9367

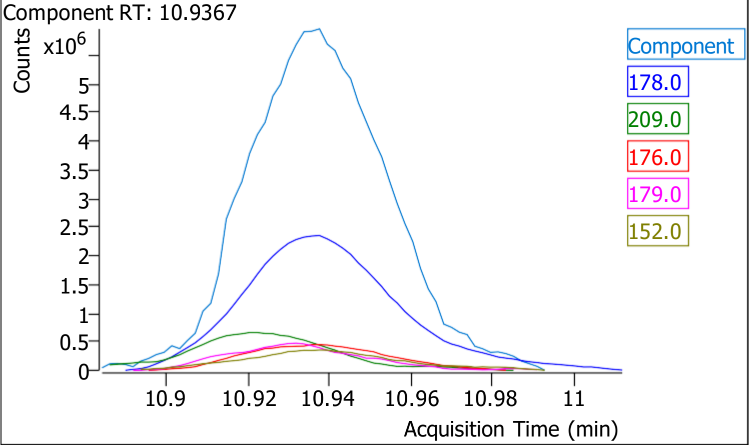


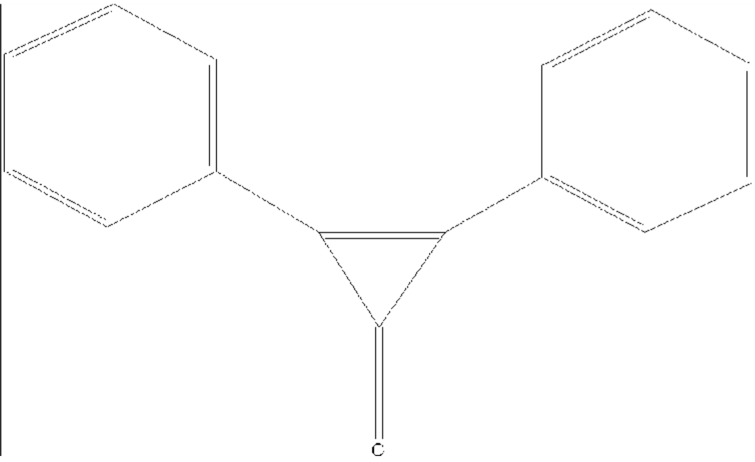

| RT | Compound Name | CAS# | Formula | Area | MI | Match Score | Sample | Sample |
|---|---|---|---|---|---|---|---|---|
| 11.2309 | 4-Methoxyphenylaldehyde trimethylene acetal | 1010429-63-9 | C11H14O3 | 28359542 | | 60.7 | 0.41 | 1.85 |

Component RT: 11.2309

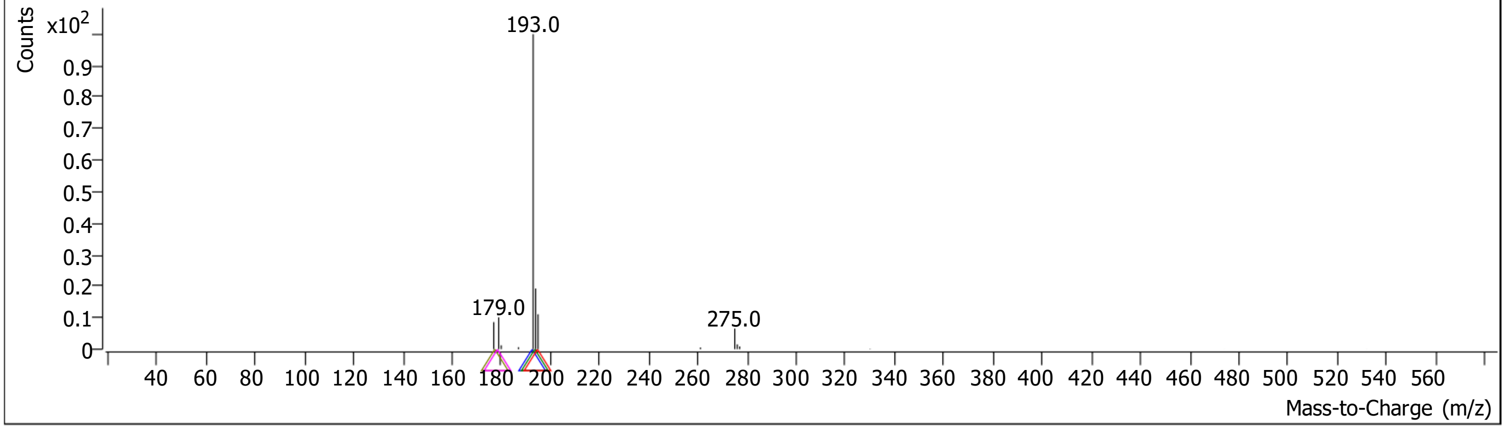


4-Methoxyphenylaldehyde trimethylene acetal (NIST20.L)

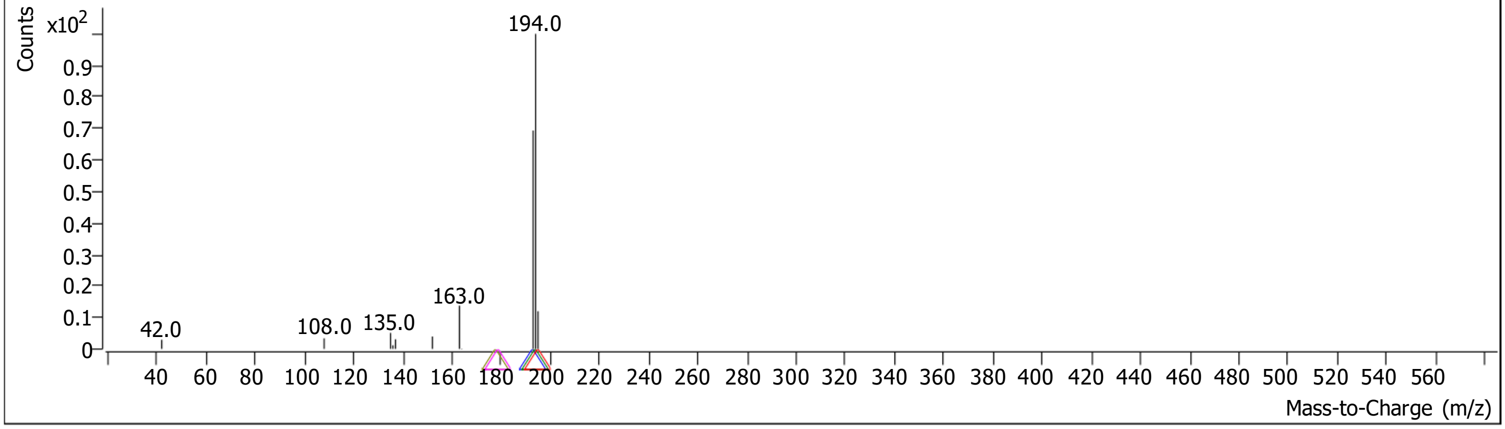


+ Scan (11.1951-11.2974 min, 54 scans) IC_E.D

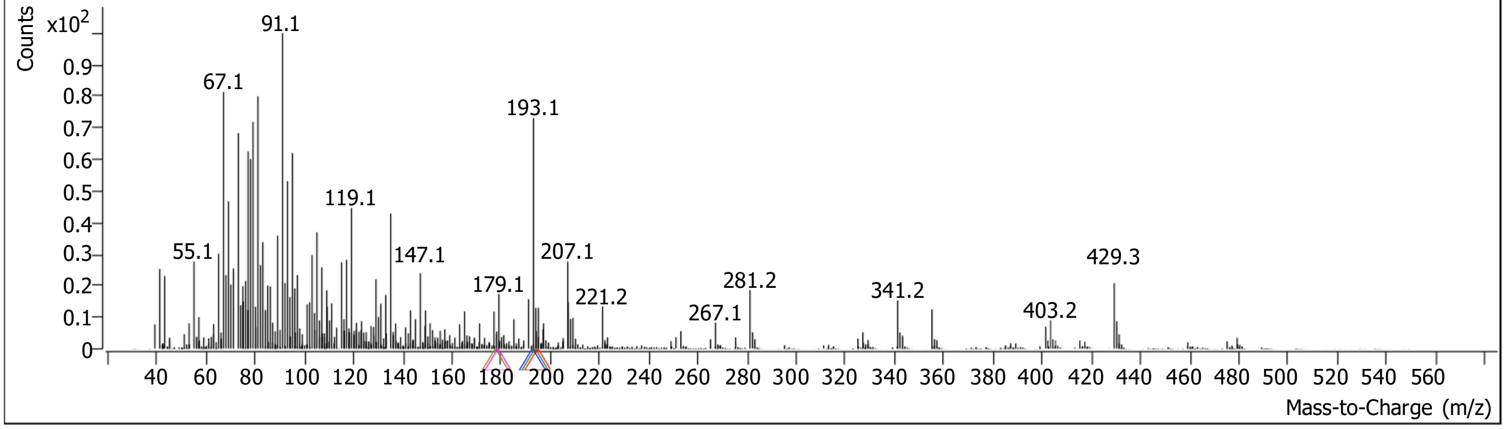


Component RT: 11.2309

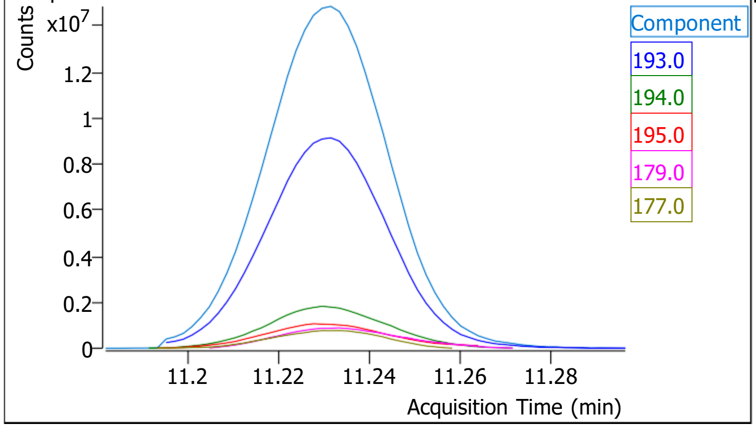


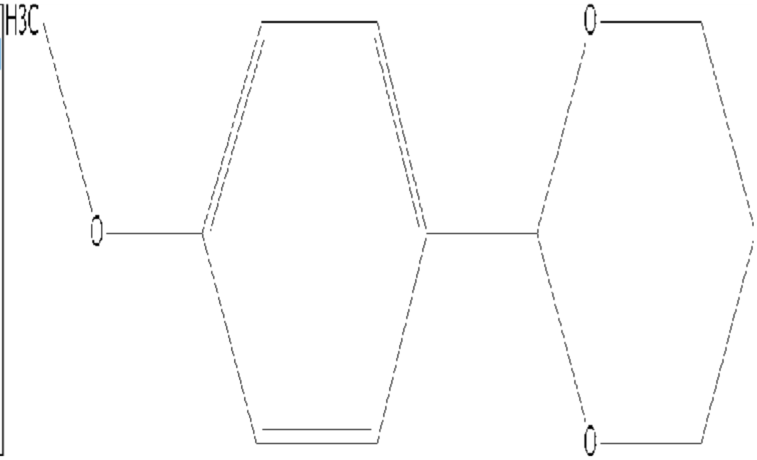

| RT | Compound Name | CAS# | Formula | Area | MI | Match Score | Sample | Sample |
|---|---|---|---|---|---|---|---|---|
| 11.2335 | s-Triazine, 2-amino-4-(piperidinomethyl)-4-piperidino- | 21868-43-9 | C14H24N6 | 36602731 | | 64.4 | 0.53 | 2.39 |

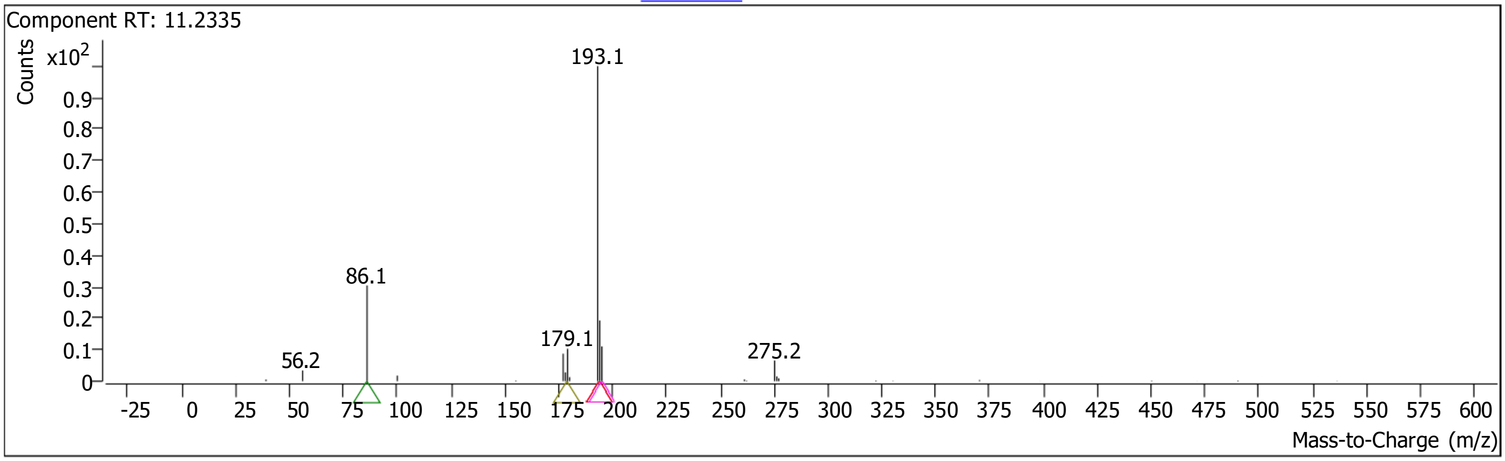


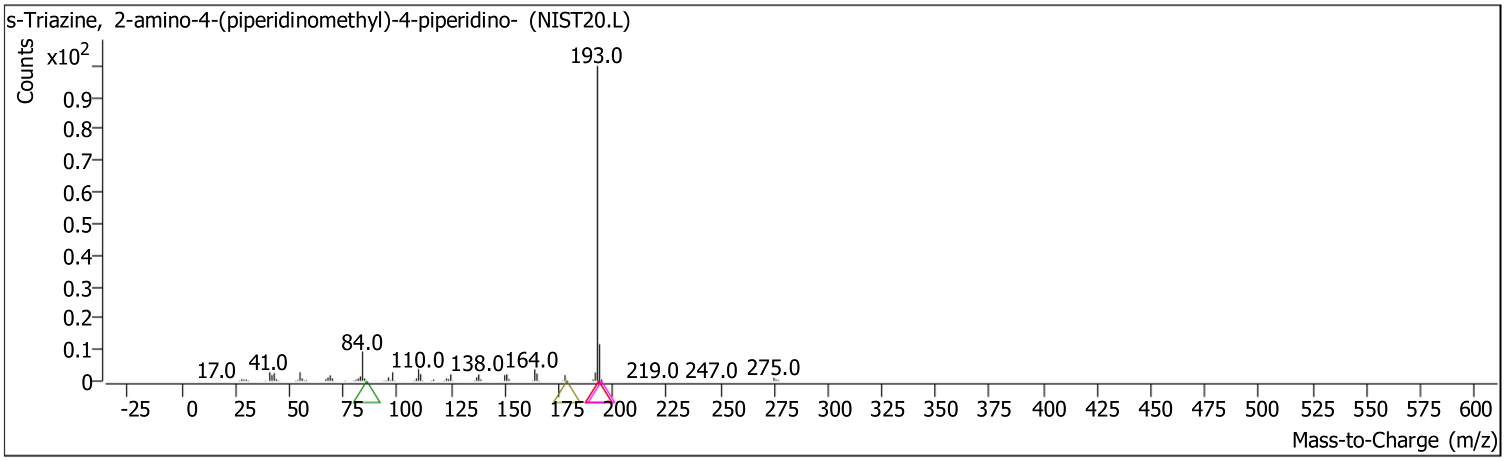


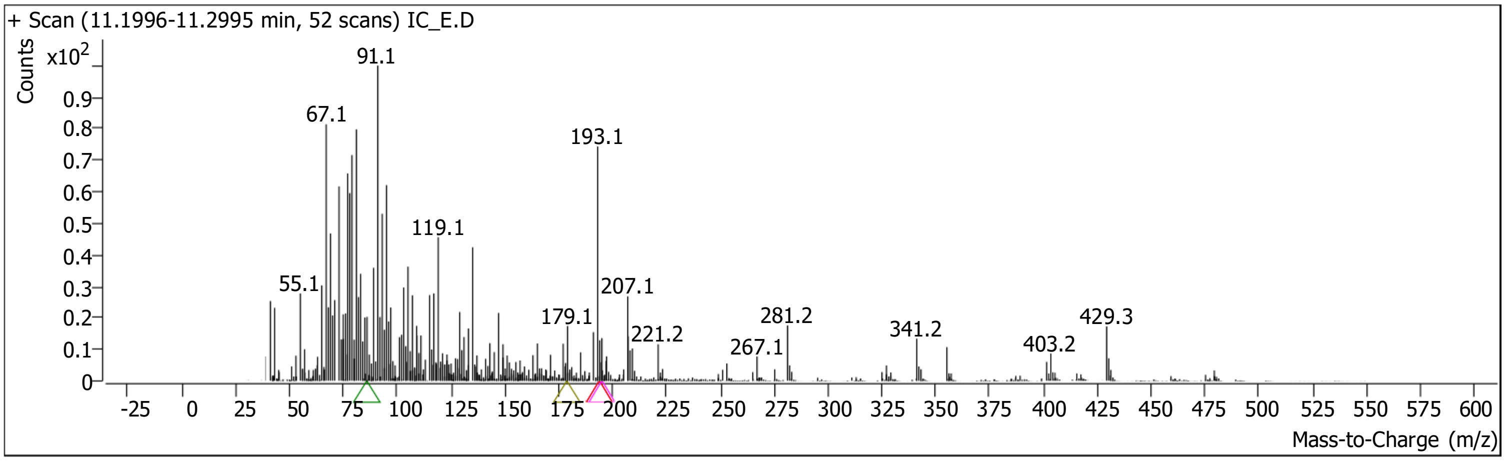


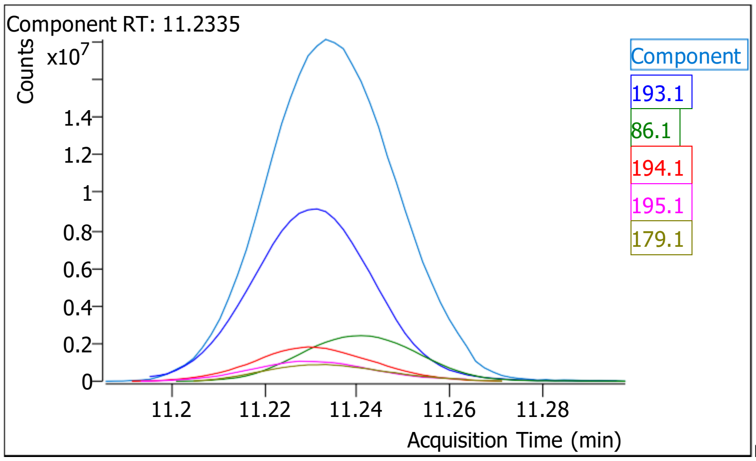


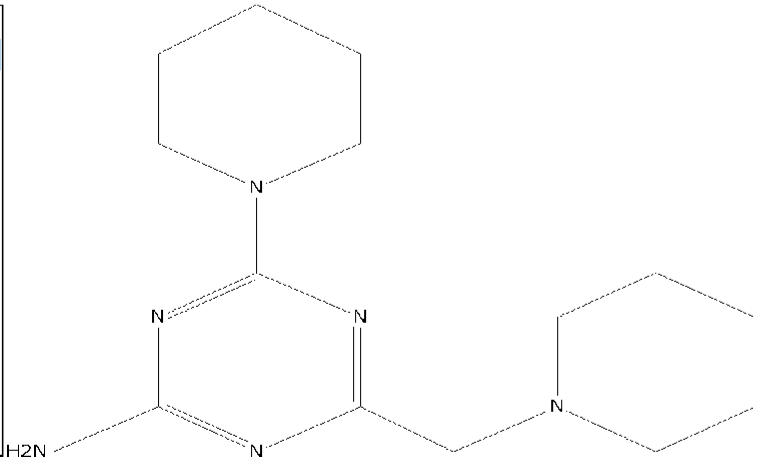


 Generated at 5:24 PM on 9/22/2025

# Unknown Analysis Report - Best Hits

| RT | Compound Name | CAS# | Formula | Area | MI | Match Score | Sample | Sample |
|---|---|---|---|---|---|---|---|---|
| 11.2905 | m-Toluic acid, 2-ethylhexyl ester | 16397-66-3 | C16H24O2 | 35764182 | | 70.9 | 0.52 | 2.34 |

Component RT: 11.2905

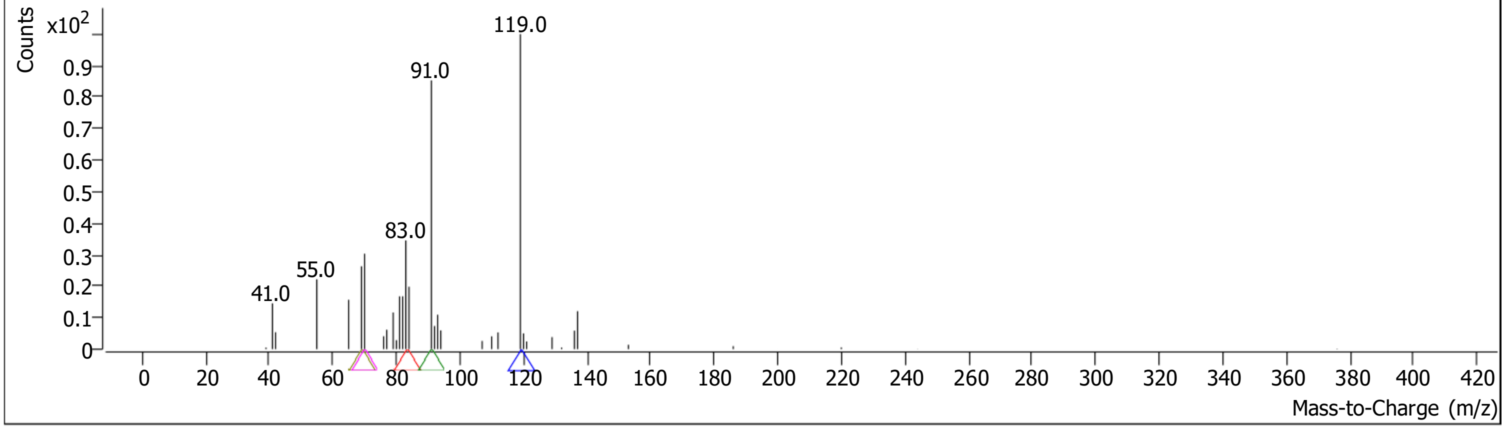


m-Toluic acid, 2-ethylhexyl ester (NIST20.L)

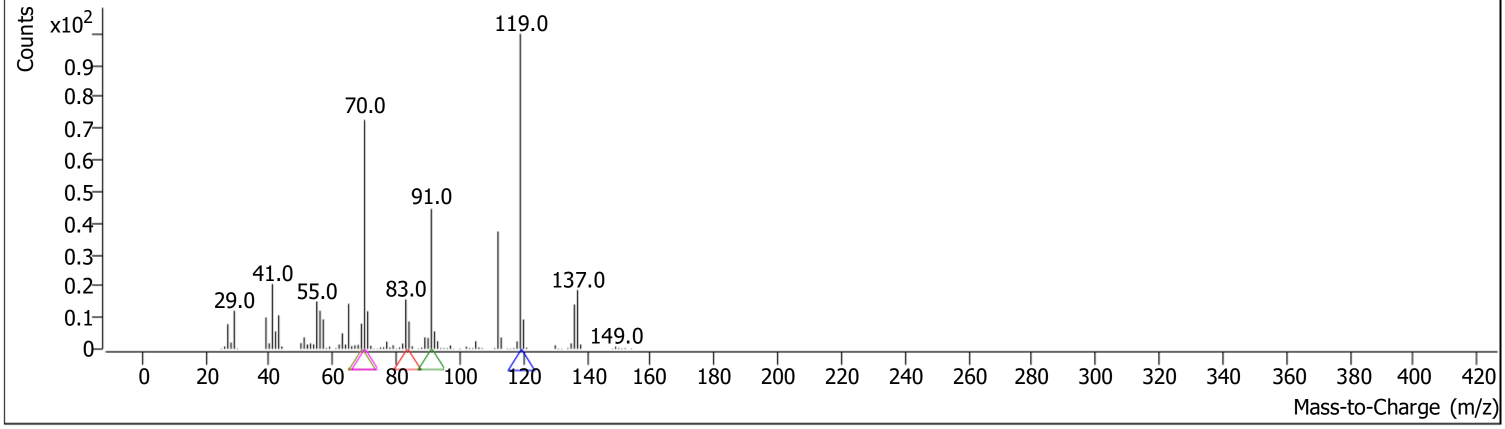


+ Scan (11.2619-11.3172 min, 29 scans) IC_E.D

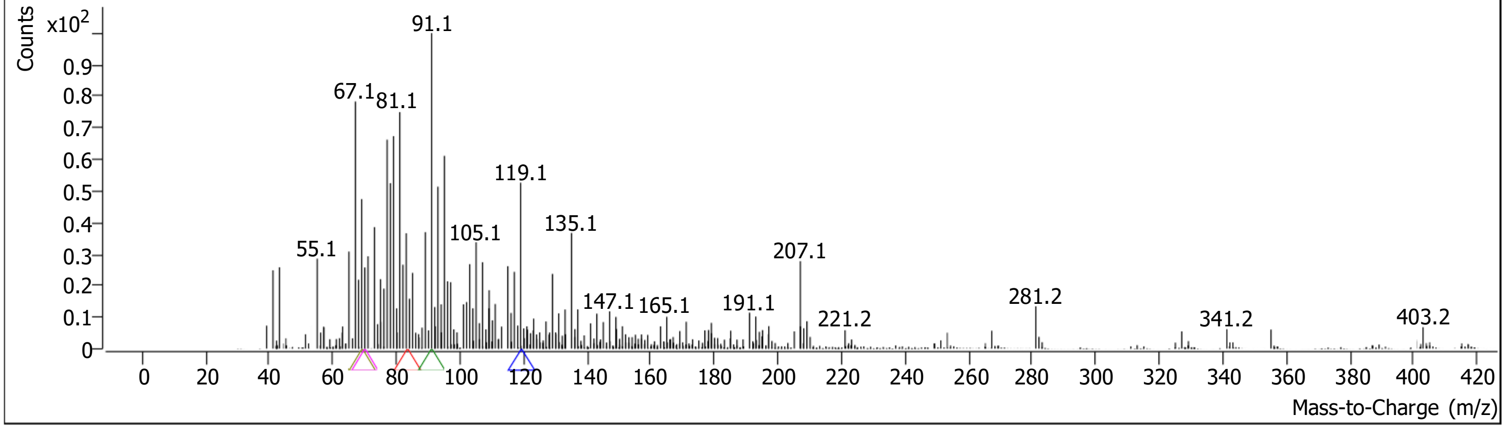


Component RT: 11.2905

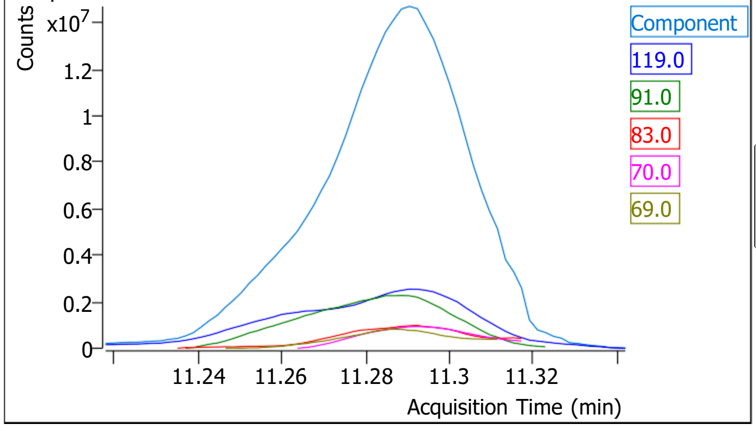


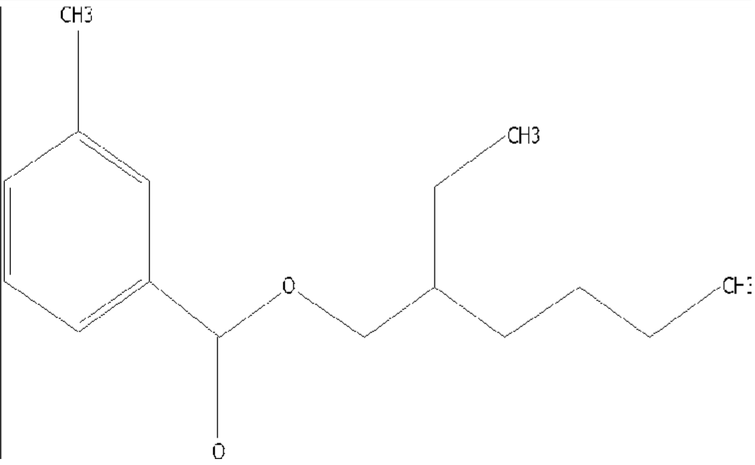

| RT | Compound Name | CAS# | Formula | Area | MI | Match Score | Sample | Sample |
|---|---|---|---|---|---|---|---|---|
| 11.3545 | Phytol | 150-86-7 | C20H40O | 278860886 | | 82.7 | 4.04 | 18.23 |

Component RT: 11.3545

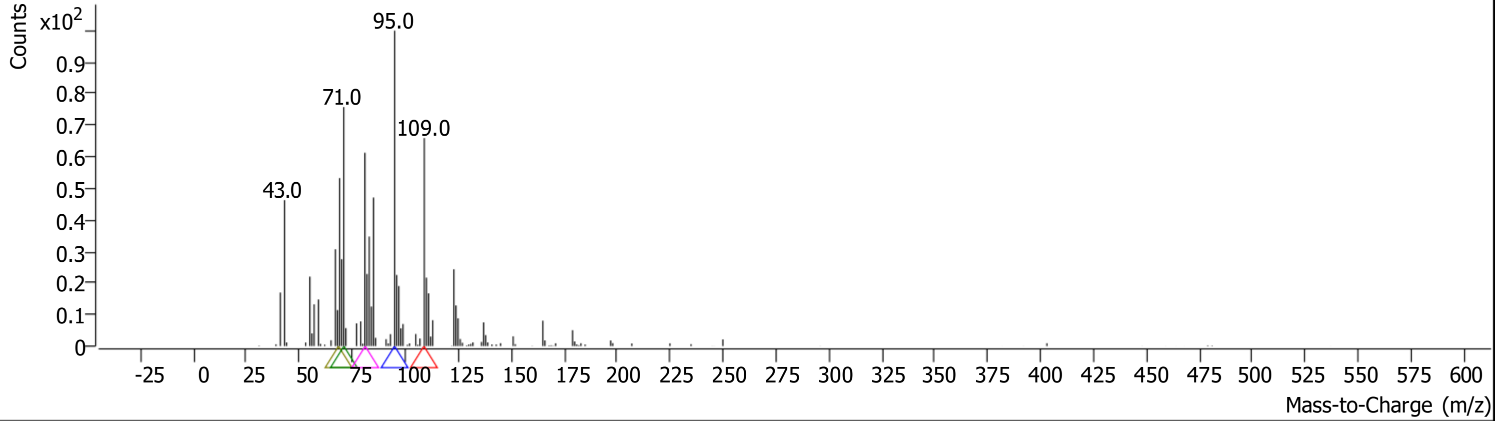


Phytol (NIST20.L)

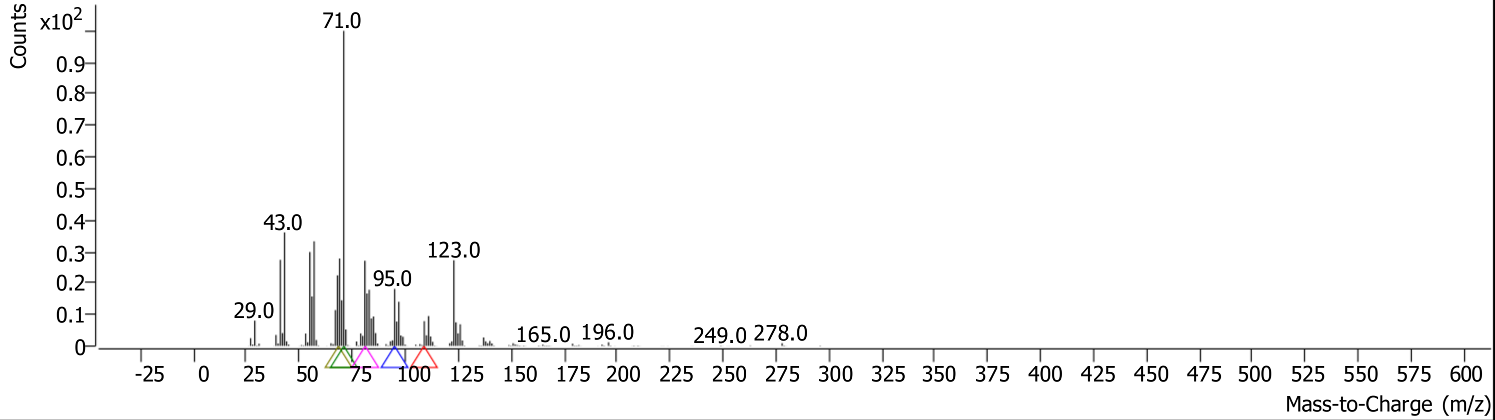


+ Scan (11.3115-11.3916 min, 43 scans) IC_E.D

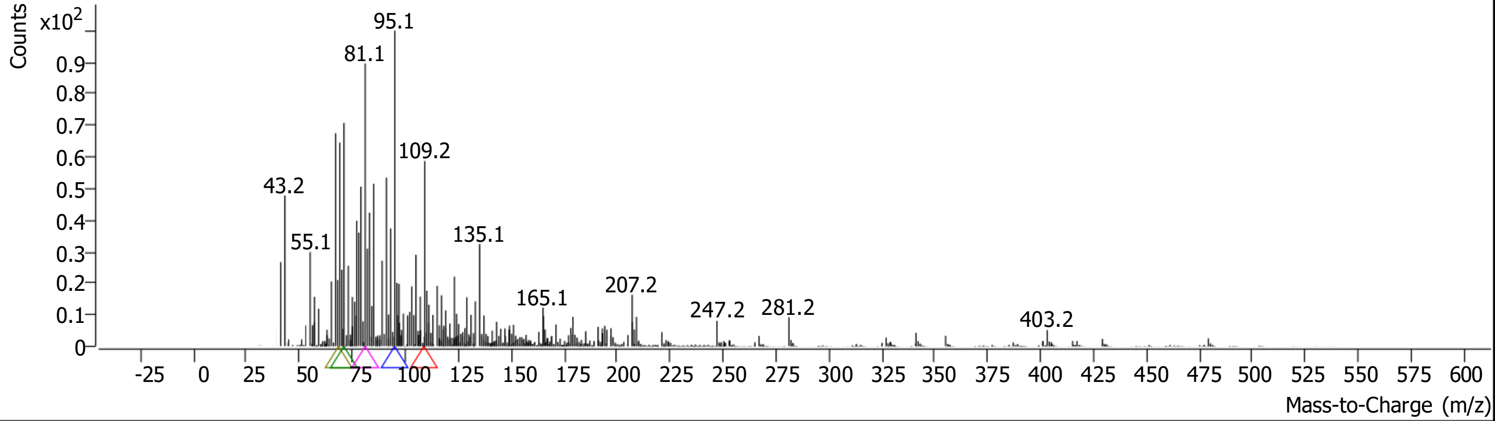


Component RT: 11.3545

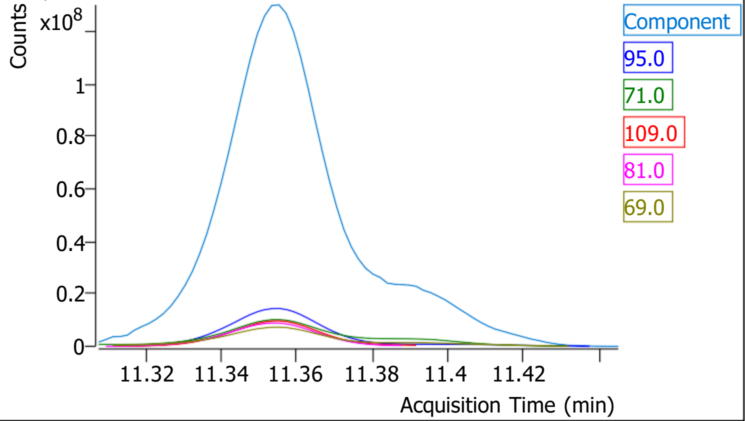


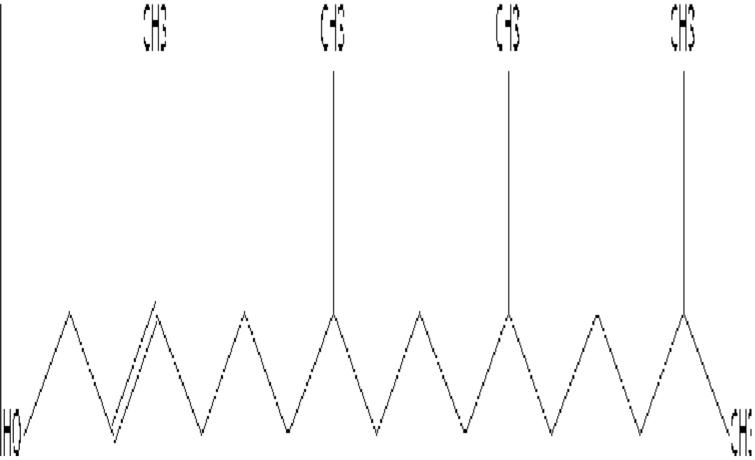

| RT | Compound Name | CAS# | Formula | Area | MI | Match Score | Sample | Sample |
|---|---|---|---|---|---|---|---|---|
| 11.5293 | Succinic acid, tridec-2-yn-1-yl tetrahydrofurfuryl ester | 1000390-72-9 | C22H36O5 | 13688696 | | 68.0 | 0.20 | 0.89 |

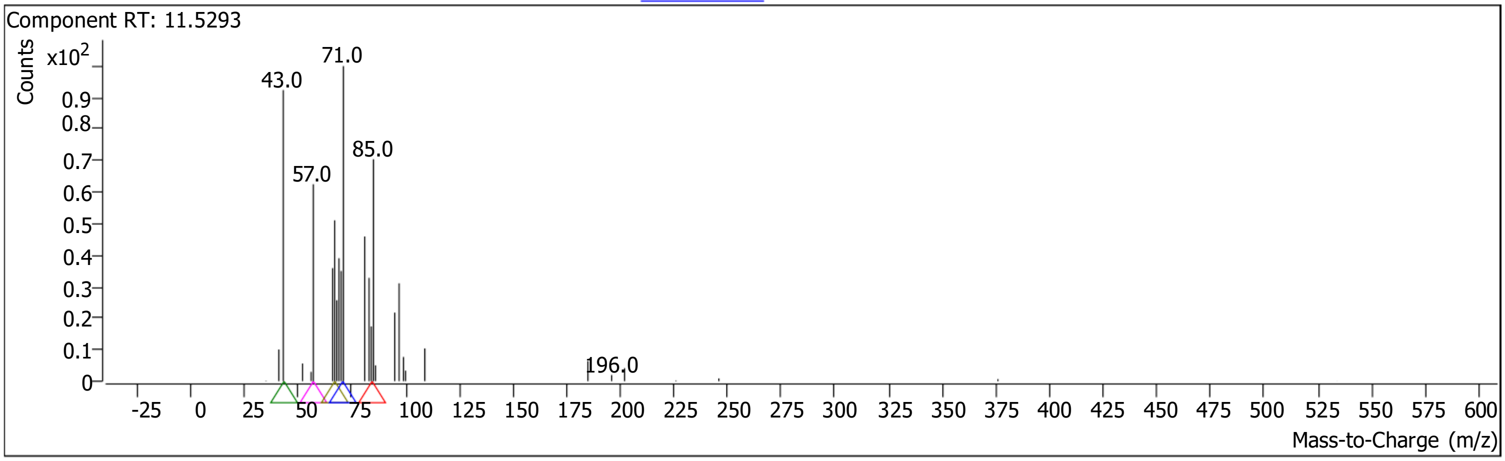


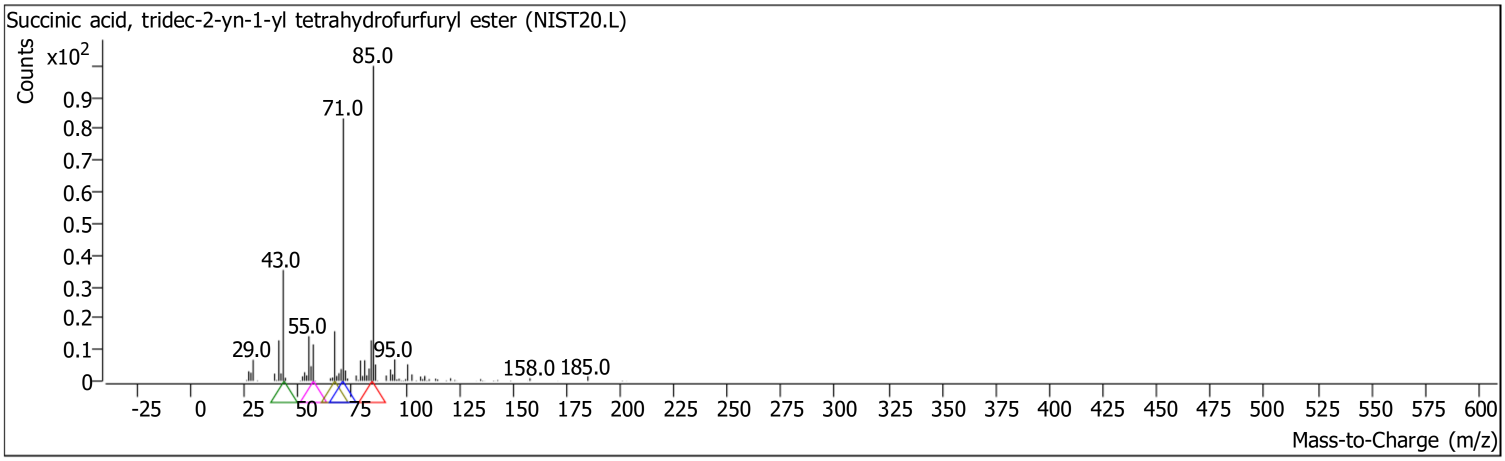


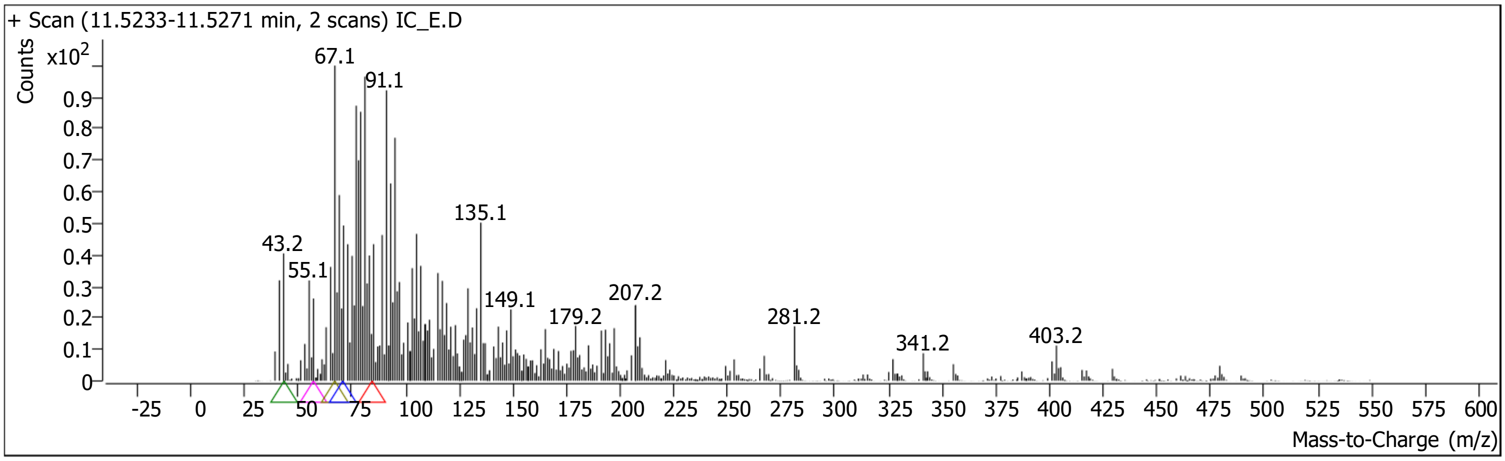


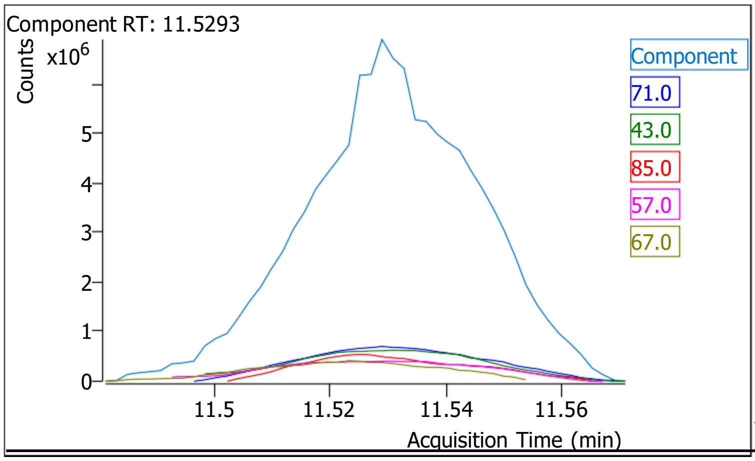


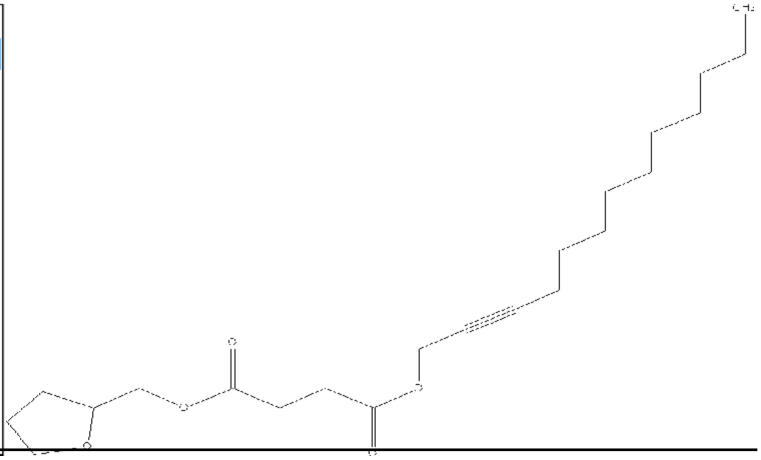

Acquisition Time (min)

 Generated at 5:24 PM on 9/22/2025

# Unknown Analysis Report - Best Hits

| RT | Compound Name | CAS# | Formula | Area | MI | Match Score | Sample | Sample |
|---|---|---|---|---|---|---|---|---|
| 11.7824 | Sulfurous acid, cyclohexylmethyl isohexyl ester | 1000309-21-5 | C13H26O3S | 20190385 | | 73.4 | 0.29 | 1.32 |

Component RT: 11.7824

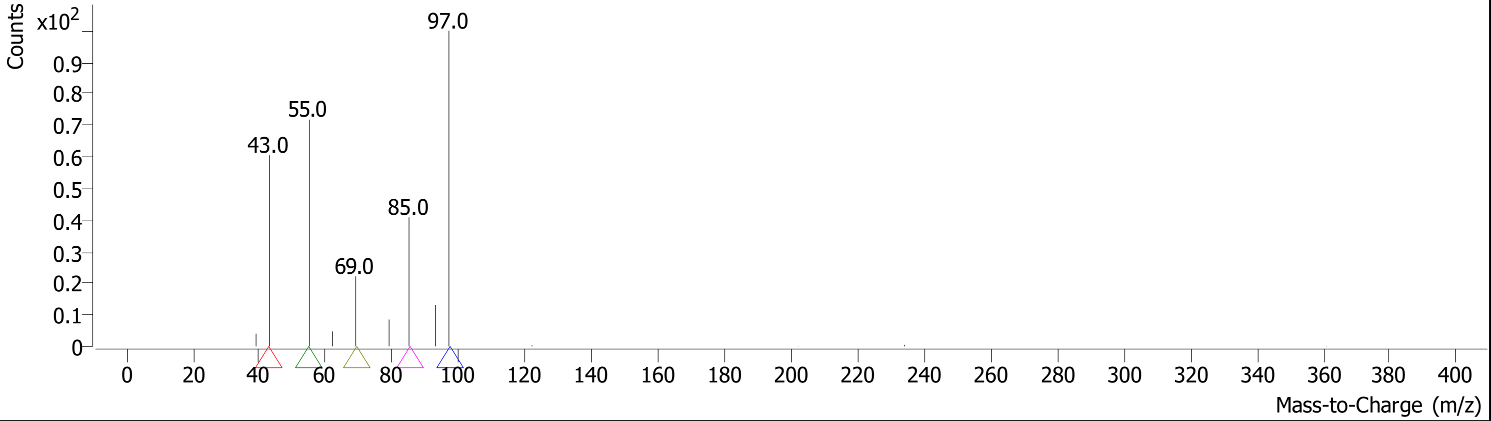


Sulfurous acid, cyclohexylmethyl isohexyl ester (NIST20.L)

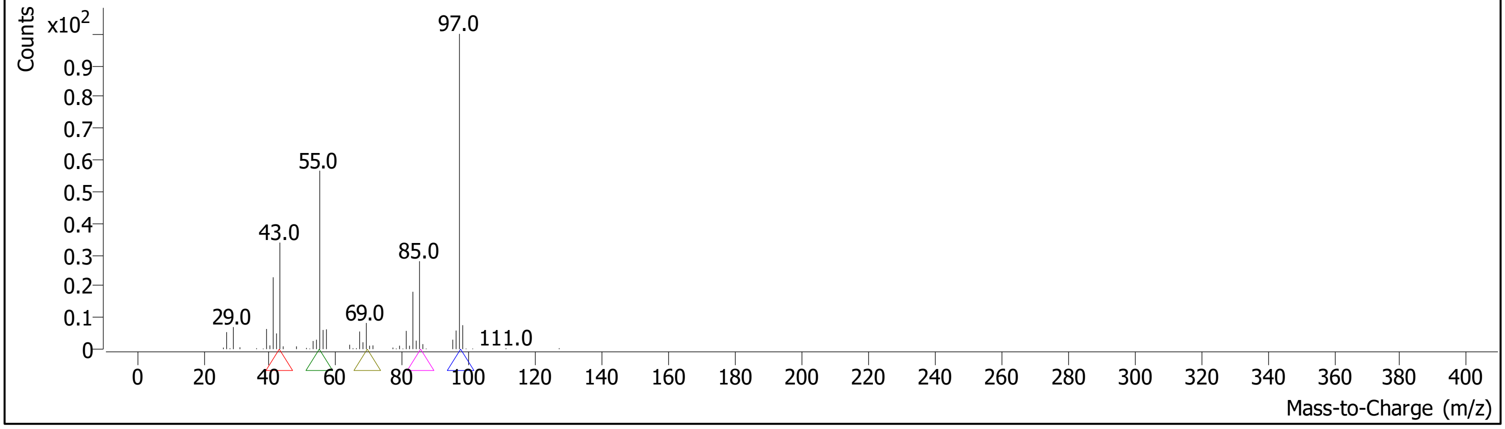


+ Scan (11.7370-11.8038 min, 36 scans) IC_E.D

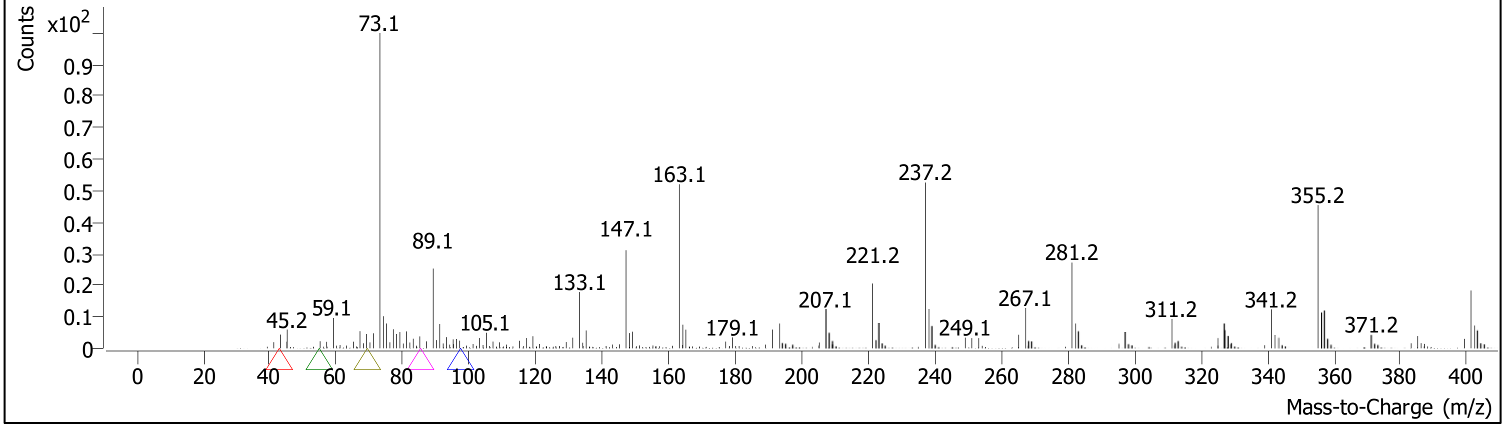


Component RT: 11.7824

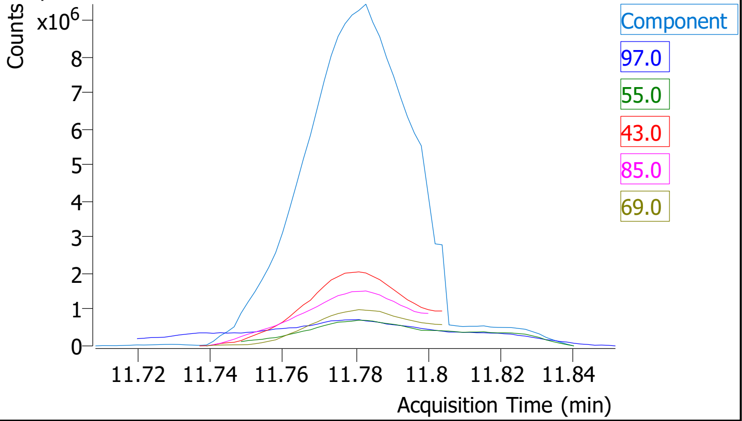


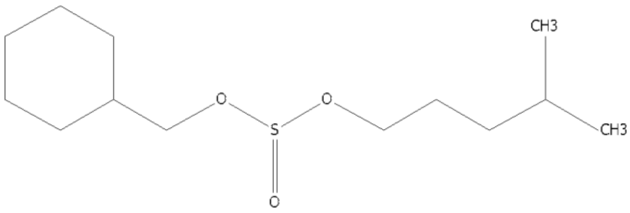


 Generated at 5:24 PM on 9/22/2025

| RT | Compound Name | CAS# | Formula | Area | MI | Match Score | Sample | Sample |
|---|---|---|---|---|---|---|---|---|
| 12.2051 | 7,9-Di-tert-butyl-1-oxaspiro(4,5)deca-6,9-diene-2,8-dione | 82304-66-3 | C17H24O3 | 140017492 | | 79.7 | 2.03 | 9.15 |

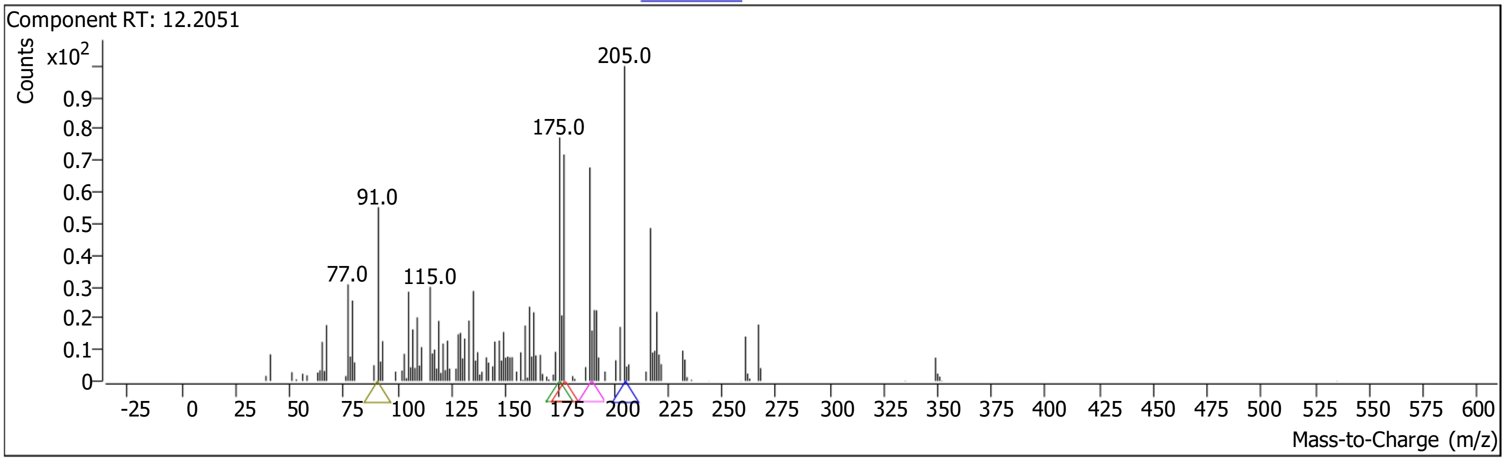


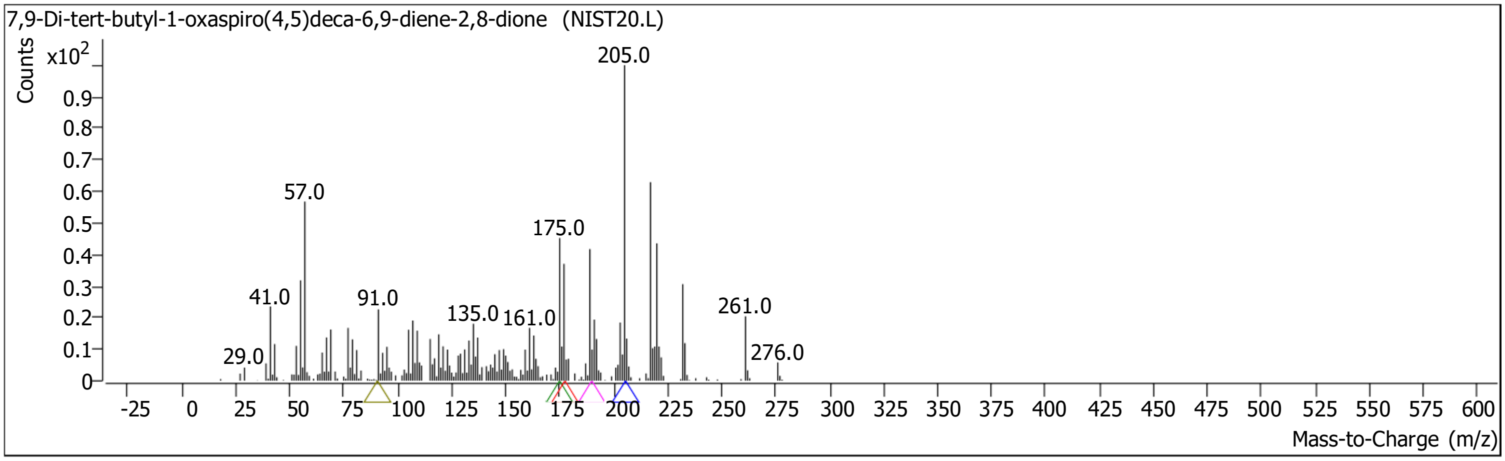


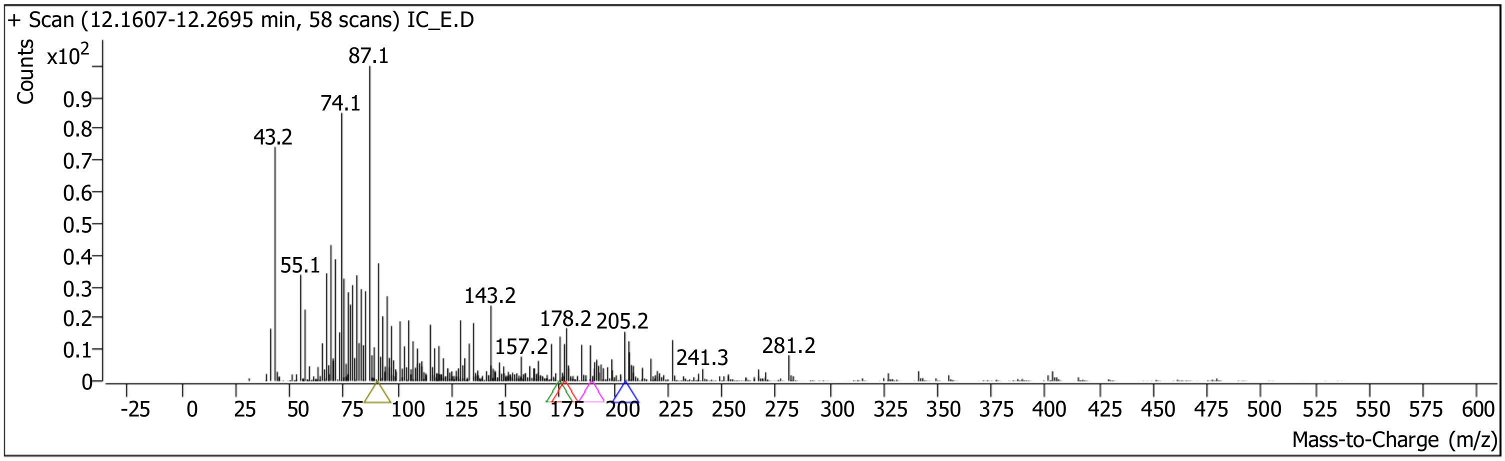


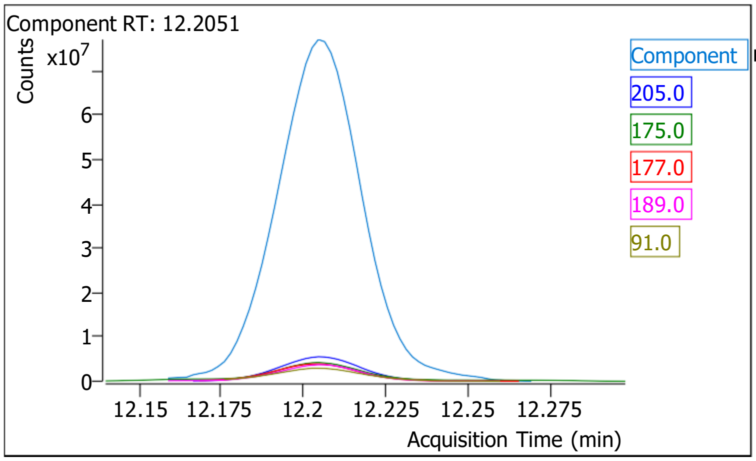


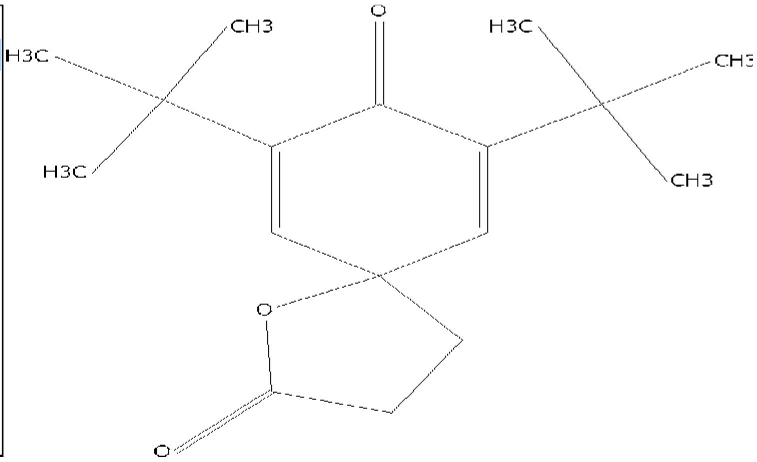



Generated at 5:24 PM on 9/22/2025

# Unknown Analysis Report - Best Hits

| RT | Compound Name | CAS# | Formula | Area | MI | Match Score | Sample | Sample |
|---|---|---|---|---|---|---|---|---|
| 12.3993 | Benzenepropanoic acid, 3,5-bis(1,1-dimethylethyl)-4-hydroxy-, methyl ester | 6386-38-5 | C18H28O3 | 5597808 | | 65.4 | 0.08 | 0.37 |

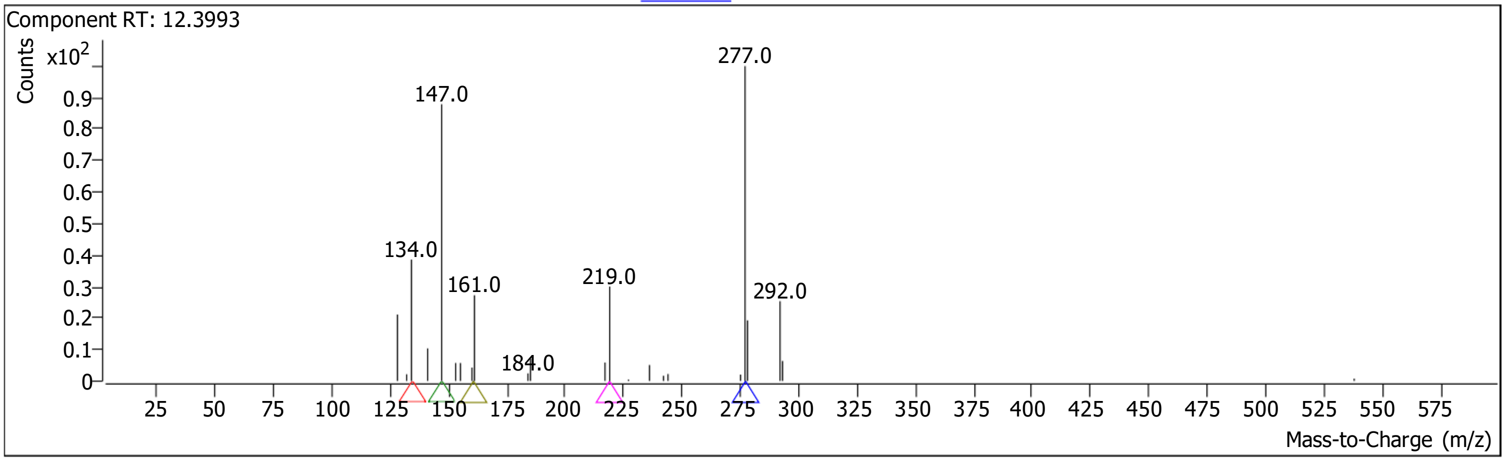


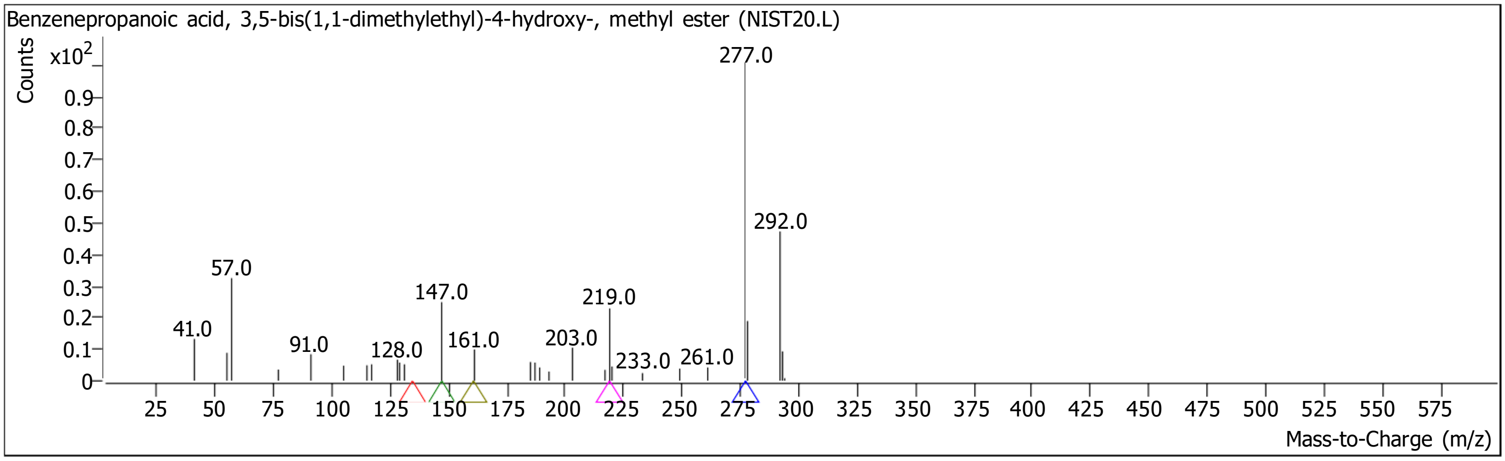


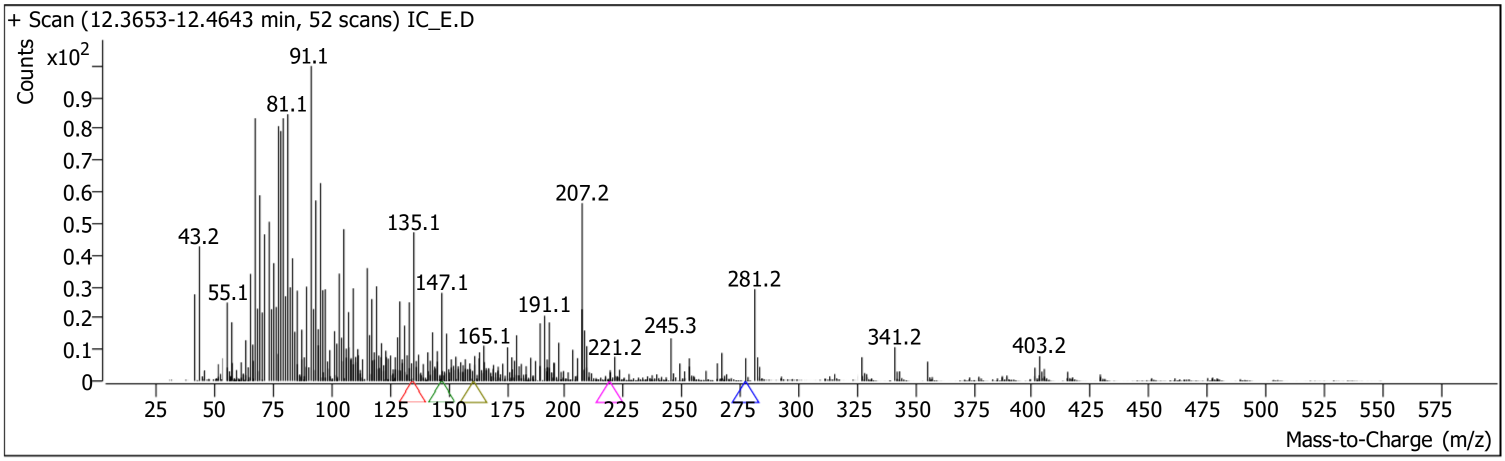


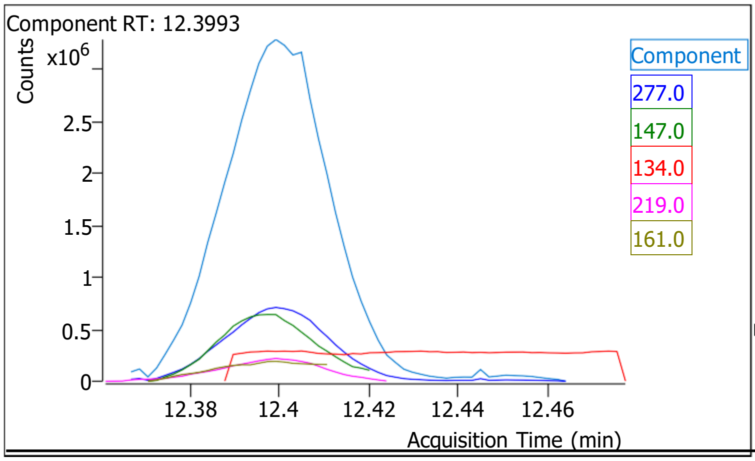


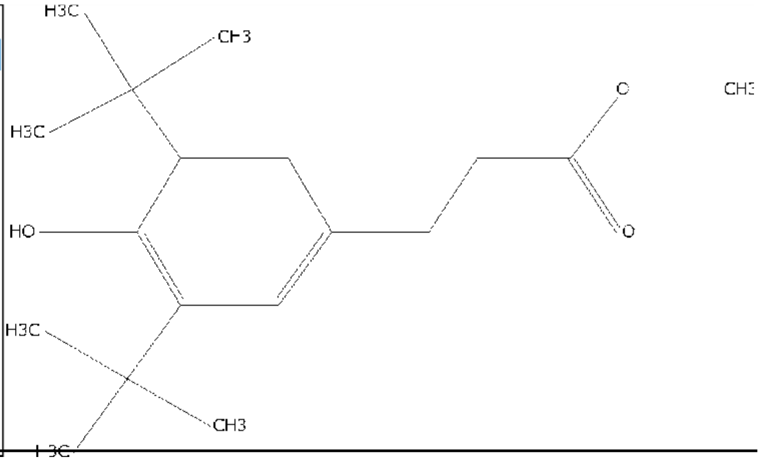


Acquisition Time (min)

| RT | Compound Name | CAS# | Formula | Area | MI | Match Score | Sample | Sample |
|---|---|---|---|---|---|---|---|---|
| 12.5257 | Pentanedioic acid, 2-oxo-, dimethyl ester | 13192-04-6 | C7H10O5 | 15447030 | | 63.4 | 0.22 | 1.01 |

Component RT: 12.5257

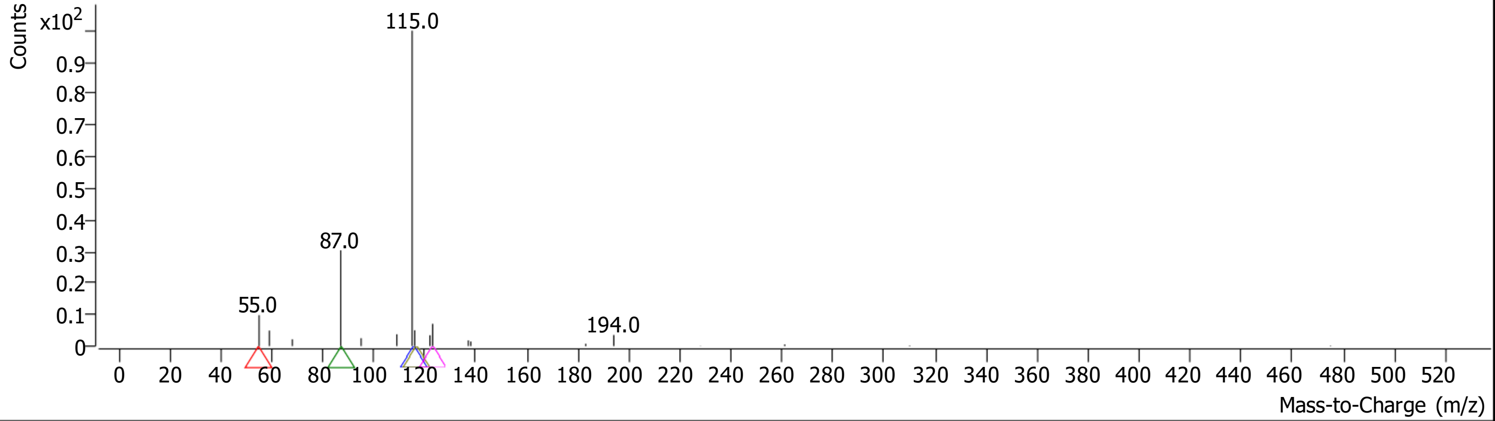


Pentanedioic acid, 2-oxo-, dimethyl ester (NIST20.L)

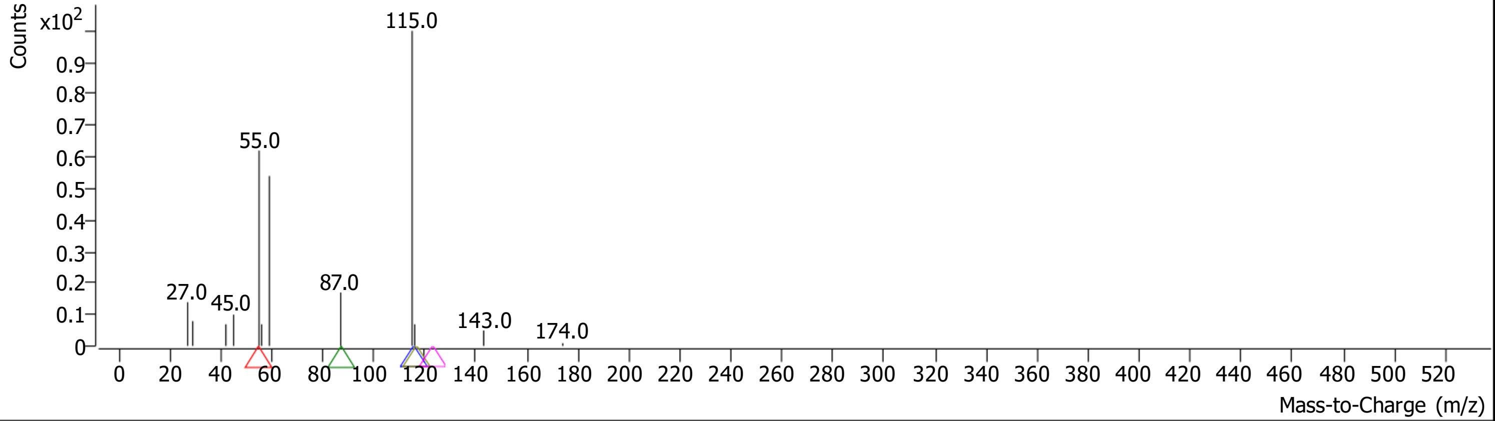


+ Scan (12.4900-12.5682 min, 41 scans) IC_E.D

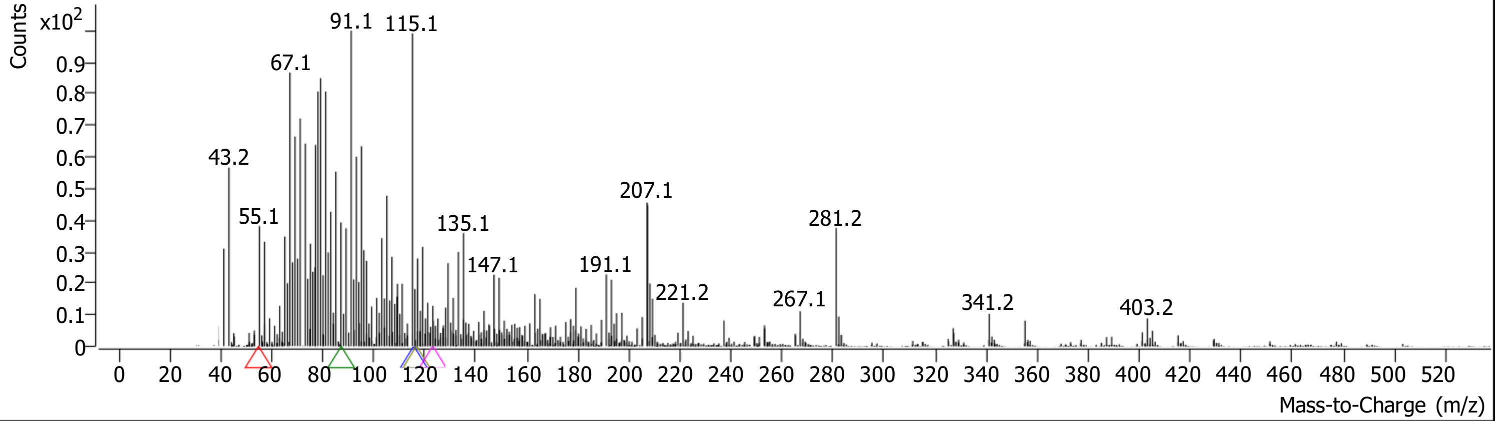


Component RT: 12.5257

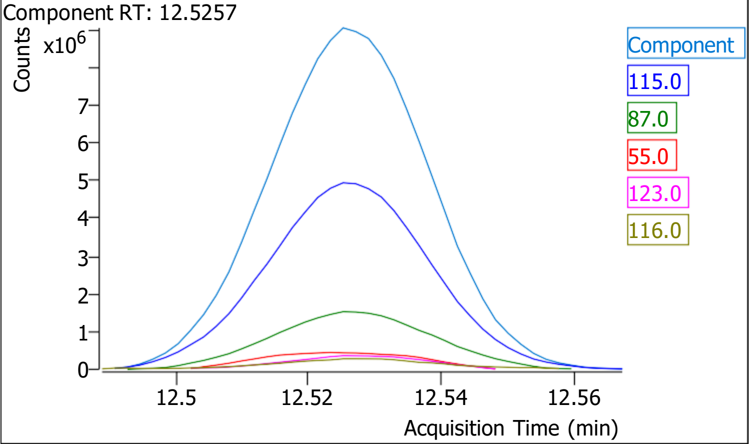


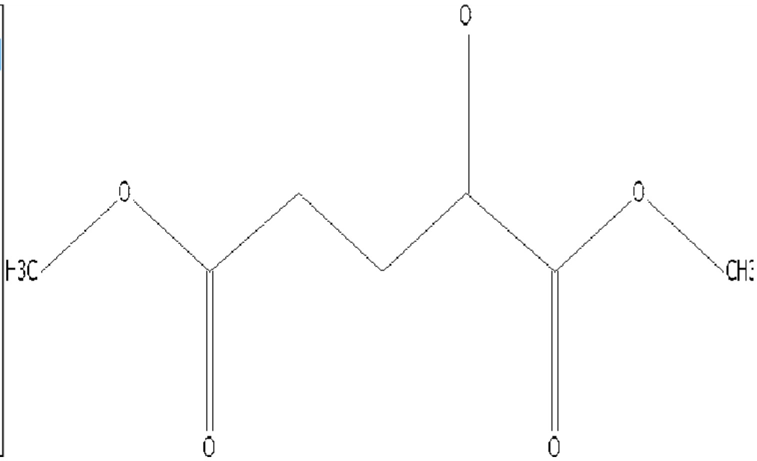

| RT | Compound Name | CAS# | Formula | Area | MI | Match Score | Sample | Sample |
|---|---|---|---|---|---|---|---|---|
| 13.7925 | 1,8,11-Heptadecatriene, (Z,Z)- | 56134-03-3 | C17H30 | 23339813 | | 74.2 | 0.34 | 1.53 |

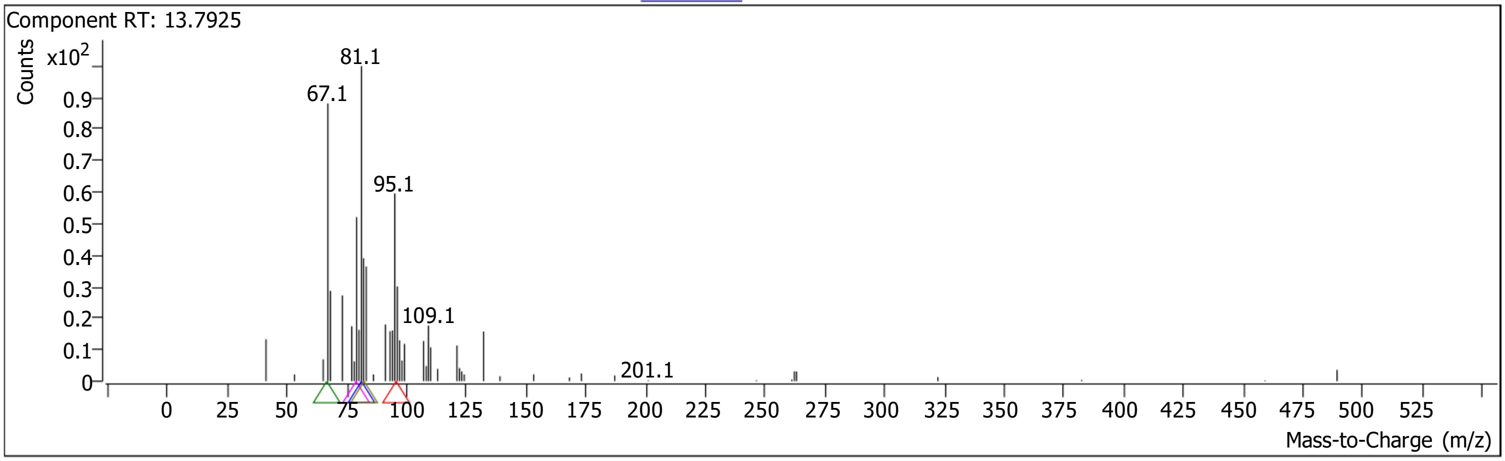


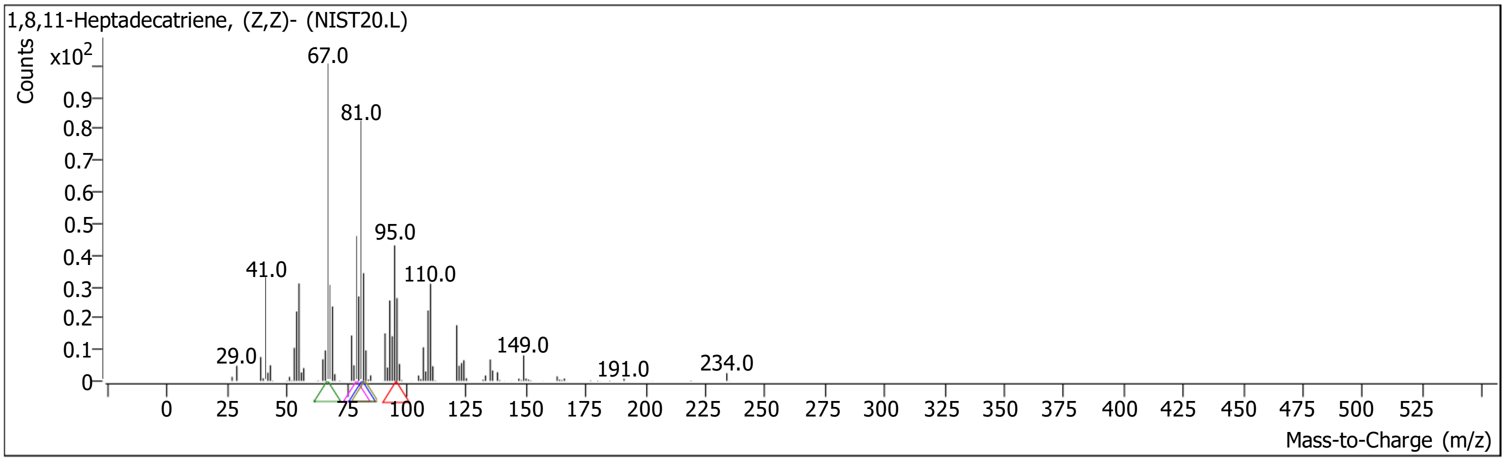


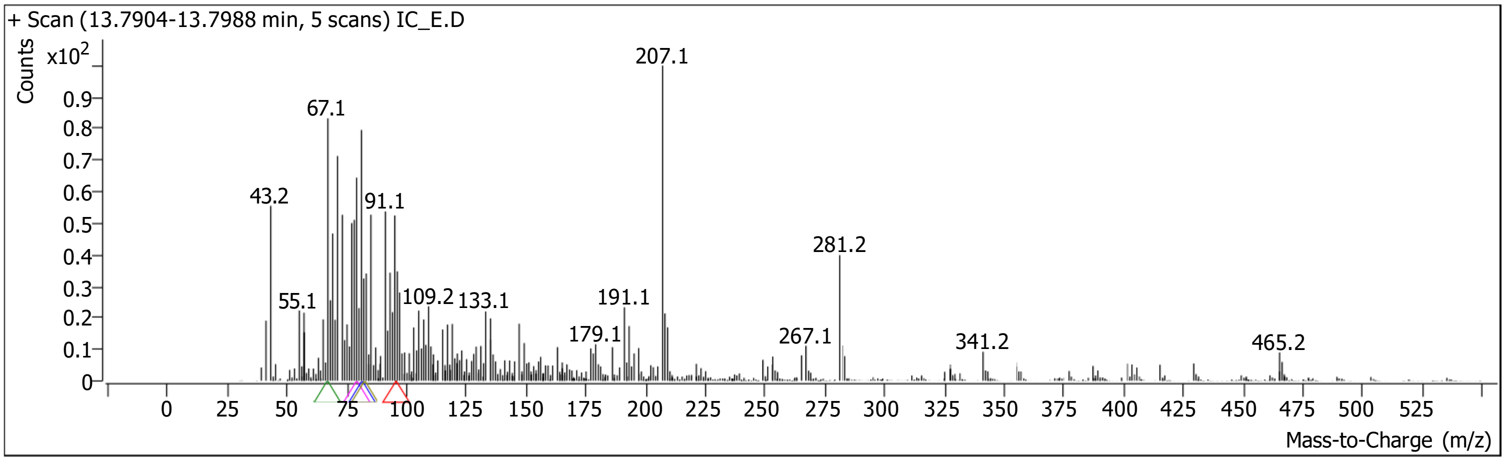


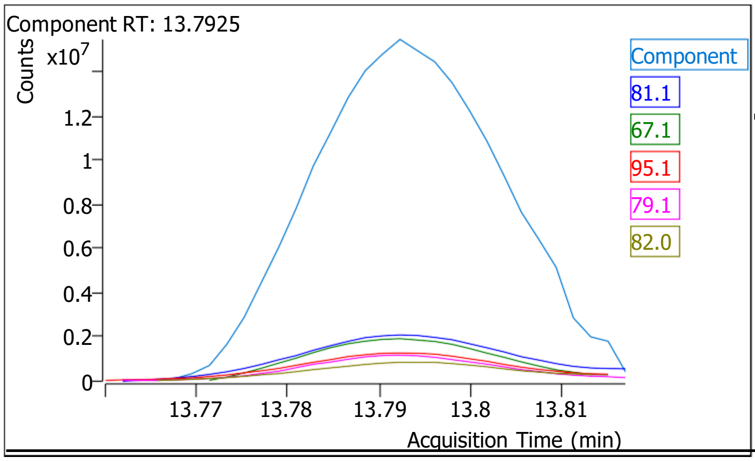


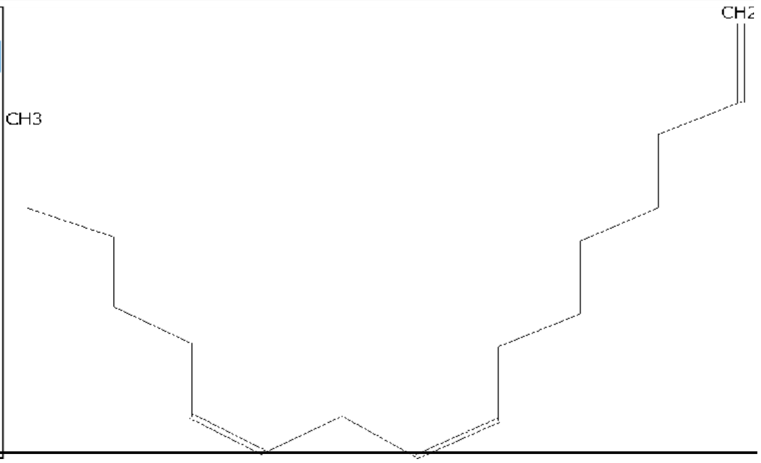


Acquisition Time (min)

 Generated at 5:24 PM on 9/22/2025

# Unknown Analysis Report - Best Hits

| RT | Compound Name | CAS# | Formula | Area | MI | Match Score | Sample | Sample |
|---|---|---|---|---|---|---|---|---|
| 13.8552 | Tricyclo[3.2.1.0(2,4)]octan-8-one, 3,3-dimethyl-, (1.alpha.,2.alpha.,4.alpha.,5.alpha.)- | 66930-01-6 | C10H14O | 13491723 | | 64.0 | 0.20 | 0.88 |

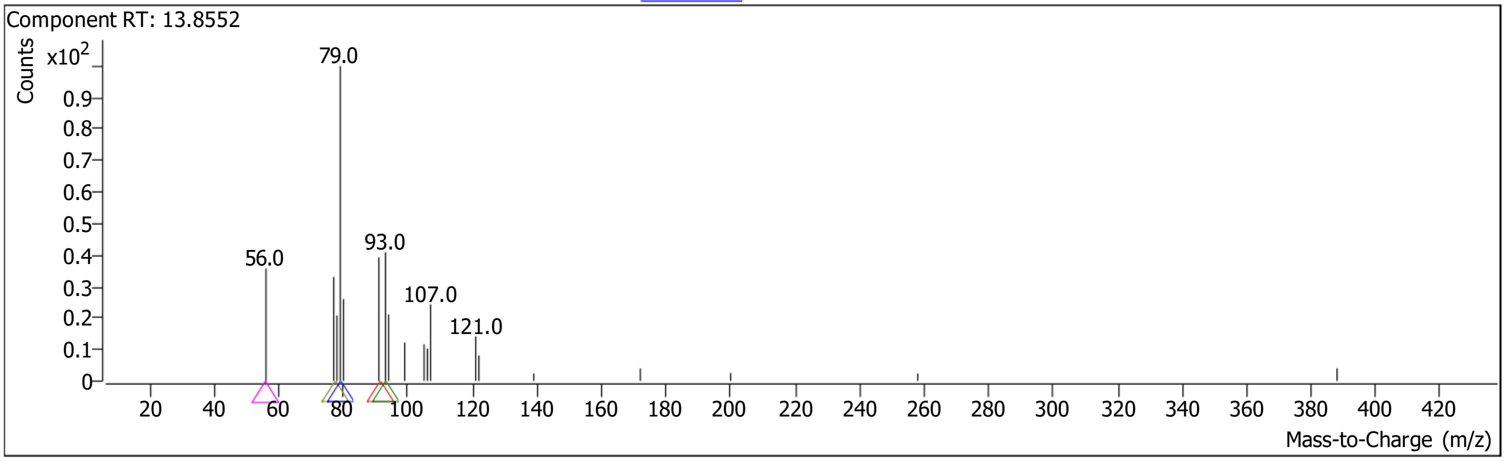


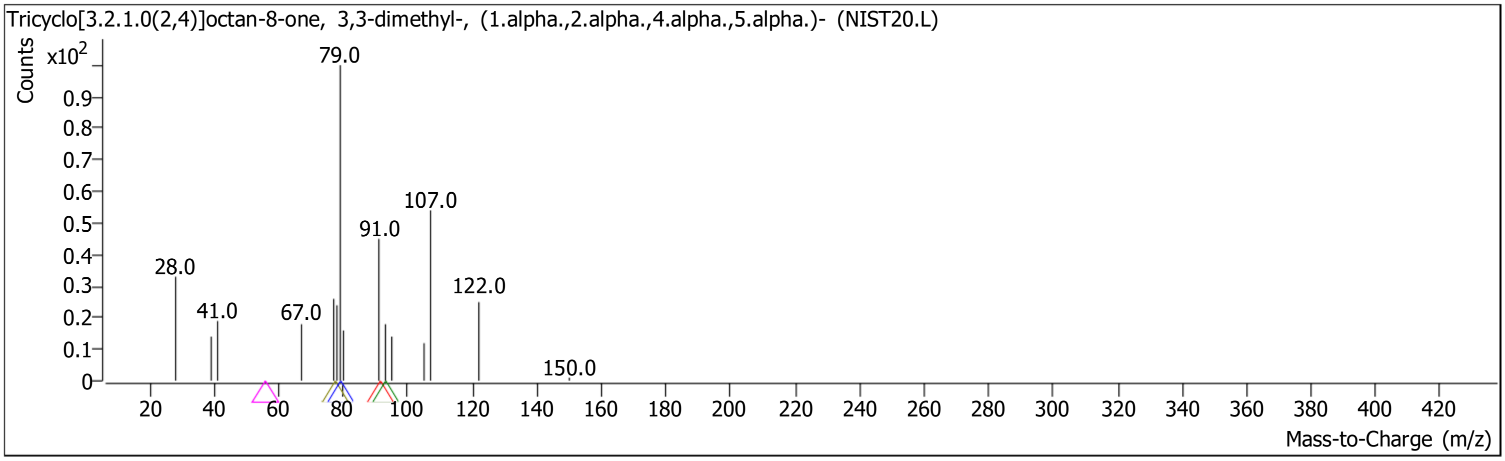


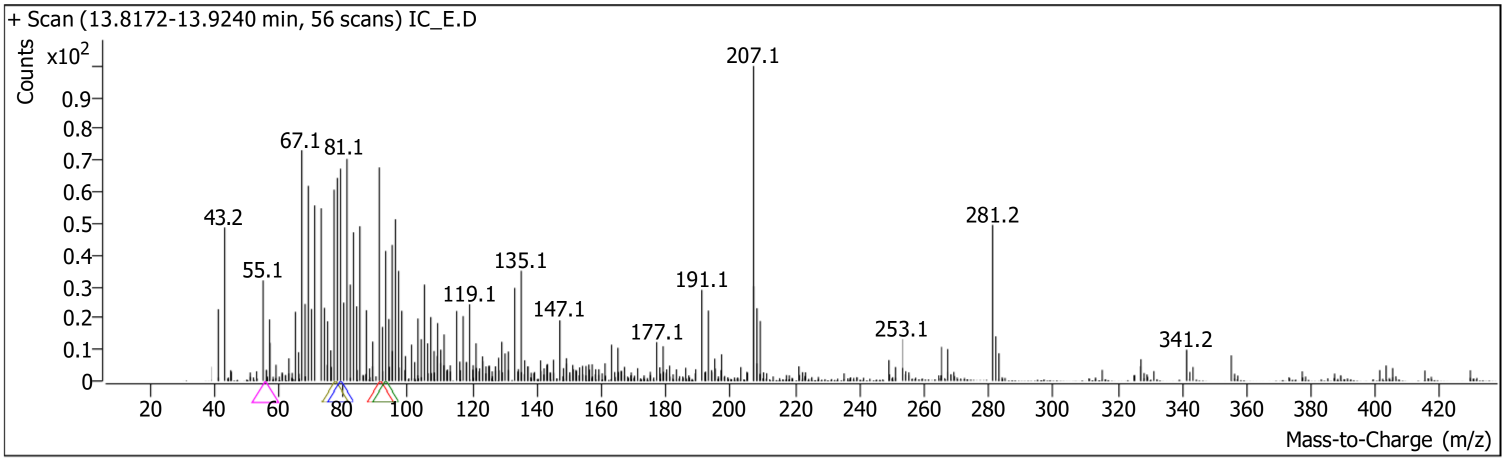


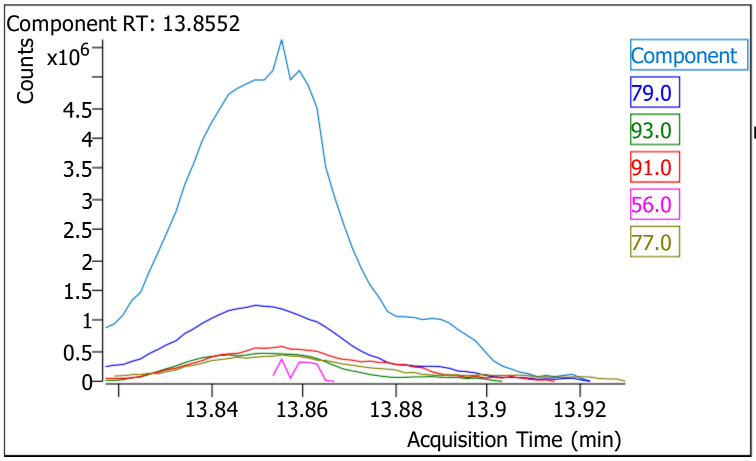


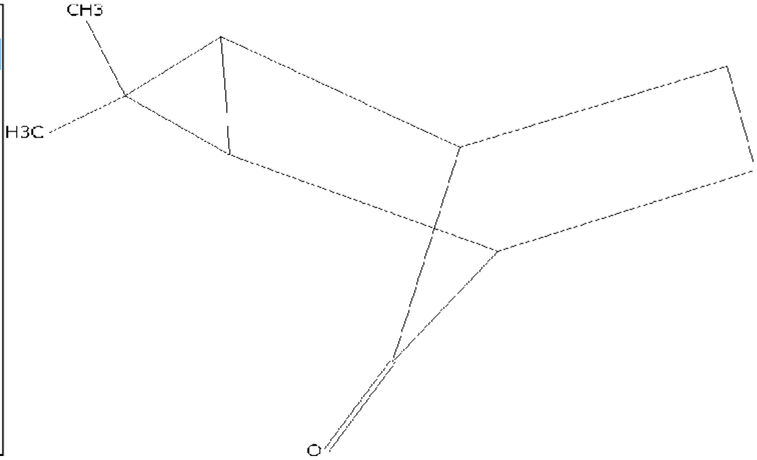

| RT | Compound Name | CAS# | Formula | Area | MI | Match Score | Sample | Sample |
|---|---|---|---|---|---|---|---|---|
| 13.8895 | 5-Cyclohexyl-1-pentene | 5729-54-4 | C11H20 | 7466289 | | 64.4 | 0.11 | 0.49 |

Component RT: 13.8895

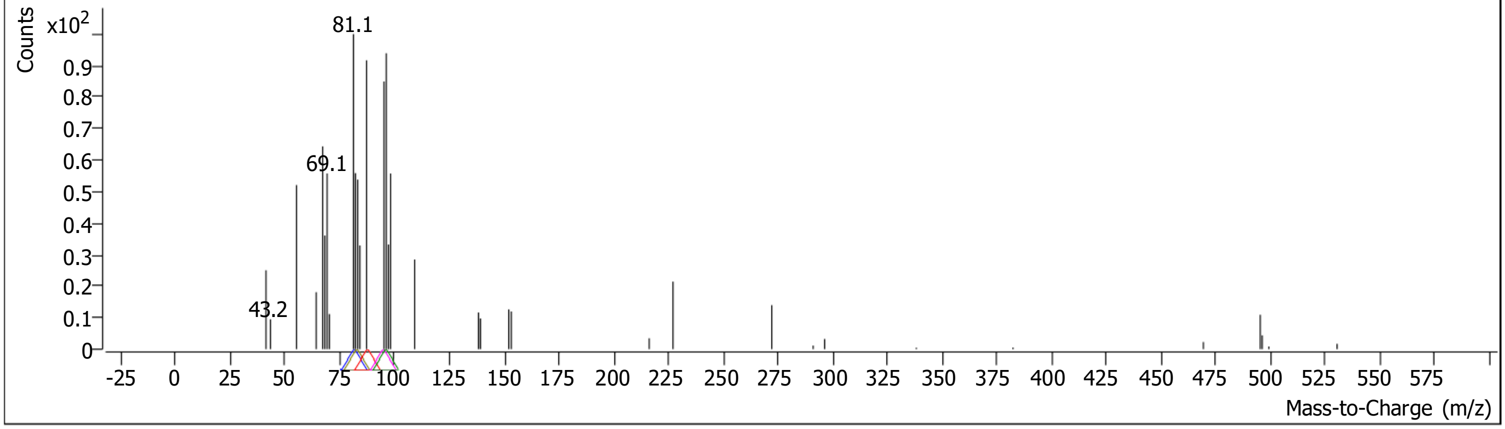


5-Cyclohexyl-1-pentene (NIST20.L)

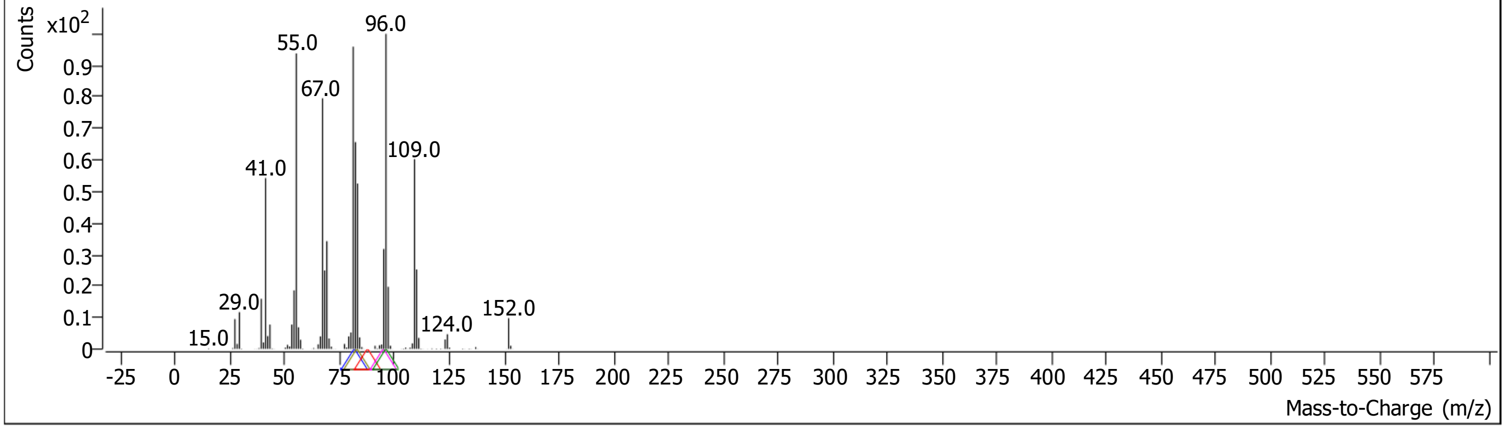


+ Scan (13.8744-13.9107 min, 20 scans) IC_E.D

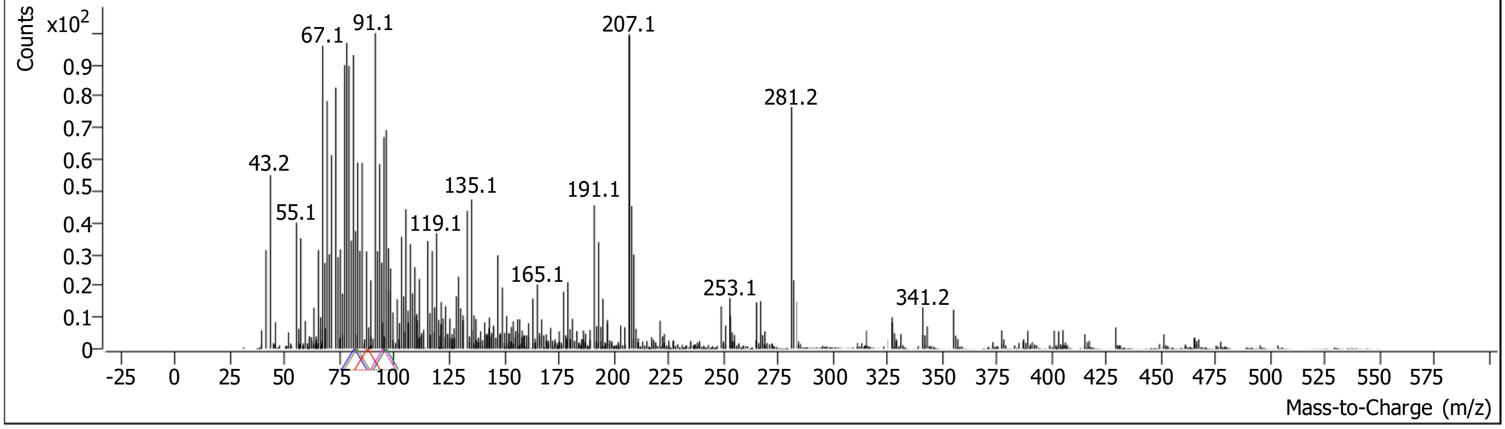


Component RT: 13.8895

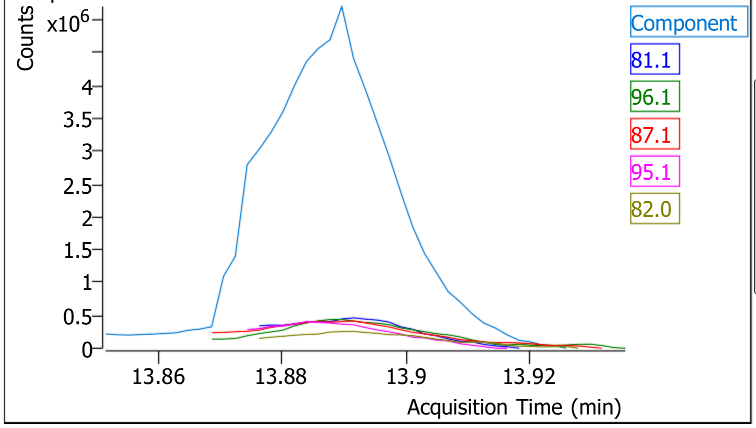


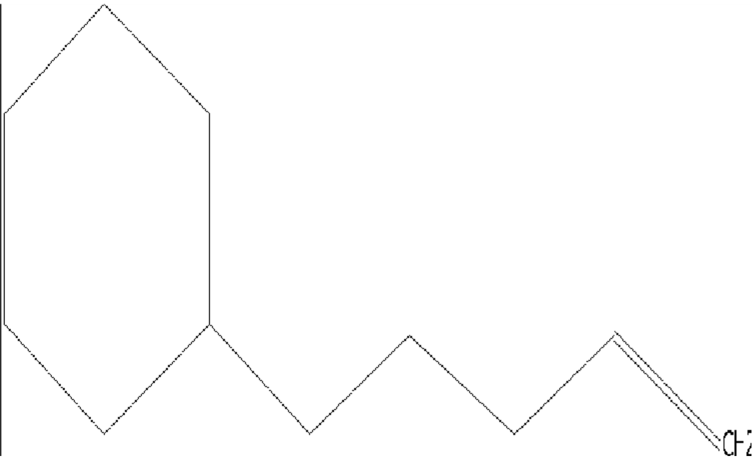

# Unknown Analysis Report - Best Hits

| RT | Compound Name | CAS# | Formula | Area | MI | Match Score | Sample | Sample |
|---|---|---|---|---|---|---|---|---|
| 13.9583 | dl-Menthol | 89-78-1 | C10H20O | 44635658 | | 80.0 | 0.65 | 2.92 |

Component RT: 13.9583

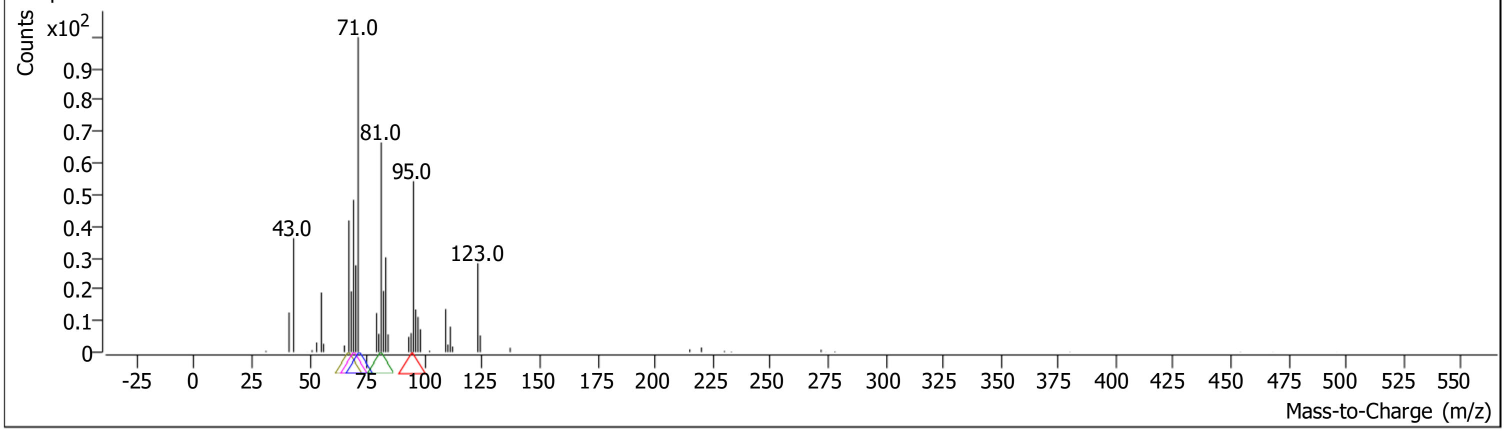


dl-Menthol (NIST20.L)

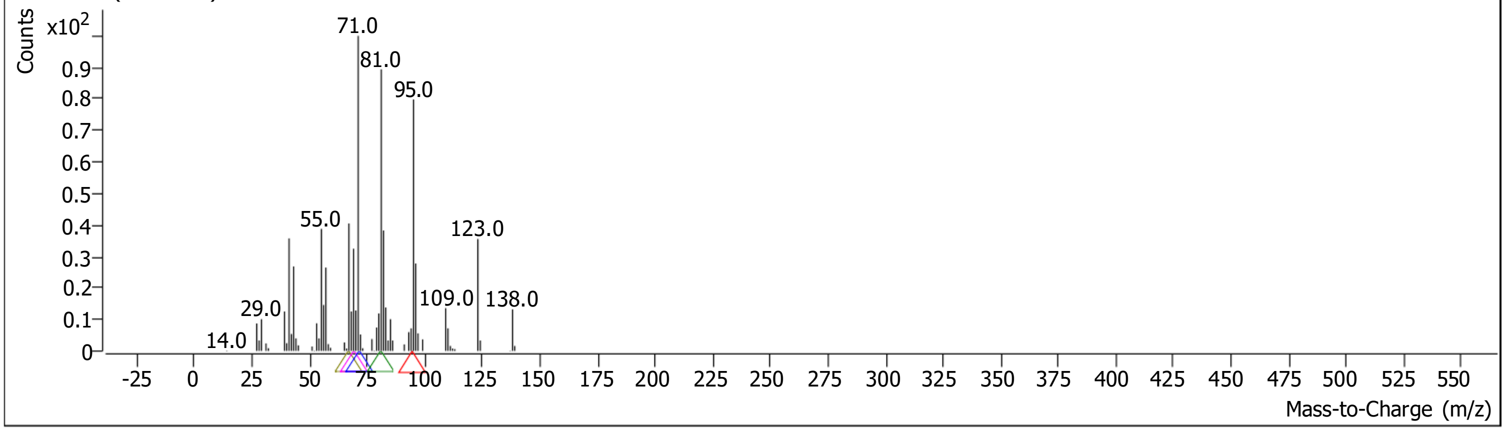


+ Scan (13.8840-14.0194 min, 72 scans) IC_E.D

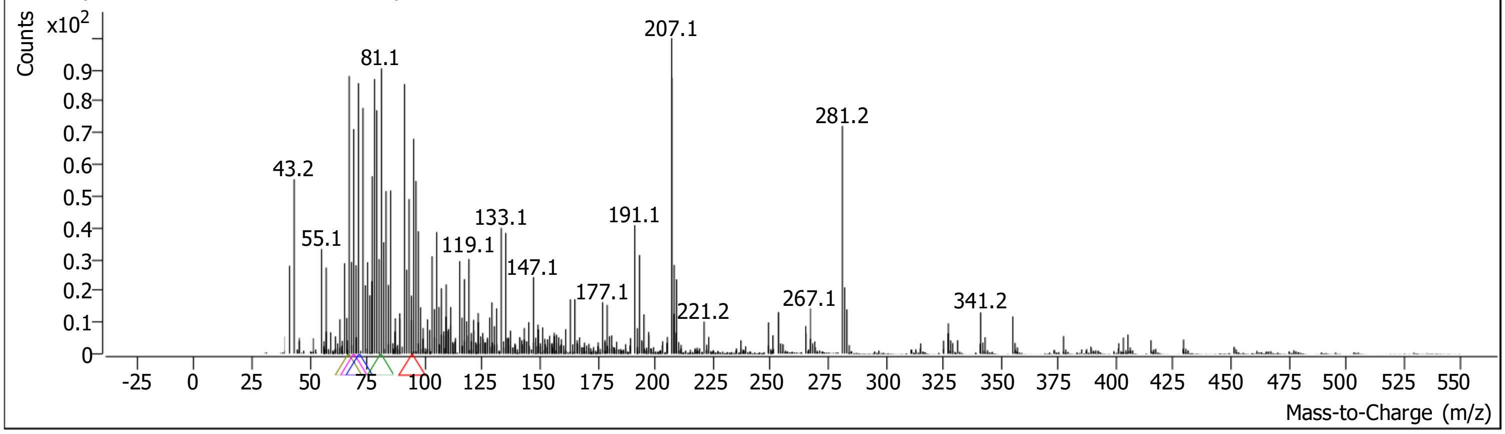


Component RT: 13.9583

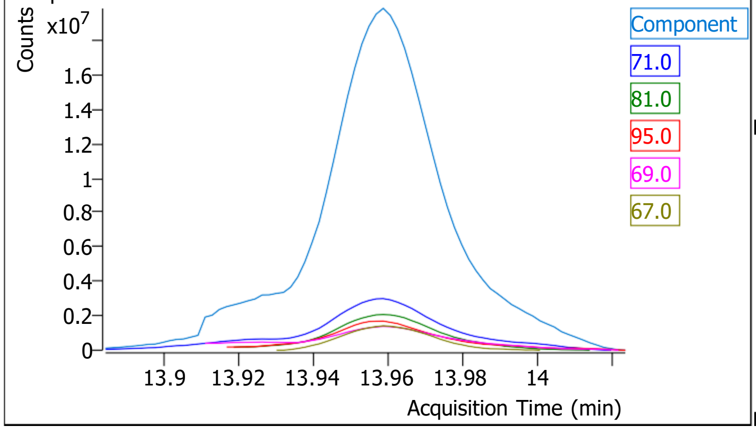


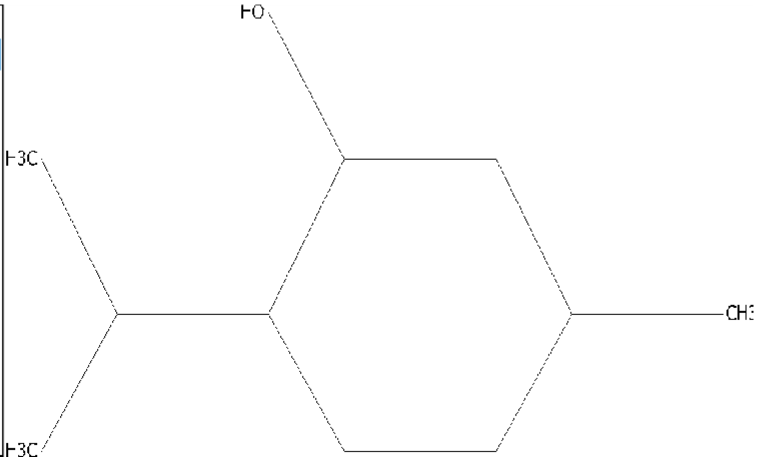

# Unknown Analysis Report - Best Hits

| RT | Compound Name | CAS# | Formula | Area | MI | Match Score | Sample | Sample |
|---|---|---|---|---|---|---|---|---|
| 14.1866 | 3-Ethyl-3-methylheptane | 17302-01-1 | C10H22 | 29404028 | | 79.9 | 0.43 | 1.92 |

Component RT: 14.1866

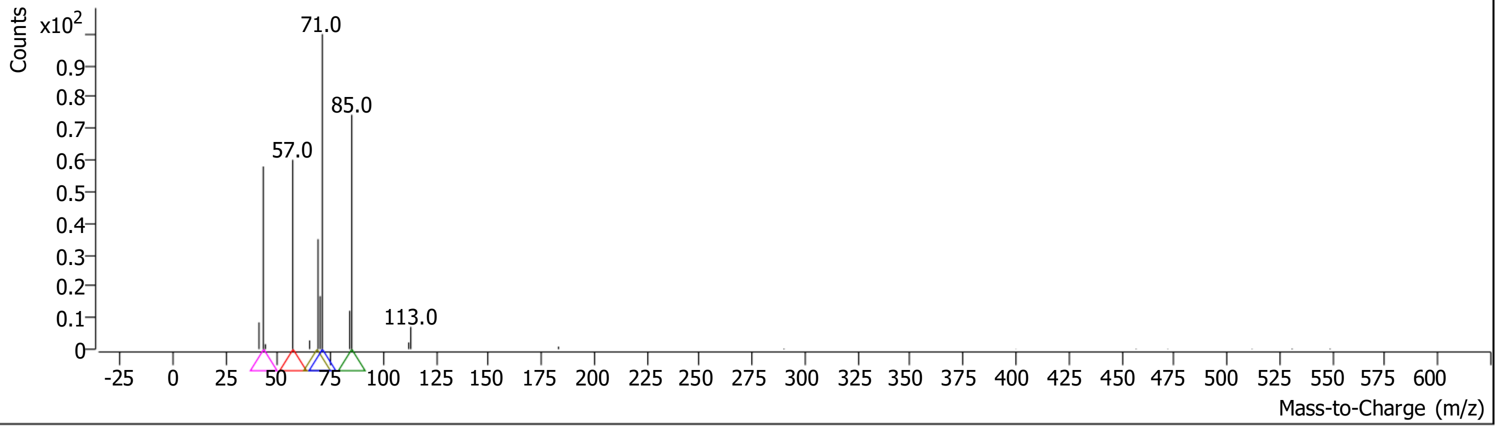


3-Ethyl-3-methylheptane (NIST20.L)

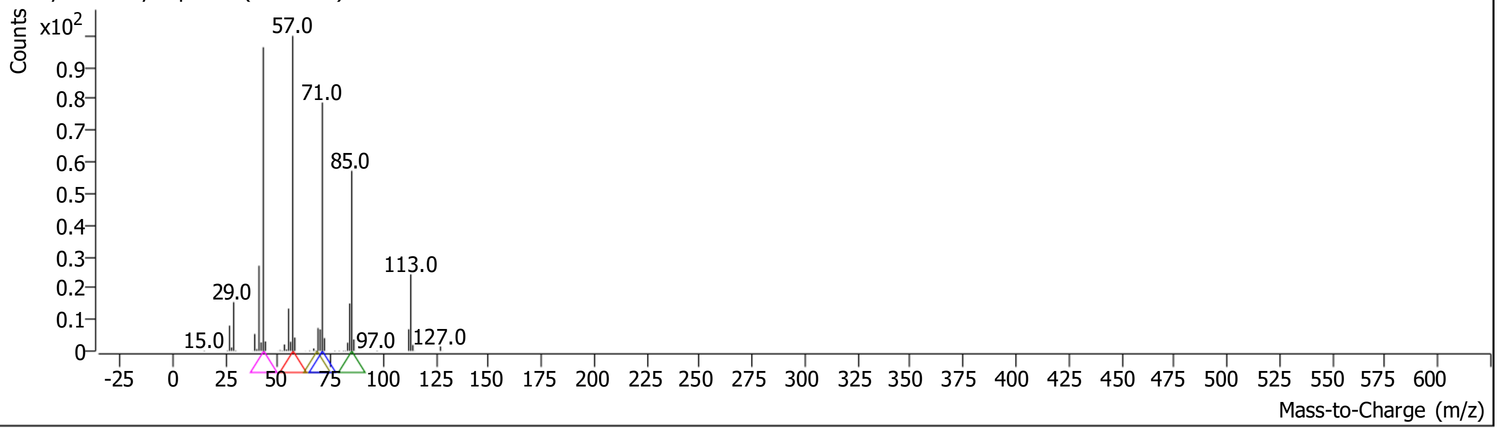


+ Scan (14.1587-14.2255 min, 36 scans) IC_E.D

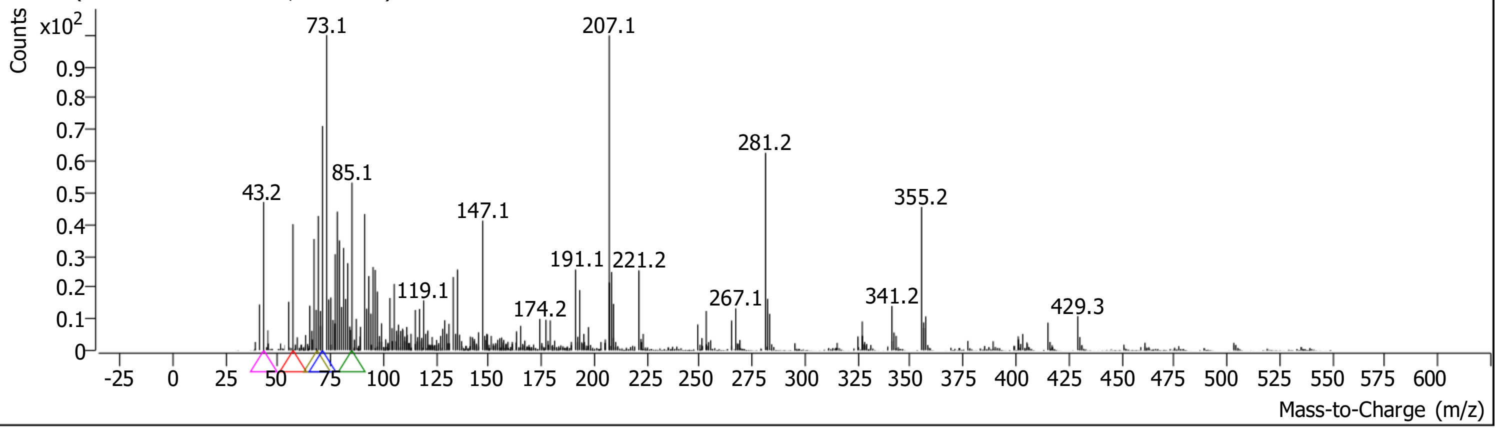


Component RT: 14.1866

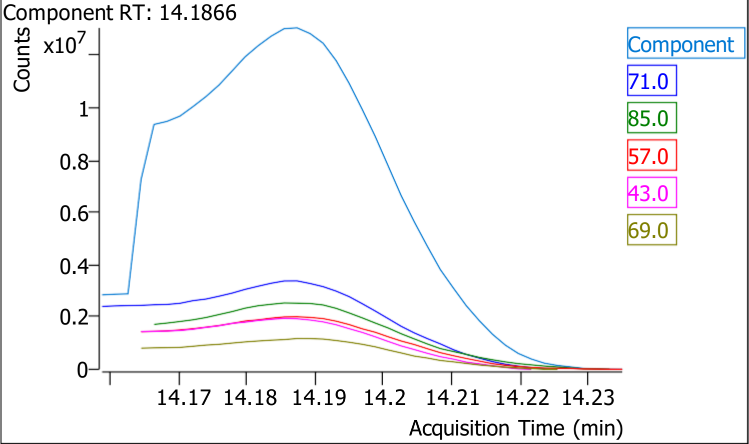


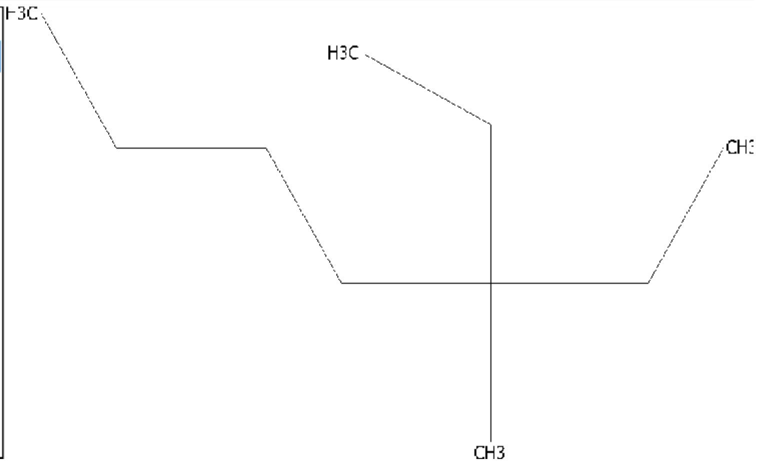

# Unknown Analysis Report - Best Hits

| RT | Compound Name | CAS# | Formula | Area | MI | Match Score | Sample | Sample |
|---|---|---|---|---|---|---|---|---|
| 14.5574 | Sulfurous acid, isohexyl pentyl ester | 1000309-14-0 | C11H24O3S | 8084505 | | 61.0 | 0.12 | 0.53 |

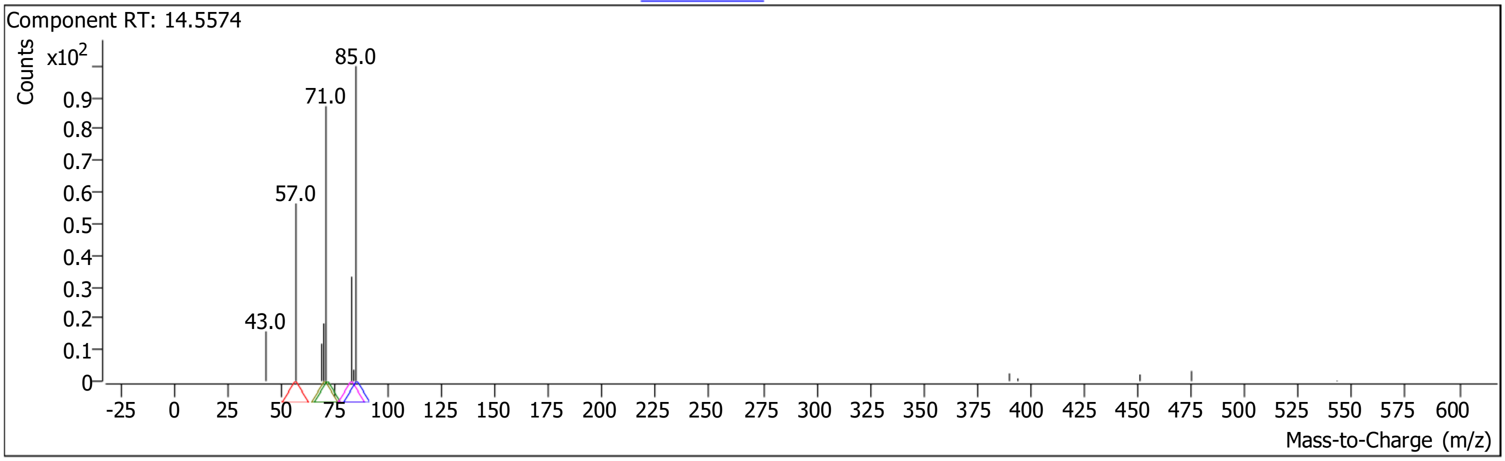


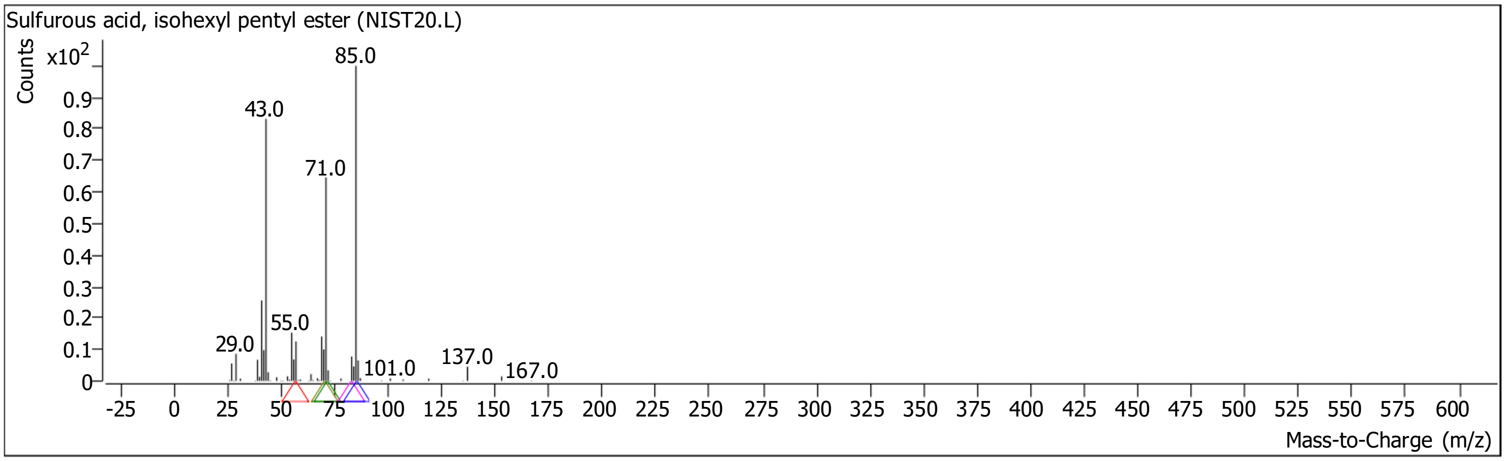


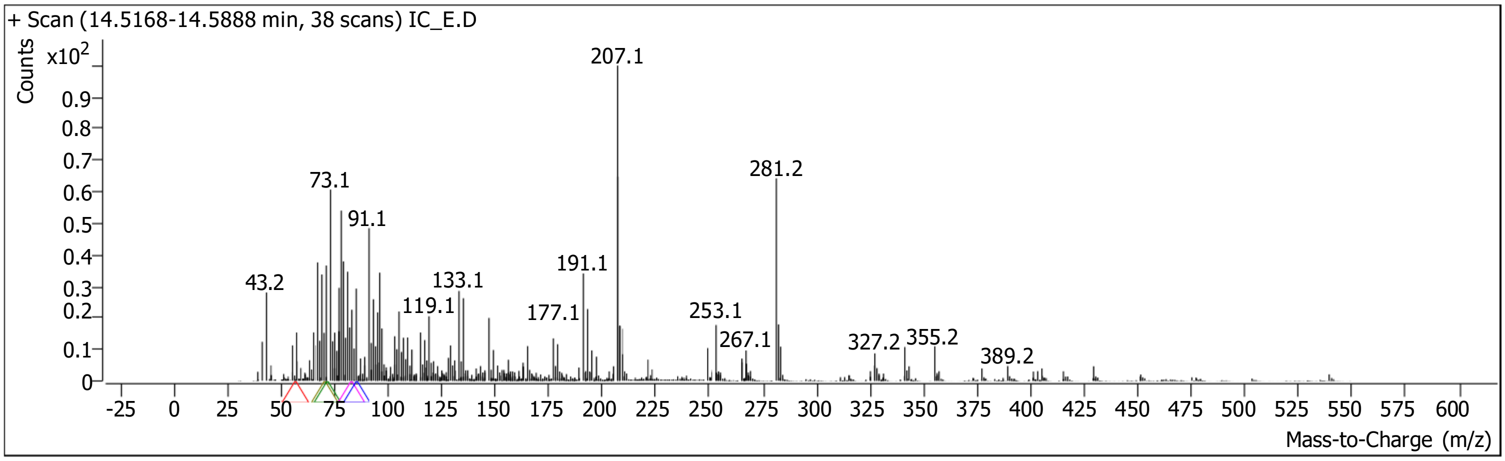


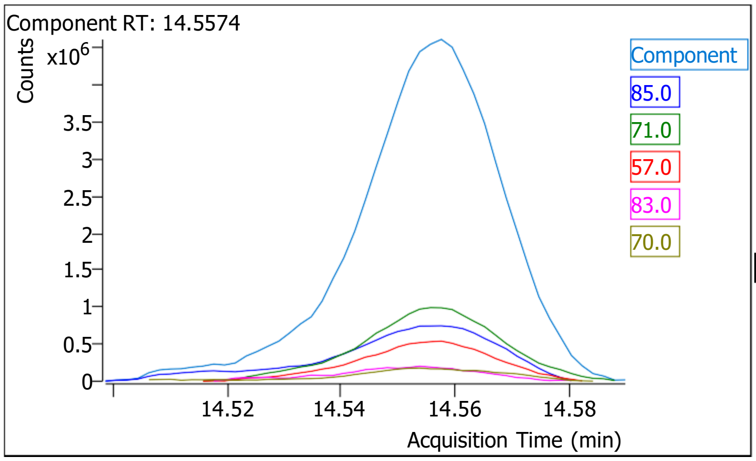


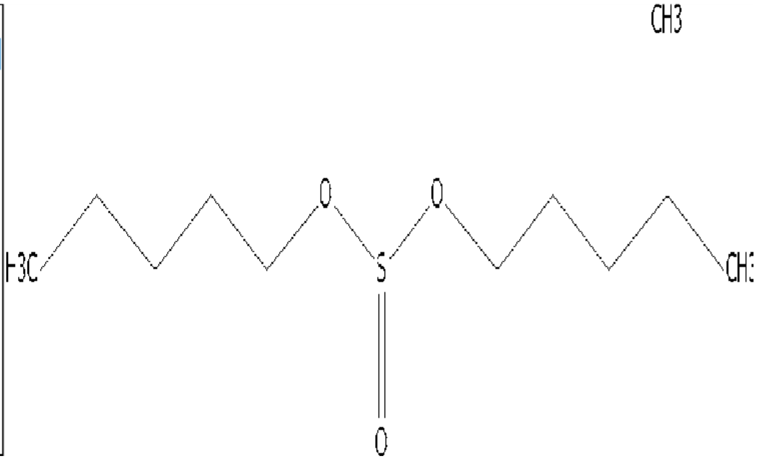


 Generated at 5:24 PM on 9/22/2025

# Unknown Analysis Report - Best Hits

| RT | Compound Name | CAS# | Formula | Area | MI | Match Score | Sample | Sample |
|---|---|---|---|---|---|---|---|---|
| 15.5630 | Sulfurous acid, nonyl pentyl ester | 1000309-14-2 | C14H30O3S | 7762090 | | 70.9 | 0.11 | 0.51 |

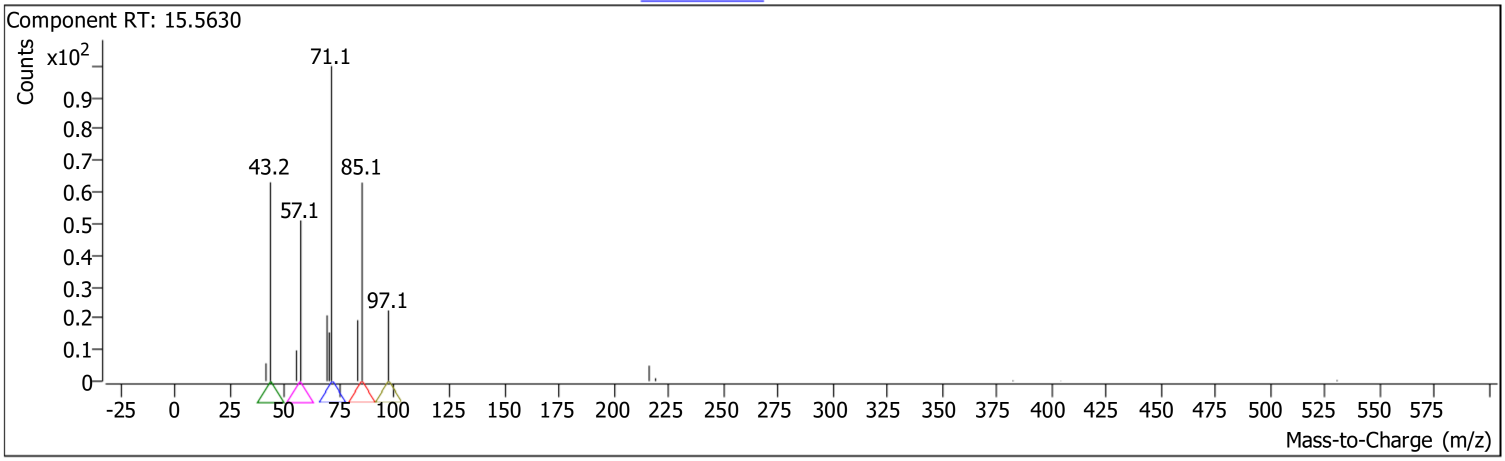


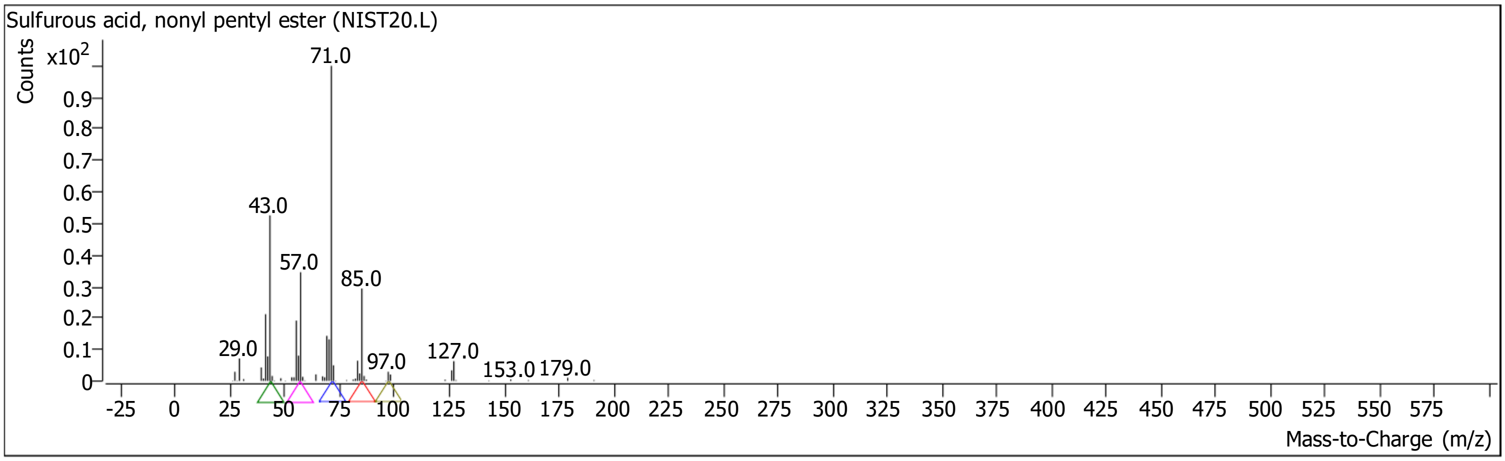


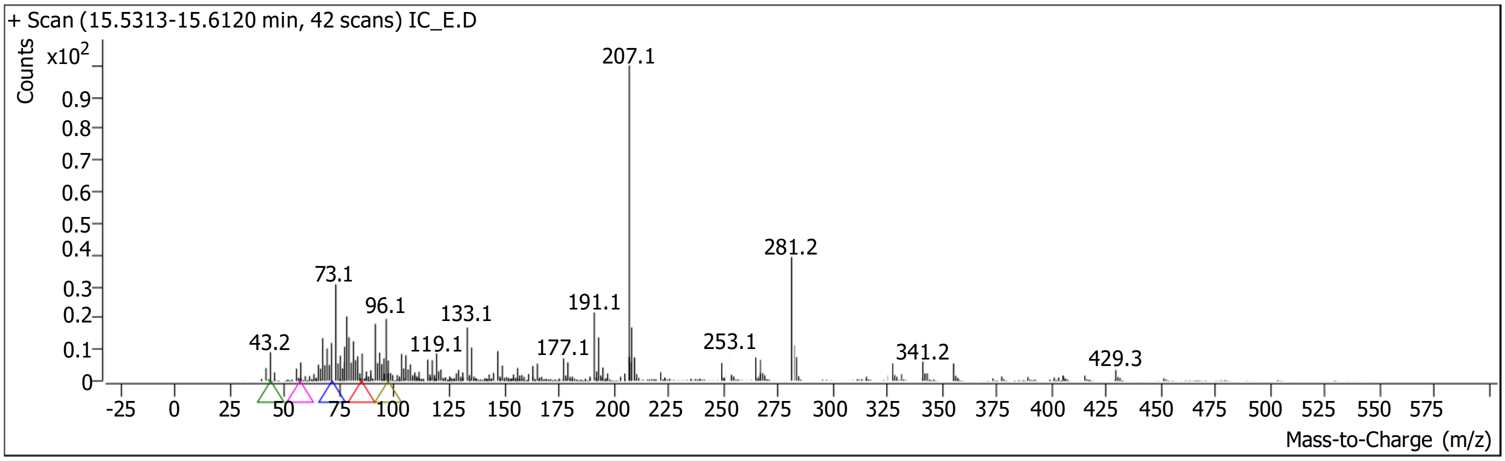


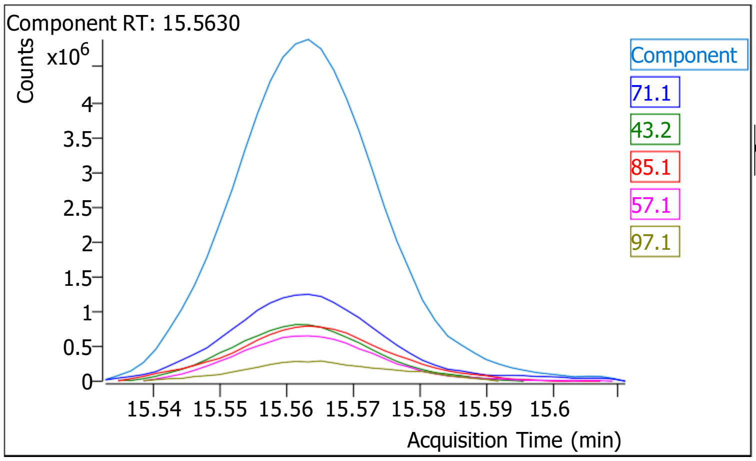


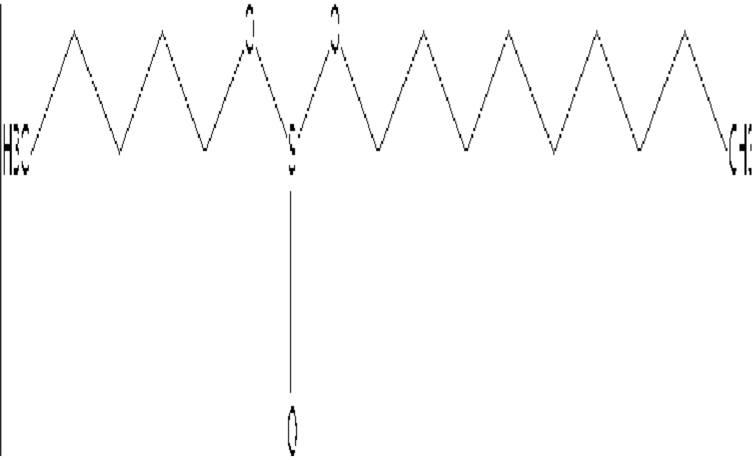

| RT | Compound Name | CAS# | Formula | Area | MI | Match Score | Sample | Sample |
|---|---|---|---|---|---|---|---|---|
| 15.8950 | Benzene, (3-octylundecyl)- | 5637-96-7 | C25H44 | 7628143 | | 60.2 | 0.11 | 0.50 |

Component RT: 15.8950

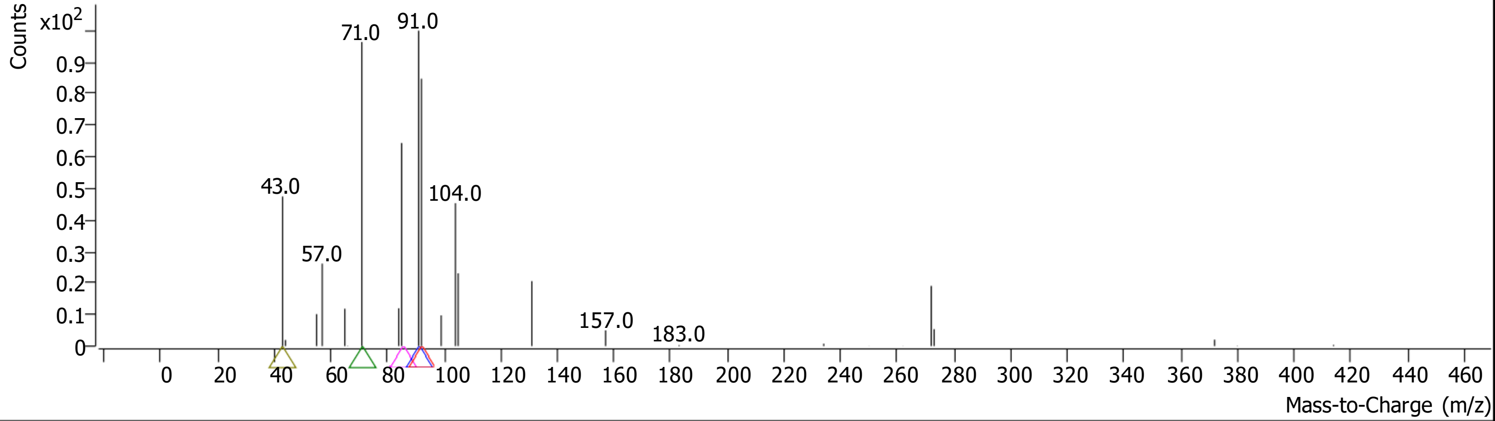


Benzene, (3-octylundecyl)- (NIST20.L)

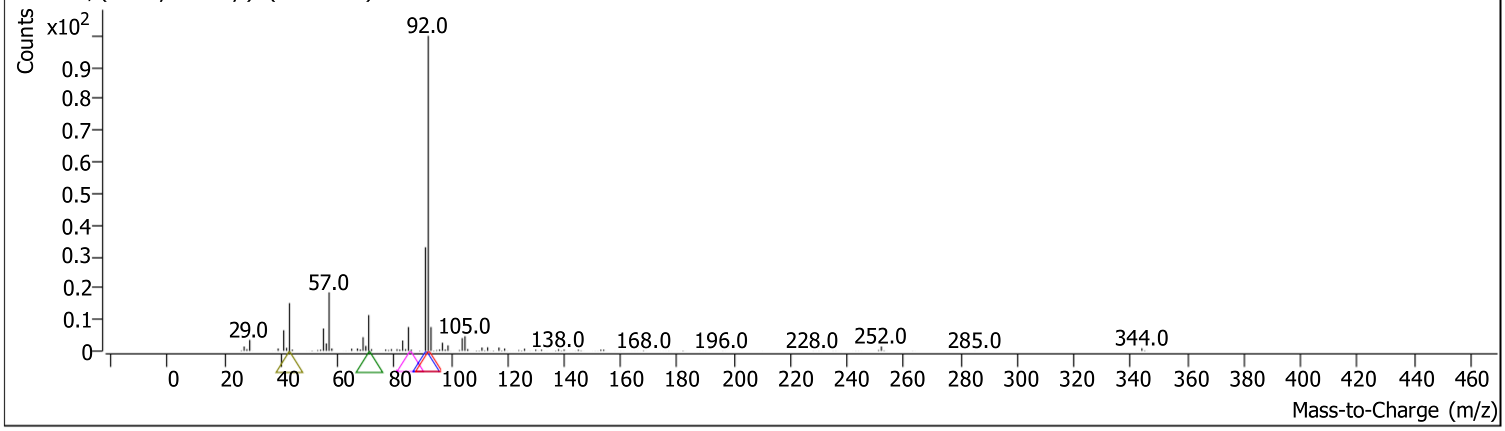


+ Scan (15.8801-15.8839 min, 3 scans) IC_E.D

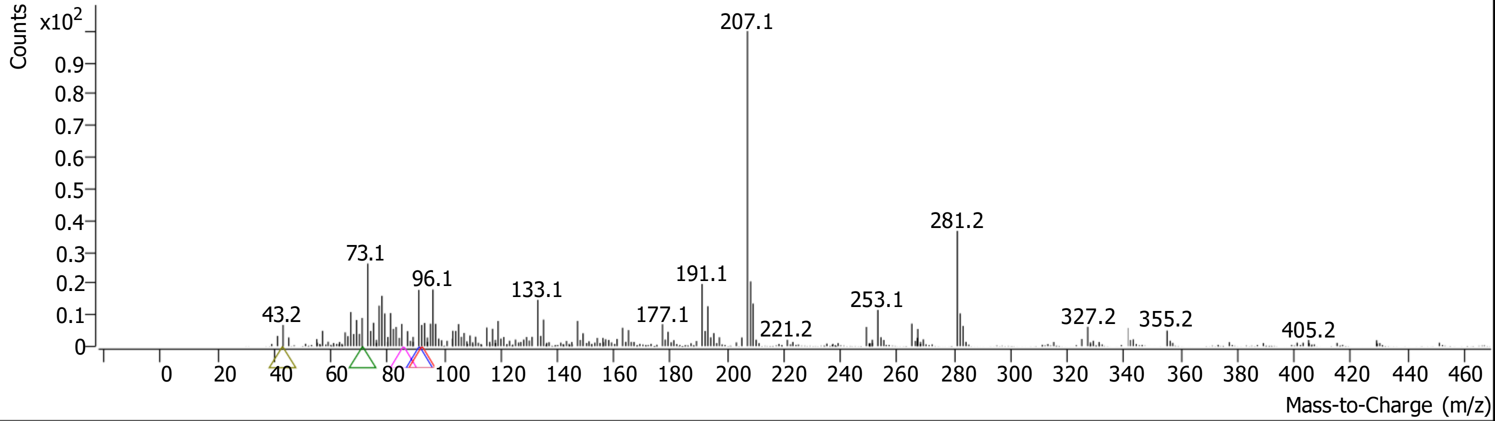


Component RT: 15.8950

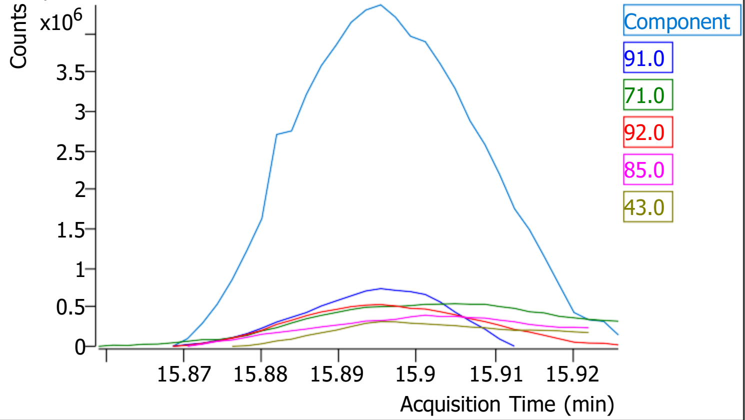


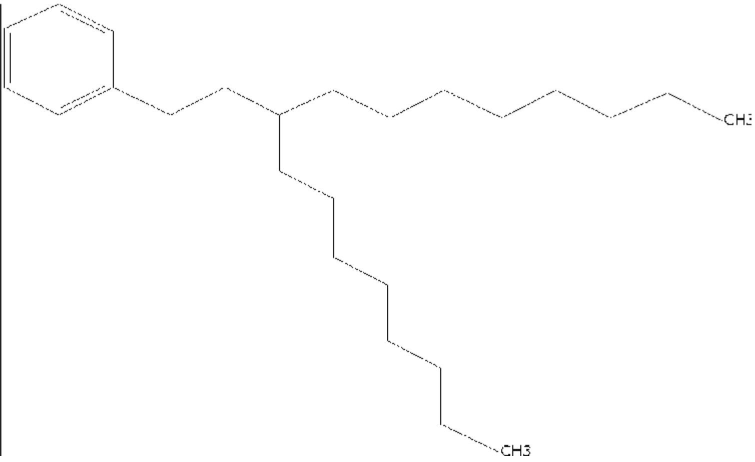

| RT | Compound Name | CAS# | Formula | Area | MI | Match Score | Sample | Sample |
|---|---|---|---|---|---|---|---|---|
| 17.2228 | benzenamine, 4-methyl-N-(4-methylphenyl)-N-[4-[2-(4-methylphenyl)ethenyl]phenyl]- | 1000402-70-2 | C29H27N | 13203886 | | 65.6 | 0.19 | 0.86 |

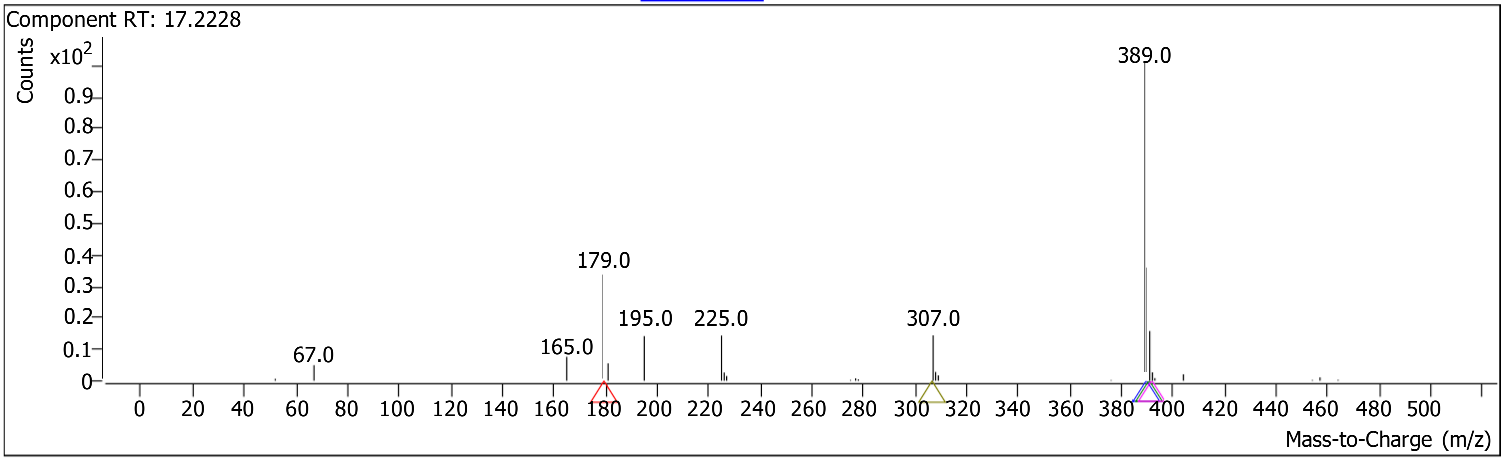


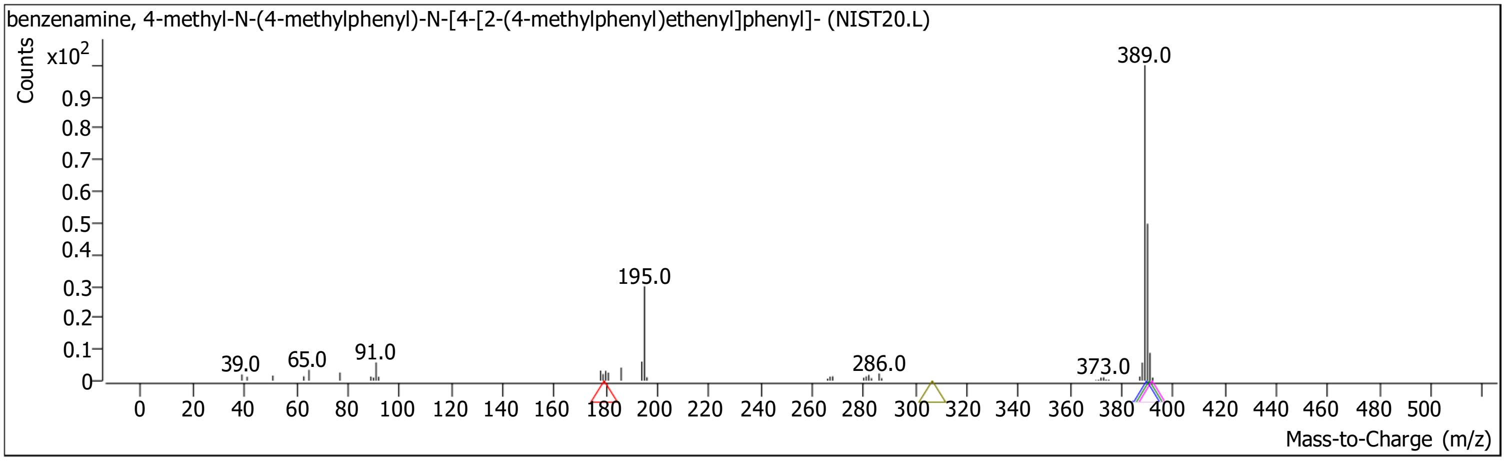


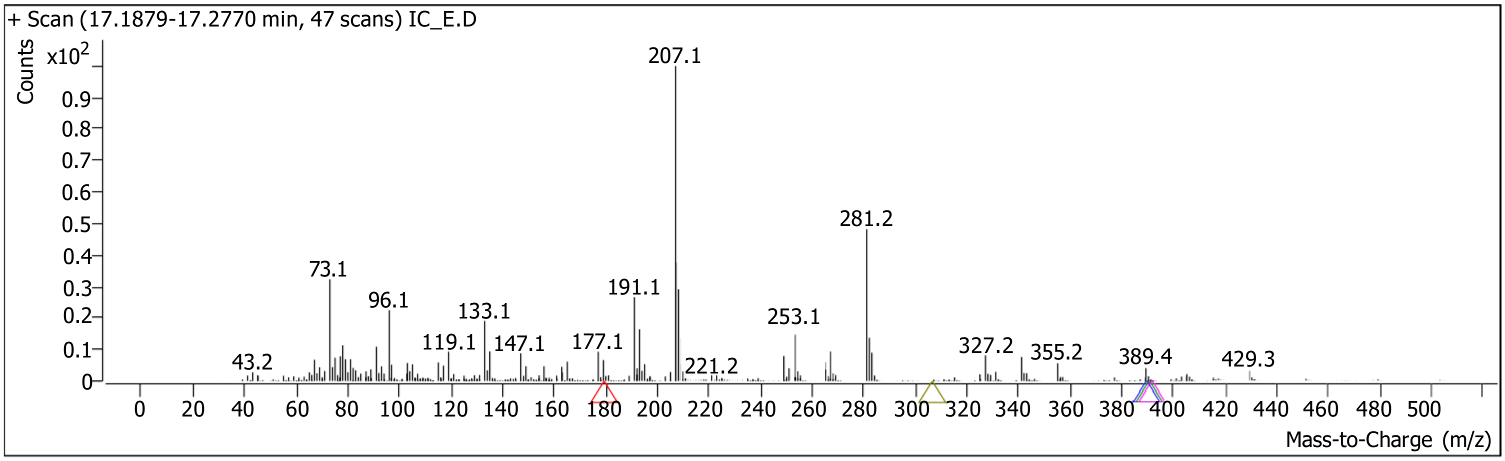


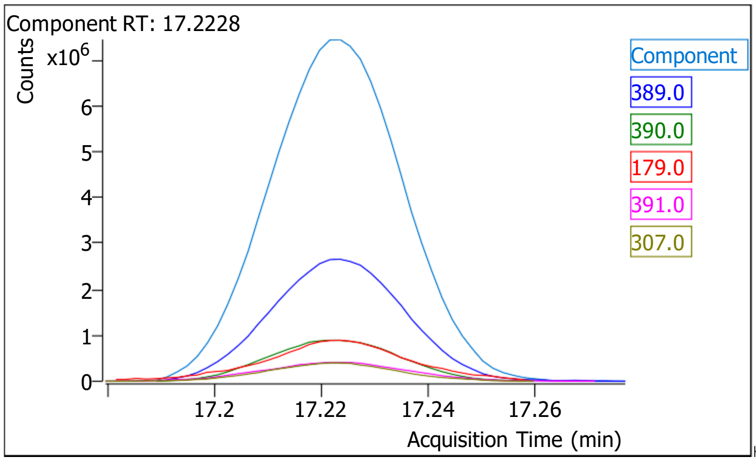


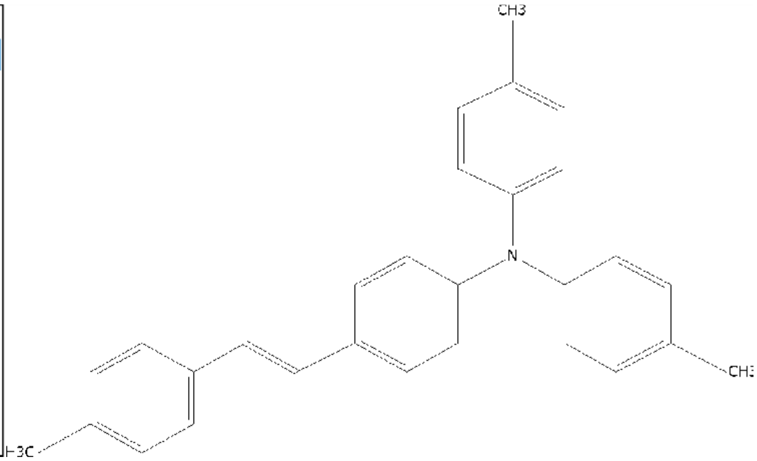

| RT | Compound Name | CAS# | Formula | Area | MI | Match Score | Sample | Sample |
|---|---|---|---|---|---|---|---|---|
| 19.6856 | 2-Methyl-3-(3-methyl-but-2-enyl)-2-(4-methyl-pent-3-enyl)-oxetane | 1000144-10-2 | C15H26O | 11278308 | | 64.2 | 0.16 | 0.74 |

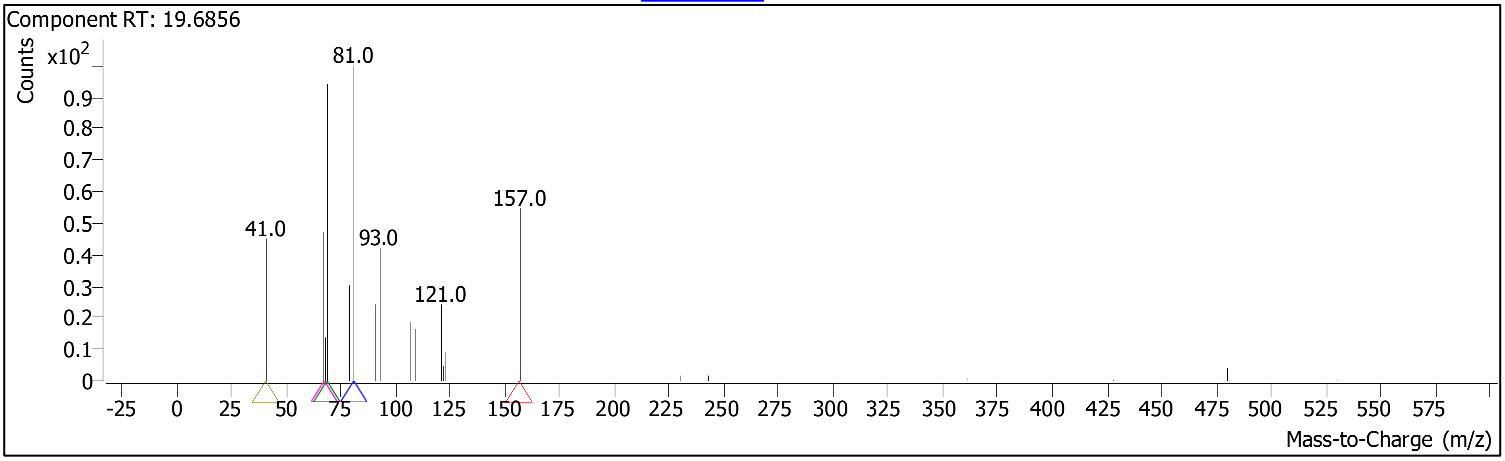


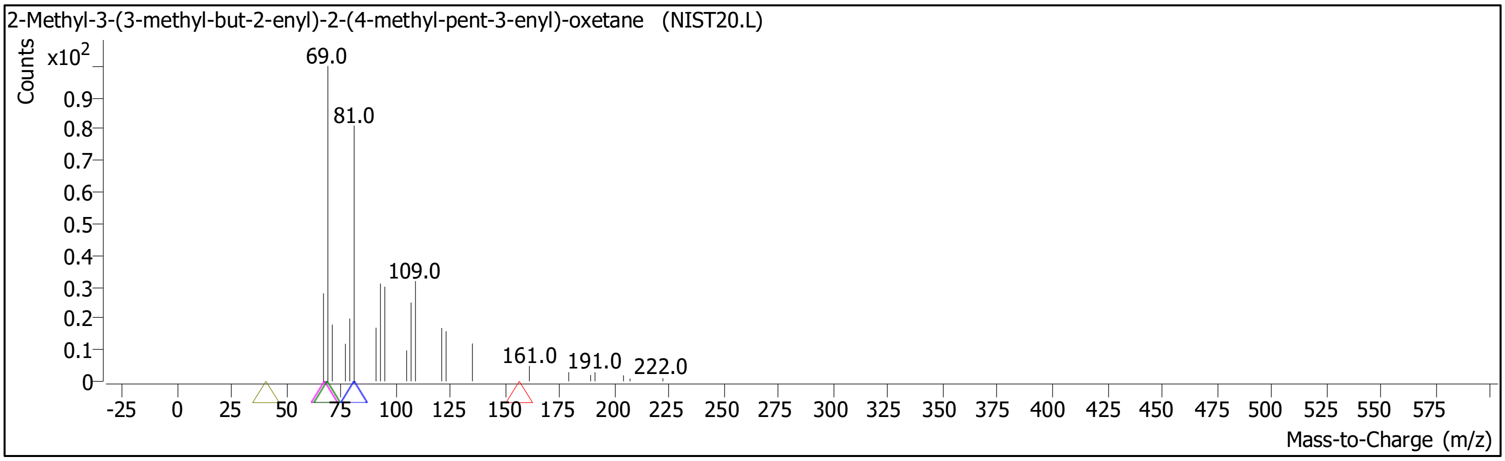


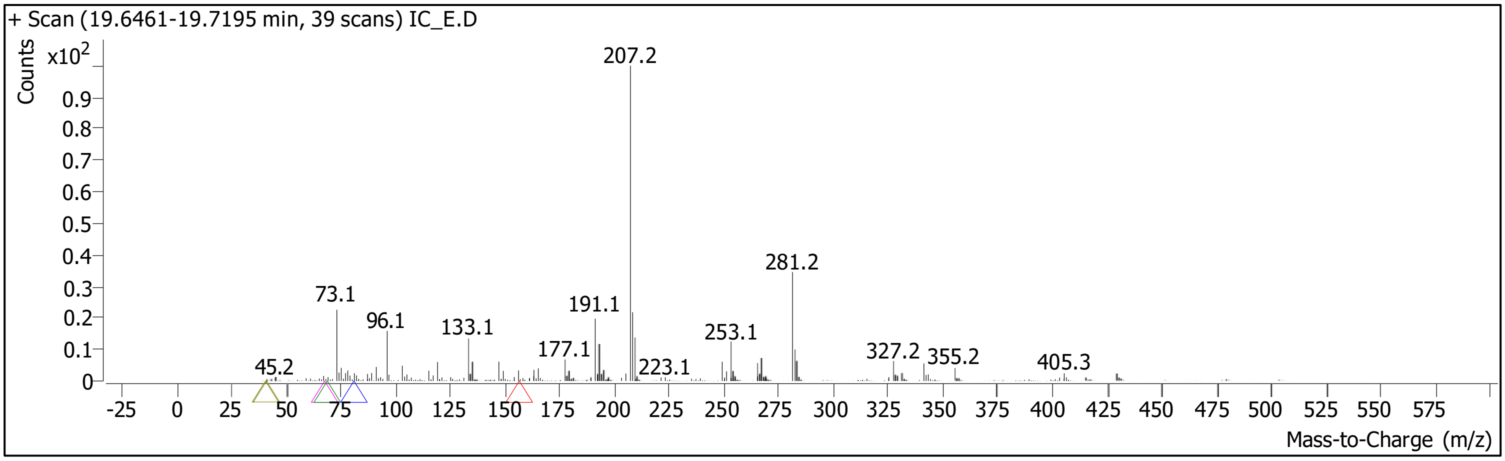


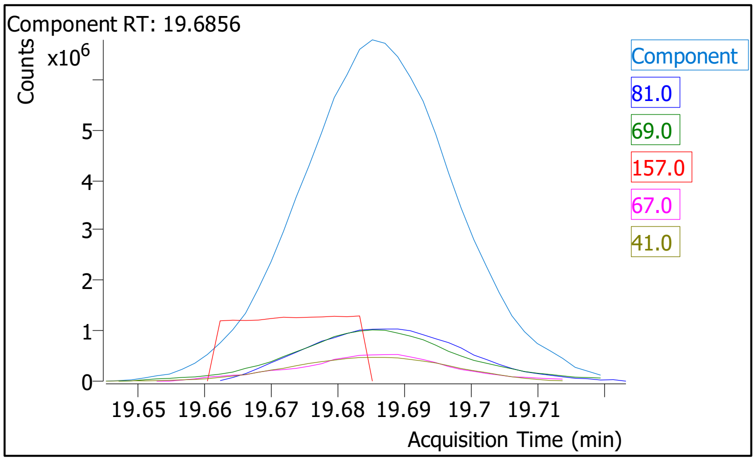


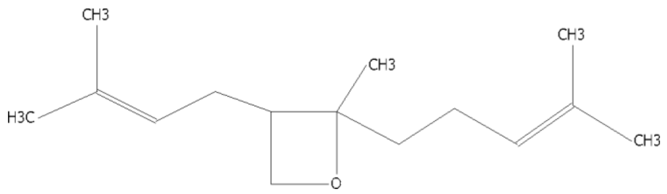


 Generated at 5:24 PM on 9/22/2025

# Unknown Analysis Report - Best Hits

| RT | Compound Name | CAS# | Formula | Area | MI | Match Score | Sample | Sample |
|---|---|---|---|---|---|---|---|---|
| 20.1259 | 1H-Pyrazole, 4,5-dihydro-3,4,5-trimethyl- | 22591-95-3 | C6H12N2 | 647995 | | 62.8 | 0.01 | 0.04 |

Component RT: 20.1259

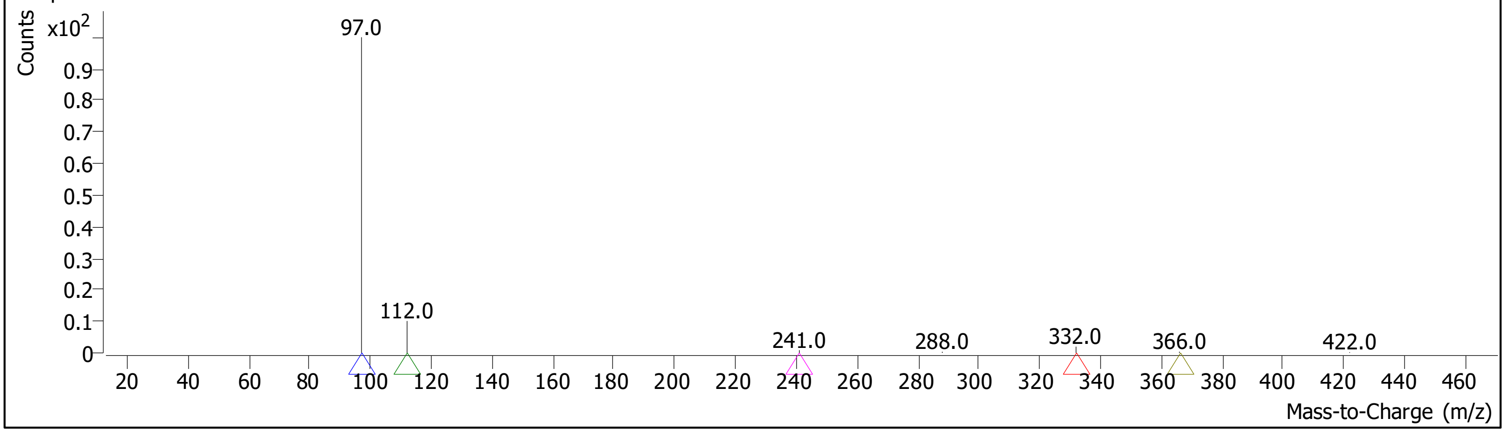


1H-Pyrazole, 4,5-dihydro-3,4,5-trimethyl- (NIST20.L)

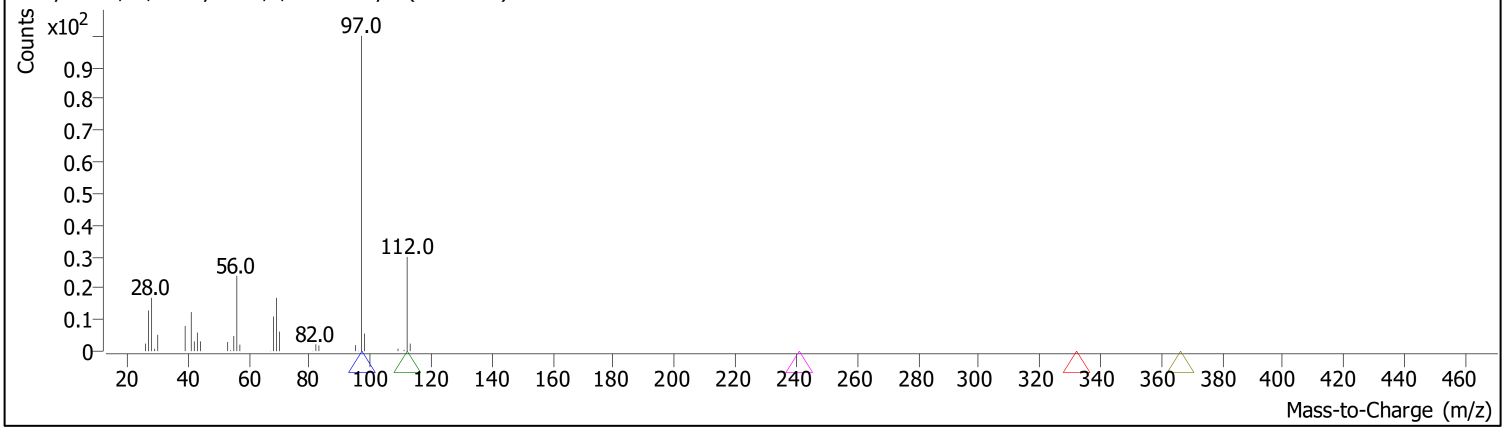


+ Scan (20.1241-20.1298 min, 3 scans) IC_E.D

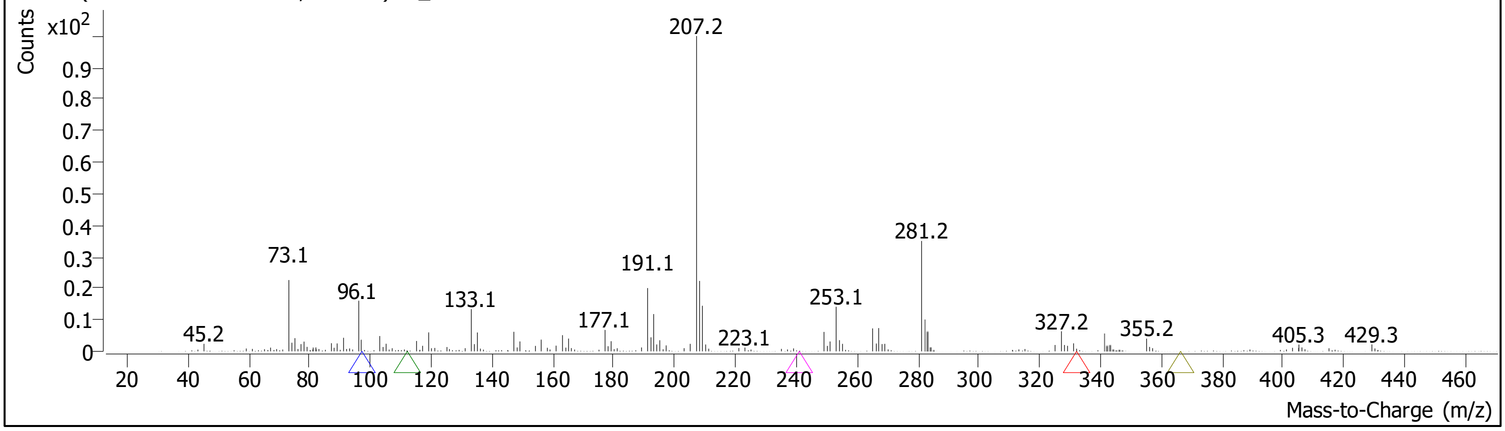


Component RT: 20.1259

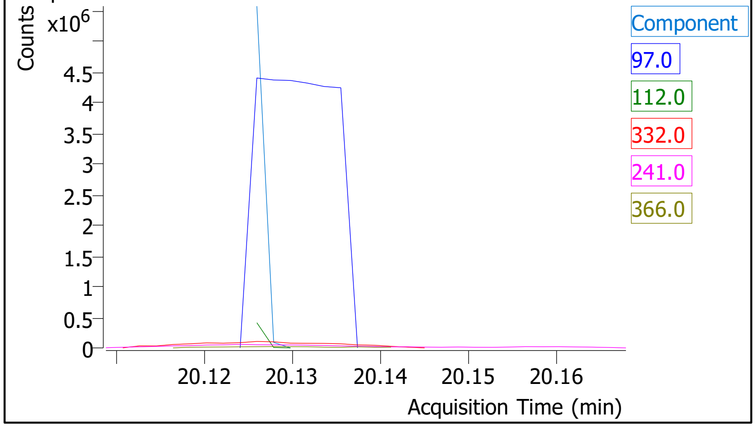


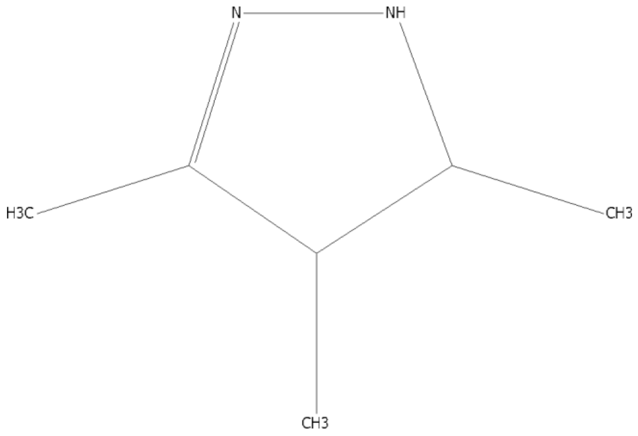

# Unknown Analysis Report - Best Hits

| RT | Compound Name | CAS# | Formula | Area | MI | Match Score | Sample | Sample |
|---|---|---|---|---|---|---|---|---|
| 23.9558 | Epilupeol; 20(29)-Lupen-3alpha-ol, acetate (isomer 1) | 1000513-01-7 | C32H52O2 | 220811549 | | 68.4 | 3.20 | 14.43 |

Component RT: 23.9558

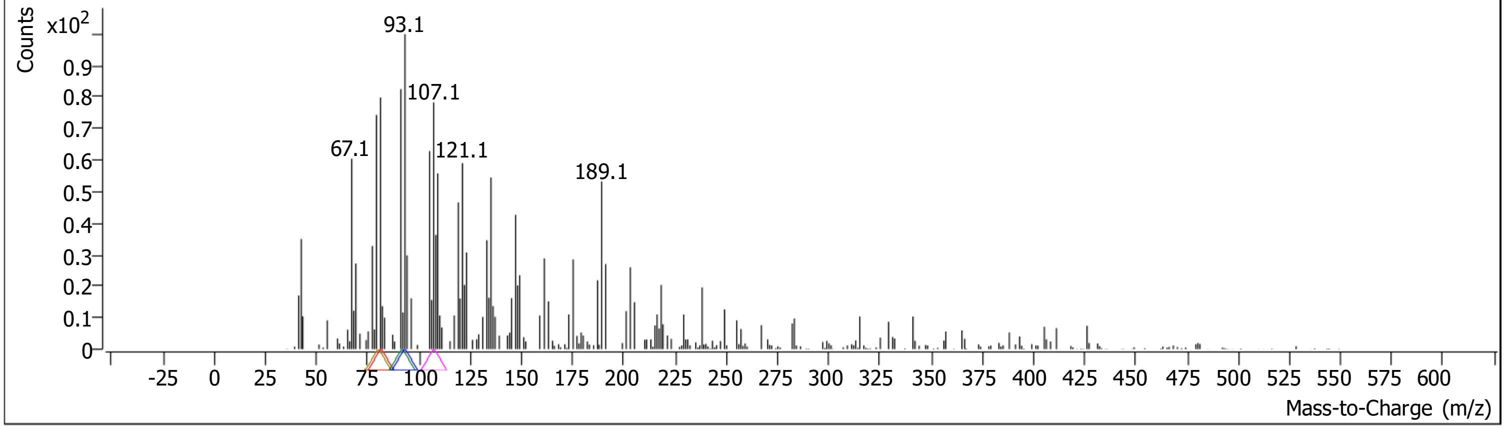


Epilupeol; 20(29)-Lupen-3alpha-ol, acetate (isomer 1) (NIST20.L)

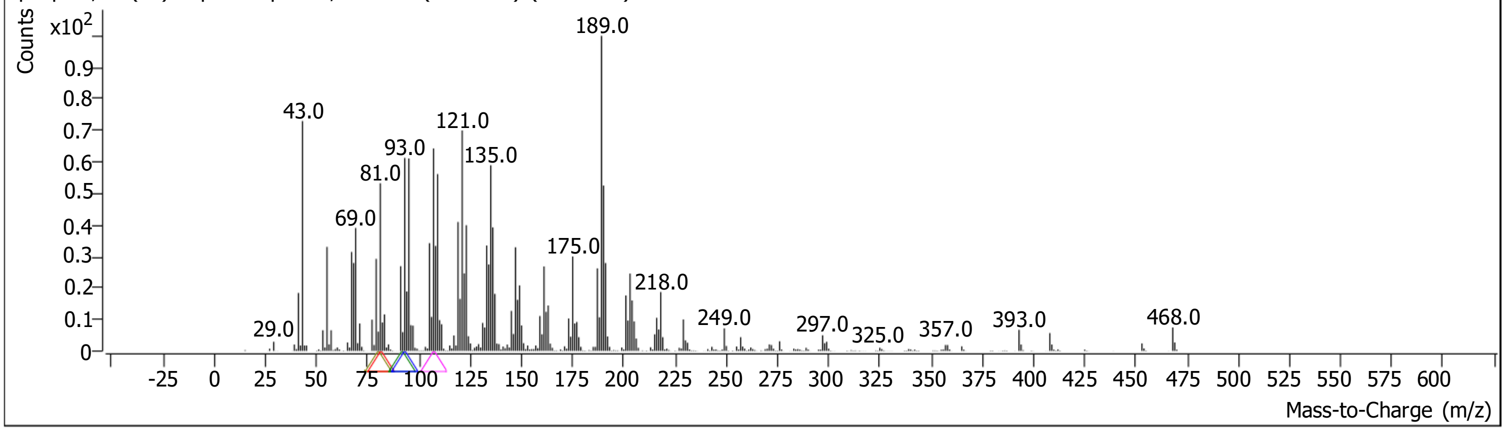


+ Scan (23.9014-24.0846 min, 96 scans) IC_E.D

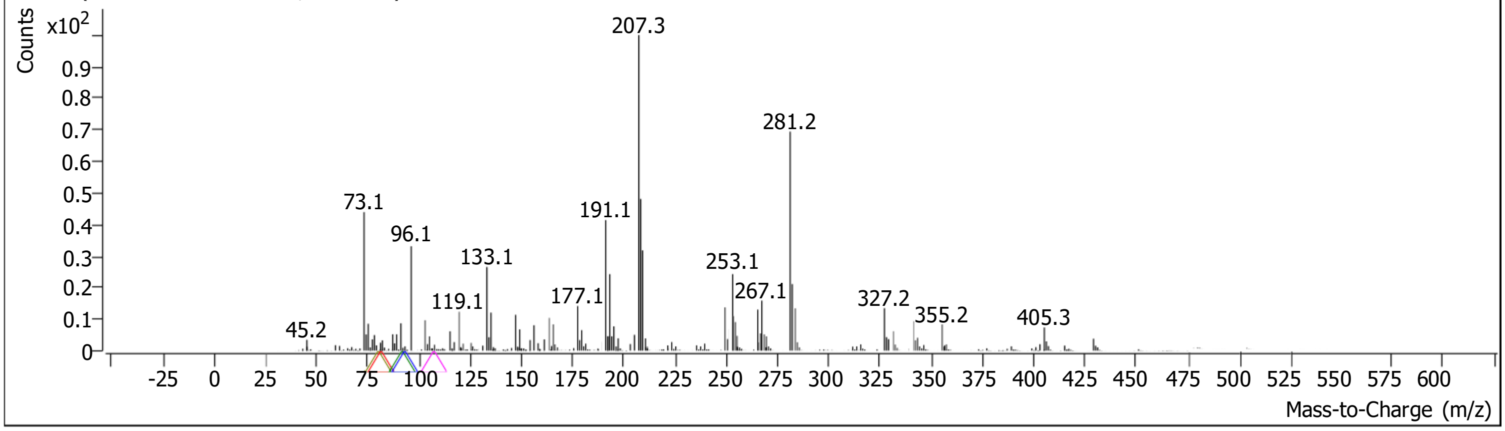


Component RT: 23.9558

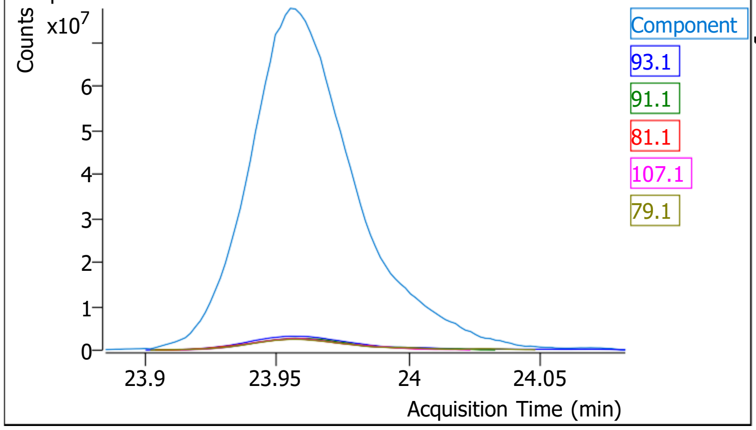


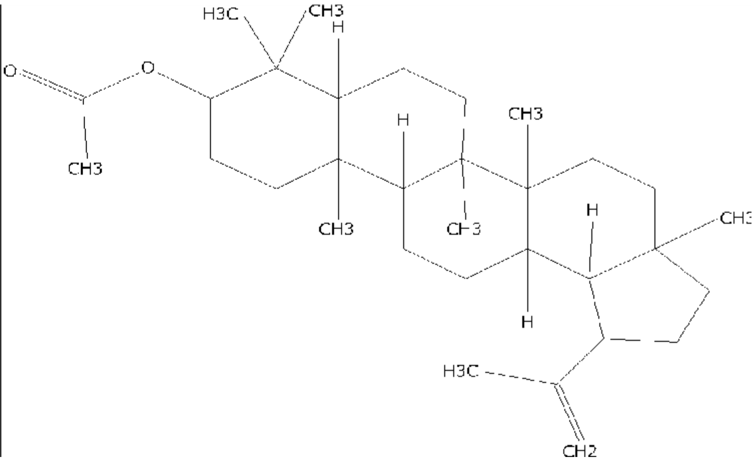


 Generated at 5:24 PM on 9/22/2025
