# Supplementary material for: GC-MS Guided Phytochemical Fingerprinting and Multi-Target Therapeutic Evaluation of Ixora chinensis Lam. Leaves: Insights into Its Hypoglycemic and Analgesic Activities
Source: Biology (Basel). 2026 Apr 8;15(8):592. doi: 10.3390/biology15080592 (PMC13114147; doi:10.3390/biology15080592)
Supplement: Supplementary file 1 [file biology-15-00592-s001.zip › biology-4239433-supplementary/biology-4239433-Supplementary file 2.pdf]

GC-MS Guided Phytochemical Fingerprinting and Multi-Target Therapeutic Evaluation of *Ixora chinensis* Leaves: Insights into Its Hypoglycemic and Analgesic Activities

Joy Baisnab [1], Md. Saiful Islam [2], Md Reduanul Haque Kavey [1], S. M. Yasin Shourav [3], Md. Riaz Hosen [1], Md. Faysal Abid [1], Shaikh Shahinur Rahman [4], Anuwatchakij Klamrak [5], Arunrat Chaveerach [6], Sakda Daduang [5,*], Md. Rasul Karim [1,*]

1. Department of Pharmacy, Islamic University, Kushtia, Kushtia-7003, Bangladesh;
2. Pharmaceutical Sciences Research Division, BCSIR Dhaka Laboratories, Bangladesh Council of Scientific and Industrial Research (BCSIR), Dhaka-1205, Bangladesh;
3. Department of Pharmaceutical Technology, Faculty of Pharmacy, University of Dhaka, Dhaka-1000, Bangladesh;
4. Department of Applied Nutrition and Food Technology, Islamic University, Kushtia, Kushtia-7003, Bangladesh;
5. Division of Pharmacognosy and Toxicology, Faculty of Pharmaceutical Sciences, Khon Kaen University, Khon Kaen 40002, Thailand;
6. Department of Biology, Faculty of Science, Khon Kaen University, Khon Kaen 40002, Thailand;

*Correspondence: mrk.kamol@gmail.com (MRK); sakdad@kku.ac.th (SD)

ORCID ID: https://orcid.org/0009-0001-9247-809X

Table S1: Molecular docking binding affinities (kcal/mol) of all screened phytocompounds identified from Ixora chinensis against peripheral analgesic, central analgesic, and hypoglycemic target proteins.

SL No.	PubChem CID	COX-1 (3N8Y)	AMPK (4RER)	μ-opioid (5C1M)	COX-2 (5F19)	α-amylase (1HNY)	α-glucosidase (3TOP)
A1	561987	-8.6	-8	-8.1	-8.1	-7.4	-7.5
A2	37497	-6.4	-6	-5.7	-6.8	-6.4	-6.4
A3	8488	-6.1	-5.8	-5.2	-5.7	-5.1	-6.1
A4	572926	-7.8	-6.7	-7	-7.8	-7	-7.4
A5	598567	-8.2	-8.9	-8.6	-8.2	-7.9	-7.6
A6	210894	-7.9	-7.2	-8.8	-8.8	-7.6	-7.4
A7	19754692	-5.7	-5.8	-5.3	-6.4	-5.3	-5.6
A8	566122	-5.4	-4.9	-5	-5.3	-4.8	-4.6
P1	76157	-6.4	-6	-6	-6.3	-5.4	-6.8
P2	604963	-6.1	-6.7	-6.4	-5.5	-5.7	-6.4
P3	57188	-6.3	-6.1	-6.5	-5.8	-5.8	-5.8
P4	7311	-6.1	-6.3	-6.5	-6.3	-6.4	-6.3
P6	586309	-6.8	-6.9	-6	-7.1	-6.1	-6.7
P7	66344	-6.5	-6.5	-6.3	-6.8	-5.6	-6.8
P8	606915	-8.1	-8.4	-8.1	-8.2	-7.6	-7.7
P9	94310	-5.8	-6.3	-6.7	-7	-5.6	-6.8
P10	15532447	-6.8	-6.2	-5.9	-6.2	-5.6	-6.5
P11	65057	-8.3	-8.4	-8.2	-8.5	-8	-8.3
P12	221645	-7	-6.6	-5.9	-7.1	-5.7	-6.8
P13	576674	-5.8	-6.3	-6.5	-7.6	-5.8	-6.7
P14	62603	-6.9	-7.1	-5.9	-7.1	-6.4	-6.9
E1	91717683	-6.1	-5.6	-6.4	-7.8	-5.5	-5.6
E2	227859	-6	-5.4	-5.4	-6.4	-5.1	-6.3
E3	6423506	-7.7	-7	-7	-7.5	-6.3	-7.5
E4	25775	-5.4	-5.2	-4.6	-5.1	-4.6	-5
T1	100332	-6.9	-6.4	-6.5	-6.1	-6.5	-5.8
T2	574441	-5.4	-5.8	-5.5	-6.5	-5.2	-6.1
T3	5280435	-6.1	-5.7	-5.9	-5.5	-5.4	-6
T4	545303	-7.3	-7.6	-6.3	-8.1	-6.6	-7.1
T5	5352709	-4.8	-4.6	-6	-5.3	-5.2	-6
T6	556530	-5.7	-6	-5.9	-6.7	-5.6	-5.9
T7	523968	-5.8	-5.8	-5.5	-6.3	-5.2	-6.3
T8	1254	-6.7	-5.8	-5.9	-6.4	-5.8	-6.8

T9	140213	-5.2	-5.1	-5	-4.7	-4.4	-5.7
T11	550119	-5.4	-6	-6.5	-7.2	-5.5	-5.8
S1	6421694	-6.1	-5.8	-6.2	-6.2	-5.5	-5.6
S2	6421380	-5.3	-4.7	-5.2	-5.1	-4.6	-4.8
S3	6421048	-5.6	-4.6	-5.4	-5.8	-4.5	-5.4

Table S2: Detailed molecular interactions between COX-1 (3N8Y) and the phytoconstituents A1 and P11, showing interacting amino acid residues, interaction types, distances (Å), and interaction categories.

Protein	Ligands	Amino Acid Residues (Distances: Å)	Types	Category
3N8Y	A1	GLY526 (3.137), PHE529 (3.769)	Carbon Hydrogen Bond	Hydrogen
		VAL349 (3.632), VAL349 (3.859), ALA527 (3.912). TYR348 (3.616)	Pi-Sigma	Hydrophobic
		PHE381 (4.742)	Pi-Pi T-shaped	
		VAL349 (4.701), ALA527 (3.750), LEU531 (4.281)	Alkyl	
		LEU531 (5.179)	Pi-Alkyl	
		TYR348 (5.909)	Pi-Sulfur	Other
	P11	ALA199 (5.402), LEU390 (5.289), MET391 (5.03)	Alkyl	Hydrophobic
		HIS207 (4.359), HIS386 (4.433), HIS388 (4.994)	Pi-Alkyl	
	Control (Diclofenac)	ARG374(2.204), ARG376(2.02)	Conventional Hydrogen Bond	Hydrogen
		PHE142(2.955)	Halogen (Cl, Br, I)	Halogen
		ARG374(4.75)	Pi-Cation	Electrostatic
		VAL145(3.752)	Pi-Sigma	Hydrophobic
		VAL145(4.827)	Alkyl	
		VAL145(5.265, 5.448)	Pi-Alkyl	

Table S3: Detailed molecular interactions between COX-2 (5F19) and the phytoconstituents A6 and P11, showing interacting amino acid residues, interaction types, distances (Å), and interaction categories.

Protein	Ligands	Amino Acid Residues (Distances: Å)	Types	Category
COX-2 (5F19)	A6	GLY45 (3.027), GLY45 (2.919)	Conventional Hydrogen Bond	Hydrogen
		CYS41 (3.492)	Carbon Hydrogen Bond	
		CYS36 (4.585), CYS47 (5.301), LEU152 (4.88), PRO153 (4.891), PRO156 (4.780), ARG469 (5.006)	Alkyl	Hydrophobic
		PRO153 (4.437)	Pi-Alkyl	
	P11	PHE529 (4.960)	Pi-Anion	Electrostatic
		ALA527 (3.920)	Pi-Sigma	Hydrophobic
		VAL349 (4.423), VAL523 (5.088), ALA527 (5.094), LEU534 (4.90)	Pi-Alkyl	
		GLY526 (4.294), ALA527 (4.459)	Amide-Pi Stacked	
		MET522 (5.895)		Other
	Control (Diclofenac)	LEU531(2.886, 2.737)	Conventional Hydrogen Bond	Hydrogen
		TRP387(5.481)	Pi-Pi T-shaped	Hydrophobic
		VAL349(5.135), VAL523 (4.61), ALA527(4.304)	Pi-Alkyl	
		MET522(5.969)	Pi-Sulfur	Other

Table S4: Detailed molecular interactions between μ-opioid (5C1M) and the phytoconstituents A6 and A5, showing interacting amino acid residues, interaction types, distances (Å), and interaction categories.

Protein	Ligands	Amino Acid Residues (Distances: Å)	Types	Category
μ-opioid (5C1M)	A6	HIS54 (2.214), ASP147 (2.298)	Conventional Hydrogen Bond	Hydrogen
		ASP147 (3.458)	Carbon Hydrogen Bond	
		VAL143 (5.49), ILE144 (4.629), ILE296 (4.63), ILE322 (5.207)	Alkyl	Hydrophobic
		TYR326 (4.783)	Pi-Alkyl	
		HIS54 (4.251)	Pi-Pi Stacked	
	A5	GLN124 (2.634),	Conventional Hydrogen Bond	Hydrogen
		VAL236 (5.130), VAL300 (4.449), VAL236 (4.265), VAL300 (3.889)	Alkyl	Hydrophobic
		ILE296 (5.380), ILE322 (5.398), ILE344 (4.67)	Pi-Alkyl	
		TYR148 (4.970)	Pi-Pi Stacked	
	Control	TYR148(2.803)	Conventional Hydrogen Bond	Hydrogen
		VAL300(3.984)	Pi-Sigma	Hydrophobic
		VAL300(5.216)	Alkyl	
		VAL236 (4.937), ILE296 (5.481), HIS54(5.427)	Pi-Alkyl	

Table S5: Detailed molecular interactions between AMPK (4RER) and the phytoconstituents A5 and P8, showing interacting amino acid residues, interaction types, distances (Å), and interaction categories.

Protein	Ligands	Amino Acid Residues (Distances: Å)	Types	Category
AMPK (4RER)	A5	LEU24 (3.394)	Carbon Hydrogen Bond	Hydrogen
		LEU24 (3.767), LEU24 (3.595), LEU148 (3.96), LEU148 (3.771)	Pi-Sigma	Hydrophobic
		LEU24 (4.11)	Alkyl	
		ALA45 (4.966), LEU148 (5.395), VAL32 (5.112), LYS47 (4.97), ALA158 (4.753)	Pi-Alkyl	
	P8	LEU24 (3.511), VAL32 (3.516), LEU148 (3.856), LEU148 (3.916)	Pi-Sigma	Hydrophobic
		MET95 (5.209), LEU148 (4.858). ALA158 (3.542), LEU24 (5.27), VAL32 (3.785), ALA45 (3.484)	Alkyl	
		ALA45 (4.623)	Pi-Alkyl	
	Control	SER260 (2.568), ARG258 (2.79), ALA259 (2.902), SER260 (2.395), PRO255 (2.119)	Conventional Hydrogen Bond	Hydrogen
		MET256 (3.485)	Carbon Hydrogen Bond	
		ASP263 (5.375)	Attractive Charge	Electrostatic

Table S6: Detailed molecular interactions between α-amylase (1HNY) and the phytoconstituents P11 and A5, showing interacting amino acid residues, interaction types, distances (Å), and interaction categories.

Protein	Ligands	Amino Acid Residues (Distances: Å)	Types	Category
α-amylase (1HN9)	P11	GLN63 (2.391)	Conventional Hydrogen Bond	Hydrogen
		TRP59 (5.317, 4.081, 4.081), TRP62 (4.366)	Pi-Pi Stacked	Hydrophobic
	A5	GLN63 (2.382)	Conventional Hydrogen Bond	Hydrogen
		ASP300 (3.793), ASP197 (3.447)	Carbon Hydrogen Bond	
		LEU162 (5.443), ALA198 (3.881)	Alkyl	Hydrophobic
		LEU162 (5.229)	Pi-Alkyl	
	Control	GLY309 (2.353), GLN302 (2.401), THR314 (2.552), ILE312 (1.930)	Conventional Hydrogen Bond	Hydrogen
		ASP317(4.012), ASP317 (2.059)	Attractive Charge	Electrostatic

Table S7: Detailed molecular interactions between α-glucosidase (3TOP) and the phytoconstituents P11 and P8, showing interacting amino acid residues, interaction types, distances (Å), and interaction categories.

Protein	Ligands	Amino Acid Residues (Distances: Å)	Types	Category
α-glucosidase (3TOP)	P11	ASP1157(4.012), ASP1526 (4.718)	Pi-Anion	Electrostatic
		TRP1355 (5.116), TYR1251 (4.976)	Pi-Pi T-shaped	Hydrophobic
	P8	TRP1749 (2.319)	Conventional Hydrogen Bond	Hydrogen
		PRO1658 (3.838)	Pi-Sigma	Hydrophobic
		TRP1749 (4.627)	Pi-Pi T-shaped	
		LYS1625 (3.489), LEU1622 (5.353), VAL1631 (4.216), PRO1658 (4.257)	Alkyl	
		LYS1625 (5.310), PRO1658 (4.975)	Pi-Alkyl	
	Control	ASP1526 (2.702), ASP1584 (2.343), ASP1420 (2.895), ASP1420 (2.687)	Conventional Hydrogen Bond	Hydrogen
		ASP1526 (3.784)	Carbon Hydrogen Bond	
		ASP1279 (2.670)	Salt Bridge	
		ASP1279 (4.570), ASP1420 (4.94), ASP1526 (4.069), ASP1279 (2.805), ASP1420 (4.944)	Attractive Charge	Electrostatic

Table S8: Evaluation of the Central analgesic activity of *Ixora chinensis* by tail flicking method

Analysis of data obtained after 30 minutes

Animal Group	Immersion time Count						Mean ± SEM	SD	% Elongation
	M-1	M-2	M-3	M-4	M-5	M-6			
CTL	1.63	1.27	1.82	1.19	2.11	1.66	1.6133±0.1401	0.3432	
Morphine	4.30	4.35	4.40	4.45	4.38	4.40	4.3800±0.0208	0.0510	171.49***
NHF-200	1.76	1.74	1.80	1.79	1.76	1.88	1.7883±0.0204	0.0500	10.84
CF-200	1.67	1.32	1.72	1.57	1.90	2.13	1.7183±0.1134	0.2777	6.51
EAF-200	1.98	1.63	1.59	1.71	1.47	1.38	1.6767±0.0642	0.1572	3.92
AQF-200	1.31	1.76	1.70	1.77	1.71	1.97	1.7033±0.0882	0.2161	5.58
NHF-400	1.89	1.85	2.81	1.73	1.71	1.09	1.8467±0.2262	0.5540	14.67
CF-400	1.95	1.49	1.74	2.45	2.18	1.88	1.9483±0.1371	0.3357	20.76
EAF-400	1.72	1.32	1.67	1.57	1.90	2.13	1.7183±0.1134	0.2777	6.51
**AQF-400**	1.27	1.85	1.97	2.67	1.85	1.49	1.8500±0.1960	0.4802	14.67

Analysis of data obtained after 60 minutes

Animal Group	Immersion time Count						Mean ± SEM	SD	% Elongation
	M-1	M-2	M-3	M-4	M-5	M-6			
CTL	1.27	1.85	1.97	2.67	1.49	1.60	1.8083±0.2004	0.4909	
Morphine	6.90	6.75	6.80	6.65	6.72	6.80	6.7700±0.0346	0.0849	274.38***
NHF-200	2.25	2.26	2.23	2.22	2.30	2.25	2.2517±0.0114	0.0279	24.52
CF-200	4.12	4.02	3.03	4.56	3.71	2.62	3.6767±0.2961	0.7253	103.32***
EAF-200	2.12	1.67	1.76	1.73	1.93	2.17	1.8967±0.0863	0.2124	4.88
AQF-200	1.77	2.34	1.87	1.99	1.90	2.08	3.3017±0.0855	0.2094	82.58***
NHF-400	2.20	2.22	2.25	2.30	2	2.25	2.2033±0.0429	0.1052	21.84
CF-400	8.13	8.79	8.07	7.84	9.88	7.71	4.5700±0.2050	0.5021	152.72***
EAF-400	1.95	1.89	2.21	1.77	2.39	2.41	2.1033±0.1107	0.2712	16.31
AQF-400	4.12	4.56	4.66	4.26	4.75	3.59	4.3233±0.1765	0.4323	139.08***

Analysis of data obtained after 90 minutes

Animal Group	Immersion time Count						Mean ± SEM	SD	% Elongation
	M-1	M-2	M-3	M-4	M-5	M-6			
**CTL**	1.45	1.93	1.77	2.09	1.88	1.97	1.8483±0.0905	0.2217	
Morphine	7.60	7.65	7.70	7.55	7.63	7.65	7.6300±0.0208	0.0510	312.81***
**NHF-200**	1.90	1.85	1.92	1.95	1.85	1.89	1.8933±0.161	0.0393	2.44
**CF-200**	4.24	3.44	4.63	4.16	4.04	3.30	3.9683±0.2065	0.5058	114.70***
**EAF-200**	2.13	2.06	3.41	1.98	1.15	1.17	1.9833±0.3376	0.8269	7.30
**AQF-200**	4.26	4.06	4.00	3.56	4.08	4.19	4.0250±0.1006	0.2464	117.767***
**NHF-400**	1.95	1.98	1.97	1.90	1.92	1.95	1.9450±0.123	0.0302	5.24
**CF-400**	4.75	4.80	4.90	5.10	5.20	5.25	5.00±0.0866	0.2121	170.51***
**EAF-400**	1.24	3.02	2.03	1.56	1.71	2.62	2.0300±0.2754	0.6746	9.83
**AQF-400**	5.25	5.60	5.30	5.20	5	5.25	5.2667±0.0792	0.1941	184.94***

Table S9: Evaluation of Analgesic activity by intraperitoneal administration of 1% Acetic acid

Animal Group	Writhing Count						Mean ± SEM	SD	Writhing (%)	Inhibition
	M-1	M-2	M-3	M-4	M-5	M-6				
**CTL**	26	21	17	23	20	22	21.50±1.23	3.02	100	-
**STD**	8	6	5	6	7	7	6.50±0.43	1.05	30.23	69.77***
**NHF-200**	15	14	16	17	15	17	15.67±0.76	1.21	72.88	27.11**
**CF-200**	20	21	22	21	20	20	20.67±0.33	0.82	96.13	3.86
**EAF-200**	20	21	21	22	20	21	20.83±0.31	0.75	96.88	3.11
**AQF-200**	17	19	17	16	15	20	17.33±0.76	1.86	80.60	19.40*

Animal Group	Writhing Count						Mean ± SEM	SD	Writhing (%)	Inhibition
	M-1	M-2	M-3	M-4	M-5	M-6				
**CTL**	26	21	17	23	20	22	21.50±1.23	3.02	100	-
**STD**	8	6	5	6	7	7	6.50±0.43	1.05	30.23	69.77***
**NHF-400**	7	8	8	7	9	7	7.67±0.33	0.82	35.67	64.33***
**CF-400**	19	20	21	20	19	21	20.67±0.33	0.89	93.02	6.97
**EAF-400**	19	21	20	19	21	18	19.67±0.49	1.21	91.48	8.51
**AQF-400**	8	10	8	10	9	10	9.17±0.40	0.98	42.65	57.35***
